# Structural model of dodecameric heat-shock protein Hsp21: Flexible N-terminal arms interact with client proteins while C-terminal tails maintain the dodecamer and chaperone activity

**DOI:** 10.1074/jbc.M116.766816

**Published:** 2017-03-21

**Authors:** Gudrun Rutsdottir, Johan Härmark, Yoran Weide, Hans Hebert, Morten I. Rasmussen, Sven Wernersson, Michal Respondek, Mikael Akke, Peter Højrup, Philip J. B. Koeck, Christopher A. G. Söderberg, Cecilia Emanuelsson

**Affiliations:** From the Departments of ‡ Biochemistry and Structural Biology and; ‖ Biophysical Chemistry and; the ** MAX IV Laboratory, Lund University, SE-221 00, Lund, Sweden,; the § School of Technology and Health, KTH/Royal Institute of Technology and Department of Biosciences and Nutrition, Karolinska Institutet, SE-171 77 Stockholm, Sweden, and; the ¶ Department of Biochemistry and Molecular Biology, University of Southern Denmark, 5230 Odense, Denmark

**Keywords:** aggregation, Arabidopsis thaliana, chloroplast, cryo-electron microscopy, homology modeling, molecular chaperone, oligomerization, protein cross-linking, small-angle X-ray scattering (SAXS), structural model

## Abstract

Small heat-shock proteins (sHsps) prevent aggregation of thermosensitive client proteins in a first line of defense against cellular stress. The mechanisms by which they perform this function have been hard to define due to limited structural information; currently, there is only one high-resolution structure of a plant sHsp published, that of the cytosolic Hsp16.9. We took interest in Hsp21, a chloroplast-localized sHsp crucial for plant stress resistance, which has even longer N-terminal arms than Hsp16.9, with a functionally important and conserved methionine-rich motif. To provide a framework for investigating structure-function relationships of Hsp21 and understanding these sequence variations, we developed a structural model of Hsp21 based on homology modeling, cryo-EM, cross-linking mass spectrometry, NMR, and small-angle X-ray scattering. Our data suggest a dodecameric arrangement of two trimer-of-dimer discs stabilized by the C-terminal tails, possibly through tail-to-tail interactions between the discs, mediated through extended I*X*V*X*I motifs. Our model further suggests that six N-terminal arms are located on the outside of the dodecamer, accessible for interaction with client proteins, and distinct from previous undefined or inwardly facing arms. To test the importance of the I*X*V*X*I motif, we created the point mutant V181A, which, as expected, disrupts the Hsp21 dodecamer and decreases chaperone activity. Finally, our data emphasize that sHsp chaperone efficiency depends on oligomerization and that client interactions can occur both with and without oligomer dissociation. These results provide a generalizable workflow to explore sHsps, expand our understanding of sHsp structural motifs, and provide a testable Hsp21 structure model to inform future investigations.

## Introduction

The small heat-shock protein (sHsp)^4^ chaperones play important roles in the cell by preventing aggregation of destabilized proteins (1–3). Indeed, a number of human diseases have been associated with malfunctioning sHsps (4). Some sHsps are constitutively expressed, and some are also stress-induced; in heat-stressed cells, the sHsps can be among the most highly up-regulated proteins (5). In plants, the sHsps confer an especially pronounced and important part of the stress response, with several sHsp paralogues being present in different cell compartments, heat-induced or developmentally regulated (6–8). The sHsps rapidly sequester destabilized client proteins, also referred to as substrate proteins (as referred to here hereafter), thereby overcoming the kinetic competition with aggregation (9). The sHsps typically act on early unfolding intermediates (10) and can capture unfolded conformations present for only a small fraction of the time. A common feature of sHsps, considered crucial for their chaperone activity, is their assembly into oligomeric structures, which are undergoing rapid subunit exchange (11–13). Yet it is not fully understood how these molecular features govern function or how the structural properties mediate the dynamic nature of the oligomer (3).

There are three structurally and functionally distinct regions in an sHsp subunit: (i) the conserved α-crystallin domain (ACD) that defines the family of sHsps and is shared by all members; (ii) the C-terminal region (CTR), with a conserved (I/V)*X*(I/V) motif (14); and (iii) the N-terminal region (NTR), which differs considerably in length and composition between the different sHsps. A picture is emerging in which the signature ACD acts as a scaffold in all sHsps, whereas the NTR and CTR have evolved to be structurally and functionally different, within and between species (2).

The ACD forms a β-sandwich fold, composed of two antiparallel sheets of three and four β-strands. The sHsps assemble into oligomers from an underlying dimeric structure. It has been reported for different sHsps that both the NTR and CTR affect oligomerization (15–21). In plants and other non-metazoan sHsps, the dimers are formed by domain swapping in which there is a strand exchange, involving β6 and also the loop between β5 and β7, between the two subunits in a dimer.

The CTR contributes to stabilization of the oligomers. In the human sHsp αB-crystallin, which, like several other mammalian sHsps, exists as a highly polydisperse mixture with a range of oligomeric forms containing between ∼10 and 40 subunits, each subunit donates and receives a CTR tail from neighboring subunits. It was early recognized by NMR that the long CTR tails (23 amino acids in length) are flexible and able to interact with substrates (22, 23) and more lately that their dynamics, followed as the peaks corresponding to Ile^159^/Ile^161^ in the (I/V)*X*(I/V) motif, contribute to the oligomer polydispersity (24). A plant sHsp, Hsp16.9 from *Triticum aestivum*, was found to be monodisperse, and the structure has been resolved to atomic resolution (PDB code 1GME) (25). This shows a dodecamer, consisting of two hexameric discs, with CTR tails shorter than in αB-crystallin (13 amino acids in length), with the (I/V)*X*(I/V) motif in one subunit binding to the hydrophobic groove formed by the β4 and β8 strands of the ACD in another subunit. Such tail-to-groove interactions occur between neighboring dimers within the same disc and between the discs. This is the only high-resolution structure of a plant sHsp published so far (25).

The NTR is even more variable between different sHsps and less understood. Seven of eight available crystal structures (25–31) lack complete information on how they are organized, but they are assumed to be intrinsically disordered, at least in part. The crystal structures represent only snapshots of a single stable conformer of what is presumably a highly dynamic set of conformers, and highly flexible regions are not observed. Some reports suggest that the NTR is facing the oligomer interior (25–31).

The chloroplast-localized sHsp, referred to as Hsp21, evolved when the land plants developed in response to the selection pressure in a non-water environment (8, 32). In Hsp21, the NTR contains a unique set of methionines in an amphipathic α-helix motif (33). We have reported previously that Hsp21 plays a crucial role in resistance to heat and oxidative stress in *Arabidopsis thaliana*, during which the Hsp21 oligomer undergoes a conformational change coupled to methionine oxidation (34), and that methionine oxidation is reversible through the action of a chloroplast-localized peptide methionine sulfoxide reductase (35). By nanoelectrospray mass spectrometry, we found that the Hsp21 oligomer is dodecameric (36) and, using negative-stain single-particle reconstruction EM at 15 Å resolution, that the two hexameric discs appeared to be rotated (37), compared with the Hsp16.9 structure (PDB code 1GME) (25). These two plant sHsp homologues show similarities as well as differences, such as the long NTR with the methionine-rich motif in Hsp21 and heat stability of the dodecamer. Hsp16.9 is a cytosolic class I sHsp. Both the chloroplast sHsp and the class I cytosolic sHps have evolved from class II cytosolic sHsps (8). In *Arabidopsis thaliana*, the most closely related paralogues to Hsp21 are the mitochondrial, thereafter the cytosolic class II, thereafter the cytosolic class I sHsps, to which the peroxisomal and endoplasmic reticulum-residing paralogues are most closely related. Hsp21 and Hsp16.9, which was used as a template for modeling, exist in two different cellular compartments; Hsp21 is found in the chloroplasts, where reactions involved in photosynthesis (*e.g.* carbon dioxide fixation and starch synthesis) dominate, whereas Hsp16.9 is found in the cytosol, where protein translation, glycolysis, and various transport processes occur. It is therefore expected that they should target and protect different proteins, such as key metabolic enzymes and translation factors (38).

The dynamic interaction between sHsps and substrate proteins is not completely understood and is presumably very different for different sHsps. Mass spectrometric approaches have shown that Hsp16.9 from *T. aestivum* and its orthologue Hsp18.1 from *Pisum sativum* undergo rapid subunit exchange, with dimeric species being the principal units of exchange (12). An extended study of the interactions between Hsp18.1 and three model substrates revealed over 300 different stoichiometries and extensive plasticity of the various sHsp-substrate complexes (39). Apparently, sHsp interactions with substrate proteins are very complex and call for complementary and non-conventional structural biology approaches.

Here we describe a structural model of Hsp21 based on a combination of homology modeling, cryo-EM, cross-linking mass spectrometry, NMR spectroscopy, site-specific mutagenesis, and small-angle X-ray scattering (SAXS). The resulting model suggests a division of labor in the chaperone activity of Hsp21, such that the CTR stabilizes the dodecamer, possibly in a tail-to-tail arrangement involving an extended (I/V)*X*(I/V) motif, while the NTR can interact with substrates on the outside of the dodecamer. The model does not exclude the possibility that dodecamers also can dissociate into subunits that interact with substrates.

## Results

### Structural model of Hsp21

**Figure 1.**
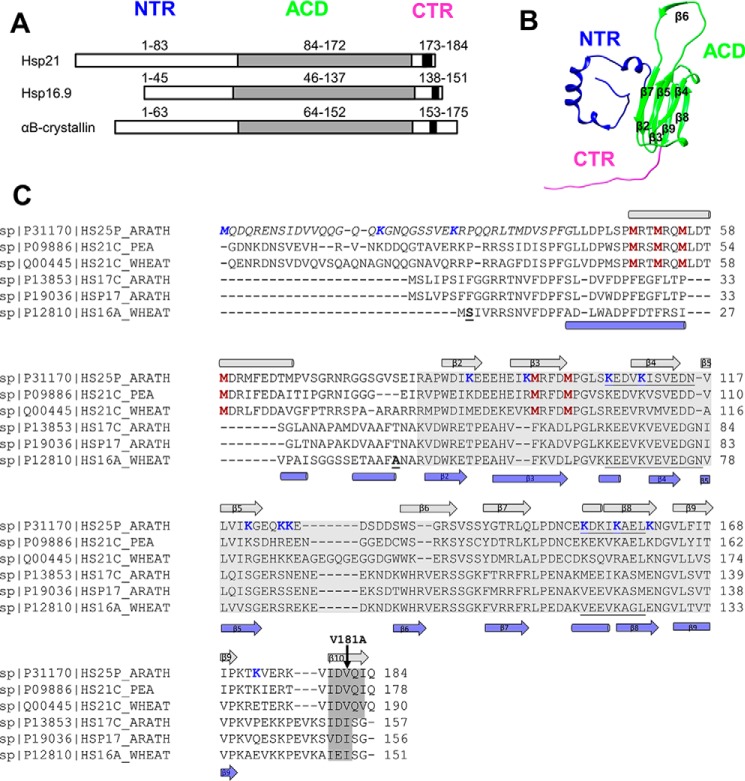
**Hsp21 has long N-terminal arms and short C-terminal tails with an extended I*X*V*X*I motif.** *A*, the sHsps share a folded ACD, a CTR, and an NTR with varying length and composition between sHsps. The NTR is longer in Hsp21 as compared with the structurally characterized homologues wheat Hsp16.9 and human αB-crystallin. The (I/V)*X*(I/V) motif, located in the CTR and shared by all sHsps, is extended to I*X*V*X*I in Hsp21. *B*, one subunit of the Hsp16.9 from *T. aestivum* (wheat); the dodecamer structure has been determined to atomic resolution (PDB code 1GME) (25) and was used as template to generate the structural model of Hsp21. *C*, sequence alignment of three chloroplast-localized (Uniprot codes P1170, P09886, and Q00445) and three cytosolic Cl I (Uniprot codes P13853, P19036, and P12810) plant sHsps. The (I/V)*X*(I/V) motif, which is conserved within all sHsps, and the extended I*X*V*X*I motif in Hsp21 are *shaded* in *dark gray*, and the ACD is *shaded* in *light gray*. In the Hsp21 sequence, the N-terminal part not included in the structural model, because the NTR is longer in Hsp21 than in Hsp16.9, is indicated in *italic type*; the six unique and conserved methionine residues are shown in *red*; the residues involved in cross-linking are shown in *blue*; and the V181A point mutation is indicated with a *black arrow*. In the Hsp16.9 sequence, the most N-terminal amino acids in the PDB file in chain A (Ser^2^) and chain B (Asn^43^) are indicated (*boldface type* and *underlined*), and the hydrophobic groove identified in Hsp16.9 (PDB code 1GME) (25) is *underlined* in both sequences. The secondary structure elements (α-helix (*cylinder*) and β-strand (*arrow*)), are indicated *above* and *below* the aligned sequences, with data for Hsp21 taken from secondary structure prediction (HH_pred, J-pred, NetSurfP, SCRATCH 3 class, Porter, and PsiPred) and for Hsp16.9 from the structure file (PDB code 1GME). The sequence names are the names of the FASTA files in the UniProt database, where *25* in the Hsp21 sequence from *A. thaliana* refers to the full mass (25 Da) of a protein subunit before the chloroplast presequence is cleaved off, resulting in a subunit mass of 21 kDa. It may be confusing that UniProt is using “25” in the sequence name for the *Arabidopsis* sequence and “21” for the orthologues in pea and wheat. There is only one known chloroplast sHsp homologue in known species so far.

A structural model of Hsp21 was generated using the crystal structure of the dodecameric Hsp16.9 (PDB code 1GME) (25) as a template for homology modeling. This template was chosen because Hsp16.9 has the highest sequence similarity to Hsp21 among the known structures (37). Each of the 12 subunits in Hsp21 has an NTR that is 41 residues longer than in the template (Fig. 1). Also, the first 42 residues in every second chain were not visible in the crystal structure; therefore, one should expect to find electron density in the cryo-EM map not unaccounted for when fitting the template-based structure model.

**Figure 2.**
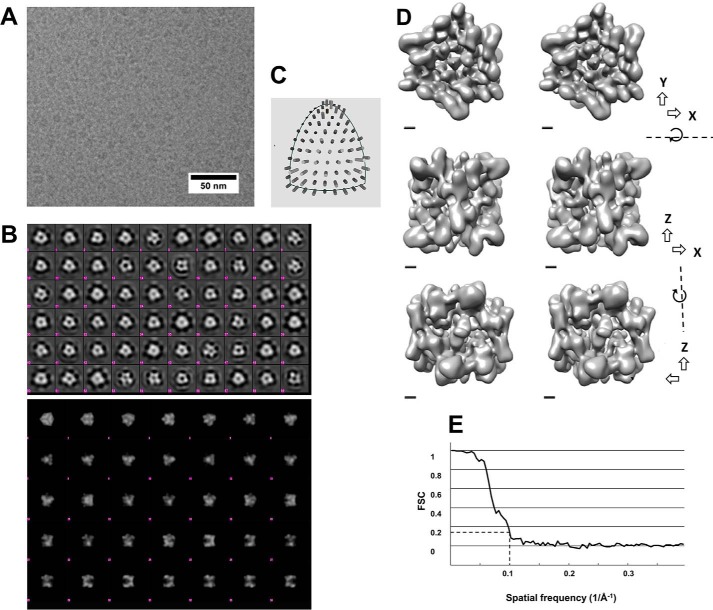
**Cryo-EM of Hsp21 to generate a density map at 10 Å resolution.** *A*, cryo-electron microscopy image of Hsp21 oligomers. The sample was plunge-frozen in liquid ethane and imaged in a JEOL JEM2100F electron microscope. Frame sets covering 2-s exposures were recorded on a DE-20 direct electron detector. The images were drift-corrected by aligning the acquired frames. *B*, *top*, class averages from a 2D classification of boxed out regions containing Hsp21 particles. There are several averages with approximate 3-fold and 2-fold symmetry (*e.g.* 3-fold: *row 5 column 9*, *row 2 column 3*; 2-fold: *row 2 column 5*). The classification was performed using Relion. The size of each *box* is ∼24 nm. *Bottom*, reprojections of the final 3D reconstruction with D3 symmetry low-pass filtered to 30 Å resolution. Several projections are similar to class averages in the *top panel*; for example, the side views in *row 5* can be recognized in class averages with approximate mirror symmetry in the *top panel* (*e.g. row 6 column 7*). *C*, distribution of angles according to EMAN2. *D*, 3D map of the Hsp21 oligomer. Surface-rendered views at contour level 4.5σ are illustrated in a *cross-eye stereo representation* using Chimera. The views are along the 3-fold axis (*top*), 2-fold axis from one side (*middle*), and 2-fold axis from the other side (*bottom*). *Scale bar*, 10 Å. *E*, Fourier shell correlation curve between reconstructions produced by splitting the data set into two halves. Both halves were reconstructed separately. The resolution of the final 3D map was calculated to 10.0 Å from the curve at FSC = 0.143.

Recombinantly expressed and purified Hsp21 protein was used to acquire single-particle cryo-EM data. The cryo-EM images showed well-separated particles around 10 nm in diameter (Fig. 2*A*). The first starting model using cryo-images was generated and refined without symmetry (C1). Because the result of this refinement exhibited a relatively clear D3 symmetry, we continued refinement of the reconstruction in D3 symmetry to a resolution of about 10 Å. Reference-free classification of Hsp21 particles from the cryo-EM data shows mainly classes with 2- and 3-fold symmetry (Fig. 2, *B* and *C*). Hence, further processing was performed in D3 symmetry. The reconstructed density map (Fig. 2*D*) of Hsp21, obtained from a data set of 18,407 particles, showed that the oligomer is ∼10 nm wide (3-fold axis view) and ∼9 nm high (2-fold axis view). The refined model and the start model are provided as Supplemental Information 1 and 2, respectively. The reconstructed map had a resolution of 10.0 Å using the “gold-standard” Fourier shell correlation (Fig. 2*E*). This cryo-EM reconstruction was not based on, but is compatible with, the 3D symmetry found in our previous reconstruction of the Hsp21 dodecamer that was based on negative stain-EM (37).

**Figure 3.**
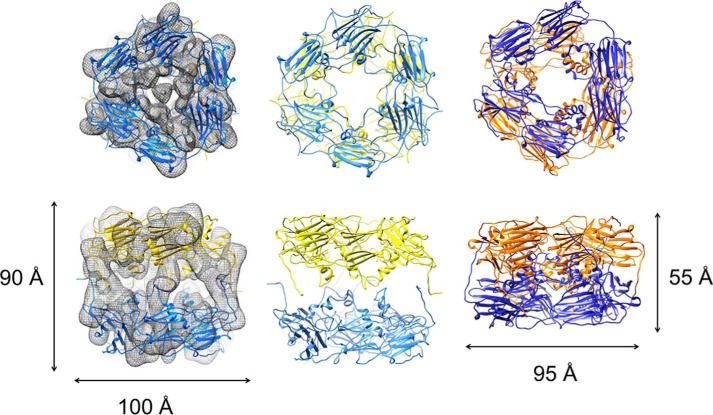
**To fit the Hsp21 structural model into the Hsp21 cryo-EM density map, the discs are rotated 30° and further separated by 35 Å.** *Left*, Hsp21 cryo-EM density map is shown with *partially transparent mesh surface representation* at contour level 4.5σ. The Hsp21 structural model is illustrated with the hexamer discs in *yellow* and *blue*. The views are along the 3-fold axis (*top*) and the 2-fold axis (*bottom*). The two discs were fitted separately to the map using the Fit in Map feature in Chimera. *Middle*, structural model of Hsp21. The Hsp21 structural model is illustrated with the discs in *yellow* and *blue*. The views are along the 3-fold axis (*top*) and the 2-fold axis (*bottom*). The top view (*top*) shows a relative rotation of the discs around the 3-fold axis of ∼30°, and the side view (*bottom*) demonstrates that the distance between the discs is extended by 35 Å in Hsp21, compared with the Hsp16.9 structure (PDB code 1GME) (25) that was used as a template for Hsp21 modeling. These differences in the Hsp21 model can also be described as an imaginary screw movement along the 3-fold axis (see supplemental Movie 1). *Right*, the structure of Hsp16.9 used as a template for Hsp21 modeling is illustrated with the discs in *yellow* and *blue*. The views are along the 3-fold axis (*top*) and the 2-fold axis (*bottom*).

The Hsp21 dimers were subsequently fitted into the cryo-EM map, using the double-disc arrangement with three dimers in each disc of the crystal structure of Hsp16.9 (PDB code 1GME) (25) as reference. To find the best fit between the model and the density map, each trimer-of-dimer disc (henceforth referred to only as “disc”) was docked individually onto the map. For optimum fitting of the Hsp21 density map, a relative *rotation* of the discs by about 30° was required, and the *distance expanded* between the discs by ∼35 Å (Fig. 3). The Hsp21 density map and the fitted Hsp21 structural model have been deposited in the EM data bank (EMDB accession number 3459) and the Protein Data Bank (PDB code 5MB8), respectively. The deposited .pdb file for the Hsp21 model and the validation report are provided as Supplemental Information 3 and 4, respectively. The shape of the cryo-EM map is a nearly quadratic, as seen from the side in the shape of a cage-like cylinder (height 90 Å), with a non-dense center. The top view looks similar to (and shows the same 30º rotation of discs as in) the previous negative-stain EM map (37). Yet the cylinder shape is a new feature of the cryo-EM map not seen in the negative-stain EM map, which was more compressed into a double-donut-like shape (height 55 Å) into which we could directly fit the structure 1GME. Such compression of the structure is not unusual in negative stain-EM, due to the drying effect. The shape obtained in cryo-EM probably better reflects the shape in solution, as also indicated by the good fit of the SAXS data (see below). The current structure model of Hsp21 suggests that the two discs are further separated from each other by 35 Å.

**Figure 4.**
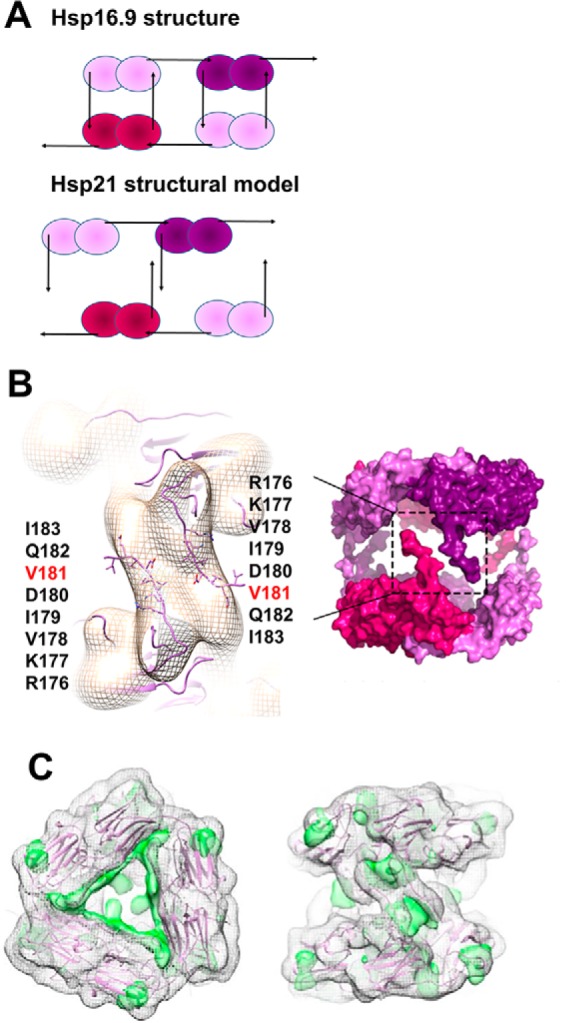
**Structural model of Hsp21; CTR interactions within and between the discs and NTR visible as positive difference density.** *A*, schematic outline of the difference between the Hsp16.9 dodecamer (25) and the Hsp21 dodecamer where the upper disc is rotated 30° and the distance between discs is expanded (see Fig. 3), such that the CTR tails from dimers in the upper and lower disc are close enough for tail-to-tail interactions and too distant for tail-to-groove interactions. *B*, *left*, magnification of the Hsp21 density map at 10.0 Å resolution with approximate fitting in Chimera of the CTR tails of the Hsp21 structure model and the amino acids in the extended I*X*V*X*I motif (^179^IDVQI^183^) and in the preceding sequence RKV presented as *sticks*. The extended I*X*V*X*I motif, as shown in Fig. 1, has a high β-strand propensity. Pairs of C-terminal tails may interact through hydrophobic interactions in β-sheets. *Right*, the rigid body used for simulation of the SAXS data (*i.e.* after removing all 82 amino acids in the NTR). *C*, positive difference density at 7σ depicted by a *green surface rendering*, following fitting of the atomic model Hsp16.9 (PDB code 1GME) (25), as seen from the top (*left*) and from the side approximately along a 2-fold axis (*right*), suggesting 3 + 3 major positive densities inside the dodecamer and 3 + 3 minor positive densities on the dodecamer outside. The fitted Hsp16.9 map is shown with a *mesh representation* at 2σ.

The rotation and expanded distance changes that were required to fit the Hsp21 structural model to the density map can be described as a movement along an imaginary screw centered at the 3-fold axis as visualized in the animation file (supplemental Movie 1). The rotation of the discs in Hsp21 brings three pairs of CTR tails from the upper and lower discs closer together (Fig. 4*A*). The CTR tail of the other subunit in each dimer connects to a neighboring dimer within the disc, in similar tail-to-groove interactions as in the Hsp16.9 crystal structure (PDB code 1GME) (25). In our model of the Hsp21 structure, the CTR tails from the upper and lower discs can make tail-to-tail interactions through the extended I*X*V*X*I motifs (Fig. 4*B*). The C-terminal tails are well-positioned in the density, and the relatively large volume occupied by only the C-terminal tails may reflect that they are quite mobile and flexible. The so-called (I/V)*X*(I/V) motif, shared among all sHsps, is in the Hsp21 orthologues extended to I*X*V*X*I (^179^IDVQI^183^), as shown in the alignment in Fig. 1. This extended I*X*V*X*I motif has a high β-strand propensity, and pairs of CTR tails may, for example, form antiparallel β-sheets. The resolution of the EM map is not sufficient to determine the exact position of the residues in the I*X*V*X*I motif, but the model clearly supports a role for these residues in making crucial contact between the discs through hydrophobic interactions. Indeed, a V181A point mutation disrupts the dodecameric arrangement, as described below.

Concerning the NTR in Hsp21, we calculated maps at different resolution levels from the atomic coordinates following fitting of Hsp16.9 discs as described above. A difference map showed positive continuous residual density extending from close to the central 3-fold axis to one of the subunit interfaces (Fig. 4*C*). Because the NTR arms of the fitted model are located toward the center of the structure, it is possible that this density in the difference map could correspond to the expected extended structure of the Hsp21 NTR. Apart from 3 + 3 major positive densities inside the dodecamer, there are also 3 + 3 minor positive densities toward the dodecamer outside, which could reflect the location of six NTR arms, which presumably are quite flexible, on the dodecamer outside.

### The structural model of Hsp21 is validated by cross-linking mass spectrometry

The structural model of Hsp21, presented above, was validated by cross-linking mass spectrometry, which plays an increasingly important role in integrative structural biology by providing distance constraints between residues within folded proteins (40, 41). A cross-linker specific to primary amines was used, and 23 cross-links were detected. As presented in Table 1 (and supported by the mass spectra in Supplemental Information 5), approximately half involve the peptide ^1^MQDQR^5^, as we observed before (42). The high incidence of the N-terminal peptide is expected because the cross-linking reaction involves the deprotonated amine, and the p*K_a_* value of the N-terminal amine is lower than that of the primary amine in a lysine side chain. Moreover, the ^1^MQDQR^5^ peptide is R-terminated and short, with a high proton affinity making it highly ionizable. The abundance of cross-links involving ^1^MQDQR^5^ presumably also reflects a high degree of flexibility of the NTR arms that enable them to cross-link to many other lysine residues. Due to this flexibility, cross-links involving ^1^MQDQR^5^ are not useful as distance constraints when validating the structural model.

**Table 1:** **Cross-links detected in Hsp21** MS/MS spectra for all detected cross-links and MS spectra for detected hybrid crosslinks are provided in Supplemental Information 5.

Lys A	Lys B	Peptide A	Peptide B	Distance Cα—Cα*^a^*	Distance within dimer Cα–Cα*^a^*	Distance within the disc Cα–Cα*^a^*	Distance between discs Cα–Cα*^a^*	Detected in subunit or dimer band or IS*^b^*	Detected as hybrid cross-link in IS*^c^*	Intra- or intersubunit*^d^*
				Å	Å	Å	Å			
**1**	**18**	**MQDQR**	**ENSIDVVQQGQQKGNQGSSVEK**					**IS**	**Y**	**4**
**1**	**18**	**MQDQR**	**ENSIDVVQQGQQKGNQGSSVEKRPQQR**					**IS**	**Y**	**4**
**1**	**27**	**MQDQR**	**GNQGSSVEKRPQQR**					**IG-M, IS**	**Y**	**2**
1	106	MQDQR	MRFDMPGLSKEDVK					IS	N	1
1	110	MQDQR	EDVKISVEDNVLVIK					IG-D	ND	3
**1**	**125**	**MQDQR**	**GEQKKEDSDDSWSGR**					**IG-M, IG-D, IS**	**Y**	**2**
**1**	**126**	**MQDQR**	**KEDSDDSWSGR**					**IG-M, IS**	**Y**	**2**
**1**	**157**	**MQDQR**	**IKAELK**					**IG-M, IS**	**Y**	**2**
**1**	**161**	**MQDQR**	**AELKNGVLFITIPK**					**IG-M, IG-D, IS**	**Y**	**2**
**1**	**173**	**MQDQR**	**TKVER**					**IG-M, IS**	**Y**	**2**
**27**	**121**	**GNQGSSVEKRPQQR**	**ISVEDNVLVIKGEQK**					**IS**	Y*	**4**
89	125	APWDIKEEEHEIK	GEQKKEDSDDSWSGR	32.9	20.4	29.0	52.0	IG-M	ND	5
**89**	**126**	**APWDIKEEEHEIKMR**	**KEDSDDSWSGR**	**35.3**	**18.9**	**32.6**	**49.2**	**IG-M, IS**	Y*	**5**
89	173	APWDIKEEEHEIK	TKVER	11.2	56.4	35.1	32.1	IS	N	1
96	126	APWDIKEEEHEIKMR	KEDSDDSWSGR	28.7	15.9	36.6	46.2	IG-M	ND	5
**106**	**161**	**FDMPGLSKEDVK**	**AELKNGVLFITIPK**	**9.3**	**16.0**	**48.3**	**58.3**	**IG-M, IS**	**Y**	**2**
121	126	ISVEDNVLVIKGEQK	KEDSDDSWSGR	17.0	27.0	29.6	35.1	IG-M	N	1
**121**	**173**	**ISVEDNVLVIKGEQK**	**TKVER**	**27.8**	**45.8**	**24.0**	**50.6**	**IS**	Y*	**5**
125	161	GEQKKEDSDDSWSGR	AELKNGVLFITIPK	17.2	16.1	45.1	52.0	IG-D	ND	3
126	157	KEDSDDSWSGR	IKAELKNGVLFITIPK	26.9	23.5	47.0	46.2	IG-M	ND	5
**126**	**161**	**KEDSDDSWSGR**	**AELKNGVLFITIPK**	**18.4**	**16.7**	**48.1**	**51.4**	**IG-D, IS**	Y*	**4**
153	157	LQLPDNCEKDK	IKAELK	9.3	44.8	33.6	58.6	IG-M	ND	5
157	173	IKAELK	TKVER	16.7	50.6	26.8	46.2	IG-M, IG-D	ND	5

*^a^* Calculated Cα–Cα distances for cross-linked lysines according to the Hsp21 structural model (within one subunit, between subunits within a dimer, between adjacent subunits in the same disc, and between the discs).

*^b^* Samples were analyzed to detect cross-links either after SDS-PAGE and in-gel digestion of bands corresponding to a cross-linked monomeric (IG-M) or dimeric (IG-D) subunit or without prior SDS-PAGE fractionation by in-solution digestion (IS). Cross-links detected in a band corresponding to a monomeric subunit are considered to be intrasubunit, not excluding the possibility that it can also be intersubunit.

*^c^* Sample was a 1:1 mixture of non-labeled and ^15^N-labeled Hsp21 subjected to in-solution digestion and analyzed for hybrid cross-links (*i.e.* cross-links between one unlabeled (^14^N) and one ^15^N-labeled peptide). Cross-links detected as hybrid cross-links (Y = yes, and Y* refers to an experiment where Hsp21 was mixed with Hsp21V^181A^) are considered to be intersubunit and are marked in boldface type. Also noted is if the cross-link was detected only as ^14^N-^14^N or ^15^N-^15^N (*i.e.* not as hybrid cross-links (*n* = no)) or not detected (ND).

*^d^* The following classifications are used concerning whether they are intra- or intersubunit: 1 = intrasubunit, 2 = intra- and intersubunit, 3 = intra- or intersubunit, 4 = inter- and possibly intrasubunit, 5 = intra- and possibly intersubunit.

**Figure 5.**
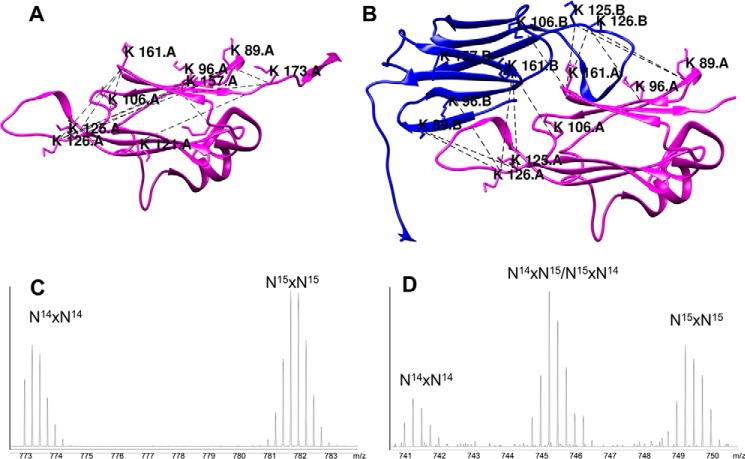
**Detected cross-links validate the structural model of Hsp21.** The detected cross-linked peptides are listed in Table 1. *A*, intrasubunit cross-links presented in one subunit of the Hsp21 structural model. *B*, intersubunit cross-links at the dimer interface presented in a dimeric subunit of the Hsp21 structural model with reciprocal swapping of the β6-strands into the β-sandwich of the neighboring subunit. *C*, MS spectrum after mixed isotope labeling, with the signature typical for when only ^14^N-^14^N or ^15^N-^15^N but no ^14^N-^15^N or ^15^N-^14^N peaks are detected as hybrid cross-linked peptides. This is evidence for intrasubunit cross-linking, in this example for Lys^121^–Lys^126^. *D*, MS spectrum after mixed isotope labeling, with the signature typical for when not only ^14^N-^14^N or ^15^N-^15^N but also ^14^N-^15^N and ^15^N-^14^N peaks are detected as hybrid cross-linked peptides. This is evidence for intersubunit cross-linking, in this example for Lys^126^–Lys^161^.

The distance constraints from all the other detected cross-links are compatible with, and therefore validate, the structural model of Hsp21, by the detected intrasubunit (Fig. 5*A*) and intersubunit cross-links at the dimer interface (Fig. 5*B*). To make this conclusion, the cross-links in Table 1 were first carefully assigned as intra- or intersubunit, and then the distances were evaluated in the structure model. As a threshold for the constraints, we used a Cα–Cα distance of 30 Å (43). To distinguish between intra- and intersubunit cross-links, two experimental approaches were used: (i) by analyzing cross-links in excised bands corresponding to cross-linked monomeric or dimeric subunit and (ii) by mixed isotope labeling and detection of hybrid cross-links. Based on whether the cross-link could be detected within a monomer band and inspection of the hybrid cross-link mass spectra, the detected cross-links were classified as shown in Table 1. A certain cross-link (*e.g.* Lys^106^–Lys^161^) is classified as 2 (intra- and intersubunit) when detected in the monomer band and as a hybrid cross-link with mass spectrum signature as in Fig. 5*C*. A cross-link (*e.g.* Lys^121^–Lys^126^) is classified as 1 (intrasubunit) when detected in the monomer band and as a hybrid cross-link with mass spectrum signature as in Fig. 5*D*, whereas a cross-link (*e.g.* Lys^96^–Lys^126^) is classified as 5 (intra- and possibly intersubunit) when detected within the monomer but so far in this data set not detected as a hybrid cross-link, permitting evaluation of its spectrum signature. The detected cross-links Lys^96^–Lys^126^, Lys^89^–Lys^125^, Lys^89^–Lys^126^, Lys^106^–Lys^161^, Lys^125^–Lys^161^, and Lys^126^–Lys^161^ are presumably all intersubunit cross-links at the dimer interface, and for Lys^106^–Lys^161^ and Lys^126^–Lys^161^, there is even proof in hybrid spectrum signatures. As seen in Table 1, all of the intrasubunit cross-links, except two, had Cα–Cα distances < 30 Å. The two exceptions were the cross-links Lys^89^–Lys^125^ and Lys^89^–Lys^126^, and they both involve the flexible loop region between β5 and β7 (see Fig. 1*C*). The cross-links Lys^106^–Lys^161^ and Lys^157^–Lys^173^ could be either intrasubunit or intersubunit and showed Cα–Cα distances < 30 Å within one subunit. Inspection of all possible distances showed that Lys^106^–Lys^161^ could also be an intersubunit cross-link within a dimer (Cα–Cα distance 16.0 Å), and Lys^157^–Lys^173^ could be an intersubunit cross-link between two dimer subunits in the same disc (Cα–Cα distance 26.8 Å). The cross-link Lys^126^–Lys^161^ could also be a cross-link between subunits within a dimer (Cα–Cα distance 16.7 Å). To summarize, all detected cross-links outside the highly flexible regions of Hsp21 were compatible with our structural model of the Hsp21 dodecamer.

### Flexible NTR arms on the outside of Hsp21 dodecamers detected by limited proteolysis and NMR

The positive densities in the cryo-EM difference maps (Fig. 4*C*) suggest that NTR arms of Hsp21 may be located on the outside of the core dodecamer. We investigated this issue further by limited proteolysis and by ^1^H-^15^N 2D NMR spectroscopy.

**Figure 6.**
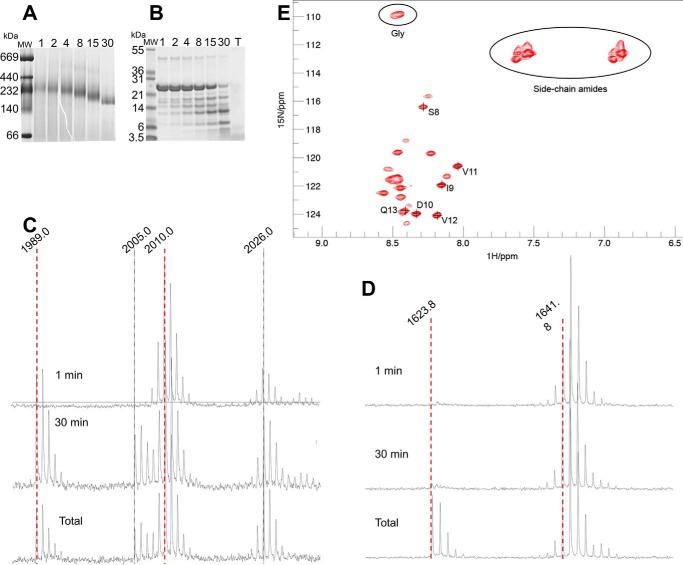
**Limited proteolysis and NMR provide further evidence for N-terminal arms on the outside of the Hsp21 dodecamer.** *A*, native PAGE; Hsp21 samples withdrawn during 1–30 min of limited proteolysis (trypsin/protein molar ratio 1:2,000), showing Hsp21 dodecamers with a gradual shift in mobility. *B*, SDS-PAGE; same samples as in *A* plus a total proteolysis sample (*T*), showing that there is only a small amount of uncleaved Hsp21 remaining after 30 min of limited proteolysis. *C*, NTR peptide; MALDI-MS spectra after 1 min, 30 min, and total proteolysis, showing a peptide from the NTR (amino acids 33–50, LTMDVSPFGLLPDPLSPMR_,_ *m*/*z* = 1,989.9), diluted with a ^15^N-labeled reference (*m*/*z* = 2,010.0). Peptide variants with oxidized methionine (Δ16 Da) are also visible at *m*/*z* = 2,005.0 and 2,026.0, respectively. Mass spectra for this peptide indicate that the NTR is completely cleaved after 30 min. *D*, ACD peptide; MALDI-MS spectra after 1 min, 30 min, and total proteolysis, showing a peptide from ACD (amino acids 84–96, APWDIKEEEHEIK_,_ *m*/*z* = 1623.8), diluted with a ^15^N-labeled reference (*m*/*z* = 1641.8). Mass spectra for this peptide indicate that the ACD is hardly cleaved after 30 min. *E*, ^1^H-^15^N HSQC spectra of the Hsp21 dodecamers with signals detected that are assigned to ^8^SIDVV^12^ in the NTR. The relative intensities of other signals were evaluated by integrating the cross-peaks using PINT (76) and comparing with the intensity of the well-resolved peak from Ser^8^.

Limited proteolysis showed that the NTR is rapidly degraded whereas Hsp21 remains dodecameric, in agreement with our previous data (44), and that all of NTR is degraded before ACD starts to be degraded (Fig. 6, *A–D*). This result indicates that the NTR behaves as an intrinsically disordered protein that is very prone to proteolysis and that the NTR is not primarily involved in maintaining the dodecamer.

Heteronuclear ^1^H-^15^N HSQC spectra acquired on the Hsp21 dodecamers at 20 °C show a set of ∼20 signals (Fig. 6*E*). Due to the large molecular size of the Hsp21 dodecamer, we do not expect to see any signals from well-ordered parts of the protein. Thus, the observed signals provide direct evidence that a subset of residues in Hsp21 are highly flexible. Using 3D ^1^H-^15^N NOESY-HSQC and ^1^H-^15^N TOCSY-HSQC spectroscopy, we could achieve residue-specific assignments for a subset of the observed peaks, namely the segment ^8^SIDVVQ^13^ (Fig. 6*E*). These results unequivocally show that this part of the NTR is flexible. In addition, the spectrum includes peaks from 2–3 glycine residues (overlapped cross-peaks at 8.45/109.8 ppm with a total intensity 2.4 times greater than that of Ser^8^), which are present only in the NTR (at positions Gly^15^, Gly^19^, and Gly^22^), but not in the CTR tail. The spectrum also includes at least 14 peaks from side-chain amide groups, which are prevalent (11 residues) in the NTR but occur only sparingly (2 residues) in the CTR. Because we observe only a single set of resonances for each residue, it is clear that flexible residues from the different subunits experience the same time-averaged molecular environment. Taken together, the NMR data provide strong evidence that several copies of the NTR segment are highly flexible and extend away from the dodecamer core.

### The V181A point mutation disrupts the Hsp21 dodecamer and chaperone activity

**Figure 7.**
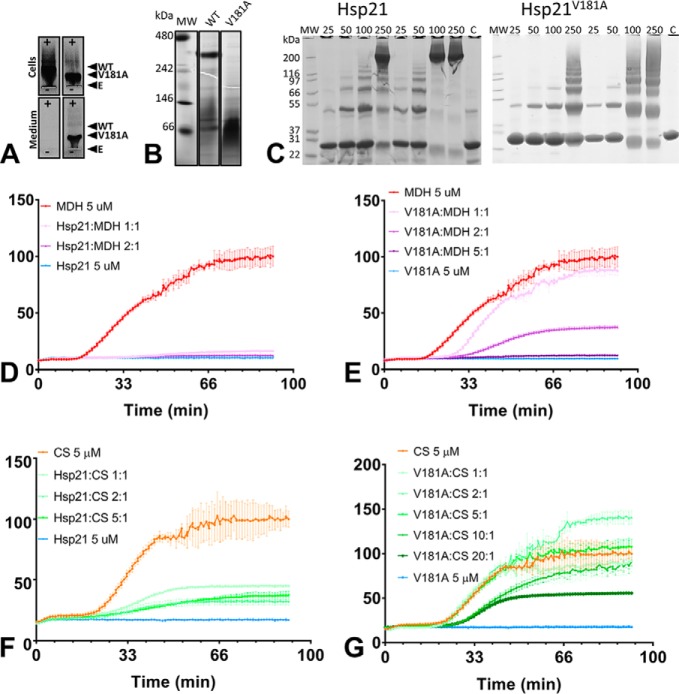
**The V181A mutational variant of Hsp21 is non-dodecameric and has decreased chaperone activity.** *A*, non-denaturing agarose gel electrophoresis. *Panels* show aliquots withdrawn from cells and medium before harvesting the cells; in each *panel pair* there is Hsp21 (*left*) and Hsp21^V181A^ (*right*). *Labels* indicate the positions of Hsp21 (*WT*) and Hsp21^V181A^ (*V181A*) and the entry point for loading (*E*). The image shows the overexpressed proteins as they migrate toward the anode (+) and that, already before purification, the mutational variant Hsp21^V181A^ is of smaller size compared with Hsp21 and that Hsp21^V181A^ leaks out into the medium. *C*, denaturing SDS-PAGE of Hsp21 WT and Hsp21^V181A^ mutational variant cross-linked with lysine-specific cross-linker at various protein concentrations (25–250 μm) at protein/cross-linker ratios of 1:10 and 1:60. *C*, control samples without cross-linker. A protein concentration of 250 μm Hsp21 corresponds to ∼5 mg/ml (calculated based on monomeric mass 21 kDa). *D*, chaperone activity of Hsp21, determined as its suppression of heat-induced aggregation of the model substrate protein MDH at molar ratios of 1:1 and 2:1. *E*, chaperone activity of Hsp21^V181A^ mutational variant, determined as its suppression of heat-induced aggregation of the model substrate protein MDH at molar ratios of 1:1, 2:1, and 5:1. *F*, chaperone activity of Hsp21, determined as its suppression of heat-induced aggregation of the model substrate protein CS at molar ratios of 1:1, 2:1, and 5:1. *G*, chaperone activity of Hsp21^V181A^ mutational variant, determined as its suppression of heat-induced aggregation of the model substrate protein CS at molar ratios of 1:1, 2:1, 5:1, 10:1, and 20:1.

To evaluate the dodecameric conformation and its functional importance in Hsp21, we created and analyzed a non-dodecameric mutational variant, V181A (Hsp21^V181A^). To design the mutant, we made a sequence alignment with cyanobacterial Hsp16.6, in which V143A substitution strongly reduced Hsp16.6 oligomerization (18), and found that the corresponding site for point mutation in Hsp21 would be Val^181^, which is in the very middle of the I*X*V*X*I motif, as seen in the alignment in Fig. 1*C*. Non-denaturing electrophoresis suggested that Hsp21^V181A^ was indeed non-dodecameric, as seen in the harvested bacterial extract after recombinant expression (Fig. 7*A*) and after purification (Fig. 7*B*), and cross-linking was used to compare the extent of oligomerization of Hsp21 and Hsp21^V181A^ using various concentrations and two different molar ratios of cross-linker to protein and SDS-PAGE analysis of the cross-linked proteins (Fig. 7*C*). The higher the protein concentration, and the higher the cross-linker/protein ratio, the more oligomeric bands are visible, with a clear difference between Hsp21 and Hsp21^V181A^. Hsp21 is mostly visible as a cross-linked dodecamer band, whereas Hsp21^V181A^ is not. At low concentrations, Hsp21^V181A^ is mostly found as bands corresponding to monomer or dimer subunits; at medium concentrations, it is also found as trimer subunits; and at the highest concentrations, it is present as oligomeric bands and a high molecular weight smear, but no dodecamer band at 240 kDa is seen. To estimate whether the Hsp21^V181A^ subunits were structurally intact and able to exchange subunits with Hsp21, we used mixed isotope labeling, and the hybrid cross-links detected (marked with asterisks in Table 1) show that Hsp21^V181A^ can exchange subunits with Hsp21 dodecamers. Specifically, the cross-link Lys^126^–Lys^161^, presumably between subunits within a dimer, suggests that not only dimers have exchanged but also monomeric subunits.

The chaperone activities of Hsp21 and Hsp21^V181A^ were compared by monitoring light scattering during heat-induced aggregation of two commonly used heat-sensitive model substrate proteins, malate dehydrogenase (MDH) and citrate synthase (CS) (Fig. 7). Hsp21 or Hsp21^V181A^ was incubated with model substrates at different molar ratios. The chaperone activity is manifested as a delay in the light-scattering increase and a lower final level of light scattering, the latter of which is taken as a measure of protection against aggregation. At a 1:1 (Hsp21/MDH) ratio, Hsp21 fully protected MDH from aggregation (Fig. 7*D*). Hsp21^V181A^ delayed aggregation of MDH at a molar ratio of 1:1 (Hsp21/MDH), but only a small fraction of MDH was protected, and it required a molar ratio of 5:1 (Hsp21/MDH) for full protection (Fig. 7*E*). Whereas the model substrate CS demanded higher chaperone/substrate molar ratios for delay and protection, the same distinct difference in chaperone activity was found for Hsp21 (Fig. 7*F*) and Hsp21^V181A^ (Fig. 7*G*). Thus, the chaperone activity is significantly decreased in the non-dodecameric Hsp21^V181A^. This is in agreement with what was previously observed with a corresponding point mutation in the cyanobacterial homologue Hsp16.6 (18).

### SAXS provides ensemble representations of Hsp21 dodecamers and the flexible NTR arms

Substrate interaction has been associated with the NTR arms, in sHsps generally (3) and in Hsp21 specifically (45). Yet there is limited structural information due to its high flexibility. In our structural model of Hsp21 (Fig. 3), only parts of the NTR were included because the NTR is 40 amino acids shorter in the template, and only 6 of the 12 NTR arms were visible in the Hsp16.9 crystal structure (PDB code 1GME) (25). Above, we demonstrated that the NTR is highly flexible, but the question remains as to how far the NTR arms might extend away from the dodecamer core. To address these points, we applied SAXS measurements interpreted using ensemble optimization to represent the average structure of the Hsp21 dodecamer, including the flexible NTR arms.

**Figure 8.**
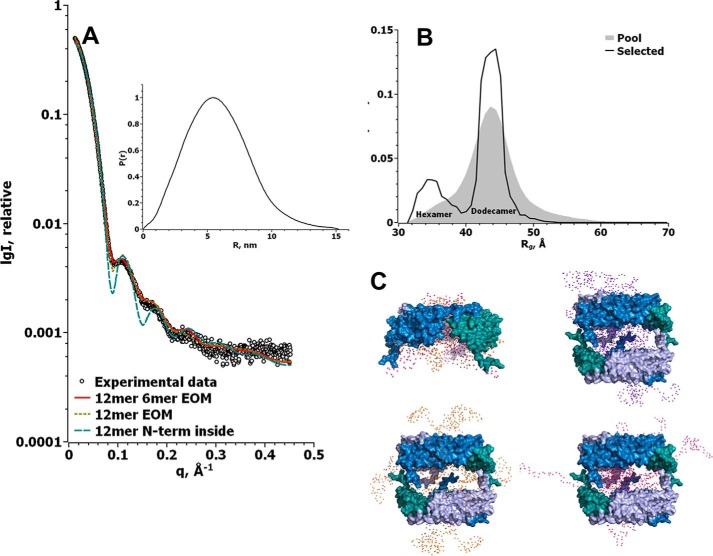
**Fit to SAXS data further validates the Hsp21 structure model based on cryo-EM and suggests that there are N-terminal arms that are flexible on the outside of the dodecamer.** *A*, Hsp21 wild-type SAXS data (*circles*) and the corresponding fits using a compact dodecamer with the N-terminal arms on the inside (*blue dashed line*, χ^2^ = 31.6 using CRYSOL), flexible extended dodecamers (*yellow dotted line*, modeled using EOM, χ^2^ = 5.52), and flexible extended hexamers and dodecamers (*red line*, modeled using EOM, χ^2^ = 1.88). The *inset* shows the Hsp21 wild-type distance distribution function calculated from the experimental SAXS data using GNOM. *B*, *R_g_* distribution for the random pool of 10,000 hexamers and 5,000 dodecamers that was used to fit the SAXS data in *A* is shown in the *gray area*. The *black line* shows the *R_g_* distribution of the optimized ensemble fitting our Hsp21 wild-type SAXS data. Whereas the selected hexamer distribution was overall compact, the selected dodecamer distribution was found at the center of the random pool. *C*, representative models from the selected pools of the hexamer and dodecamer, created by EOM. The rigid core is shown in a *surface representation*, where the three dimers of each disc are *colored* in *blue*, *light blue*, and *teal*, respectively, and the modeled flexible parts are shown as *spheres*. Two hexamer models are shown in *one image* because they were very similar, whereas the dodecamer models are shown as single objects to better see the flexible parts.

We collected SAXS data of Hsp21 at three different protein concentrations ranging from 2.5 to 10 mg/ml. No aggregation was observed and only small repulsion effects at high concentration; thus, we proceeded by merging the SAXS data from 5 and 10 mg/ml Hsp21 (Table 2 and Fig. 8). The values of radius of gyration (*R_g_*) and *D*_max_ were found to be 4.5 and 15.2 nm, respectively. Molecular mass estimates using Porod volume (*V_p_*) and DAMMIF volume (*V_a_*) were 316 and 272 kDa, respectively. We interpreted the SAXS data using the ensemble optimization method (EOM) (46, 47). To model the missing NTR arms of Hsp21, we used our structural model of the Hsp21 dodecamer, without the NTR, as a rigid body. A good fit to the experimental data was obtained assuming NTR arms on the outside (χ^2^ = 5.52), whereas modeling NTR arms on the inside resulted in a very poor fit (χ^2^ = 31.6). The best fit (χ^2^ = 1.88) was found using a pool of dodecamers and hexamers. Any other combination of oligomers (dimer, hexamer, and dodecamer) resulted in higher values for χ^2^ and visibly worse fits to the experimental SAXS data. Approximately 74% of the optimized ensemble was dodecamer, and 26% was hexamer. The dimer-hexamer-dodecamer pool gave the same results as hexamer-dodecamer, with no dimers in the optimized ensemble (results not shown). From the graph in Fig. 8*B*, it is apparent that the hexamer distribution was selected from the more compact part of the available random pool of 10,000 models, whereas the dodecamer distribution was selected more uniformly around the center of the available random pool of 5,000 models. Thus, we conclude that the NTR arms of Hsp21 are extending further away from the core oligomer and are more accessible to the surrounding solution in the dodecamer than in the hexamer, possibly due to space constraints. Fig. 8*C* shows representative models of the optimized ensemble as selected by the program GAJOE; this is also visualized in the animation file (supplemental Movie 2). This is a collection of images from the EOM simulations, which cannot be used to study clashes between the ACD and the modeled arms, and is provided merely because it does give an impression of the dynamic movement of the N termini. The good fit to the SAXS data (Fig. 8*A*) further validates the Hsp21 structural model based on cryo-EM data (Fig. 3).

**Table 2:** **SAXS data for Hsp21 and Hsp21^V181A^** Radius of gyration (*R_g_*), maximum size (*D*_max_), and Porod volume (*V_p_*) were calculated from SAXS data as described under “Experimental procedures.” Molecular mass estimates MM*_p_* and MM*_a_* were calculated by dividing *V_p_* by 1.6 and the DAMMIF model excluded volume by 2, respectively.

Sample	Concentration	Rg	*D*_max_	*V_p_*	MM*_p_*	MM*_a_*
	*mg/ml*	*nm*	*nm*	*nm^3^*	*kDa*	*kDa*
Hsp21WT	—*^a^*	4.48 ± 0.05	15.2	510	319	272
Hsp21WT	2.5	4.56 ± 0.05	15.5	522	326	
Hsp21WT	5	4.48 ± 0.05	15.5	506	316	
Hsp21WT	10	4.32 ± 0.05	14.0	485	303	
V181A	2.5	4.21 ± 0.07	17.5	116	73	79
V181A	5	4.10 ± 0.04	17.5	128	80	89
V181A	10	4.14 ± 0.02	17.0	143	90	101
V181A SEC^80^*^b^*		3.44 ± 0.06	14.0	113	70	75
V181A SEC^81^*^b^*		3.33 ± 0.05	13.0	113	71	74
V181A SEC^82^*^b^*		3.30 ± 0.04	13.5	113	71	44
V181A SEC^83^*^b^*		3.19 ± 0.05	11.5	108	67	68
V181A SEC^84^*^b^*		3.16 ± 0.06	11.3	103	64	61

*^a^* Merged from concentrations 5 and 10 mg/ml.

*^b^* From SEC; numbers refer to the retention time in minutes.

**Figure 9.**
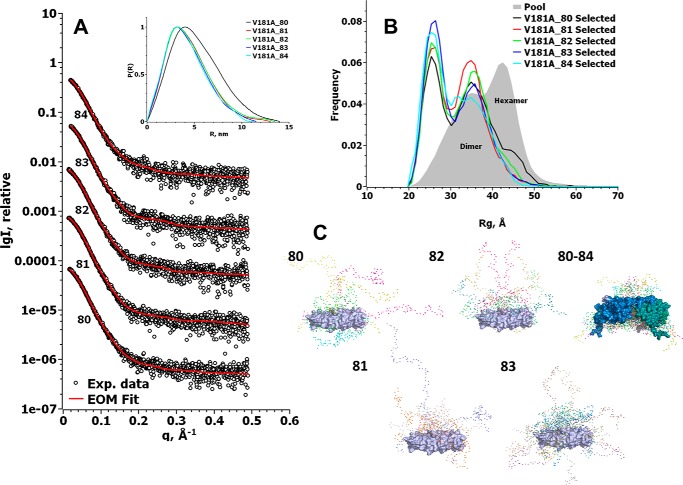
**Online SEC-SAXS shows that Hsp21^V181A^ mutational variant is a heterogeneous mixture of mainly dimers and some hexamers.** *A*, experimental SAXS data of Hsp21^V181A^ mutational variant after size-exclusion chromatography. The *number below* each *curve* represents the respective retention time in minutes on the column, and the *red line* is the EOM fit of the data using a pool of dimers and hexamers. The *inset* shows the Hsp21^V181A^ distance distribution function calculated from the experimental SAXS data using GNOM. *B*, *R_g_* distribution for the random pool of 10,000 dimers and 10,000 hexamers, which was used to fit the SAXS data in *A*, is shown in the *gray area*. The *colored lines* show the *R_g_* distribution of the optimized ensemble fitting our Hsp21V181A SAXS data. The selected distributions consist of dimers with between 13% (V181_80) and 8% (V181_84) hexamers. *C*, representative models from the selected pools of the hexamer and dimers, created by EOM. The rigid core is shown in a *surface representation* where the dimers are *colored* in *light blue*, *blue*, and *teal*, and the modeled flexible parts are shown as *spheres*. The *numbers* are as in *A*, including the following number of conformers: 80, four conformers; 81, four conformers; 82, four conformers; 83, eight conformers; 80–84, five hexameric conformers (due to space limitations, we left out the dimer representations from retention time 84 min).

We also used SAXS to further investigate the distribution of Hsp21^V181A^ among different oligomers. The Hsp21^V181A^ variant appears to be non-dodecameric (Fig. 9), in agreement with the results from cross-linking (Fig. 7*B*), and showed concentration-dependent oligomerization in solution (Table 2). Whereas the *R_g_* and *D*_max_ remained around the same values of 4.1–4.2 and 17–17.5 nm, respectively, the molecular mass increased with concentration from 73 to 90 kDa, as calculated from the *V_p_* (Table 2).

To reduce heterogeneity in particle size, the sample was applied to online size-exclusion chromatography SAXS (SEC-SAXS). The separated fractions (fractions 80–84) showed masses ranging from 64 to 71 kDa (Table 2). To analyze the SEC-SAXS data for Hsp21^V181A^, we used EOM and the same pool of models as for Hsp21 wild type. The best fit (χ^2^ between 0.57 and 0.60) was attained using a pool of 10,000 dimers and 10,000 hexamers, resulting in the *R_g_* distribution of the optimized ensemble shown in Fig. 9*B*. This lack of dodecamers confirms the impression that Hsp21^V181A^ is non-dodecameric. The optimized ensemble calculated for the five SAXS data sets taken at five different retention times contains between 87 and 82% dimers, and 13 to 8% hexamers, going from 80 to 84 min of retention time. This equilibrium between Hsp21^V181A^ hexamers and Hsp21^V181A^ dimers is what is observed in the SAXS cell, and we caution that some dissociation of hexamers into dimers may have occurred following elution from the size-exclusion column. In addition, loss of separation quality might occur due to the 2-m-long tubing (inner diameter 0.5 mm, volume ∼400 μl) needed to connect the FPLC to the SAXS cell at the beamline. The *R_g_* and *D*_max_ for these data sets (80–84-min retention time) were between 3.44 and 3.21 nm and between 11.3 and 14 nm, respectively. We conclude that Hsp21^V181A^ is composed of a mixture of mainly dimers and some hexamers.

This loss of the dodecameric conformation in Hsp21^V181A^ corroborates the structural model of Hsp21 because it is in agreement with a decreased affinity in the tail-to-tail interactions between different discs in Hsp21 (Fig. 4). The presence of dimers suggests that also the tail-to-groove interactions between neighboring dimer subunits, as in the Hsp16.9 crystal structure (PDB code 1GME) (25), are weakened in Hsp21^V181A^.

To summarize, our results suggest that the substrate-binding NTR arms are highly flexible and accessible on the outside of the Hsp21 dodecamer, whereas the CTR tails in Hsp21 play a critical role in maintaining the dodecamer topology, such that a single point mutation in the extended I*X*V*X*I motif results in decreased oligomerization coinciding with decreased chaperone activity.

## Discussion

### Hsp21 structural model with rotated discs and pairwise CTR tail interactions

In this study, we describe Hsp21, a chloroplast-localized sHsp, which has a long NTR (82 amino acids) with conserved methionine residues that are important for substrate binding and scavenging of reactive oxygen species (35, 44). We present a structural model of the Hsp21 dodecamer based on homology modeling, cryo-EM, and SAXS, which is validated by independent data from limited proteolysis, cross-linking mass spectrometry, and NMR spectroscopy. Compared with the crystal structure of Hsp16.9 (PDB code 1GME) (25) that was used as a modeling template, the cross-links detected at the dimer interface (Fig. 5*B*) appear to confirm a dimerization mode with reciprocal swapping of the β6-strands into the β-sandwich of the neighboring subunit. Several of the corresponding amino acids in the structure of αB-crystallin (PDB code 3J07) are at Cα–Cα distances exceeding 30 Å. Thus the cross-links we detected in Hsp21 are incompatible with the metazoan type of dimer interface. A difference between the structural model of Hsp21 and the Hsp16.9 modeling template, however, is that the Hsp21 dodecamer shows a rotation of 30° between the two discs, which are also further apart from each other (Fig. 3). The CTR tail-to-groove interactions occur only within the same disc, and the interaction between the discs is instead modeled as a direct CTR tail-to-tail interaction through an extended I*X*V*X*I motif (Figs. 1 and 4). The difference between the Hsp16.9 crystal structure and the Hsp21 model with the two discs rotated and further apart could be due to the longer NTR in Hsp21 compared with that in Hsp16.9 or possibly to a difference between the structure in solution and a certain structural conformation amenable for crystallization.

### Flexible NTR arms on the outside of the Hsp21 dodecamer

The NTR arms of the Hsp21 dodecamer appear flexible, dynamic, and available for substrate binding according to the representative models of the optimized SAXS ensemble (Fig. 8), similar to what was previously suggested for dimers of Hsp16.9 in a simulation study (48). The SAXS data on Hsp21 are also supported by limited proteolysis and NMR (Fig. 6) and by positive density in the cryo-EM maps (Fig. 4), suggesting six arms in the dodecamer interior and six arms available on the outside for immediate substrate binding without dissociation of the dodecamer. This certainly does not exclude the possibility that dodecamers also can dissociate into subunits that interact with substrates. The possibility that flexible NTR arms may be available for substrate interaction on the outer surface of intact oligomers, and not only on a smaller population of transient dimers present during dynamic subunit exchange, was also recently suggested in a revised model of sHsp activity (49, 50). It remains to be shown under what conditions the substrate proteins interact directly with the dodecamers and under what conditions they interact with dissociated subunits.

### CTR tails in Hsp21 crucial for oligomerization and chaperone activity

As a tool to investigate the importance of the oligomerization for chaperone activity, we also evaluated the structure-function relationship of the non-dodecameric mutational variant of Hsp21, Hsp21^V181A^, which appears to be a mixture of mainly dimers and some hexamers (Fig. 9). That the mutant is a mixture of a dimer and hexamer populations in equilibrium, with distribution between them depending on the concentration (Table 2), is partly why it is not that straightforward to determine the oligomeric state of the mutant by SEC and non-denaturing PAGE. The difficulty is also partly due to the fact that the mutant protein appears to be quite sticky and prone to interfere with the column material. We had to add 0.1% Tween 20 for online SEC-SAXS of the mutant, and the size determination by SEC is further complicated by the non-globular shape suggested by the SAXS data. Nevertheless, this V181A point mutation should only affect the flexible CTR tails of the protein, not the folded parts in the protein core. Thus, it appears reasonable that the Hsp21^V181A^ substitution does not perturb the subunit structure, whereas single point mutations in folded regions commonly have this effect (51). In line with this expectation, cross-linking of unlabeled Hsp21^V181A^ and ^15^N-labeled Hsp21 resulted in qualitatively similar hybrid cross-links observed for wild-type Hsp21 (Table 1 and Fig. 5), including the Lys^126^–Lys^161^ cross-link, which most likely occurs between subunits within a dimer. This indicates that Hsp21^V181A^ is structurally unperturbed and forms subunits able to intermix with Hsp21. It is therefore of interest that the chaperone activity of Hsp21^V181A^ is very low (Fig. 7, *E* and *F*) compared with Hsp21 (Fig. 7, *D* and *E*), despite the absence of a structural perturbation of the dimeric building blocks. How can such a drastic reduction in chaperone activity be explained? The Val to Ala substitution should not be expected to cause any large effect on the capacity to interact with hydrophobic surfaces in substrates, particularly because the CTR tails provide a very small surface area for potential substrate binding compared with the NTR arms (300 Å^2^ compared with 800–1,700 Å^2^ as estimated for Hsp16.9 (48). We speculate that the dodecameric configuration may be required for the capability of sHsps to form weak and transient interactions with substrate. The equilibrium between substrate-bound and substrate-free dimers may be pushed toward substrate release if coupled to the equilibrium between substrate-free dimers and dodecamers, which in wild-type Hsp21 is heavily skewed toward dodecamers. The dodecamer is dependent on the CTR tails, and therefore even without a direct role in substrate binding, the CTR tails may indirectly affect the chaperone activity, whereas the NTR arms may affect the chaperone activity more directly through substrate binding.

The NTR arms of Hsp21 show a dual character, with properties of both intrinsically disordered protein and amphipathic helix, with the possibility to switch between the two. Thus, Hsp21 is probably an example of a conditionally disordered chaperone (52) that may switch between self-recognition and substrate binding, as suggested for sHsps that are regulated thermally (53) or by oxidation (54). Recently, Bardwell and co-workers (55, 56) used a well-characterized and elegant experimental system and found that chaperone interactions are both electrostatic and hydrophobic. They also provided strong evidence for a rapid binding of unfolding substrates, with a modest (micromolar) affinity, that can compete with and prevent aggregation. Subsequently, while still bound to the chaperone, a slower substrate refolding can occur, which in itself triggers substrate release. Intriguingly, this may be a widespread mechanism of function for ATP-independent chaperones, including sHsps. The coupled equilibria between dodecamers and dimers, which we proposed above to possibly be important for the chaperone activity in Hsp21, could further facilitate such substrate release.

To summarize, we have obtained a structural model of Hsp21 that fits both SAXS data and cryo-EM data, suggesting a division of labor in the chaperone activity of Hsp21 such that the CTR tails maintain the oligomeric structure necessary for the chaperone activity, and the NTR arms take part in the interactions with the substrate proteins.

## Experimental procedures

### Proteins, recombinant expression, and purification of Hsp21 and Hsp21^V181A^

For recombinant expression of *A. thaliana* Hsp21 (UniProt code P31170) and the mutational variant Hsp21^V181A^, a plasmid with codon-optimized sequence was subcloned into pJC20 vector without the presequence (a service purchased from GenScript, Piscataway, NJ) and transformed into *Escherichia coli* strain ER2566 and expressed using overnight autoinduction. A 1-liter N-Z amine/yeast (ZY) growth medium containing 50 mg/ml ampicillin, 1 mm MgSO_4_, 5.48 mm glycerol, 0.28 mm glucose, 0.58 mm α-lactose, 2.50 mm (NH_4_)_2_SO_4_, 0.59 mm KH_2_PO_4_, and 5.00 mm Na_2_HPO_4_ was inoculated with 1 ml of LB broth containing 50 mg/ml ampicillin grown for 10 h after inoculation from one colony. This was incubated at 37 °C and 120 RPM for 15 h. Agarose-gel electrophoresis was run in 1% agarose at pH 8.6 (57) to monitor the content of overexpressed protein in the *E. coli* cultures by Coomassie Brilliant Blue staining. Aliquots of 1 ml were withdrawn and centrifuged to separate supernatants (medium) from pellets (cells), which were dissolved in 0.5 ml of 8 m urea. Samples were loaded into punched-out wells in the agarose, and negatively charged proteins migrated under non-denaturing conditions toward the anode. The cells were harvested with centrifugation at 6,000 × *g* for 10 min and frozen at −20 °C. Cell pellets were dissolved in buffer (50 mm HEPES, pH 7.5, 150 mm NaCl, 10 mm DTT, and 1 mm EDTA), with DNase (Sigma-Aldrich, Stockholm, Sweden) and protease inhibitor (cOmplete EDTA-free; Roche Diagnostics Scandinavia AB, Stockholm, Sweden) added just before cell lysis, which was performed by passing through a French press, followed by a 1-h centrifugation at 20,000 × *g*. The pellet was discarded, and the proteins were precipitated from the supernatant using (NH_4_)_2_SO_4_. First the contaminating proteins were precipitated (1-h centrifugation at 17,000 × *g* at 55% saturation) and discarded, and then Hsp21 was precipitated at 95% (NH_4_)_2_SO_4_ saturation. After 1 h of centrifugation at 17,000 × *g*, the pellet containing precipitated Hsp21 was dissolved in 50 mm HEPES, 150 mm NaCl, 10 mm DTT, 1 mm EDTA, pH 7.5. Before the next step, the Hsp21 solution was centrifuged (10 min, 10,000 × *g*) to remove potential aggregates. The protein was further purified by size-exclusion chromatography, using a HiLoad/Superdex 200 HR 16/60a column from GE Healthcare LifeSciences (Uppsala, Sweden) equilibrated with elution buffer (50 mm HEPES, pH 7.5, 150 mm NaCl, 1 mm EDTA, 10 mm DTT). Proteins were eluted at a flow rate of 1.5 ml/min. Hsp21 should elute at a position corresponding to its theoretically calculated mass, 240 kDa. Contaminating proteins were mainly of smaller sizes and were eluted after the Hsp21 protein. Fractions containing Hsp21 were pooled, concentrated, and incubated with 4 m urea for 20 min on ice and then loaded on a HiLoad/Superdex 200 HR 16/60a column again, and Hsp21 eluted as monomeric subunits at a position corresponding to its theoretically calculated mass of 21 kDa. The fractions containing Hsp21 were collected and dialyzed for 15–20 h with urea-free buffer (50 mm HEPES, 100 mm NaCl, 10 mm DTT, 5 mm MgCl_2_, pH 8) and then concentrated. After analysis on a silver-stained SDS-polyacrylamide gel, it was noticed that the second column run with urea-treated sample was not required to increase the purity of the sample. The urea-treated samples were pooled with the samples without urea treatment. This was all dialyzed with urea-free buffer (50 mm HEPES, 100 mm NaCl, 10 mm DTT, 5 mm MgCl_2_, pH 8) and then concentrated.

### Recombinant expression and purification of Hsp21^V181A^

Plasmid with a V181A point mutation was transformed into *E. coli* BL21 DE3 star PLysS and expressed in the same way as Hsp21. The Hsp21^V181A^ protein was found to leak into the cell growth medium, where it was found as a single protein in large amounts and relatively pure. The cells were harvested with centrifugation at 6,000 × *g* for 10 min. DTT was added to the cell growth media to a final concentration of 20 mm. The purification protocol for dodecameric Hsp21, based on early elution in SEC, was not amenable to HSP21^V181A^, and the following protocol for purification was developed, which is based on the observation that HSP21^V181A^ was expelled into the growth medium in relatively pure form, from which it could be purified by ion-exchange chromatography. The growth medium was collected and, after adjustment of pH to 8.0, diluted 10 times with Millipore water to lower the ionic strength. Solid DE23 anion exchanger (DEAE-cellulose) from GE Healthcare was added, 140 g/liter. The slurry was incubated at 45 °C for 5 min under constant stirring and then filtrated through filter paper from Munktell (Grycksbo, Sweden). After washing with two column volumes of buffer (10 mm Tris, pH 8.0, and 10 mm DTT), elution of bound proteins was obtained by the stepwise addition of buffer with NaCl in different concentrations, 0.1, 0.15, and 0.5 m. The elution with 0.1 and 0.15 mm NaCl contained pure Hsp21^V181A^ and was pooled and concentrated using Amicon Ultra centrifugal filter unit Ultra-15, molecular weight cut-off 10 kDa from Sigma-Aldrich.

### Other proteins and reagents

MDH and CS from *Sus scrofa* (pig) (Uniprot code P00346 and P00889, respectively) were purchased from Sigma-Aldrich. The cross-linking reagent bis(sulfosuccinimidyl)suberate (BS3), was obtained from Thermo Fisher Scientific (Rockford, IL). Denaturing and non-denaturing electrophoresis was done according to Ref. 45.

### Cryo-electron microscopy sample preparation and imaging

For cryo-EM, 3 μl of Hsp21 solution (5 μg/ml) was applied onto 400-mesh glow-discharged Quantifoil R2/4 grids (Quantifoil Micro Tools GmbH, Grosslöbichau, Germany) coated with a thin layer of continuous carbon. After addition of Hsp21, the grids were incubated for 30 s, blotted for 3 s, and then vitrified in liquid ethane using an FEI Vitrobot plunge freezer (temperature = 18 °C, humidity = 100%). Frozen grids were stored in liquid nitrogen until further use. The grids were loaded into a Gatan 626 cryo-holder and transferred to a JEOL JEM2100F electron microscope that was operating at 200 kV. The grids were maintained at approximately −180 °C during the entire data collection processes. Images were recorded at 2–5-μm defocus on a DE-20 direct electron detector (Direct Electron) at a magnification of ×50,000, resulting in a sampling distance of 1.24 Å/pixel. Each image was exposed for 2 s using a frame rate of 20 frames/s (dose rate ∼1.4 e^−^/Å^2^/frame), giving an accumulated dose of ∼60 e^−^/Å^2^. The data set consisted of a total of 152 images.

### Image processing

The effect of sample drift was compensated for each frame using the DE_process_frames-2.7.1.py script (58). The initial five frames were discarded due to the large amount of drift. For the remaining frames (frames 6–40), full frame alignment was performed. The alignment procedure was iterated four times, and the final drift-corrected images were imported for initial evaluation using EMAN2 (59). 12 images were discarded due to contamination, low contrast, or high defocus, reducing the data set to 140 images. References for template-based particle picking were selected manually using XMIPP (60), producing 1,008 particles from a subset of 21 images. The particle coordinate files were imported to RELION and used for reference-free 2D classification (61). The predominant class average images (*n* = 8) were used as templates for automatically selecting 33,456 particles from 140 images in RELION (62). Particles in ice-contaminated areas were manually deselected. The coordinates of the automatically picked particles were imported to EMAN2 (59) for further processing. Reference-free 2D class averaging was performed to discard false positives in the data set of the automatically picked particles and to select references for generating an initial model. The 2D classification was iterated another time to discard more false positives, resulting in a set of 18,407 particles selected for 3D refinement. The 3D refinement was performed using D3 symmetry. An initial round of 3D refinement was performed using a downscaled (2.48 Å/pixel) data set, aiming at low resolution. The final map of the first 3D refinement was used as a starting model for a second 3D refinement, using the full-sized (1.24 Å/pixel) data set as input, aiming at medium resolution. Finally, a third 3D refinement, using the final map of the second refinement as a starting model and the full-sized data set, aiming at high resolution was generated. The resolution reported for the final map of the third 3D refinement, generated using the gold-standard Fourier shell correlation (FSC) procedure (63), was calculated at FSC = 0.143 (64).

### Homology modeling of Hsp21

To generate a homology model of Hsp21, the crystal structure of Hsp16.9 (PDB code 1GME) (25) was used as template. The Hsp21 sequence was extracted from P31170 by removing the chloroplast transit peptide (37). Alignment of the Hsp21 and Hsp16.9 (Q41560) sequences was performed with Clustal Omega (65). Separate Hsp21 homology models, with chain A and B in 1GME as templates, were generated using the software MODELLER (66). The extended sequence in Hsp21, representing the prolonged NTR arm and the unresolved region in Hsp16.9 chain B, were deleted from the produced Hsp21 models. The dimeric arrangement of chain A and B in Hsp16.9 was then used as guidance for constructing the Hsp21 dimer. Model building of the Hsp21 dimer was performed using the Match-Maker extension in Chimera (67). Two hexameric discs, retrieved from the crystal structure of Hsp16.9, acted as template for producing two Hsp21 hexamers. The Hsp21 hexameric discs were subsequently docked into the cryo-EM density map. First, rough manual fitting was performed, followed by utilizing the Fit in Map command in Chimera (68).

### Difference map calculation

Structure factors were generated by sfall in the ccp4 package (69, 70) from the coordinates of Hsp16.9 (PDB code 1GME) (25) following fitting to the Hsp21 cryo-EM map described above. Maps covering the 1GME dodecameric complex were calculated at 5, 10, and 15 Å resolution, respectively. The 10 Å 1GME map was resampled to the same sampling interval as the Hsp21 map, and both maps were scaled to have 0 mean value and S.D. of 1.0. A difference map was determined using the command “vop,” which edits volume data, to create new volume data in Chimera (68), subtracting the 1GME map from the Hsp21 map. Thus, positive contours should correspond to residual density arising from the additional sequence motif in Hsp21.

### Chemical cross-linking and proteolysis

The protein concentration was 50 μm or as otherwise stated. The cross-linker BS3 was dissolved in distilled water to a concentration of 30 mm immediately before use. Samples were incubated with BS3 in a 10- or 60-fold excess to protein at room temperature. After 20 min, the cross-linking reaction was quenched by adding 1 m Tris to a final concentration of 20 mm. The samples were subjected to total proteolysis, by either in-solution digestion or in-gel digestion. For in-solution digestion, the samples were incubated for 10 min at room temperature in 3.3 m urea for protein denaturation and then diluted with 50 mm NH_4_HCO_3_ to a urea concentration < 1.5 m before trypsin was added to a 1:100 molar ratio (trypsin/protein), after which the solution was incubated for 30 min at 37 °C, more trypsin was added to molar ratio a 1:50, and the solution was incubated at 37 °C overnight. The tryptic digestion was stopped by adding formic acid for pH <2. For in-gel digestion, the Shevchenko protocol was used (71). Before LC-MS/MS, all samples were pretreated on Poros R2 reversed phase microcolumns as described previously (72), except that formic acid was used instead of trifluoroacetic acid. Mixed isotope labeling was performed as described previously (42) to distinguish between intrasubunit cross-links and intersubunit cross-links that may be detected as hybrid ^14^N-^15^N cross-links. Non-labeled Hsp21 was incubated with ^15^N-labeled Hsp21 at a 1:1 ratio (Hx1), and non-labeled Hsp21^V181A^ was incubated with ^15^N-labeled Hsp21 at 1:12, 2:12, 6:12, and 1:1 (Hx2–Hx5). This was done at 37 °C for 1 h. The samples were then cross-linked and trypsin-digested (either in gel or in solution) as described above. For limited proteolysis, samples were digested without urea in 0.1 m NH_4_HCO_3_ for 1–30 min at a 1:2,000 molar ratio (trypsin/protein) and stopped by the addition of 10% formic acid to lower pH to 2. Samples were analyzed by MALDI mass spectrometry as described previously (42). For quantitative evaluation of peptides formed during limited proteolysis, samples were diluted with ^15^N-labeled reference before dried droplet sample deposition on a target plate, by premixing samples with aliquots of a sample of ^15^N-labeled Hsp21 that had been subjected to total proteolysis.

### Mass spectrometric data acquisition

Peptides were subjected to reversed phase nano-LC before mass spectrometric analyses in an LTQ-Orbitrap Velos Pro mass spectrometer (Thermo Fischer Scientific) equipped with a nanoEasy spray ion source (Proxeon Biosystems, Odense, Denmark). The chromatographic separation was performed at 40 °C on a 15-cm (75-μm inner diameter) EASY-spray column packed with 3-μm resin (Proxeon Biosystems, Odense, Denmark). The nano-HPLC intelligent flow control gradient was 5–20% solvent B (0.1% (v/v) formic acid, 100% (v/v) acetonitrile in water) in solvent A (0.1% (v/v) formic acid in water) during 60 min and then 20–40% during 30 min, followed with an increase to 90% during 5 min. A flow rate of 300 nl/min was used through the whole gradient. An MS scan (usually 350–2,000 *m*/*z*) was recorded in the Orbitrap mass analyzer set at a resolution of 60,000 at 400 *m*/*z*. The MS is followed by data-dependent collision-induced dissociation MS/MS scans on the 10 most intense multiply charged ions in the LTQ, excluding +1 and +2.

### Mass spectrometric data analysis

Raw data were converted to mgf format by Mascot Distiller (version 2.3) and further filtered to retain only the top 125 most intense peaks per scan with the software MassAI (version August 2015 (72, 73)). Sequence coverage was 100%. The identification of cross-links was made with the following search settings: fragmentation mode: collision-induced dissociation, 10 ppm MS accuracy, 0.05 Da MS/MS accuracy, trypsin (pig) as enzyme, 2 allowed missed cleavages; variable modifications: M (M-ox), K (BS3-internal x-link (x-linker)), K (BS3-H20 (x-linker)), * (BS3-H20 (N-term)), cross-linkers BS3-d0. The searches were performed with the setting “Also xlink modified peptides” with the filtering option “Crosslink peptide only if peptide is observed as deadend,” which reduces the number of false positives. The MS/MS spectra of proposed cross-links were then manually validated and accepted only if four criteria were fulfilled: score > 25, intensity > 1,000, at least 30% of fragment ions from both peptides, no major peaks unexplained. Thus, there are no false positives in the table with detected cross-links and no false discovery rate to be calculated. The approved MS/MS spectra are provided in the supplemental information. After carrying out high-quality molecular dynamics simulations of 807 proteins representative of diverse protein folds to investigate the relationship between lysine-lysine distances in experimental starting structures and in simulation ensembles (43), we concluded that for DSS/BS3, a distance constraint of 26–30 Å between Cα atoms is appropriate.

### Chaperone activity measurements

The aggregation of the thermosensitive model substrates MDH and CS and how added Hsp21 prevents aggregation were evaluated by light-scattering changes at 360 nm, as described previously (45), and using GraphPad Prism version 7, data were normalized to the spontaneous aggregation of each model substrate.

### NMR spectroscopy and data analysis

All experiments were performed at 20 °C on Agilent VNMRS spectrometers operating at magnetic field strengths of 11.7 and 14.1 T. ^1^H-^15^N NOESY-HSQC and ^1^H-^15^N TOCSY-HSQC 3D experiments were recorded at 11.7 T with spectral widths of 8,012.821 Hz sampled by 1,024 complex points in ω_1_ (^1^H), 8,012.821 Hz sampled by 64 complex points in ω_2_ (^1^H), and 1,000 Hz sampled by 32 complex points in ω_3_ (^15^N). The carrier was placed at 4.82 ppm for ω_1_ and ω_2_ and 116.91 ppm for ω_3_.

^1^H-^15^N HSQC experiments were recorded at 14.1 T with a spectral width of 10,000 Hz sampled by 1,024 complex points in ω_2_ (^1^H) and 2,000 Hz sampled by 256 complex points in ω_1_ (^15^N). The carriers were placed at 4.82 and 119.30 ppm.

All spectra were processed using NMRPipe (74). Resonance assignments were carried out using CcpNmr analysis (75). Peak volumes were integrated using PINT (76).

### SAXS data collection and analysis

The SAXS data were collected at the MAX IV Laboratory Max II beamline I911-SAXS (77). The data were initially reduced and processed using an automatic pipeline of scripts developed at MAX-Lab and using the ATSAS tools (78, 79). Data were normalized to the intensity of the transmitted beam, and buffer scattering was subtracted. Further processing was done using the ATSAS software package (78, 79). With Primus, in ATSAS, forward scattering *I*(0) and the *R_g_* were estimated using the Guinier approximation. Similarly, in Primus, we could calculate the distance distribution functions *P*(*R*) of the scattering patterns and estimate the maximum particle dimensions *D*_max_. Primus also automatically calculates the excluded volume of the hydrated particle (Porod volume *V_p_*) (78, 80) together with the distance distribution function. All data sets were modeled with DAMMIF (81), and the *ab initio* model's excluded volume *V_a_* was divided by 2 to estimate the molecular weight (78).

All samples were prepared at three different concentrations between 2.5 and 10 mg/ml to investigate concentration-dependent effects. Before measurement, the samples were centrifuged in a temperature-controlled tabletop centrifuge at 10,000 rpm for 10 min. We also performed on-line size-exclusion chromatography on Hsp21^V181A^, using an ÄKTA Pure (GE Healthcare) FPLC. The sample, 250 μl of Hsp21^V181A^, was applied to a Superdex 200 Increase 10/300 (GE Healthcare) equilibrated with 50 mm HEPES buffer, pH 7.5, and 100 mm NaCl. The flow rate was 0.15 ml/min, and SAXS data were acquired every minute. To decrease sample interaction with the column, 0.1% Tween 20 was added to the mobile phase.

### Modeling of Hsp21 NTR arms

We used EOM (46, 47) to complement the structural model of Hsp21 with the flexible NTR arms of the Hsp21 hexamer and dodecamer. The sequence of the NTR is ∼40 amino acids shorter in Hsp16.9 used as template in generating the structural model of Hsp21; furthermore, only 6 of 12 NTR arms are resolved in the crystal structure (PDB code 1GME) (25). The structural model of Hsp21 was used after removing all amino acids preceding the folded domain (*i.e.* positions 1–82) as a rigid body in EOM simulation to visualize the 12 NTR arms, corresponding to amino acids 1–82 in Hsp21. Using the feature RanCh in the EOM program suite, we calculated 10,000 dimers using two rigid bodies of residues 83–171. We calculated 10,000 hexamers and 5,000 dodecamers using 6 and 12 rigid bodies, respectively, of residues 83–184. The feature GAJOE in the EOM program suite was finally used with default parameters to find an optimized ensemble best fitting the given SAXS data. GAJOE employs a genetic algorithm to select subsets of protein models to minimize the discrepancy between the experimental data and the average theoretical scattering of the subset. The experimental SAXS data were analyzed using all possible combinations of pools: dimer, hexamer, dodecamer, dimer-hexamer, dimer-dodecamer, hexamer-dodecamer, and dimer-hexamer-dodecamer. Furthermore, to test the hypothesis of a compact Hsp21 dodecamer, we identified a subunit conformation from the best EOM modeling attempt where the N-terminal was inside the dodecamer. This conformation was then simply superimposed on the other 11 subunits, and the resulting dodecamer was saved with all N-terminal arms inside. CRYSOL (82) was subsequently used to estimate the fit of this compact dodecamer to the experimental data.

## Author contributions

G. R. and C. E. designed the study with H. H. and C. A. G. S. G. R. and Y. W. purified proteins, G. R. performed mass spectrometry, and G. R. and C. E. performed data analysis. J. H., P. J. B. K., and H. H. performed homology modeling, cryo-EM, and 3D construction. M. I. R. and P. H. developed and adapted software for cross-link data analysis. S. W., M. R., and M. A. performed NMR and data analysis. G. R. and C. A. G. S. collected SAXS data, and C. A. G. S. performed data analysis and EOM simulations. G. R., J. H., M. A., and C. E. wrote the manuscript. All authors reviewed the results and approved the final version of the manuscript.

## Supplementary Material

Supplemental Data

## Footnotes

4 The abbreviations used are:

sHsp: small heat-shock protein
ACD: α-crystallin domain in sHsps
CTR: C-terminal region in sHsps
NTR: N-terminal region in sHsps
Hsp21: the 21-kDa chloroplast-localized small heat-shock protein
SAXS: small-angle X-ray scattering
PDB: Protein Data Bank
HSQC: heteronuclear single quantum coherence
EOM: ensemble optimization method
SEC: size-exclusion chromatography
BS3: bis(sulfosuccinimidyl) suberate
FSC: Fourier shell correlation
MDH: malate dehydrogenase
CS: citrate synthase.
