# Supplementary material for: Structural model of dodecameric heat-shock protein Hsp21: Flexible N-terminal arms interact with client proteins while C-terminal tails maintain the dodecamer and chaperone activity
Source: J Biol Chem. 2017 Mar 21;292(19):8103–21. doi: 10.1074/jbc.M116.766816 (PMC5427286; doi:10.1074/jbc.M116.766816)
Supplement: Supplemental Data [file supp_292_19_8103__index.html]

Structural model of dodecameric heat-shock protein Hsp21: Flexible N-terminal arms interact with client proteins while C-terminal tails maintain the dodecamer and chaperone activity — Hsp21 structural model — Supplemental Data 

# Structural model of dodecameric heat-shock protein Hsp21: Flexible N-terminal arms interact with client proteins while C-terminal tails maintain the dodecamer and chaperone activity

## Supplemental Data

- Supplemental Material
- Supplemental information 1\_hsp21\_map
- Supplemental information 2\_hsp21\_map\_start\_model
- Supplemental information 3\_hsp21\_model\_170406.pdb
- Supplemental information 4\_validation\_report\_hsp21\_model\_170406
- Supplemental information 5\_MS\_and\_MSMS-spectra
- Supplemental movie 1\_hsp21\_screw
- Supplemental movie 2\_hsp21\_nterm
