# Supplementary material for: Structural model of dodecameric heat-shock protein Hsp21: Flexible N-terminal arms interact with client proteins while C-terminal tails maintain the dodecamer and chaperone activity
Source: J Biol Chem. 2017 Mar 21;292(19):8103–21. doi: 10.1074/jbc.M116.766816 (PMC5427286; doi:10.1074/jbc.M116.766816)
Supplement: Supplemental Data [file supp_M116.766816_Supplemental_information_content_list_corr.docx]

**Supplemental information - content list**

**Supplemental information 1 Cryo-EM density map for Hsp21 - refined model**

After final refinement this reconstructed density map of Hsp21 at 10 Å resolution was obtained showing an Hsp21 oligomer approximately 10 nm wide (3-fold axis view) and 9 nm high (2-fold axis view) as presented in Fig. 2. This .mrc file is deposited in the EM data bank as EMDB #3459.

*File name: Supplemental information 1_hsp21_map.mrc*

**Supplemental information 2 Cryo-EM density map for Hsp21 - start model**

This .mrc file is the first starting model generated and refined without symmetry (C1). Further processing and refinement of the reconstruction was performed in D3-symmetry, resulting in the density map presented in Fig. 2 and the .mrc file in Supplemental information 1.

*File name: Supplemental information 2_ hsp21_map_start_model.*mrc

**Supplemental information 3 Structural model of Hsp21**

The Hsp21 structural model is based on the crystal structure of the dodecameric Hsp16.9 (PDB # 1GME) as a template for homology modelling and fitting into the cryo-EM density map presented in Fig. 2. After a relative rotation of the two discs by about 30° and expanding the distance between the discs by about 35 Å, as illustrated in the Supplemental movie 1, the structural model of Hsp21 presented in Fig. 3 was obtained and the .pdb file deposited in the Protein Data bank as PDB # 5MB8.

*File name: Supplemental information 3_hsp21_model.pdb*

**Supplemental information 4 Validation report for structural model of Hsp21**

The wwPDB/EMDataBank EM Map/Model Validation Summary Report, entitled ‘Hsp21 dodecamer, structural model based on cryo-EM and homology modelling’, was produced by the wwPDB biocuration pipeline after annotation of the structure. The structural model of Hsp21 is presented in Fig. 3. The .pdb file of the Hsp21 model is provided as Supplemental information 3, and deposited in Protein data Bank as PDB # 5MB8.

*File name: Supplemental information 4_validation report_hsp21_model.pdf*

**Supplemental information 5 MS and MSMS spectra for crosslinked Hsp21 peptides**

This file contains mass spectra (MSMS, or MSMS and ^14^N-^15^N MS) in support for the identified crosslinked peptides in Table 1.

*File name: Supplemental information 5_MS and MSMS-spectra.pdf*

**Supplemental movie 1 Rotation and expanded distance changes in Hsp21 structural model**

The rotation and expanded distance changes required to fit the Hsp21 structural model presented in Fig. 3 to the density map presented in Fig. 2 here described as movement along an imaginary screw.

*File name: Supplemental movie 1_hsp21_screw.mov*

**Supplemental movie 2 Dynamic movement of the N-terminal arms of Hsp21 in EOM simulations**

The SAXS data presented in Fig. 8 were interpreted using the Enzemble Optimization Method (EOM). A collection of images from the EOM simulations indicate a dynamic movement of the N-terminal arms that extend away from the core dodecamer of Hsp21.

*File name: Supplemental movie 2_hsp21_nterm.mov*
