# Supplementary material for: Structural model of dodecameric heat-shock protein Hsp21: Flexible N-terminal arms interact with client proteins while C-terminal tails maintain the dodecamer and chaperone activity
Source: J Biol Chem. 2017 Mar 21;292(19):8103–21. doi: 10.1074/jbc.M116.766816 (PMC5427286; doi:10.1074/jbc.M116.766816)
Supplement: Supplemental Data [file supp_M116.766816_Supplemental_information_4_validation_report_hsp21_model_170406.pdf]

Full wwPDB/EMDataBank EM Map/Model Validation Report (i)

Apr 6, 2017 – 04:01 PM BST

Deposition ID : D_1200004358
PDB ID : *(not yet assigned)*

This is a Full wwPDB/EMDataBank EM Map/Model Validation Report.

This report is produced by the wwPDB Deposition System during initial deposition but before annotation of the structure.

We welcome your comments at *validation@mail.wwpdb.org*
A user guide is available at
http://wwpdb.org/validation/2016/EMValidationReportHelp
with specific help available everywhere you see the (i) symbol.

MolProbity	:	4.02b-467
Percentile statistics	:	20161228.v01 (using entries in the PDB archive December 28th 2016)
Ideal geometry (proteins)	:	Engh & Huber (2001)
Ideal geometry (DNA, RNA)	:	Parkinson et. al. (1996)
Validation Pipeline (wwPDB-VP)	:	rb-20029077

PRELIMINARY VALIDATION REPORT

1 Overall quality at a glance (i)

The following experimental techniques were used to determine the structure:
ELECTRON MICROSCOPY

The reported resolution of this entry is unknown.

Percentile scores (ranging between 0-100) for global validation metrics of the entry are shown in the following graphic. The table shows the number of entries on which the scores are based.

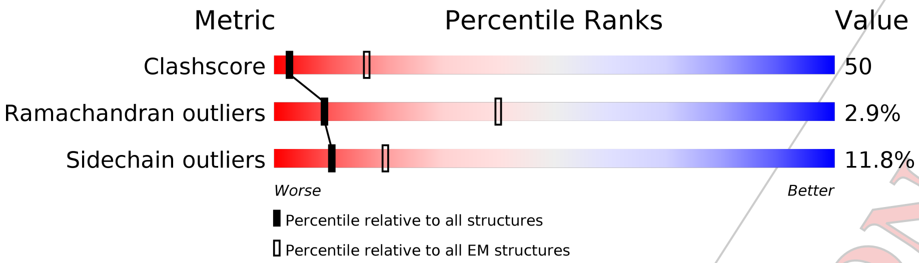

Metric	Whole archive (#Entries)	EM structures (#Entries)
Clashscore	125131	1336
Ramachandran outliers	121729	1120
Sidechain outliers	121581	1026

The table below summarises the geometric issues observed across the polymeric chains. The red, orange, yellow and green segments on the bar indicate the fraction of residues that contain outliers for >=3, 2, 1 and 0 types of geometric quality criteria. A grey segment represents the fraction of residues that are not modelled. The numeric value for each fraction is indicated below the corresponding segment, with a dot representing fractions <=5%

Mol	Chain	Length	Quality of chain
1	A	143	44% 36% 15% 5%
1	C	143	45% 36% 15% 5%
1	D	143	43% 36% 15% 5%
1	G	143	43% 36% 15% 5%
1	I	143	45% 35% 15% 6%
1	J	143	44% 36% 15% 5%
2	B	102	48% 31% 9% 12%
2	E	102	49% 32% 8% 11%
2	F	102	48% 31% 9% 12%

Continued on next page...

PRELIMINARY VALIDATION REPORT

Continued from previous page...

Mol	Chain	Length	Quality of chain
2	H	102	50% 30% 9% 11%
2	K	102	49% 32% 9% 10%
2	L	102	48% 30% 11% 11%

PRELIMINARY VALIDATION REPORT

2 Entry composition (i)

There are 2 unique types of molecules in this entry. The entry contains 23808 atoms, of which 12006 are hydrogens and 0 are deuteriums.

In the tables below, the AltConf column contains the number of residues with at least one atom in alternate conformation and the Trace column contains the number of residues modelled with at most 2 atoms.

- Molecule 1 is a protein.

Mol	Chain	Residues	Atoms	AltConf	Trace
1	A	143	Total C H N O S<br>2301 707 1159 200 226 9	0	0
1	C	143	Total C H N O S<br>2301 707 1159 200 226 9	0	0
1	D	143	Total C H N O S<br>2301 707 1159 200 226 9	0	0
1	G	143	Total C H N O S<br>2301 707 1159 200 226 9	0	0
1	I	143	Total C H N O S<br>2301 707 1159 200 226 9	0	0
1	J	143	Total C H N O S<br>2301 707 1159 200 226 9	0	0

- Molecule 2 is a protein.

Mol	Chain	Residues	Atoms	AltConf	Trace
2	B	102	Total C H N O S<br>1667 516 842 142 164 3	0	0
2	E	102	Total C H N O S<br>1667 516 842 142 164 3	0	0
2	F	102	Total C H N O S<br>1667 516 842 142 164 3	0	0
2	H	102	Total C H N O S<br>1667 516 842 142 164 3	0	0
2	K	102	Total C H N O S<br>1667 516 842 142 164 3	0	0
2	L	102	Total C H N O S<br>1667 516 842 142 164 3	0	0

PRELIMINARY VALIDATION REPORT

3 Residue-property plots (i)

These plots are drawn for all protein, RNA and DNA chains in the entry. The first graphic for a chain summarises the proportions of the various outlier classes displayed in the second graphic. The second graphic shows the sequence view annotated by issues in geometry. Residues are color-coded according to the number of geometric quality criteria for which they contain at least one outlier: green = 0, yellow = 1, orange = 2 and red = 3 or more. Stretches of 2 or more consecutive residues without any outlier are shown as a green connector. Residues present in the sample, but not in the model, are shown in grey.

- Molecule 1:

Chain A:

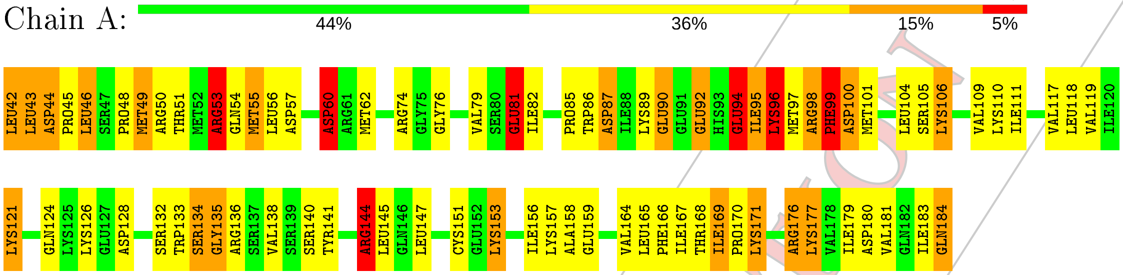

- Molecule 1:

Chain C:

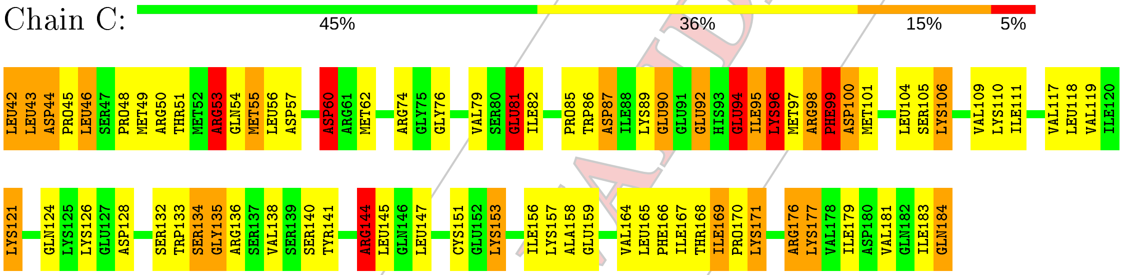

- Molecule 1:

Chain D:

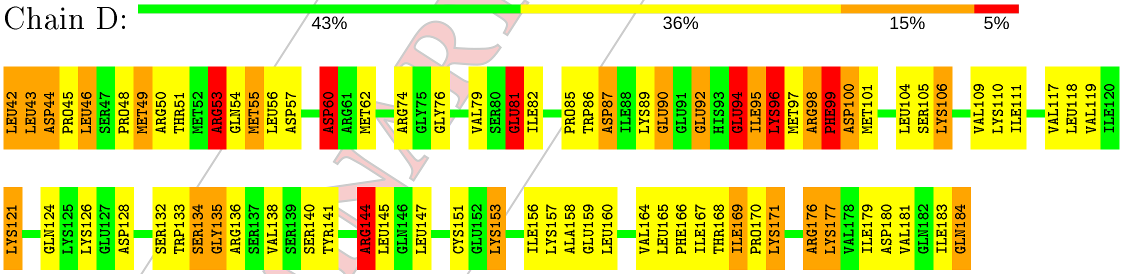

- Molecule 1:

Chain G:

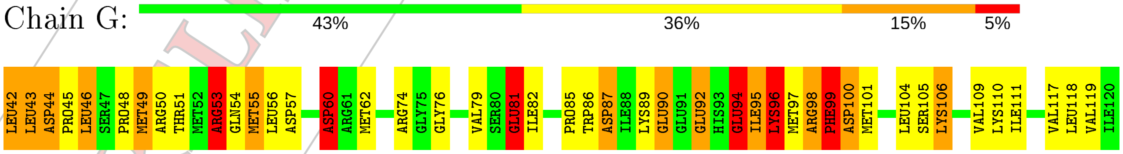

PRELIMINARY VALIDATION REPORT

LYS121, GLN124 LYS125 LYS126 GLU127 ASP128, SER132 TRP133 SER134 GLY135 ARG136 SER137 VAL138 SER139 SER140 TYR141, ARG144 LEU145 GLN146 LEU147, CYS151 GLU152 LYS153, ILE156 LYS157 ALA158 GLU159 LEU160, VAL164 LEU165 PHE166 ILE167 THR168 ILE169 PRO170 LYS171, ARG176 LYS177 VAL178 ILE179 ASP180 VAL181 GLN182 ILE183 GLN184

- Molecule 1:

Chain I: 45% 35% 15% 6%

LEU42 LEU43 ASP44 PRO45 LEU46 SER47 PRO48 MET49 ARG50 THR51 MET52 ARG53 GLN54 MET55 LEU56 ASP57, ASP60 ARG61 MET62, ARG74 GLY75 GLY76, VAL79 SER80 GLU81 ILE82, PRO85 TRP86 ASP87 ILE88 LYS89 GLU90 GLU91 GLU92 HIS93 GLU94 ILE95 LYS96 MET97 ARG98 PHE99 ASP100 MET101, LEU104 SER105 LYS106, VAL109 LYS110 ILE111, VAL117 LEU118 VAL119 ILE120

LYS121, GLN124 LYS125 LYS126 GLU127 ASP128, TRP133 SER134 GLY135 ARG136 SER137 VAL138 SER139 SER140 TYR141, ARG144 LEU145 GLN146 LEU147, CYS151 GLU152 LYS153, ILE156 LYS157 ALA158 GLU159, VAL164 LEU165 PHE166 ILE167 THR168 ILE169 PRO170 LYS171, ARG176 LYS177 VAL178 ILE179 ASP180 VAL181 GLN182 ILE183 GLN184

- Molecule 1:

Chain J: 44% 36% 15% 5%

LEU42 LEU43 ASP44 PRO45 LEU46 SER47 PRO48 MET49 ARG50 THR51 MET52 ARG53 GLN54 MET55 LEU56 ASP57, ASP60 ARG61 MET62, ARG74 GLY75 GLY76, VAL79 SER80 GLU81 ILE82, PRO85 TRP86 ASP87 ILE88 LYS89 GLU90 GLU91 GLU92 HIS93 GLU94 ILE95 LYS96 MET97 ARG98 PHE99 ASP100 MET101, LEU104 SER105 LYS106, VAL109 LYS110 ILE111, VAL117 LEU118 VAL119 ILE120

LYS121, GLN124 LYS125 LYS126 GLU127 ASP128, SER132 TRP133 SER134 GLY135 ARG136 SER137 VAL138 SER139 SER140 TYR141, ARG144 LEU145 GLN146 LEU147, CYS151 GLU152 LYS153, ILE156 LYS157 ALA158 GLU159, VAL164 LEU165 PHE166 ILE167 THR168 ILE169 PRO170 LYS171, ARG176 LYS177 VAL178 ILE179 ASP180 VAL181 GLN182 ILE183 GLN184

- Molecule 2:

Chain B: 48% 31% 9% 12%

ARG83 ALA84 PRO85 TRP86 ASP87 ILE88 LYS89 GLU90 GLU91 GLU92 HIS93 GLU94 ILE95 LYS96 MET97 ARG98 PHE99 ASP100 MET101, LEU104 SER105 LYS106 GLU107, LYS110 ILE111 SER112 VAL113 GLU114 ASP115 ASN116 VAL117 LEU118 VAL119 ILE120, GLN124, ASP130 ASP131 SER132 TRP133 SER134, TYR141 GLY142 THR143 ARG144 LEU145 GLN146 LEU147, LYS153 ASP154 LYS155 ILE156 LYS157 ALA158

LYS161, VAL164 LEU165 PHE166 ILE167 THR168 ILE169 PRO170 LYS171, VAL174 GLU175 ARG176, GLN184

- Molecule 2:

Chain E: 49% 32% 8% 11%

ARG83 ALA84 PRO85 TRP86 ASP87 ILE88 LYS89 GLU90 GLU91 GLU92 HIS93 GLU94 ILE95 LYS96 MET97 ARG98 PHE99 ASP100 MET101, LEU104 SER105 LYS106 GLU107, LYS110 ILE111 SER112 VAL113 GLU114 ASP115 ASN116 VAL117 LEU118 VAL119 ILE120, GLN124, ASP130 ASP131 SER132 TRP133 SER134, TYR141 GLY142 THR143 ARG144 LEU145 GLN146 LEU147, LYS153 ASP154 LYS155 ILE156 LYS157 ALA158

LYS161, VAL164 LEU165 PHE166 ILE167 THR168 ILE169 PRO170 LYS171, VAL174 GLU175 ARG176, GLN184

- Molecule 2:

Chain F: 48% 31% 9% 12%

PRELIMINARY VALIDATION REPORT

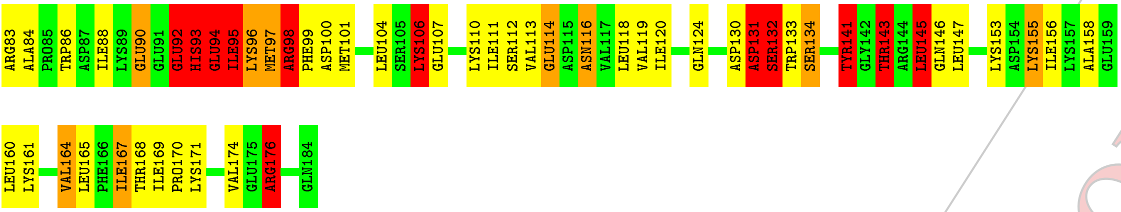

• Molecule 2:

Chain H:

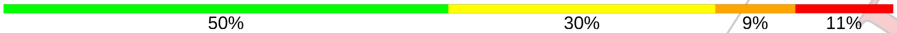

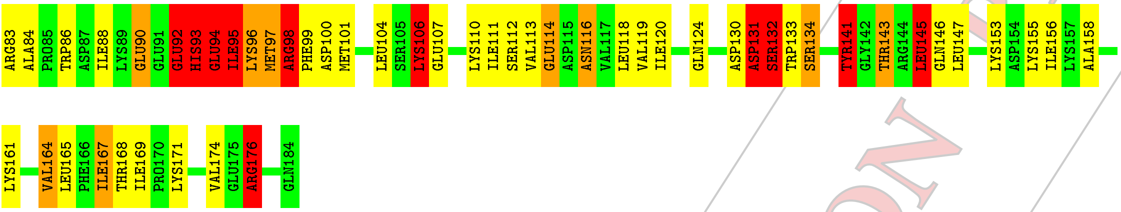

• Molecule 2:

Chain K:

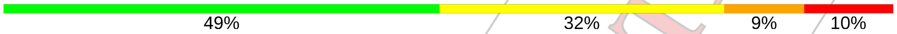

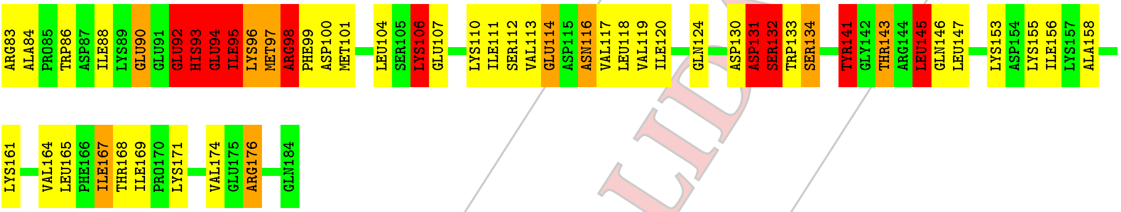

• Molecule 2:

Chain L:

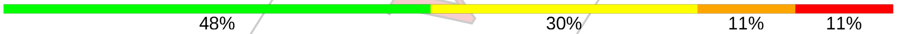

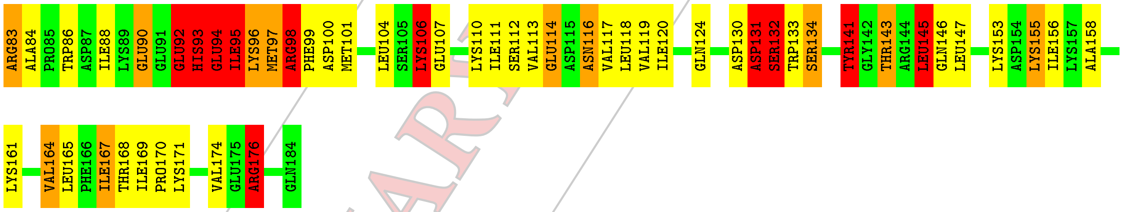

PRELIMINARY VALIDATION REPORT

4 Experimental information (i)

Property	Value	Source
Reconstruction method	Not provided	Depositor
Imposed symmetry	POINT, Not provided	Depositor
Number of images used	Not provided	Depositor
Resolution determination method	Not provided	Depositor
CTF correction method	Not provided	Depositor
Microscope	Not provided	Depositor
Voltage (kV)	Not provided	Depositor
Electron dose $(e^{-}/\text{Å}^{2})$	Not provided	Depositor
Minimum defocus (nm)	Not provided	Depositor
Maximum defocus (nm)	Not provided	Depositor
Magnification	Not provided	Depositor
Image detector	Not provided	Depositor

PRELIMINARY VALIDATION REPORT

5 Model quality (i)

5.1 Standard geometry (i)

The Z score for a bond length (or angle) is the number of standard deviations the observed value is removed from the expected value. A bond length (or angle) with $|Z| > 5$ is considered an outlier worth inspection. RMSZ is the root-mean-square of all Z scores of the bond lengths (or angles).

Mol	Chain	Bond lengths		Bond angles	
		RMSZ	$\#\|Z\| >2$	RMSZ	$\#\|Z\| >2$
1	A	0.76	0/1157	1.51	22/1549 (1.4%)
1	C	0.76	0/1157	1.51	22/1549 (1.4%)
1	D	0.75	0/1157	1.51	22/1549 (1.4%)
1	G	0.77	0/1157	1.51	22/1549 (1.4%)
1	I	0.77	0/1157	1.52	22/1549 (1.4%)
1	J	0.77	0/1157	1.51	22/1549 (1.4%)
2	B	0.76	0/836	2.00	15/1119 (1.3%)
2	E	0.76	0/836	2.03	15/1119 (1.3%)
2	F	0.75	0/836	2.02	14/1119 (1.3%)
2	H	0.77	0/836	2.07	13/1119 (1.2%)
2	K	0.79	0/836	1.89	15/1119 (1.3%)
2	L	0.78	0/836	1.99	15/1119 (1.3%)
All	All	0.77	0/11958	1.73	219/16008 (1.4%)

Chiral center outliers are detected by calculating the chiral volume of a chiral center and verifying if the center is modelled as a planar moiety or with the opposite hand.A planarity outlier is detected by checking planarity of atoms in a peptide group, atoms in a mainchain group or atoms of a sidechain that are expected to be planar.

Mol	Chain	#Chirality outliers	#Planarity outliers
1	A	0	17
1	C	0	17
1	D	0	17
1	G	0	17
1	I	0	20
1	J	0	17
2	B	0	12
2	E	0	14
2	F	0	12
2	H	0	14
2	K	0	14
2	L	0	13
All	All	0	184

PRELIMINARY VALIDATION REPORT

There are no bond length outliers.

All (219) bond angle outliers are listed below:

Mol	Chain	Res	Type	Atoms	Z	Observed(^{o})	Ideal(^{o})
2	H	98	ARG	NE-CZ-NH1	37.70	139.15	120.30
2	F	98	ARG	NE-CZ-NH1	35.83	138.21	120.30
2	E	98	ARG	NE-CZ-NH1	35.53	138.07	120.30
2	B	98	ARG	NE-CZ-NH1	35.22	137.91	120.30
2	L	98	ARG	NE-CZ-NH1	34.52	137.56	120.30
2	K	98	ARG	NE-CZ-NH1	28.21	134.40	120.30
2	H	98	ARG	NH1-CZ-NH2	-20.89	96.42	119.40
2	F	98	ARG	NH1-CZ-NH2	-20.79	96.53	119.40
2	E	98	ARG	NH1-CZ-NH2	-20.26	97.12	119.40
2	B	98	ARG	NH1-CZ-NH2	-19.93	97.48	119.40
2	L	98	ARG	NH1-CZ-NH2	-19.34	98.12	119.40
2	K	132	SER	CA-CB-OG	14.91	151.47	111.20
2	H	132	SER	CA-CB-OG	14.74	150.99	111.20
2	E	132	SER	CA-CB-OG	14.51	150.37	111.20
2	K	98	ARG	NH1-CZ-NH2	-14.35	103.61	119.40
2	F	132	SER	CA-CB-OG	13.63	148.01	111.20
2	B	132	SER	CA-CB-OG	13.60	147.92	111.20
2	L	132	SER	CA-CB-OG	13.58	147.87	111.20
1	C	98	ARG	NE-CZ-NH1	12.40	126.50	120.30
1	I	98	ARG	NE-CZ-NH1	12.26	126.43	120.30
1	G	98	ARG	NE-CZ-NH1	11.97	126.28	120.30
1	A	98	ARG	NE-CZ-NH1	11.97	126.28	120.30
1	J	98	ARG	NE-CZ-NH1	11.96	126.28	120.30
1	D	98	ARG	NE-CZ-NH1	11.92	126.26	120.30
2	L	141	TYR	CB-CG-CD2	-10.36	114.78	121.00
2	K	141	TYR	CB-CG-CD2	-10.32	114.81	121.00
2	F	141	TYR	CB-CG-CD2	-10.30	114.82	121.00
2	H	141	TYR	CB-CG-CD2	-10.29	114.83	121.00
2	H	98	ARG	CD-NE-CZ	10.22	137.91	123.60
2	F	98	ARG	CD-NE-CZ	9.71	137.20	123.60
2	E	98	ARG	CD-NE-CZ	9.63	137.09	123.60
2	F	106	LYS	CA-CB-CG	9.62	134.55	113.40
2	K	106	LYS	CA-CB-CG	9.60	134.51	113.40
2	L	106	LYS	CA-CB-CG	9.59	134.51	113.40
2	E	106	LYS	CA-CB-CG	9.59	134.50	113.40
2	B	106	LYS	CA-CB-CG	9.56	134.44	113.40
2	B	141	TYR	CB-CG-CD2	-9.56	115.26	121.00
2	H	106	LYS	CA-CB-CG	9.55	134.41	113.40
2	B	98	ARG	CD-NE-CZ	9.43	136.80	123.60
2	E	141	TYR	CB-CG-CD2	-9.40	115.36	121.00

Continued on next page...

PRELIMINARY VALIDATION REPORT

Continued from previous page...

Mol	Chain	Res	Type	Atoms	Z	Observed(^{o})	Ideal(^{o})
2	E	132	SER	N-CA-CB	9.25	124.38	110.50
2	L	98	ARG	CD-NE-CZ	9.18	136.46	123.60
2	K	132	SER	N-CA-CB	9.12	124.17	110.50
2	K	98	ARG	CD-NE-CZ	8.92	136.09	123.60
2	B	95	ILE	CA-CB-CG1	8.71	127.55	111.00
2	K	95	ILE	CA-CB-CG1	8.70	127.54	111.00
2	E	95	ILE	CA-CB-CG1	8.69	127.50	111.00
2	L	95	ILE	CA-CB-CG1	8.67	127.48	111.00
2	H	95	ILE	CA-CB-CG1	8.34	126.85	111.00
2	F	95	ILE	CA-CB-CG1	8.31	126.79	111.00
2	H	145	LEU	CB-CG-CD2	8.24	125.00	111.00
2	F	145	LEU	CB-CG-CD2	8.21	124.95	111.00
1	D	55	MET	CA-CB-CG	8.13	127.12	113.30
1	G	55	MET	CA-CB-CG	8.12	127.10	113.30
2	B	145	LEU	CB-CG-CD2	8.11	124.79	111.00
2	E	145	LEU	CB-CG-CD2	8.10	124.78	111.00
2	K	145	LEU	CB-CG-CD2	8.10	124.76	111.00
2	L	145	LEU	CB-CG-CD2	8.10	124.77	111.00
1	C	55	MET	CA-CB-CG	8.09	127.05	113.30
1	I	55	MET	CA-CB-CG	8.08	127.04	113.30
1	I	100	ASP	CB-CG-OD1	-7.86	111.23	118.30
2	L	106	LYS	CG-CD-CE	7.83	135.38	111.90
2	B	106	LYS	CG-CD-CE	7.83	135.38	111.90
2	F	106	LYS	CG-CD-CE	7.83	135.38	111.90
2	K	106	LYS	CG-CD-CE	7.83	135.38	111.90
2	H	106	LYS	CG-CD-CE	7.82	135.35	111.90
2	E	106	LYS	CG-CD-CE	7.81	135.34	111.90
1	A	55	MET	CA-CB-CG	7.81	126.57	113.30
1	G	100	ASP	CB-CG-OD1	-7.80	111.28	118.30
1	J	55	MET	CA-CB-CG	7.77	126.51	113.30
2	B	131	ASP	CB-CG-OD2	-7.39	111.65	118.30
2	L	131	ASP	CB-CG-OD2	-7.39	111.65	118.30
2	E	131	ASP	CB-CG-OD2	-7.30	111.73	118.30
2	H	131	ASP	CB-CG-OD2	-7.25	111.77	118.30
2	K	131	ASP	CB-CG-OD2	-7.21	111.81	118.30
2	F	131	ASP	CB-CG-OD2	-7.20	111.82	118.30
1	A	60	ASP	CB-CG-OD2	-7.18	111.84	118.30
1	J	100	ASP	CB-CG-OD1	-7.17	111.84	118.30
1	D	60	ASP	CB-CG-OD2	-7.16	111.86	118.30
1	C	60	ASP	CB-CG-OD2	-7.15	111.87	118.30
1	D	176	ARG	NE-CZ-NH1	7.07	123.84	120.30
1	A	100	ASP	CB-CG-OD1	-7.05	111.95	118.30

Continued on next page...

PRELIMINARY VALIDATION REPORT

Continued from previous page...

Mol	Chain	Res	Type	Atoms	Z	Observed(°)	Ideal(°)
1	I	60	ASP	CB-CG-OD2	-7.05	111.96	118.30
1	G	176	ARG	NE-CZ-NH1	7.01	123.81	120.30
1	J	60	ASP	CB-CG-OD2	-7.01	111.99	118.30
1	A	184	GLN	CA-CB-CG	6.95	128.69	113.40
1	J	184	GLN	CA-CB-CG	6.95	128.69	113.40
1	A	176	ARG	NE-CZ-NH1	6.94	123.77	120.30
1	G	184	GLN	CA-CB-CG	6.94	128.67	113.40
1	I	184	GLN	CA-CB-CG	6.94	128.67	113.40
1	D	184	GLN	CA-CB-CG	6.93	128.65	113.40
1	C	184	GLN	CA-CB-CG	6.91	128.60	113.40
1	J	53	ARG	NE-CZ-NH2	-6.86	116.87	120.30
1	G	53	ARG	NE-CZ-NH2	-6.85	116.88	120.30
1	J	176	ARG	NE-CZ-NH1	6.83	123.72	120.30
1	G	60	ASP	CB-CG-OD2	-6.83	112.15	118.30
1	C	100	ASP	CB-CG-OD1	-6.82	112.17	118.30
1	C	176	ARG	NE-CZ-NH1	6.80	123.70	120.30
1	I	53	ARG	NE-CZ-NH2	-6.78	116.91	120.30
1	I	176	ARG	NE-CZ-NH1	6.75	123.68	120.30
1	C	96	LYS	CB-CA-C	6.75	123.89	110.40
1	C	53	ARG	NE-CZ-NH2	-6.73	116.93	120.30
1	J	96	LYS	CB-CA-C	6.71	123.81	110.40
1	A	44	ASP	CB-CG-OD2	-6.69	112.28	118.30
1	A	53	ARG	NE-CZ-NH2	-6.68	116.96	120.30
1	A	96	LYS	CB-CA-C	6.68	123.75	110.40
1	D	44	ASP	CB-CG-OD2	-6.66	112.31	118.30
1	D	96	LYS	CB-CA-C	6.65	123.69	110.40
1	G	96	LYS	CB-CA-C	6.64	123.68	110.40
1	I	96	LYS	CB-CA-C	6.60	123.61	110.40
1	J	184	GLN	CB-CG-CD	6.58	128.71	111.60
1	I	184	GLN	CB-CG-CD	6.56	128.65	111.60
1	G	184	GLN	CB-CG-CD	6.56	128.65	111.60
1	C	44	ASP	CB-CG-OD2	-6.55	112.40	118.30
1	A	184	GLN	CB-CG-CD	6.55	128.63	111.60
1	D	184	GLN	CB-CG-CD	6.55	128.63	111.60
1	C	184	GLN	CB-CG-CD	6.53	128.57	111.60
1	I	44	ASP	CB-CG-OD2	-6.51	112.44	118.30
1	D	53	ARG	NE-CZ-NH2	-6.50	117.05	120.30
1	J	44	ASP	CB-CG-OD2	-6.50	112.45	118.30
1	G	44	ASP	CB-CG-OD2	-6.44	112.50	118.30
2	H	131	ASP	CB-CG-OD1	-6.38	112.56	118.30
2	K	131	ASP	CB-CG-OD1	-6.38	112.56	118.30
1	A	95	ILE	N-CA-C	-6.34	93.89	111.00

Continued on next page...

PRELIMINARY VALIDATION REPORT

Continued from previous page...

Mol	Chain	Res	Type	Atoms	Z	Observed(°)	Ideal(°)
1	D	95	ILE	N-CA-C	-6.33	93.90	111.00
1	D	100	ASP	CB-CG-OD1	-6.32	112.62	118.30
2	F	131	ASP	CB-CG-OD1	-6.31	112.62	118.30
1	J	95	ILE	N-CA-C	-6.31	93.97	111.00
1	C	95	ILE	N-CA-C	-6.31	93.97	111.00
1	I	95	ILE	N-CA-C	-6.30	93.98	111.00
2	B	131	ASP	CB-CG-OD1	-6.30	112.63	118.30
1	G	95	ILE	N-CA-C	-6.30	94.00	111.00
2	L	131	ASP	CB-CG-OD1	-6.26	112.66	118.30
2	E	131	ASP	CB-CG-OD1	-6.26	112.67	118.30
1	A	95	ILE	N-CA-CB	6.13	124.91	110.80
1	I	95	ILE	N-CA-CB	6.12	124.86	110.80
1	J	95	ILE	N-CA-CB	6.11	124.86	110.80
1	D	95	ILE	N-CA-CB	6.11	124.85	110.80
1	C	95	ILE	N-CA-CB	6.11	124.85	110.80
1	G	95	ILE	N-CA-CB	6.09	124.81	110.80
2	F	164	VAL	CG1-CB-CG2	5.98	120.47	110.90
2	H	164	VAL	CG1-CB-CG2	5.95	120.41	110.90
2	E	164	VAL	CG1-CB-CG2	5.92	120.37	110.90
1	I	134	SER	CB-CA-C	5.91	121.33	110.10
1	A	184	GLN	OE1-CD-NE2	-5.88	108.37	121.90
1	G	184	GLN	OE1-CD-NE2	-5.87	108.41	121.90
1	J	184	GLN	OE1-CD-NE2	-5.85	108.44	121.90
1	I	184	GLN	OE1-CD-NE2	-5.84	108.47	121.90
1	C	184	GLN	OE1-CD-NE2	-5.82	108.53	121.90
1	D	184	GLN	OE1-CD-NE2	-5.81	108.54	121.90
1	J	166	PHE	CB-CG-CD1	-5.81	116.73	120.80
2	L	164	VAL	CG1-CB-CG2	5.77	120.13	110.90
1	G	166	PHE	CB-CG-CD1	-5.76	116.77	120.80
2	B	164	VAL	CG1-CB-CG2	5.76	120.12	110.90
1	A	166	PHE	CB-CG-CD1	-5.74	116.78	120.80
1	C	98	ARG	NH1-CZ-NH2	-5.73	113.09	119.40
1	C	166	PHE	CB-CG-CD1	-5.72	116.80	120.80
1	I	98	ARG	NH1-CZ-NH2	-5.67	113.16	119.40
1	I	166	PHE	CB-CG-CD1	-5.66	116.84	120.80
1	D	166	PHE	CB-CG-CD1	-5.65	116.84	120.80
2	K	176	ARG	NE-CZ-NH2	-5.62	117.49	120.30
2	L	176	ARG	NE-CZ-NH2	-5.57	117.52	120.30
2	H	176	ARG	NE-CZ-NH2	-5.57	117.52	120.30
1	D	98	ARG	NH1-CZ-NH2	-5.56	113.29	119.40
1	J	98	ARG	NH1-CZ-NH2	-5.55	113.29	119.40
1	G	98	ARG	NH1-CZ-NH2	-5.55	113.29	119.40

Continued on next page...

PRELIMINARY VALIDATION REPORT

Continued from previous page...

Mol	Chain	Res	Type	Atoms	Z	Observed(°)	Ideal(°)
2	B	176	ARG	NE-CZ-NH2	-5.55	117.53	120.30
1	G	144	ARG	NE-CZ-NH1	5.54	123.07	120.30
2	E	176	ARG	NE-CZ-NH2	-5.53	117.53	120.30
1	A	55	MET	CB-CA-C	-5.53	99.34	110.40
1	A	98	ARG	NH1-CZ-NH2	-5.52	113.32	119.40
1	J	55	MET	CB-CA-C	-5.50	99.39	110.40
1	I	144	ARG	NE-CZ-NH1	5.46	123.03	120.30
1	A	43	LEU	CB-CG-CD1	5.43	120.23	111.00
1	C	43	LEU	CB-CG-CD1	5.42	120.21	111.00
1	C	99	PHE	CB-CA-C	5.42	121.23	110.40
1	D	144	ARG	NE-CZ-NH1	5.41	123.01	120.30
1	A	144	ARG	NE-CZ-NH1	5.41	123.01	120.30
1	J	43	LEU	CB-CG-CD1	5.40	120.18	111.00
1	C	144	ARG	NE-CZ-NH1	5.39	123.00	120.30
1	D	99	PHE	CB-CA-C	5.39	121.18	110.40
1	I	99	PHE	CB-CA-C	5.38	121.17	110.40
1	D	74	ARG	NE-CZ-NH1	5.38	122.99	120.30
1	D	43	LEU	CB-CG-CD1	5.38	120.14	111.00
1	C	74	ARG	NE-CZ-NH1	5.37	122.98	120.30
1	G	43	LEU	CB-CG-CD1	5.36	120.12	111.00
1	I	43	LEU	CB-CG-CD1	5.36	120.11	111.00
1	J	99	PHE	CB-CA-C	5.34	121.09	110.40
1	J	144	ARG	NE-CZ-NH1	5.34	122.97	120.30
1	A	99	PHE	CB-CA-C	5.34	121.07	110.40
1	I	74	ARG	NE-CZ-NH1	5.33	122.97	120.30
1	G	99	PHE	CB-CA-C	5.33	121.05	110.40
2	F	176	ARG	NE-CZ-NH2	-5.32	117.64	120.30
1	C	55	MET	CB-CA-C	-5.31	99.77	110.40
1	D	55	MET	CB-CA-C	-5.31	99.78	110.40
1	G	55	MET	CB-CA-C	-5.28	99.83	110.40
1	I	55	MET	CB-CA-C	-5.28	99.83	110.40
1	J	74	ARG	NE-CZ-NH1	5.28	122.94	120.30
1	A	74	ARG	NE-CZ-NH1	5.28	122.94	120.30
1	J	134	SER	CB-CA-C	5.27	120.11	110.10
1	G	74	ARG	NE-CZ-NH1	5.26	122.93	120.30
1	A	134	SER	CB-CA-C	5.20	119.97	110.10
1	C	134	SER	CB-CA-C	5.19	119.97	110.10
1	C	96	LYS	N-CA-CB	-5.13	101.37	110.60
1	D	134	SER	CB-CA-C	5.12	119.83	110.10
1	G	134	SER	CB-CA-C	5.12	119.83	110.10
1	I	96	LYS	N-CA-CB	-5.10	101.42	110.60
2	B	98	ARG	NE-CZ-NH2	5.08	122.84	120.30

Continued on next page...

PRELIMINARY VALIDATION REPORT

Continued from previous page...

Mol	Chain	Res	Type	Atoms	Z	Observed(^{o})	Ideal(^{o})
1	D	96	LYS	N-CA-CB	-5.07	101.47	110.60
2	K	98	ARG	NE-CZ-NH2	-5.07	117.77	120.30
1	G	96	LYS	N-CA-CB	-5.06	101.49	110.60
1	J	96	LYS	N-CA-CB	-5.05	101.51	110.60
2	L	141	TYR	CB-CG-CD1	5.04	124.02	121.00
2	K	141	TYR	CB-CG-CD1	5.02	124.01	121.00
2	L	83	ARG	NE-CZ-NH1	5.02	122.81	120.30
2	E	143	THR	N-CA-CB	5.02	119.83	110.30
2	F	143	THR	N-CA-CB	5.01	119.82	110.30
1	A	96	LYS	N-CA-CB	-5.00	101.60	110.60
2	B	143	THR	N-CA-CB	5.00	119.80	110.30

There are no chirality outliers.

All (184) planarity outliers are listed below:

Mol	Chain	Res	Type	Group
1	A	100	ASP	Sidechain
1	A	128	ASP	Sidechain
1	A	135	GLY	Mainchain
1	A	159	GLU	Sidechain
1	A	169	ILE	Mainchain
1	A	42	LEU	Mainchain
1	A	44	ASP	Sidechain,Mainchain
1	A	49	MET	Mainchain
1	A	51	THR	Peptide
1	A	53	ARG	Sidechain
1	A	60	ASP	Sidechain
1	A	81	GLU	Mainchain
1	A	90	GLU	Sidechain
1	A	92	GLU	Peptide
1	A	94	GLU	Mainchain,Peptide
2	B	100	ASP	Sidechain
2	B	114	GLU	Sidechain
2	B	131	ASP	Sidechain
2	B	90	GLU	Sidechain
2	B	92	GLU	Sidechain
2	B	93	HIS	Sidechain,Peptide
2	B	94	GLU	Mainchain
2	B	96	LYS	Mainchain
2	B	97	MET	Peptide
2	B	98	ARG	Sidechain,Mainchain
1	C	100	ASP	Sidechain

Continued on next page...

PRELIMINARY VALIDATION REPORT

Continued from previous page...

Mol	Chain	Res	Type	Group
1	C	128	ASP	Sidechain
1	C	135	GLY	Mainchain
1	C	159	GLU	Sidechain
1	C	169	ILE	Mainchain
1	C	42	LEU	Mainchain
1	C	44	ASP	Sidechain,Mainchain
1	C	49	MET	Mainchain
1	C	51	THR	Peptide
1	C	53	ARG	Sidechain
1	C	60	ASP	Sidechain
1	C	81	GLU	Mainchain
1	C	90	GLU	Sidechain
1	C	92	GLU	Peptide
1	C	94	GLU	Mainchain,Peptide
1	D	100	ASP	Sidechain
1	D	128	ASP	Sidechain
1	D	135	GLY	Mainchain
1	D	159	GLU	Sidechain
1	D	169	ILE	Mainchain
1	D	42	LEU	Mainchain
1	D	44	ASP	Sidechain,Mainchain
1	D	49	MET	Mainchain
1	D	51	THR	Peptide
1	D	53	ARG	Sidechain
1	D	60	ASP	Sidechain
1	D	81	GLU	Mainchain
1	D	90	GLU	Sidechain
1	D	92	GLU	Peptide
1	D	94	GLU	Mainchain,Peptide
2	E	100	ASP	Sidechain
2	E	114	GLU	Sidechain
2	E	131	ASP	Sidechain
2	E	132	SER	Mainchain
2	E	90	GLU	Sidechain
2	E	92	GLU	Sidechain
2	E	93	HIS	Sidechain,Mainchain,Peptide
2	E	94	GLU	Mainchain
2	E	96	LYS	Mainchain
2	E	97	MET	Peptide
2	E	98	ARG	Sidechain,Mainchain
2	F	100	ASP	Sidechain
2	F	114	GLU	Sidechain

Continued on next page...

PRELIMINARY VALIDATION REPORT

Continued from previous page...

Mol	Chain	Res	Type	Group
2	F	131	ASP	Sidechain
2	F	90	GLU	Sidechain
2	F	92	GLU	Sidechain
2	F	93	HIS	Sidechain,Peptide
2	F	94	GLU	Mainchain
2	F	96	LYS	Mainchain
2	F	97	MET	Peptide
2	F	98	ARG	Sidechain,Mainchain
1	G	100	ASP	Sidechain
1	G	128	ASP	Sidechain
1	G	135	GLY	Mainchain
1	G	159	GLU	Sidechain
1	G	169	ILE	Mainchain
1	G	42	LEU	Mainchain
1	G	44	ASP	Sidechain,Mainchain
1	G	49	MET	Mainchain
1	G	51	THR	Peptide
1	G	53	ARG	Sidechain
1	G	60	ASP	Sidechain
1	G	81	GLU	Mainchain
1	G	90	GLU	Sidechain
1	G	92	GLU	Peptide
1	G	94	GLU	Mainchain,Peptide
2	H	100	ASP	Sidechain
2	H	114	GLU	Sidechain
2	H	131	ASP	Sidechain
2	H	132	SER	Mainchain
2	H	90	GLU	Sidechain
2	H	92	GLU	Sidechain
2	H	93	HIS	Sidechain,Peptide
2	H	94	GLU	Mainchain
2	H	96	LYS	Mainchain
2	H	97	MET	Mainchain,Peptide
2	H	98	ARG	Sidechain,Mainchain
1	I	100	ASP	Sidechain
1	I	128	ASP	Sidechain
1	I	133	TRP	Peptide
1	I	134	SER	Peptide
1	I	135	GLY	Mainchain,Peptide
1	I	159	GLU	Sidechain
1	I	169	ILE	Mainchain
1	I	42	LEU	Mainchain

Continued on next page...

PRELIMINARY VALIDATION REPORT

Continued from previous page...

Mol	Chain	Res	Type	Group
1	I	44	ASP	Sidechain,Mainchain
1	I	49	MET	Mainchain
1	I	51	THR	Peptide
1	I	53	ARG	Sidechain
1	I	60	ASP	Sidechain
1	I	81	GLU	Mainchain
1	I	90	GLU	Sidechain
1	I	92	GLU	Peptide
1	I	94	GLU	Mainchain,Peptide
1	J	100	ASP	Sidechain
1	J	128	ASP	Sidechain
1	J	135	GLY	Mainchain
1	J	159	GLU	Sidechain
1	J	169	ILE	Mainchain
1	J	42	LEU	Mainchain
1	J	44	ASP	Sidechain,Mainchain
1	J	49	MET	Mainchain
1	J	51	THR	Peptide
1	J	53	ARG	Sidechain
1	J	60	ASP	Sidechain
1	J	81	GLU	Mainchain
1	J	90	GLU	Sidechain
1	J	92	GLU	Peptide
1	J	94	GLU	Mainchain,Peptide
2	K	100	ASP	Sidechain
2	K	114	GLU	Sidechain
2	K	131	ASP	Sidechain
2	K	132	SER	Mainchain
2	K	90	GLU	Sidechain
2	K	92	GLU	Sidechain
2	K	93	HIS	Sidechain,Mainchain,Peptide
2	K	94	GLU	Mainchain
2	K	96	LYS	Mainchain
2	K	97	MET	Peptide
2	K	98	ARG	Sidechain,Mainchain
2	L	100	ASP	Sidechain
2	L	114	GLU	Sidechain
2	L	131	ASP	Sidechain
2	L	90	GLU	Sidechain
2	L	92	GLU	Sidechain
2	L	93	HIS	Sidechain,Mainchain,Peptide
2	L	94	GLU	Mainchain

Continued on next page...

PRELIMINARY VALIDATION REPORT

Continued from previous page...

Mol	Chain	Res	Type	Group
2	L	96	LYS	Mainchain
2	L	97	MET	Peptide
2	L	98	ARG	Sidechain,Mainchain

5.2 Too-close contacts (i)

In the following table, the Non-H and H(model) columns list the number of non-hydrogen atoms and hydrogen atoms in the chain respectively. The H(added) column lists the number of hydrogen atoms added and optimized by MolProbity. The Clashes column lists the number of clashes within the asymmetric unit, whereas Symm-Clashes lists symmetry related clashes.

Mol	Chain	Non-H	H(model)	H(added)	Clashes	Symm-Clashes
1	A	1142	1159	1156	145	0
1	C	1142	1159	1156	144	0
1	D	1142	1159	1156	145	0
1	G	1142	1159	1156	150	0
1	I	1142	1159	1156	147	0
1	J	1142	1159	1156	141	0
2	B	825	842	839	120	0
2	E	825	842	839	115	0
2	F	825	842	839	115	0
2	H	825	842	839	119	0
2	K	825	842	839	122	0
2	L	825	842	839	118	0
All	All	11802	12006	11970	1195	0

The all-atom clashscore is defined as the number of clashes found per 1000 atoms (including hydrogen atoms). The all-atom clashscore for this structure is 50.

All (1195) close contacts within the same asymmetric unit are listed below, sorted by their clash magnitude.

Atom-1	Atom-2	Interatomic distance (Å)	Clash overlap (Å)
2:B:98:ARG:HH21	2:B:164:VAL:HG21	1.21	1.05
2:H:98:ARG:HH21	2:H:164:VAL:HG21	1.20	1.05
2:F:98:ARG:HH21	2:F:164:VAL:HG21	1.21	1.04
2:E:98:ARG:HH21	2:E:164:VAL:HG21	1.23	1.04
2:L:98:ARG:HH21	2:L:164:VAL:HG21	1.24	1.00
2:K:98:ARG:NH2	2:K:164:VAL:HG21	1.80	0.96
2:H:98:ARG:NH2	2:H:164:VAL:HG21	1.86	0.91
2:F:98:ARG:NH2	2:F:164:VAL:HG21	1.86	0.91

Continued on next page...

PRELIMINARY VALIDATION REPORT

Continued from previous page...

Atom-1	Atom-2	Interatomic distance (Å)	Clash overlap (Å)
2:L:98:ARG:NH2	2:L:164:VAL:HG21	1.85	0.90
2:E:98:ARG:NH2	2:E:164:VAL:HG21	1.86	0.90
1:I:135:GLY:HA2	2:K:98:ARG:HH11	1.35	0.90
2:B:98:ARG:NH2	2:B:164:VAL:HG21	1.85	0.89
2:H:143:THR:HB	1:J:53:ARG:HG3	1.60	0.82
1:A:53:ARG:HG3	2:F:143:THR:HB	1.61	0.82
2:H:99:PHE:CE1	2:H:143:THR:HG21	2.14	0.82
2:F:99:PHE:CE1	2:F:143:THR:HG21	2.14	0.82
2:B:143:THR:HB	1:C:53:ARG:HG3	1.60	0.81
2:E:143:THR:HB	1:D:53:ARG:HG3	1.61	0.81
1:G:53:ARG:HG3	2:K:143:THR:HB	1.61	0.81
2:B:118:LEU:HB2	2:B:147:LEU:HD21	1.62	0.81
1:I:53:ARG:HG3	2:L:143:THR:HB	1.60	0.81
2:L:118:LEU:HB2	2:L:147:LEU:HD21	1.62	0.81
2:K:99:PHE:CE1	2:K:143:THR:HG21	2.15	0.81
2:E:99:PHE:CE1	2:E:143:THR:HG21	2.15	0.81
2:B:99:PHE:CE1	2:B:143:THR:HG21	2.14	0.81
2:H:118:LEU:HB2	2:H:147:LEU:HD21	1.62	0.81
2:L:99:PHE:CE1	2:L:143:THR:HG21	2.15	0.80
2:F:118:LEU:HB2	2:F:147:LEU:HD21	1.62	0.80
1:I:55:MET:SD	2:L:97:MET:HE3	2.22	0.80
2:K:118:LEU:HB2	2:K:147:LEU:HD21	1.62	0.80
2:E:118:LEU:HB2	2:E:147:LEU:HD21	1.62	0.80
2:E:118:LEU:HD12	2:E:145:LEU:HD23	1.65	0.79
2:B:118:LEU:HD12	2:B:145:LEU:HD23	1.65	0.79
2:K:118:LEU:HD12	2:K:145:LEU:HD23	1.65	0.79
2:L:118:LEU:HD12	2:L:145:LEU:HD23	1.65	0.79
2:H:84:ALA:HB1	2:H:97:MET:SD	2.23	0.79
2:F:84:ALA:HB1	2:F:97:MET:SD	2.23	0.78
2:B:97:MET:HE3	1:C:55:MET:SD	2.24	0.78
1:I:96:LYS:HE2	2:K:132:SER:N	1.97	0.78
2:K:84:ALA:HB1	2:K:97:MET:SD	2.23	0.78
1:C:96:LYS:HE2	2:E:132:SER:N	1.97	0.78
2:E:84:ALA:HB1	2:E:97:MET:SD	2.24	0.78
2:L:84:ALA:HB1	2:L:97:MET:SD	2.23	0.77
2:E:97:MET:HE3	1:D:55:MET:SD	2.25	0.77
2:F:118:LEU:HD12	2:F:145:LEU:HD23	1.65	0.77
1:A:96:LYS:HE2	2:B:132:SER:N	2.00	0.77
2:B:84:ALA:HB1	2:B:97:MET:SD	2.24	0.77
2:H:118:LEU:HD12	2:H:145:LEU:HD23	1.65	0.77
1:G:96:LYS:HE2	2:H:132:SER:N	1.98	0.76

Continued on next page...

PRELIMINARY VALIDATION REPORT

Continued from previous page...

Atom-1	Atom-2	Interatomic distance (Å)	Clash overlap (Å)
1:J:96:LYS:HE2	2:L:132:SER:N	2.01	0.76
2:B:98:ARG:CZ	2:B:164:VAL:HG11	2.15	0.76
1:I:135:GLY:HA2	2:K:98:ARG:NH1	2.01	0.76
2:F:98:ARG:CZ	2:F:164:VAL:HG11	2.16	0.75
2:L:98:ARG:CZ	2:L:164:VAL:HG11	2.17	0.75
1:D:96:LYS:HE2	2:F:132:SER:N	2.00	0.75
1:J:98:ARG:HB2	2:L:132:SER:HB3	1.69	0.75
2:H:98:ARG:CZ	2:H:164:VAL:HG11	2.17	0.74
2:E:98:ARG:CZ	2:E:164:VAL:HG11	2.18	0.74
2:K:98:ARG:CZ	2:K:164:VAL:HG11	2.19	0.73
1:G:55:MET:SD	2:K:97:MET:HE3	2.29	0.73
1:G:98:ARG:HG3	2:H:132:SER:HB2	1.70	0.72
1:C:135:GLY:HA2	2:E:98:ARG:HH11	1.54	0.72
1:I:106:LYS:HD3	1:I:106:LYS:H	1.55	0.72
1:J:135:GLY:HA2	2:L:98:ARG:HH11	1.55	0.72
1:C:106:LYS:H	1:C:106:LYS:HD3	1.55	0.72
1:I:134:SER:HB3	2:K:98:ARG:HH21	1.54	0.72
1:I:184:GLN:HA	2:L:106:LYS:HD2	1.70	0.72
2:H:106:LYS:HD2	1:J:184:GLN:HA	1.70	0.72
1:A:184:GLN:HA	2:F:106:LYS:HD2	1.71	0.71
1:A:179:ILE:HG22	2:F:112:SER:HB2	1.72	0.71
1:G:106:LYS:HD3	1:G:106:LYS:H	1.55	0.71
1:G:53:ARG:HH12	2:K:141:TYR:HB2	1.55	0.71
2:B:106:LYS:HD2	1:C:184:GLN:HA	1.70	0.71
1:D:106:LYS:HD3	1:D:106:LYS:H	1.55	0.71
1:G:184:GLN:HA	2:K:106:LYS:HD2	1.70	0.71
2:H:112:SER:HB2	1:J:179:ILE:HG22	1.72	0.71
2:H:141:TYR:HB2	1:J:53:ARG:HH12	1.54	0.71
2:E:106:LYS:HD2	1:D:184:GLN:HA	1.70	0.71
1:A:53:ARG:HH12	2:F:141:TYR:HB2	1.55	0.71
2:E:141:TYR:HB2	1:D:53:ARG:HH12	1.56	0.70
2:E:112:SER:HB2	1:D:179:ILE:HG22	1.72	0.70
1:I:53:ARG:HH12	2:L:141:TYR:HB2	1.55	0.70
1:I:179:ILE:HG22	2:L:112:SER:HB2	1.72	0.70
2:B:112:SER:HB2	1:C:179:ILE:HG22	1.72	0.70
1:J:106:LYS:HD3	1:J:106:LYS:H	1.55	0.70
1:G:179:ILE:HG22	2:K:112:SER:HB2	1.72	0.70
2:B:145:LEU:HD12	1:C:55:MET:HG2	1.73	0.70
1:D:99:PHE:CZ	1:D:109:VAL:HG21	2.26	0.70
1:G:99:PHE:CZ	1:G:109:VAL:HG21	2.26	0.70
2:H:120:ILE:HB	2:H:143:THR:HG23	1.73	0.70

Continued on next page...

PRELIMINARY VALIDATION REPORT

Continued from previous page...

Atom-1	Atom-2	Interatomic distance (Å)	Clash overlap (Å)
1:I:55:MET:HG2	2:L:145:LEU:HD12	1.73	0.70
1:A:106:LYS:HD3	1:A:106:LYS:H	1.55	0.69
2:B:141:TYR:HB2	1:C:53:ARG:HH12	1.56	0.69
1:C:99:PHE:CZ	1:C:109:VAL:HG21	2.26	0.69
2:B:92:GLU:HG3	2:B:168:THR:CG2	2.22	0.69
2:E:92:GLU:HG3	2:E:168:THR:CG2	2.22	0.69
2:F:120:ILE:HB	2:F:143:THR:HG23	1.73	0.69
2:H:145:LEU:HD12	1:J:55:MET:HG2	1.74	0.69
1:I:99:PHE:CZ	1:I:109:VAL:HG21	2.26	0.69
1:A:99:PHE:CZ	1:A:109:VAL:HG21	2.26	0.69
1:J:99:PHE:CZ	1:J:109:VAL:HG21	2.26	0.69
2:K:92:GLU:HG3	2:K:168:THR:CG2	2.22	0.69
2:L:92:GLU:HG3	2:L:168:THR:CG2	2.22	0.69
1:A:55:MET:HG2	2:F:145:LEU:HD12	1.74	0.69
1:D:135:GLY:HA2	2:F:98:ARG:HH11	1.56	0.69
2:L:120:ILE:HB	2:L:143:THR:HG23	1.73	0.69
2:E:145:LEU:HD12	1:D:55:MET:HG2	1.73	0.69
1:G:55:MET:HG2	2:K:145:LEU:HD12	1.73	0.69
2:B:120:ILE:HB	2:B:143:THR:HG23	1.73	0.69
2:F:92:GLU:HG3	2:F:168:THR:CG2	2.22	0.69
2:E:120:ILE:HB	2:E:143:THR:HG23	1.73	0.69
2:K:120:ILE:HB	2:K:143:THR:HG23	1.73	0.69
2:K:98:ARG:HH21	2:K:164:VAL:HG21	1.55	0.69
2:H:92:GLU:HG3	2:H:168:THR:CG2	2.22	0.68
1:I:151:CYS:SG	1:I:169:ILE:HG23	2.34	0.68
1:C:151:CYS:SG	1:C:169:ILE:HG23	2.34	0.68
1:A:151:CYS:SG	1:A:169:ILE:HG23	2.34	0.68
1:J:151:CYS:SG	1:J:169:ILE:HG23	2.34	0.68
1:D:98:ARG:HB2	2:F:132:SER:HB3	1.76	0.68
1:J:86:TRP:CE3	1:J:145:LEU:HD21	2.29	0.68
1:A:86:TRP:CE3	1:A:145:LEU:HD21	2.29	0.68
1:A:56:LEU:HD11	1:A:62:MET:CE	2.24	0.67
1:G:56:LEU:HD11	1:G:62:MET:CE	2.24	0.67
1:C:86:TRP:CE3	1:C:145:LEU:HD21	2.29	0.67
1:J:56:LEU:HD11	1:J:62:MET:CE	2.24	0.67
1:D:56:LEU:HD11	1:D:62:MET:CE	2.24	0.67
1:I:53:ARG:HG3	2:L:143:THR:CB	2.24	0.67
1:I:86:TRP:CE3	1:I:145:LEU:HD21	2.30	0.67
1:A:111:ILE:HD11	1:A:165:LEU:CD2	2.25	0.67
1:A:98:ARG:HB2	2:B:132:SER:HB3	1.75	0.67
1:D:151:CYS:SG	1:D:169:ILE:HG23	2.34	0.67

Continued on next page...

PRELIMINARY VALIDATION REPORT

Continued from previous page...

Atom-1	Atom-2	Interatomic distance (Å)	Clash overlap (Å)
2:H:97:MET:HE3	1:J:55:MET:SD	2.33	0.67
2:E:143:THR:CB	1:D:53:ARG:HG3	2.24	0.67
1:G:53:ARG:HG3	2:K:143:THR:CB	2.24	0.67
2:B:143:THR:CB	1:C:53:ARG:HG3	2.24	0.67
1:C:111:ILE:HD11	1:C:165:LEU:CD2	2.25	0.67
1:I:111:ILE:HD11	1:I:165:LEU:CD2	2.25	0.67
1:J:111:ILE:HD11	1:J:165:LEU:CD2	2.25	0.67
1:D:111:ILE:HD11	1:D:165:LEU:CD2	2.25	0.67
1:G:111:ILE:HD11	1:G:165:LEU:CD2	2.25	0.67
1:D:86:TRP:CE3	1:D:145:LEU:HD21	2.29	0.67
1:A:55:MET:SD	2:F:97:MET:HE3	2.34	0.66
1:G:86:TRP:CE3	1:G:145:LEU:HD21	2.29	0.66
1:C:56:LEU:HD11	1:C:62:MET:CE	2.24	0.66
1:I:56:LEU:HD11	1:I:62:MET:CE	2.24	0.66
1:C:135:GLY:N	2:E:98:ARG:HD2	2.11	0.66
2:E:111:ILE:HD13	2:E:158:ALA:HB2	1.78	0.66
1:A:53:ARG:HG3	2:F:143:THR:CB	2.24	0.66
2:H:143:THR:CB	1:J:53:ARG:HG3	2.24	0.66
1:I:153:LYS:HE2	1:I:156:ILE:CG2	2.26	0.66
2:K:111:ILE:HD13	2:K:158:ALA:HB2	1.78	0.66
1:C:153:LYS:HE2	1:C:156:ILE:CG2	2.26	0.66
1:A:135:GLY:N	2:B:98:ARG:HD2	2.11	0.65
1:A:153:LYS:HE2	1:A:156:ILE:CG2	2.26	0.65
1:I:184:GLN:HA	2:L:106:LYS:CD	2.26	0.65
1:C:82:ILE:HD11	1:C:101:MET:HG3	1.79	0.65
1:D:96:LYS:HE2	2:F:131:ASP:C	2.17	0.65
2:B:106:LYS:CD	1:C:184:GLN:HA	2.26	0.65
1:D:82:ILE:HD11	1:D:101:MET:HG3	1.79	0.65
1:G:96:LYS:HE2	2:H:131:ASP:C	2.17	0.65
1:J:153:LYS:HE2	1:J:156:ILE:CG2	2.27	0.65
1:D:153:LYS:HE2	1:D:156:ILE:CG2	2.26	0.65
1:C:96:LYS:HE2	2:E:132:SER:CA	2.26	0.65
1:D:135:GLY:N	2:F:98:ARG:HD2	2.10	0.65
1:I:82:ILE:HD11	1:I:101:MET:HG3	1.79	0.65
1:I:96:LYS:HE2	2:K:132:SER:CA	2.26	0.65
2:E:106:LYS:CD	1:D:184:GLN:HA	2.27	0.65
1:G:153:LYS:HE2	1:G:156:ILE:CG2	2.26	0.65
1:J:135:GLY:N	2:L:98:ARG:HD2	2.11	0.65
1:G:184:GLN:HA	2:K:106:LYS:CD	2.26	0.65
1:G:82:ILE:HD11	1:G:101:MET:HG3	1.79	0.65
1:J:96:LYS:HE2	2:L:131:ASP:C	2.17	0.65

Continued on next page...

PRELIMINARY VALIDATION REPORT

Continued from previous page...

Atom-1	Atom-2	Interatomic distance (Å)	Clash overlap (Å)
1:A:96:LYS:HE2	2:B:131:ASP:C	2.17	0.65
1:D:98:ARG:HG3	2:F:132:SER:HB2	1.79	0.65
2:H:98:ARG:NH2	2:H:164:VAL:HG11	2.12	0.65
1:I:96:LYS:HE2	2:K:131:ASP:C	2.17	0.65
2:F:98:ARG:NH2	2:F:164:VAL:HG11	2.12	0.64
1:G:96:LYS:HE2	2:H:132:SER:CA	2.26	0.64
2:B:111:ILE:HD13	2:B:158:ALA:HB2	1.78	0.64
1:C:96:LYS:HE2	2:E:131:ASP:C	2.17	0.64
2:L:111:ILE:HD13	2:L:158:ALA:HB2	1.78	0.64
2:F:111:ILE:HD13	2:F:158:ALA:HB2	1.78	0.64
2:H:111:ILE:HD13	2:H:158:ALA:HB2	1.78	0.64
1:G:92:GLU:HB3	1:G:94:GLU:H	1.63	0.64
2:H:106:LYS:CD	1:J:184:GLN:HA	2.27	0.64
1:J:92:GLU:HB3	1:J:94:GLU:H	1.63	0.64
1:A:92:GLU:HB3	1:A:94:GLU:H	1.63	0.64
1:D:92:GLU:HB3	1:D:94:GLU:H	1.63	0.64
2:E:98:ARG:NH2	2:E:164:VAL:HG11	2.12	0.64
1:A:184:GLN:HA	2:F:106:LYS:CD	2.26	0.64
1:D:96:LYS:HE2	2:F:132:SER:CA	2.28	0.64
1:J:104:LEU:HD11	1:J:109:VAL:HB	1.80	0.64
1:J:95:ILE:HD12	1:J:167:ILE:HG23	1.79	0.63
1:A:104:LEU:HD11	1:A:109:VAL:HB	1.80	0.63
1:A:82:ILE:HD11	1:A:101:MET:HG3	1.79	0.63
1:A:95:ILE:HD12	1:A:167:ILE:HG23	1.79	0.63
1:I:92:GLU:CB	1:I:94:GLU:H	2.11	0.63
1:I:92:GLU:HB3	1:I:94:GLU:H	1.63	0.63
1:A:96:LYS:HE2	2:B:132:SER:CA	2.28	0.63
1:A:98:ARG:HG3	2:B:132:SER:HB2	1.80	0.63
1:C:92:GLU:HB3	1:C:94:GLU:H	1.63	0.63
1:G:95:ILE:HD12	1:G:167:ILE:HG23	1.79	0.63
1:J:82:ILE:HD11	1:J:101:MET:HG3	1.79	0.63
1:J:92:GLU:CB	1:J:94:GLU:H	2.12	0.63
1:D:81:GLU:CD	1:D:138:VAL:HG13	2.20	0.63
1:A:92:GLU:CB	1:A:94:GLU:H	2.12	0.62
2:B:97:MET:SD	1:C:55:MET:SD	2.96	0.62
1:C:95:ILE:HD12	1:C:167:ILE:HG23	1.79	0.62
1:D:95:ILE:HD12	1:D:167:ILE:HG23	1.79	0.62
1:D:92:GLU:CB	1:D:94:GLU:H	2.12	0.62
1:G:92:GLU:CB	1:G:94:GLU:H	2.12	0.62
1:A:55:MET:SD	2:F:95:ILE:CG2	2.87	0.62
1:C:92:GLU:CB	1:C:94:GLU:H	2.12	0.62

Continued on next page...

PRELIMINARY VALIDATION REPORT

Continued from previous page...

Atom-1	Atom-2	Interatomic distance (Å)	Clash overlap (Å)
1:D:104:LEU:HD11	1:D:109:VAL:HB	1.80	0.62
2:E:97:MET:SD	1:D:55:MET:SD	2.97	0.62
1:G:55:MET:SD	2:K:97:MET:SD	2.97	0.62
1:J:96:LYS:HE2	2:L:132:SER:CA	2.28	0.62
2:B:118:LEU:HD13	2:B:119:VAL:N	2.15	0.62
1:G:135:GLY:N	2:H:98:ARG:HD2	2.13	0.62
1:C:104:LEU:HD11	1:C:109:VAL:HB	1.80	0.62
1:D:95:ILE:HD11	1:D:169:ILE:CD1	2.29	0.62
2:L:118:LEU:HD13	2:L:119:VAL:N	2.15	0.62
1:A:95:ILE:HD11	1:A:169:ILE:CD1	2.29	0.62
1:I:95:ILE:HD12	1:I:167:ILE:HG23	1.79	0.62
1:J:95:ILE:HD11	1:J:169:ILE:CD1	2.29	0.62
1:G:95:ILE:HD11	1:G:169:ILE:CD1	2.29	0.62
1:I:104:LEU:HD11	1:I:109:VAL:HB	1.80	0.62
1:I:55:MET:SD	2:L:97:MET:SD	2.97	0.62
2:H:145:LEU:HD12	1:J:55:MET:CG	2.29	0.62
1:A:55:MET:CG	2:F:145:LEU:HD12	2.29	0.62
1:G:104:LEU:HD11	1:G:109:VAL:HB	1.81	0.62
1:G:81:GLU:CD	1:G:138:VAL:HG13	2.20	0.62
1:I:95:ILE:HD11	1:I:169:ILE:CD1	2.30	0.62
2:L:98:ARG:NH2	2:L:164:VAL:HG11	2.14	0.62
1:C:81:GLU:CD	1:C:138:VAL:HG13	2.20	0.62
2:H:95:ILE:CG2	1:J:55:MET:SD	2.88	0.62
1:I:81:GLU:CD	1:I:138:VAL:HG13	2.21	0.61
1:I:85:PRO:HG2	2:K:132:SER:HB3	1.82	0.61
1:I:135:GLY:N	2:K:98:ARG:HD2	2.15	0.61
2:K:118:LEU:HD13	2:K:119:VAL:N	2.15	0.61
1:C:95:ILE:HD11	1:C:169:ILE:CD1	2.30	0.61
2:E:118:LEU:HD13	2:E:119:VAL:N	2.15	0.61
1:G:55:MET:CG	2:K:145:LEU:HD12	2.31	0.61
1:C:135:GLY:HA2	2:E:98:ARG:NH1	2.16	0.61
2:E:145:LEU:HD12	1:D:55:MET:CG	2.31	0.61
2:B:145:LEU:HD12	1:C:55:MET:CG	2.30	0.61
2:E:112:SER:CB	1:D:179:ILE:HG22	2.31	0.61
1:A:95:ILE:H	1:A:168:THR:HA	1.65	0.61
1:G:179:ILE:HG22	2:K:112:SER:CB	2.31	0.61
1:I:55:MET:CG	2:L:145:LEU:HD12	2.31	0.61
1:A:81:GLU:CD	1:A:138:VAL:HG13	2.21	0.61
1:G:95:ILE:H	1:G:168:THR:HA	1.65	0.61
2:H:118:LEU:HD13	2:H:119:VAL:N	2.15	0.61
1:J:95:ILE:H	1:J:168:THR:HA	1.65	0.61

Continued on next page...

PRELIMINARY VALIDATION REPORT

Continued from previous page...

Atom-1	Atom-2	Interatomic distance (Å)	Clash overlap (Å)
2:B:167:ILE:HD13	2:B:168:THR:N	2.16	0.60
2:F:118:LEU:HD13	2:F:119:VAL:N	2.15	0.60
2:L:167:ILE:HD13	2:L:168:THR:N	2.16	0.60
1:D:95:ILE:H	1:D:168:THR:HA	1.65	0.60
1:J:81:GLU:CD	1:J:138:VAL:HG13	2.21	0.60
1:I:95:ILE:H	1:I:168:THR:HA	1.65	0.60
1:J:135:GLY:HA2	2:L:98:ARG:NH1	2.16	0.60
1:C:95:ILE:H	1:C:168:THR:HA	1.65	0.60
1:G:135:GLY:CA	2:H:98:ARG:HH11	2.14	0.60
2:B:94:GLU:HG3	2:B:147:LEU:HD22	1.83	0.60
2:B:92:GLU:HG3	2:B:168:THR:HG23	1.83	0.60
1:G:151:CYS:SG	1:G:169:ILE:HG23	2.42	0.60
2:K:98:ARG:NH1	2:K:164:VAL:HG11	2.17	0.60
2:L:94:GLU:HG3	2:L:147:LEU:HD22	1.83	0.60
1:A:179:ILE:HG22	2:F:112:SER:CB	2.31	0.60
2:B:112:SER:CB	1:C:179:ILE:HG22	2.31	0.60
2:H:112:SER:CB	1:J:179:ILE:HG22	2.31	0.60
2:K:167:ILE:HD13	2:K:168:THR:N	2.17	0.60
1:I:179:ILE:HG22	2:L:112:SER:CB	2.31	0.60
2:L:92:GLU:HG3	2:L:168:THR:HG23	1.84	0.60
2:E:167:ILE:HD13	2:E:168:THR:N	2.17	0.60
2:B:98:ARG:NH2	2:B:164:VAL:HG11	2.16	0.59
1:A:56:LEU:HD11	1:A:62:MET:HE3	1.83	0.59
1:A:99:PHE:HZ	1:A:109:VAL:HG21	1.66	0.59
1:D:135:GLY:CA	2:F:98:ARG:HH11	2.15	0.59
1:I:95:ILE:CD1	1:I:118:LEU:HD22	2.33	0.59
2:L:104:LEU:HD21	2:L:124:GLN:HG2	1.85	0.59
1:A:92:GLU:HA	1:A:171:LYS:HG3	1.84	0.59
1:C:95:ILE:CD1	1:C:118:LEU:HD22	2.33	0.59
1:D:99:PHE:CE2	1:D:165:LEU:HD22	2.38	0.59
1:J:92:GLU:HA	1:J:171:LYS:HG3	1.84	0.59
1:J:99:PHE:HZ	1:J:109:VAL:HG21	1.66	0.59
1:C:99:PHE:HZ	1:C:109:VAL:HG21	1.66	0.59
1:D:92:GLU:HA	1:D:171:LYS:HG3	1.84	0.59
2:F:167:ILE:HD13	2:F:168:THR:N	2.17	0.59
1:G:99:PHE:CE2	1:G:165:LEU:HD22	2.38	0.59
2:B:104:LEU:HD21	2:B:124:GLN:HG2	1.85	0.59
1:C:99:PHE:CE2	1:C:165:LEU:HD22	2.38	0.59
1:C:92:GLU:HA	1:C:171:LYS:HG3	1.84	0.59
1:G:92:GLU:HA	1:G:171:LYS:HG3	1.84	0.59
2:H:167:ILE:HD13	2:H:168:THR:N	2.17	0.59

Continued on next page...

PRELIMINARY VALIDATION REPORT

Continued from previous page...

Atom-1	Atom-2	Interatomic distance (Å)	Clash overlap (Å)
1:I:99:PHE:CE2	1:I:165:LEU:HD22	2.38	0.59
2:F:94:GLU:HG3	2:F:147:LEU:HD22	1.84	0.59
2:F:92:GLU:HG3	2:F:168:THR:HG23	1.83	0.59
1:I:92:GLU:HA	1:I:171:LYS:HG3	1.84	0.59
2:B:143:THR:HB	1:C:53:ARG:CG	2.32	0.59
2:E:92:GLU:HG3	2:E:168:THR:HG23	1.84	0.59
2:H:92:GLU:HG3	2:H:168:THR:HG23	1.83	0.59
1:I:99:PHE:HZ	1:I:109:VAL:HG21	1.66	0.59
1:A:55:MET:HE1	2:F:97:MET:HB2	1.85	0.59
1:D:95:ILE:CD1	1:D:118:LEU:HD22	2.33	0.59
1:G:124:GLN:HB2	1:G:138:VAL:HG11	1.85	0.59
1:D:86:TRP:CD2	1:D:145:LEU:HD21	2.38	0.58
1:G:86:TRP:CD2	1:G:145:LEU:HD21	2.38	0.58
1:G:95:ILE:CD1	1:G:118:LEU:HD22	2.33	0.58
2:H:94:GLU:HG3	2:H:147:LEU:HD22	1.85	0.58
2:H:97:MET:HB2	1:J:55:MET:HE1	1.85	0.58
1:I:53:ARG:CG	2:L:143:THR:HB	2.32	0.58
1:C:135:GLY:CA	2:E:98:ARG:HH11	2.16	0.58
1:D:135:GLY:HA2	2:F:98:ARG:NH1	2.18	0.58
2:K:94:GLU:HG3	2:K:147:LEU:HD22	1.83	0.58
1:A:156:ILE:HG12	1:A:167:ILE:HD11	1.85	0.58
1:A:95:ILE:CD1	1:A:118:LEU:HD22	2.32	0.58
1:D:124:GLN:HB2	1:D:138:VAL:HG11	1.85	0.58
1:J:156:ILE:HG12	1:J:167:ILE:HD11	1.85	0.58
1:I:86:TRP:CD2	1:I:145:LEU:HD21	2.38	0.58
1:J:99:PHE:CE2	1:J:165:LEU:HD22	2.38	0.58
1:A:99:PHE:CE2	1:A:165:LEU:HD22	2.38	0.58
1:C:86:TRP:CD2	1:C:145:LEU:HD21	2.39	0.58
2:E:94:GLU:HG3	2:E:147:LEU:HD22	1.83	0.58
2:K:92:GLU:HG3	2:K:168:THR:HG23	1.84	0.58
1:J:95:ILE:CD1	1:J:118:LEU:HD22	2.33	0.58
2:H:97:MET:SD	1:J:55:MET:SD	3.01	0.58
1:C:157:LYS:HB2	1:C:168:THR:HG22	1.86	0.58
1:D:81:GLU:OE1	1:D:138:VAL:HG13	2.04	0.58
1:A:55:MET:SD	2:F:95:ILE:HG22	2.44	0.58
1:D:156:ILE:HG12	1:D:167:ILE:HD11	1.85	0.58
2:E:143:THR:HB	1:D:53:ARG:CG	2.32	0.58
2:F:99:PHE:HE1	2:F:143:THR:HG21	1.67	0.58
1:G:156:ILE:HG12	1:G:167:ILE:HD11	1.85	0.58
1:G:53:ARG:CG	2:K:143:THR:HB	2.32	0.58
1:J:86:TRP:CD2	1:J:145:LEU:HD21	2.38	0.58

Continued on next page...

PRELIMINARY VALIDATION REPORT

Continued from previous page...

Atom-1	Atom-2	Interatomic distance (Å)	Clash overlap (Å)
1:A:86:TRP:CD2	1:A:145:LEU:HD21	2.38	0.58
2:B:120:ILE:HD12	2:B:165:LEU:CD2	2.34	0.58
1:C:101:MET:HG2	1:C:104:LEU:HB2	1.85	0.58
1:G:101:MET:HG2	1:G:104:LEU:HB2	1.85	0.58
1:I:101:MET:HG2	1:I:104:LEU:HB2	1.85	0.58
1:I:157:LYS:HB2	1:I:168:THR:HG22	1.86	0.58
2:L:120:ILE:HD12	2:L:165:LEU:CD2	2.34	0.58
1:I:55:MET:SD	2:L:95:ILE:CG2	2.92	0.58
1:D:101:MET:HG2	1:D:104:LEU:HB2	1.85	0.58
1:A:55:MET:SD	2:F:97:MET:SD	3.01	0.58
2:H:143:THR:HB	1:J:53:ARG:CG	2.32	0.58
1:I:98:ARG:HB2	2:K:132:SER:HB2	1.84	0.58
1:J:101:MET:HG2	1:J:104:LEU:HB2	1.86	0.58
1:A:53:ARG:CG	2:F:143:THR:HB	2.32	0.57
2:B:95:ILE:CG2	1:C:55:MET:SD	2.92	0.57
2:H:111:ILE:HD12	2:H:120:ILE:HD11	1.86	0.57
1:I:55:MET:SD	2:L:95:ILE:HG22	2.44	0.57
1:A:101:MET:HG2	1:A:104:LEU:HB2	1.86	0.57
1:G:55:MET:SD	2:K:95:ILE:HG22	2.44	0.57
2:H:104:LEU:HD21	2:H:124:GLN:HG2	1.85	0.57
1:G:55:MET:SD	2:K:95:ILE:CG2	2.92	0.57
2:B:95:ILE:HG22	1:C:55:MET:SD	2.44	0.57
2:E:104:LEU:HD21	2:E:124:GLN:HG2	1.85	0.57
1:G:183:ILE:HG23	2:K:107:GLU:OE2	2.05	0.57
2:B:118:LEU:HD11	2:B:120:ILE:HG12	1.86	0.57
2:E:107:GLU:OE2	1:D:183:ILE:HG23	2.05	0.57
2:E:116:ASN:HB3	1:D:177:LYS:HZ1	1.70	0.57
2:E:120:ILE:HD12	2:E:165:LEU:CD2	2.34	0.57
2:F:111:ILE:HD12	2:F:120:ILE:HD11	1.87	0.57
1:G:177:LYS:HZ1	2:K:116:ASN:HB3	1.70	0.57
1:G:81:GLU:OE1	1:G:138:VAL:HG13	2.05	0.57
2:H:99:PHE:HE1	2:H:143:THR:HG21	1.68	0.57
2:H:120:ILE:HD12	2:H:165:LEU:CD2	2.34	0.57
2:H:95:ILE:HG22	1:J:55:MET:SD	2.44	0.57
2:E:95:ILE:CG2	1:D:55:MET:SD	2.92	0.57
2:H:98:ARG:NH2	2:H:164:VAL:CG2	2.66	0.57
2:K:104:LEU:HD21	2:K:124:GLN:HG2	1.85	0.57
2:K:120:ILE:HD12	2:K:165:LEU:CD2	2.34	0.57
1:A:124:GLN:HB2	1:A:138:VAL:HG11	1.85	0.57
2:F:104:LEU:HD21	2:F:124:GLN:HG2	1.85	0.57
1:I:124:GLN:HB2	1:I:138:VAL:HG11	1.85	0.57

Continued on next page...

PRELIMINARY VALIDATION REPORT

Continued from previous page...

Atom-1	Atom-2	Interatomic distance (Å)	Clash overlap (Å)
1:J:124:GLN:HB2	1:J:138:VAL:HG11	1.85	0.57
2:L:118:LEU:HD11	2:L:120:ILE:HG12	1.86	0.57
1:C:124:GLN:HB2	1:C:138:VAL:HG11	1.85	0.57
1:D:157:LYS:HB2	1:D:168:THR:HG22	1.86	0.57
2:E:95:ILE:HG22	1:D:55:MET:SD	2.44	0.57
2:F:120:ILE:HD12	2:F:165:LEU:CD2	2.34	0.57
1:G:55:MET:SD	2:K:97:MET:CE	2.92	0.57
1:A:53:ARG:HH22	2:F:141:TYR:HB2	1.70	0.57
1:G:157:LYS:HB2	1:G:168:THR:HG22	1.86	0.57
2:H:141:TYR:HB2	1:J:53:ARG:HH22	1.70	0.57
1:I:156:ILE:HG12	1:I:167:ILE:HD11	1.86	0.57
1:J:98:ARG:HG3	2:L:132:SER:HB2	1.86	0.57
1:C:156:ILE:HG12	1:C:167:ILE:HD11	1.86	0.57
2:E:141:TYR:HB2	1:D:53:ARG:HH22	1.69	0.56
1:D:99:PHE:HZ	1:D:109:VAL:HG21	1.66	0.56
2:F:98:ARG:NH2	2:F:164:VAL:CG2	2.66	0.56
2:F:118:LEU:HD11	2:F:120:ILE:HG12	1.86	0.56
2:H:118:LEU:HD11	2:H:120:ILE:HG12	1.86	0.56
1:A:81:GLU:OE1	1:A:138:VAL:HG13	2.05	0.56
2:B:107:GLU:OE2	1:C:183:ILE:HG23	2.05	0.56
1:G:99:PHE:HZ	1:G:109:VAL:HG21	1.66	0.56
1:G:98:ARG:NH2	2:H:133:TRP:CZ2	2.73	0.56
1:J:157:LYS:HB2	1:J:168:THR:HG22	1.86	0.56
1:G:53:ARG:HH22	2:K:141:TYR:HB2	1.70	0.56
1:I:183:ILE:HG23	2:L:107:GLU:OE2	2.05	0.56
1:A:135:GLY:CA	2:B:98:ARG:HH11	2.19	0.56
1:A:157:LYS:HB2	1:A:168:THR:HG22	1.86	0.56
1:A:183:ILE:HG23	2:F:107:GLU:OE2	2.05	0.56
1:A:98:ARG:NH2	2:B:133:TRP:CZ2	2.73	0.56
1:D:98:ARG:NH2	2:F:133:TRP:CZ2	2.73	0.56
1:I:53:ARG:HH22	2:L:141:TYR:HB2	1.70	0.56
2:H:107:GLU:OE2	1:J:183:ILE:HG23	2.05	0.56
1:J:98:ARG:NH2	2:L:133:TRP:CZ2	2.73	0.56
2:K:111:ILE:HD12	2:K:120:ILE:HD11	1.87	0.56
2:L:99:PHE:HE1	2:L:143:THR:HG21	1.68	0.56
2:B:116:ASN:HB3	1:C:177:LYS:HZ1	1.70	0.56
2:B:99:PHE:HE1	2:B:143:THR:HG21	1.68	0.56
1:C:81:GLU:OE1	1:C:138:VAL:HG13	2.04	0.56
2:E:111:ILE:HD12	2:E:120:ILE:HD11	1.86	0.56
1:C:98:ARG:NH2	2:E:133:TRP:CZ2	2.74	0.56
1:J:81:GLU:OE1	1:J:138:VAL:HG13	2.05	0.56

Continued on next page...

PRELIMINARY VALIDATION REPORT

Continued from previous page...

Atom-1	Atom-2	Interatomic distance (Å)	Clash overlap (Å)
1:I:177:LYS:HZ1	2:L:116:ASN:HB3	1.70	0.56
1:A:95:ILE:HD11	1:A:169:ILE:HD12	1.88	0.56
2:B:141:TYR:HB2	1:C:53:ARG:HH22	1.70	0.56
2:E:156:ILE:HD12	2:E:169:ILE:HD11	1.88	0.56
1:I:81:GLU:OE1	1:I:138:VAL:HG13	2.05	0.56
1:I:98:ARG:NH2	2:K:133:TRP:CZ2	2.74	0.56
2:B:111:ILE:HD12	2:B:120:ILE:HD11	1.87	0.56
1:D:95:ILE:HD11	1:D:169:ILE:HD12	1.88	0.56
1:D:82:ILE:HD11	1:D:101:MET:CG	2.36	0.56
1:J:95:ILE:HD11	1:J:169:ILE:HD12	1.88	0.56
1:I:55:MET:SD	2:L:97:MET:CE	2.93	0.56
1:C:82:ILE:HD11	1:C:101:MET:CG	2.36	0.56
2:H:116:ASN:HB3	1:J:177:LYS:HZ1	1.70	0.56
2:K:156:ILE:HD12	2:K:169:ILE:HD11	1.88	0.56
2:L:111:ILE:HD12	2:L:120:ILE:HD11	1.87	0.56
1:A:177:LYS:HZ1	2:F:116:ASN:HB3	1.70	0.56
2:F:156:ILE:HD12	2:F:169:ILE:HD11	1.88	0.55
2:H:156:ILE:HD12	2:H:169:ILE:HD11	1.88	0.55
2:K:118:LEU:HD11	2:K:120:ILE:HG12	1.86	0.55
1:I:82:ILE:HD11	1:I:101:MET:CG	2.36	0.55
2:E:110:LYS:NZ	1:D:179:ILE:HD12	2.22	0.55
2:E:118:LEU:HD11	2:E:120:ILE:HG12	1.86	0.55
1:G:82:ILE:HD11	1:G:101:MET:CG	2.36	0.55
2:B:104:LEU:HD11	2:B:141:TYR:CE2	2.41	0.55
1:G:95:ILE:HD11	1:G:169:ILE:HD12	1.88	0.55
1:A:82:ILE:HD11	1:A:101:MET:CG	2.36	0.55
2:E:104:LEU:HD11	2:E:141:TYR:CE2	2.41	0.55
1:G:179:ILE:HD12	2:K:110:LYS:NZ	2.22	0.55
1:I:179:ILE:HD12	2:L:110:LYS:NZ	2.22	0.55
1:A:135:GLY:HA2	2:B:98:ARG:HH11	1.71	0.55
2:B:110:LYS:NZ	1:C:179:ILE:HD12	2.22	0.55
1:J:82:ILE:HD11	1:J:101:MET:CG	2.36	0.55
1:A:55:MET:N	2:F:145:LEU:CD1	2.70	0.55
2:H:145:LEU:CD1	1:J:55:MET:N	2.70	0.55
2:E:145:LEU:CD1	1:D:55:MET:N	2.70	0.55
1:G:55:MET:N	2:K:145:LEU:CD1	2.70	0.55
1:I:55:MET:N	2:L:145:LEU:CD1	2.70	0.55
2:L:156:ILE:HD12	2:L:169:ILE:HD11	1.88	0.55
2:B:145:LEU:CD1	1:C:55:MET:N	2.70	0.54
1:G:156:ILE:HD12	1:G:169:ILE:HG12	1.89	0.54
1:A:89:LYS:HD3	1:A:90:GLU:H	1.72	0.54

Continued on next page...

PRELIMINARY VALIDATION REPORT

Continued from previous page...

Atom-1	Atom-2	Interatomic distance (Å)	Clash overlap (Å)
2:B:110:LYS:HZ1	1:C:179:ILE:HG13	1.71	0.54
2:B:156:ILE:HD12	2:B:169:ILE:HD11	1.88	0.54
2:B:88:ILE:HG23	2:B:93:HIS:NE2	2.23	0.54
1:C:89:LYS:HD3	1:C:90:GLU:H	1.72	0.54
1:G:111:ILE:HD11	1:G:165:LEU:HD22	1.90	0.54
1:I:89:LYS:HD3	1:I:90:GLU:H	1.72	0.54
1:D:111:ILE:HD11	1:D:165:LEU:HD22	1.90	0.54
1:D:89:LYS:HD3	1:D:90:GLU:H	1.72	0.54
2:H:110:LYS:NZ	1:J:179:ILE:HD12	2.22	0.54
1:J:89:LYS:HD3	1:J:90:GLU:H	1.72	0.54
1:A:179:ILE:HD12	2:F:110:LYS:NZ	2.22	0.54
2:E:90:GLU:C	2:E:174:VAL:HG23	2.28	0.54
1:G:89:LYS:HD3	1:G:90:GLU:H	1.72	0.54
2:H:104:LEU:HD11	2:H:141:TYR:CE2	2.42	0.54
2:L:104:LEU:HD11	2:L:141:TYR:CE2	2.42	0.54
2:B:111:ILE:HD11	2:B:167:ILE:CG1	2.38	0.54
2:E:97:MET:CE	1:D:55:MET:SD	2.95	0.54
2:F:90:GLU:C	2:F:174:VAL:HG23	2.28	0.54
2:H:111:ILE:HD11	2:H:167:ILE:CG1	2.38	0.54
2:H:90:GLU:C	2:H:174:VAL:HG23	2.28	0.54
2:K:90:GLU:C	2:K:174:VAL:HG23	2.28	0.54
2:L:88:ILE:HG23	2:L:93:HIS:NE2	2.23	0.54
2:B:90:GLU:C	2:B:174:VAL:HG23	2.28	0.54
2:F:104:LEU:HD11	2:F:141:TYR:CE2	2.42	0.54
2:F:111:ILE:HD11	2:F:167:ILE:CG1	2.38	0.54
1:I:179:ILE:HG13	2:L:110:LYS:HZ1	1.71	0.54
2:H:110:LYS:HZ1	1:J:179:ILE:HG13	1.73	0.54
2:K:104:LEU:HD11	2:K:141:TYR:CE2	2.42	0.54
2:L:90:GLU:C	2:L:174:VAL:HG23	2.28	0.54
2:H:120:ILE:HD12	2:H:165:LEU:HD21	1.90	0.54
1:I:111:ILE:HD11	1:I:165:LEU:HD22	1.90	0.54
2:L:111:ILE:HD11	2:L:167:ILE:CG1	2.38	0.54
1:C:85:PRO:HG2	2:E:132:SER:HB2	1.89	0.54
1:I:95:ILE:HD11	1:I:169:ILE:HD12	1.88	0.54
2:K:99:PHE:HE1	2:K:143:THR:HG21	1.68	0.54
1:C:111:ILE:HD11	1:C:165:LEU:HD22	1.90	0.54
1:D:156:ILE:HD12	1:D:169:ILE:HG12	1.90	0.54
2:B:97:MET:CE	1:C:55:MET:SD	2.95	0.54
2:E:111:ILE:HD11	2:E:167:ILE:CG1	2.38	0.54
1:C:119:VAL:HG22	1:C:144:ARG:CD	2.38	0.53
1:D:56:LEU:HD11	1:D:62:MET:HE3	1.89	0.53

Continued on next page...

PRELIMINARY VALIDATION REPORT

Continued from previous page...

Atom-1	Atom-2	Interatomic distance (Å)	Clash overlap (Å)
2:F:120:ILE:HD12	2:F:165:LEU:HD21	1.90	0.53
1:G:179:ILE:HG13	2:K:110:LYS:HZ1	1.74	0.53
2:K:111:ILE:HD11	2:K:167:ILE:CG1	2.38	0.53
1:A:98:ARG:NH2	2:B:133:TRP:CE2	2.77	0.53
2:E:99:PHE:HE1	2:E:143:THR:HG21	1.68	0.53
1:A:179:ILE:HG13	2:F:110:LYS:HZ1	1.73	0.53
1:G:119:VAL:HG22	1:G:144:ARG:CD	2.38	0.53
1:I:119:VAL:HG22	1:I:144:ARG:CD	2.38	0.53
1:J:98:ARG:NH2	2:L:133:TRP:CE2	2.77	0.53
1:C:43:LEU:HD12	1:C:145:LEU:CD1	2.38	0.53
1:D:119:VAL:HG22	1:D:144:ARG:CD	2.38	0.53
1:D:42:LEU:N	1:D:42:LEU:HD12	2.24	0.53
1:G:42:LEU:HD12	1:G:42:LEU:N	2.24	0.53
1:I:43:LEU:HD12	1:I:145:LEU:CD1	2.38	0.53
1:A:119:VAL:HG22	1:A:144:ARG:CD	2.38	0.53
1:G:98:ARG:NH2	2:H:133:TRP:CE2	2.77	0.53
1:A:43:LEU:HD12	1:A:145:LEU:CD1	2.38	0.53
1:D:98:ARG:NH2	2:F:133:TRP:CE2	2.77	0.53
1:I:42:LEU:HD12	1:I:42:LEU:N	2.24	0.53
2:K:88:ILE:HG23	2:K:93:HIS:NE2	2.23	0.53
2:L:116:ASN:HB2	2:L:153:LYS:HE3	1.91	0.53
2:L:98:ARG:NH2	2:L:164:VAL:CG2	2.66	0.53
2:B:116:ASN:HB2	2:B:153:LYS:HE3	1.91	0.53
1:C:98:ARG:NH2	2:E:133:TRP:CE2	2.77	0.53
1:J:119:VAL:HG22	1:J:144:ARG:CD	2.39	0.53
2:L:120:ILE:HD12	2:L:165:LEU:HD21	1.90	0.53
1:A:111:ILE:HD11	1:A:165:LEU:HD22	1.90	0.53
1:C:42:LEU:HD12	1:C:42:LEU:N	2.24	0.53
2:E:143:THR:CA	1:D:53:ARG:HG3	2.39	0.53
1:J:43:LEU:HD12	1:J:145:LEU:CD1	2.39	0.53
1:I:98:ARG:NH2	2:K:133:TRP:CE2	2.77	0.53
2:E:88:ILE:HG23	2:E:93:HIS:NE2	2.23	0.53
1:G:53:ARG:NH1	2:K:141:TYR:HB2	2.23	0.53
2:H:88:ILE:HG23	2:H:93:HIS:NE2	2.23	0.53
1:G:53:ARG:HG3	2:K:143:THR:CA	2.39	0.53
2:B:120:ILE:HD12	2:B:165:LEU:HD21	1.90	0.53
1:A:53:ARG:HG3	2:F:143:THR:CA	2.39	0.53
2:F:88:ILE:HG23	2:F:93:HIS:NE2	2.23	0.53
1:G:135:GLY:HA2	2:H:98:ARG:HH11	1.74	0.53
1:J:42:LEU:HD12	1:J:42:LEU:N	2.24	0.53
2:H:143:THR:CA	1:J:53:ARG:HG3	2.39	0.53

Continued on next page...

PRELIMINARY VALIDATION REPORT

Continued from previous page...

Atom-1	Atom-2	Interatomic distance (Å)	Clash overlap (Å)
1:A:156:ILE:HD12	1:A:169:ILE:HG12	1.90	0.53
1:A:53:ARG:HH12	2:F:141:TYR:CB	2.22	0.53
2:B:98:ARG:NH2	2:B:164:VAL:CG2	2.67	0.53
1:I:53:ARG:HG3	2:L:143:THR:CA	2.39	0.53
1:J:156:ILE:HD12	1:J:169:ILE:HG12	1.90	0.53
1:A:42:LEU:HD12	1:A:42:LEU:N	2.24	0.52
2:B:143:THR:CA	1:C:53:ARG:HG3	2.39	0.52
1:J:111:ILE:HD11	1:J:165:LEU:HD22	1.90	0.52
2:H:141:TYR:CB	1:J:53:ARG:HH12	2.22	0.52
2:E:110:LYS:HZ1	1:D:179:ILE:HG13	1.75	0.52
2:E:95:ILE:HG12	2:E:167:ILE:HD12	1.91	0.52
1:D:43:LEU:HD12	1:D:145:LEU:CD1	2.38	0.52
2:E:120:ILE:HD12	2:E:165:LEU:HD21	1.90	0.52
1:G:43:LEU:HD12	1:G:145:LEU:CD1	2.39	0.52
1:G:53:ARG:HH12	2:K:141:TYR:CB	2.22	0.52
1:G:45:PRO:HG3	1:G:86:TRP:CZ3	2.45	0.52
1:J:76:GLY:HA2	1:J:79:VAL:HG22	1.92	0.52
1:J:136:ARG:HH21	2:L:83:ARG:HD2	1.75	0.52
1:A:136:ARG:HH21	2:B:83:ARG:HD2	1.75	0.52
1:C:95:ILE:HD11	1:C:169:ILE:HD12	1.89	0.52
1:D:45:PRO:HG3	1:D:86:TRP:CZ3	2.45	0.52
1:J:42:LEU:HD23	1:J:117:VAL:HG11	1.92	0.52
1:A:42:LEU:HD23	1:A:117:VAL:HG11	1.92	0.52
2:B:95:ILE:HG12	2:B:167:ILE:HD12	1.92	0.52
2:E:141:TYR:CB	1:D:53:ARG:HH12	2.23	0.52
2:H:116:ASN:HB2	2:H:153:LYS:HE3	1.91	0.52
2:K:98:ARG:NH2	2:K:164:VAL:CG2	2.65	0.52
1:A:76:GLY:HA2	1:A:79:VAL:HG22	1.92	0.52
2:E:141:TYR:HB2	1:D:53:ARG:NH1	2.24	0.52
2:F:116:ASN:HB2	2:F:153:LYS:HE3	1.91	0.52
1:G:56:LEU:HD11	1:G:62:MET:HE3	1.90	0.52
2:H:94:GLU:HB3	2:H:169:ILE:HB	1.90	0.52
1:C:56:LEU:HD11	1:C:62:MET:HE3	1.90	0.52
1:J:135:GLY:CA	2:L:98:ARG:HH11	2.20	0.52
2:K:120:ILE:HD12	2:K:165:LEU:HD21	1.91	0.52
2:K:95:ILE:HG12	2:K:167:ILE:HD12	1.92	0.52
2:L:95:ILE:HG12	2:L:167:ILE:HD12	1.92	0.52
1:C:76:GLY:HA2	1:C:79:VAL:HG22	1.92	0.52
1:C:45:PRO:HG3	1:C:86:TRP:CZ3	2.45	0.52
1:I:156:ILE:HD12	1:I:169:ILE:HG12	1.90	0.52
1:I:56:LEU:HD11	1:I:62:MET:HE3	1.90	0.52

Continued on next page...

PRELIMINARY VALIDATION REPORT

Continued from previous page...

Atom-1	Atom-2	Interatomic distance (Å)	Clash overlap (Å)
1:C:156:ILE:HD12	1:C:169:ILE:HG12	1.90	0.52
1:I:45:PRO:HG3	1:I:86:TRP:CZ3	2.45	0.52
2:K:116:ASN:HB2	2:K:153:LYS:HE3	1.91	0.52
1:I:53:ARG:HH12	2:L:141:TYR:CB	2.22	0.51
1:I:76:GLY:HA2	1:I:79:VAL:HG22	1.92	0.51
1:I:53:ARG:NH1	2:L:141:TYR:HB2	2.24	0.51
2:E:116:ASN:HB2	2:E:153:LYS:HE3	1.91	0.51
2:F:94:GLU:HB3	2:F:169:ILE:HB	1.91	0.51
1:A:92:GLU:HB3	1:A:94:GLU:N	2.25	0.51
1:G:42:LEU:HD23	1:G:117:VAL:HG11	1.92	0.51
1:J:92:GLU:HB3	1:J:94:GLU:N	2.25	0.51
1:D:92:GLU:HB3	1:D:94:GLU:N	2.25	0.51
1:G:92:GLU:HB3	1:G:94:GLU:N	2.25	0.51
1:A:45:PRO:HG3	1:A:86:TRP:CZ3	2.45	0.51
1:D:42:LEU:HD23	1:D:117:VAL:HG11	1.92	0.51
1:J:45:PRO:HG3	1:J:86:TRP:CZ3	2.45	0.51
2:K:120:ILE:HB	2:K:143:THR:CG2	2.41	0.51
2:B:141:TYR:CB	1:C:53:ARG:HH12	2.23	0.51
1:C:136:ARG:HH21	2:E:83:ARG:HD2	1.75	0.51
2:E:120:ILE:HB	2:E:143:THR:CG2	2.41	0.51
2:H:95:ILE:HG12	2:H:167:ILE:HD12	1.93	0.51
2:E:98:ARG:NH2	2:E:164:VAL:CG2	2.66	0.51
2:B:110:LYS:HZ1	1:C:179:ILE:CG1	2.24	0.51
2:F:95:ILE:HG12	2:F:167:ILE:HD12	1.93	0.51
1:G:136:ARG:HH21	2:H:83:ARG:HD2	1.74	0.51
1:D:136:ARG:HH21	2:F:83:ARG:HD2	1.75	0.51
1:G:76:GLY:HA2	1:G:79:VAL:HG22	1.92	0.51
1:I:42:LEU:HD23	1:I:117:VAL:HG11	1.92	0.50
1:C:92:GLU:HB3	1:C:94:GLU:N	2.25	0.50
1:I:92:GLU:HB3	1:I:94:GLU:N	2.25	0.50
1:C:42:LEU:HD23	1:C:117:VAL:HG11	1.92	0.50
1:C:92:GLU:CG	1:C:170:PRO:HA	2.42	0.50
2:E:93:HIS:CD2	2:E:171:LYS:HD2	2.47	0.50
2:K:94:GLU:HB3	2:K:169:ILE:HB	1.93	0.50
2:K:93:HIS:CD2	2:K:171:LYS:HD2	2.47	0.50
2:L:94:GLU:HB3	2:L:169:ILE:HB	1.93	0.50
1:D:76:GLY:HA2	1:D:79:VAL:HG22	1.92	0.50
1:G:147:LEU:HD11	1:G:169:ILE:HD13	1.93	0.50
1:I:147:LEU:HD11	1:I:169:ILE:HD13	1.93	0.50
1:I:179:ILE:CG1	2:L:110:LYS:HZ1	2.24	0.50
2:L:93:HIS:CD2	2:L:171:LYS:HD2	2.47	0.50

Continued on next page...

PRELIMINARY VALIDATION REPORT

Continued from previous page...

Atom-1	Atom-2	Interatomic distance (Å)	Clash overlap (Å)
2:B:141:TYR:HB2	1:C:53:ARG:NH1	2.25	0.50
2:B:94:GLU:HB3	2:B:169:ILE:HB	1.93	0.50
2:E:94:GLU:HB3	2:E:169:ILE:HB	1.93	0.50
1:I:92:GLU:CG	1:I:170:PRO:HA	2.42	0.50
1:I:89:LYS:CD	1:I:90:GLU:H	2.25	0.50
1:C:89:LYS:CD	1:C:90:GLU:H	2.25	0.50
1:J:89:LYS:CD	1:J:90:GLU:H	2.25	0.50
1:A:89:LYS:CD	1:A:90:GLU:H	2.25	0.50
2:B:93:HIS:CD2	2:B:171:LYS:HD2	2.47	0.50
1:J:147:LEU:HD11	1:J:169:ILE:HD13	1.93	0.50
1:A:147:LEU:HD11	1:A:169:ILE:HD13	1.93	0.50
1:A:134:SER:C	2:B:98:ARG:CZ	2.80	0.50
1:D:89:LYS:CD	1:D:90:GLU:H	2.24	0.50
1:D:95:ILE:N	1:D:168:THR:HA	2.27	0.50
2:F:97:MET:HB3	2:F:167:ILE:CG2	2.42	0.50
1:G:89:LYS:CD	1:G:90:GLU:H	2.24	0.50
1:D:87:ASP:HB2	1:D:96:LYS:HB3	1.94	0.50
2:E:95:ILE:HD13	2:E:169:ILE:CG1	2.42	0.50
1:G:95:ILE:N	1:G:168:THR:HA	2.27	0.50
2:B:95:ILE:HD13	2:B:169:ILE:CG1	2.42	0.49
1:D:147:LEU:HD11	1:D:169:ILE:HD13	1.93	0.49
2:F:93:HIS:CD2	2:F:171:LYS:HD2	2.47	0.49
2:H:97:MET:HB3	2:H:167:ILE:CG2	2.42	0.49
2:H:93:HIS:CD2	2:H:171:LYS:HD2	2.47	0.49
2:K:95:ILE:HD13	2:K:169:ILE:CG1	2.42	0.49
2:B:88:ILE:HG23	2:B:93:HIS:CD2	2.47	0.49
1:G:135:GLY:CA	2:H:98:ARG:NH1	2.75	0.49
1:J:95:ILE:N	1:J:168:THR:HA	2.27	0.49
1:A:135:GLY:CA	2:B:98:ARG:NH1	2.75	0.49
1:A:95:ILE:N	1:A:168:THR:HA	2.27	0.49
2:F:94:GLU:HG2	2:F:147:LEU:HA	1.94	0.49
1:J:56:LEU:HD11	1:J:62:MET:HE3	1.93	0.49
2:L:120:ILE:HB	2:L:143:THR:CG2	2.41	0.49
2:L:95:ILE:HD13	2:L:169:ILE:CG1	2.42	0.49
2:E:97:MET:HB3	2:E:167:ILE:CG2	2.43	0.49
1:G:87:ASP:HB2	1:G:96:LYS:HB3	1.94	0.49
2:H:141:TYR:HB2	1:J:53:ARG:NH1	2.23	0.49
2:K:97:MET:HB3	2:K:167:ILE:CG2	2.43	0.49
2:L:88:ILE:HG23	2:L:93:HIS:CD2	2.48	0.49
2:E:99:PHE:CE2	2:E:120:ILE:HG13	2.48	0.49
1:A:184:GLN:HB2	2:F:106:LYS:N	2.27	0.49

Continued on next page...

PRELIMINARY VALIDATION REPORT

Continued from previous page...

Atom-1	Atom-2	Interatomic distance (Å)	Clash overlap (Å)
2:B:120:ILE:HB	2:B:143:THR:CG2	2.41	0.49
1:A:92:GLU:CG	1:A:170:PRO:HA	2.41	0.49
2:E:113:VAL:O	1:D:177:LYS:HB2	2.13	0.49
1:A:177:LYS:HB2	2:F:113:VAL:O	2.13	0.49
1:A:53:ARG:NH1	2:F:141:TYR:HB2	2.23	0.49
2:H:113:VAL:O	1:J:177:LYS:HB2	2.13	0.49
2:H:94:GLU:HG2	2:H:147:LEU:HA	1.95	0.49
1:G:134:SER:C	2:H:98:ARG:CZ	2.80	0.49
1:G:135:GLY:HA3	2:H:98:ARG:HH11	1.77	0.49
2:H:106:LYS:N	1:J:184:GLN:HB2	2.27	0.49
1:G:92:GLU:CG	1:G:170:PRO:HA	2.42	0.49
1:I:133:TRP:HB3	1:I:135:GLY:H	1.77	0.49
2:K:99:PHE:CE2	2:K:120:ILE:HG13	2.48	0.49
1:C:96:LYS:CE	2:E:131:ASP:C	2.81	0.49
1:I:95:ILE:N	1:I:168:THR:HA	2.27	0.49
2:E:106:LYS:H	1:D:184:GLN:HB2	1.78	0.48
2:E:106:LYS:N	1:D:184:GLN:HB2	2.27	0.48
2:E:88:ILE:HG23	2:E:93:HIS:CD2	2.47	0.48
1:A:184:GLN:HB2	2:F:106:LYS:H	1.78	0.48
1:J:134:SER:C	2:L:98:ARG:CZ	2.82	0.48
1:G:184:GLN:HB2	2:K:106:LYS:N	2.27	0.48
1:G:177:LYS:HB2	2:K:113:VAL:O	2.13	0.48
2:K:88:ILE:HG23	2:K:93:HIS:CD2	2.47	0.48
1:C:135:GLY:CA	2:E:98:ARG:NH1	2.75	0.48
2:B:106:LYS:H	1:C:184:GLN:HB2	1.78	0.48
1:C:95:ILE:N	1:C:168:THR:HA	2.27	0.48
1:D:92:GLU:CG	1:D:170:PRO:HA	2.42	0.48
1:G:85:PRO:HG2	2:H:132:SER:HB3	1.95	0.48
1:I:96:LYS:CE	2:K:131:ASP:C	2.81	0.48
1:J:92:GLU:CG	1:J:170:PRO:HA	2.42	0.48
2:B:99:PHE:CE2	2:B:120:ILE:HG13	2.48	0.48
2:H:106:LYS:H	1:J:184:GLN:HB2	1.79	0.48
2:L:97:MET:HB3	2:L:167:ILE:CG2	2.43	0.48
2:B:113:VAL:O	1:C:177:LYS:HB2	2.13	0.48
2:B:97:MET:HB3	2:B:167:ILE:CG2	2.43	0.48
2:B:106:LYS:N	1:C:184:GLN:HB2	2.27	0.48
2:H:88:ILE:HG23	2:H:93:HIS:CD2	2.48	0.48
1:I:177:LYS:HB2	2:L:113:VAL:O	2.13	0.48
1:I:184:GLN:HB2	2:L:106:LYS:N	2.27	0.48
1:A:53:ARG:HB3	2:F:143:THR:HA	1.96	0.48
1:A:87:ASP:HB2	1:A:96:LYS:HB3	1.94	0.48

Continued on next page...

PRELIMINARY VALIDATION REPORT

Continued from previous page...

Atom-1	Atom-2	Interatomic distance (Å)	Clash overlap (Å)
1:C:99:PHE:CE1	1:C:101:MET:HB2	2.49	0.48
1:G:53:ARG:HB3	2:K:143:THR:HA	1.96	0.48
1:I:99:PHE:CE1	1:I:101:MET:HB2	2.49	0.48
2:H:143:THR:HA	1:J:53:ARG:HB3	1.96	0.48
2:L:99:PHE:CE2	2:L:120:ILE:HG13	2.48	0.48
1:D:96:LYS:CE	2:F:131:ASP:C	2.82	0.48
2:E:143:THR:HA	1:D:53:ARG:HB3	1.96	0.48
2:F:88:ILE:HG23	2:F:93:HIS:CD2	2.48	0.48
2:F:99:PHE:CE2	2:F:120:ILE:HG13	2.47	0.48
1:G:184:GLN:HB2	2:K:106:LYS:H	1.79	0.48
1:G:96:LYS:CE	2:H:131:ASP:C	2.82	0.48
1:J:87:ASP:HB2	1:J:96:LYS:HB3	1.94	0.48
1:I:184:GLN:HB2	2:L:106:LYS:H	1.79	0.48
2:H:99:PHE:CE2	2:H:120:ILE:HG13	2.47	0.48
1:I:87:ASP:HB2	1:I:96:LYS:HB3	1.94	0.48
1:C:87:ASP:HB2	1:C:96:LYS:HB3	1.94	0.48
2:E:94:GLU:HG2	2:E:147:LEU:HA	1.95	0.48
1:G:135:GLY:HA2	2:H:98:ARG:CD	2.44	0.48
1:I:134:SER:HA	2:K:98:ARG:HD2	1.95	0.48
2:K:94:GLU:HG2	2:K:147:LEU:HA	1.95	0.48
2:B:94:GLU:HG2	2:B:147:LEU:HA	1.95	0.47
1:C:147:LEU:HD11	1:C:169:ILE:HD13	1.95	0.47
1:G:99:PHE:CE1	1:G:101:MET:HB2	2.49	0.47
2:H:120:ILE:HB	2:H:143:THR:CG2	2.41	0.47
1:I:136:ARG:HH21	2:K:83:ARG:HD2	1.77	0.47
2:L:94:GLU:HG2	2:L:147:LEU:HA	1.95	0.47
1:A:43:LEU:HD12	1:A:145:LEU:HD11	1.97	0.47
1:A:96:LYS:HE3	2:B:130:ASP:O	2.14	0.47
2:B:143:THR:HA	1:C:53:ARG:HB3	1.96	0.47
1:C:43:LEU:HD12	1:C:145:LEU:HD11	1.97	0.47
1:I:53:ARG:HB3	2:L:143:THR:HA	1.96	0.47
1:J:99:PHE:CE1	1:J:101:MET:HB2	2.49	0.47
1:A:99:PHE:CE1	1:A:101:MET:HB2	2.49	0.47
1:A:99:PHE:CZ	1:A:165:LEU:HB2	2.50	0.47
1:D:134:SER:C	2:F:98:ARG:CZ	2.82	0.47
1:D:99:PHE:CE1	1:D:101:MET:HB2	2.49	0.47
1:I:96:LYS:HE3	2:K:130:ASP:O	2.14	0.47
1:J:135:GLY:CA	2:L:98:ARG:NH1	2.76	0.47
1:J:43:LEU:HD12	1:J:145:LEU:HD11	1.97	0.47
2:K:104:LEU:HD21	2:K:124:GLN:HA	1.97	0.47
1:A:96:LYS:CE	2:B:131:ASP:C	2.81	0.47

Continued on next page...

PRELIMINARY VALIDATION REPORT

Continued from previous page...

Atom-1	Atom-2	Interatomic distance (Å)	Clash overlap (Å)
2:F:120:ILE:HB	2:F:143:THR:CG2	2.41	0.47
2:F:95:ILE:HD13	2:F:169:ILE:CG1	2.44	0.47
1:I:134:SER:HA	2:K:98:ARG:CD	2.44	0.47
1:I:43:LEU:HD12	1:I:145:LEU:HD11	1.97	0.47
1:J:99:PHE:CZ	1:J:165:LEU:HB2	2.50	0.47
1:J:96:LYS:HE3	2:L:130:ASP:O	2.14	0.47
1:C:134:SER:C	2:E:98:ARG:CZ	2.82	0.47
2:H:95:ILE:HD13	2:H:169:ILE:CG1	2.44	0.47
1:I:134:SER:C	2:K:98:ARG:CZ	2.83	0.47
1:G:55:MET:HE3	2:K:86:TRP:HA	1.96	0.47
2:L:111:ILE:HD11	2:L:167:ILE:HG12	1.96	0.47
1:G:55:MET:HA	2:K:97:MET:HE3	1.96	0.47
1:I:99:PHE:CZ	1:I:165:LEU:HB2	2.49	0.47
2:B:111:ILE:HD11	2:B:167:ILE:HG12	1.97	0.47
1:C:96:LYS:HE3	2:E:130:ASP:O	2.15	0.47
1:C:99:PHE:CZ	1:C:165:LEU:HB2	2.49	0.47
2:E:111:ILE:HD11	2:E:167:ILE:HG12	1.97	0.47
1:G:96:LYS:HE3	2:H:130:ASP:O	2.14	0.47
2:K:111:ILE:HD11	2:K:167:ILE:HG12	1.97	0.47
2:L:104:LEU:HD21	2:L:124:GLN:HA	1.97	0.47
1:G:43:LEU:HD12	1:G:145:LEU:HD11	1.97	0.47
1:J:96:LYS:CE	2:L:131:ASP:C	2.82	0.47
1:D:43:LEU:HD12	1:D:145:LEU:HD11	1.97	0.47
1:D:96:LYS:HE3	2:F:130:ASP:O	2.14	0.47
1:D:99:PHE:CZ	1:D:165:LEU:HB2	2.49	0.47
1:G:99:PHE:CZ	1:G:165:LEU:HB2	2.49	0.47
2:H:97:MET:CE	1:J:55:MET:SD	3.02	0.47
1:C:158:ALA:HB2	1:C:167:ILE:CD1	2.46	0.46
2:E:104:LEU:HD21	2:E:124:GLN:HA	1.97	0.46
1:I:158:ALA:HB2	1:I:167:ILE:CD1	2.46	0.46
1:A:86:TRP:CD1	1:A:95:ILE:CG2	2.99	0.46
1:A:135:GLY:HA2	2:B:98:ARG:CD	2.45	0.46
1:C:82:ILE:CG2	1:C:99:PHE:HB2	2.46	0.46
1:J:96:LYS:C	1:J:96:LYS:HD3	2.36	0.46
1:A:96:LYS:C	1:A:96:LYS:HD3	2.36	0.46
1:C:96:LYS:HD3	1:C:96:LYS:C	2.34	0.46
1:J:98:ARG:HD2	1:J:164:VAL:HG11	1.98	0.46
1:J:86:TRP:CD1	1:J:95:ILE:CG2	2.99	0.46
1:I:86:TRP:CD1	1:I:95:ILE:CG2	2.99	0.46
1:D:158:ALA:HB2	1:D:167:ILE:CD1	2.45	0.46
1:D:135:GLY:CA	2:F:98:ARG:NH1	2.75	0.46

Continued on next page...

PRELIMINARY VALIDATION REPORT

Continued from previous page...

Atom-1	Atom-2	Interatomic distance (Å)	Clash overlap (Å)
1:I:55:MET:HE3	2:L:86:TRP:HA	1.96	0.46
1:I:82:ILE:CG2	1:I:99:PHE:HB2	2.46	0.46
2:B:104:LEU:HD21	2:B:124:GLN:HA	1.98	0.46
1:C:86:TRP:CD1	1:C:95:ILE:CG2	2.99	0.46
1:G:158:ALA:HB2	1:G:167:ILE:CD1	2.46	0.46
2:H:111:ILE:HD11	2:H:167:ILE:HG12	1.96	0.46
1:A:158:ALA:HB2	1:A:167:ILE:CD1	2.46	0.46
1:A:98:ARG:HD2	1:A:164:VAL:HG11	1.98	0.46
1:A:82:ILE:CG2	1:A:99:PHE:HB2	2.46	0.46
1:C:98:ARG:HG3	2:E:132:SER:H	1.80	0.46
1:J:158:ALA:HB2	1:J:167:ILE:CD1	2.46	0.46
1:J:82:ILE:CG2	1:J:99:PHE:HB2	2.46	0.46
1:A:55:MET:SD	2:F:97:MET:CE	3.03	0.46
2:F:104:LEU:HD21	2:F:124:GLN:HA	1.97	0.46
2:F:111:ILE:HD11	2:F:167:ILE:HG12	1.97	0.46
2:H:110:LYS:HZ1	1:J:179:ILE:CG1	2.28	0.46
1:J:85:PRO:HG3	2:L:133:TRP:C	2.36	0.46
2:H:104:LEU:HD21	2:H:124:GLN:HA	1.97	0.46
1:J:95:ILE:HD12	1:J:167:ILE:CG2	2.46	0.46
1:A:135:GLY:HA2	2:B:98:ARG:NH1	2.31	0.46
1:A:95:ILE:HD12	1:A:167:ILE:CG2	2.46	0.46
1:G:96:LYS:HD3	1:G:96:LYS:C	2.35	0.46
1:D:86:TRP:CD1	1:D:95:ILE:HG23	2.52	0.45
1:I:96:LYS:HD3	1:I:96:LYS:C	2.35	0.45
2:K:161:LYS:O	2:K:164:VAL:HG22	2.15	0.45
1:A:134:SER:C	2:B:98:ARG:HD2	2.36	0.45
1:D:96:LYS:C	1:D:96:LYS:HD3	2.36	0.45
1:D:82:ILE:CG2	1:D:99:PHE:HB2	2.46	0.45
1:G:82:ILE:CG2	1:G:99:PHE:HB2	2.46	0.45
2:K:114:GLU:CD	2:K:119:VAL:HG21	2.37	0.45
1:I:147:LEU:CD1	1:I:169:ILE:HD13	2.47	0.45
1:A:85:PRO:HG3	2:B:133:TRP:C	2.36	0.45
1:C:157:LYS:HB2	1:C:168:THR:CG2	2.46	0.45
1:C:147:LEU:CD1	1:C:169:ILE:HD13	2.47	0.45
1:D:147:LEU:CD1	1:D:169:ILE:HD13	2.47	0.45
1:G:147:LEU:CD1	1:G:169:ILE:HD13	2.46	0.45
1:G:86:TRP:CD1	1:G:95:ILE:HG23	2.52	0.45
1:I:157:LYS:HB2	1:I:168:THR:CG2	2.46	0.45
1:A:134:SER:HA	2:B:98:ARG:HD2	1.98	0.45
2:B:119:VAL:HA	2:B:143:THR:O	2.17	0.45
2:B:110:LYS:HD3	1:C:181:VAL:HG22	1.99	0.45

Continued on next page...

PRELIMINARY VALIDATION REPORT

Continued from previous page...

Atom-1	Atom-2	Interatomic distance (Å)	Clash overlap (Å)
1:D:85:PRO:HG3	2:F:133:TRP:C	2.36	0.45
1:D:86:TRP:CD1	1:D:95:ILE:CG2	2.99	0.45
2:E:114:GLU:CD	2:E:119:VAL:HG21	2.37	0.45
1:G:157:LYS:HB2	1:G:168:THR:CG2	2.46	0.45
1:G:86:TRP:CD1	1:G:95:ILE:CG2	2.99	0.45
1:D:119:VAL:HG22	1:D:144:ARG:HD2	1.98	0.45
1:D:134:SER:C	2:F:98:ARG:HD2	2.37	0.45
1:G:119:VAL:HG22	1:G:144:ARG:HD2	1.98	0.45
2:L:161:LYS:O	2:L:164:VAL:HG22	2.16	0.45
1:A:179:ILE:CG1	2:F:110:LYS:HZ1	2.29	0.45
1:I:95:ILE:HD12	1:I:167:ILE:CG2	2.46	0.45
1:I:181:VAL:HG22	2:L:110:LYS:HD3	1.99	0.45
2:L:119:VAL:HA	2:L:143:THR:O	2.17	0.45
2:L:114:GLU:CD	2:L:119:VAL:HG21	2.37	0.45
1:A:147:LEU:CD1	1:A:169:ILE:HD13	2.47	0.45
1:D:98:ARG:HD2	1:D:164:VAL:HG11	1.98	0.45
2:H:119:VAL:HA	2:H:143:THR:O	2.17	0.45
1:G:134:SER:C	2:H:98:ARG:HD2	2.37	0.45
1:J:147:LEU:CD1	1:J:169:ILE:HD13	2.47	0.45
1:J:86:TRP:CD1	1:J:95:ILE:HG23	2.51	0.45
1:C:95:ILE:HD11	1:C:169:ILE:HD11	1.99	0.45
1:D:157:LYS:HB2	1:D:168:THR:CG2	2.46	0.45
1:I:119:VAL:HG22	1:I:144:ARG:HD2	1.98	0.45
2:L:114:GLU:OE1	2:L:119:VAL:HG21	2.17	0.45
2:B:86:TRP:HA	1:C:55:MET:HE3	1.99	0.45
1:C:177:LYS:H	1:C:177:LYS:HD2	1.82	0.45
1:D:177:LYS:HD2	1:D:177:LYS:H	1.82	0.45
2:F:119:VAL:HA	2:F:143:THR:O	2.17	0.45
2:F:114:GLU:OE1	2:F:119:VAL:HG21	2.17	0.45
1:G:98:ARG:HD2	1:G:164:VAL:HG11	1.99	0.45
1:A:157:LYS:HB2	1:A:168:THR:CG2	2.46	0.44
2:B:114:GLU:CD	2:B:119:VAL:HG21	2.37	0.44
1:C:119:VAL:HG22	1:C:144:ARG:HD2	1.98	0.44
2:E:86:TRP:HA	1:D:55:MET:HE3	1.99	0.44
2:H:114:GLU:OE1	2:H:119:VAL:HG21	2.17	0.44
1:I:177:LYS:H	1:I:177:LYS:HD2	1.82	0.44
1:I:85:PRO:HG2	2:K:132:SER:CB	2.47	0.44
1:I:98:ARG:HG3	2:K:132:SER:H	1.82	0.44
1:A:119:VAL:HG22	1:A:144:ARG:HD2	1.98	0.44
1:A:86:TRP:CD1	1:A:95:ILE:HG23	2.52	0.44
2:B:114:GLU:OE1	2:B:119:VAL:HG21	2.17	0.44

Continued on next page...

PRELIMINARY VALIDATION REPORT

Continued from previous page...

Atom-1	Atom-2	Interatomic distance (Å)	Clash overlap (Å)
2:E:110:LYS:HD3	1:D:181:VAL:HG22	1.98	0.44
2:H:114:GLU:CD	2:H:119:VAL:HG21	2.37	0.44
1:J:157:LYS:HB2	1:J:168:THR:CG2	2.46	0.44
1:J:177:LYS:H	1:J:177:LYS:HD2	1.82	0.44
1:C:119:VAL:HG22	1:C:144:ARG:HD3	1.99	0.44
1:G:55:MET:CE	2:K:86:TRP:HA	2.48	0.44
1:I:46:LEU:H	1:I:46:LEU:HD22	1.83	0.44
1:G:181:VAL:HG22	2:K:110:LYS:HD3	1.99	0.44
1:A:177:LYS:HD2	1:A:177:LYS:H	1.82	0.44
2:F:114:GLU:CD	2:F:119:VAL:HG21	2.37	0.44
1:G:177:LYS:HD2	1:G:177:LYS:H	1.82	0.44
1:G:98:ARG:CG	2:H:132:SER:HB2	2.45	0.44
2:H:110:LYS:HD3	1:J:181:VAL:HG22	1.99	0.44
2:K:98:ARG:NH2	2:K:164:VAL:HG11	2.32	0.44
2:L:93:HIS:HA	2:L:96:LYS:HB2	2.00	0.44
1:C:92:GLU:C	1:C:94:GLU:H	2.21	0.44
2:E:114:GLU:OE1	2:E:119:VAL:HG21	2.17	0.44
2:E:161:LYS:O	2:E:164:VAL:HG22	2.17	0.44
1:I:82:ILE:HG13	1:I:141:TYR:CZ	2.53	0.44
2:K:119:VAL:HA	2:K:143:THR:O	2.17	0.44
1:A:43:LEU:CD2	1:A:50:ARG:HE	2.31	0.44
1:C:82:ILE:HG13	1:C:141:TYR:CZ	2.53	0.44
1:C:46:LEU:H	1:C:46:LEU:HD22	1.83	0.44
2:E:119:VAL:HA	2:E:143:THR:O	2.17	0.44
1:A:181:VAL:HG22	2:F:110:LYS:HD3	1.99	0.44
1:G:179:ILE:CG1	2:K:110:LYS:HZ1	2.30	0.44
1:J:119:VAL:HG22	1:J:144:ARG:HD2	1.98	0.44
1:J:56:LEU:HA	1:J:56:LEU:HD22	1.86	0.44
1:I:184:GLN:CA	2:L:106:LYS:HD2	2.46	0.44
2:B:93:HIS:HA	2:B:96:LYS:HB2	2.00	0.44
1:C:43:LEU:CD2	1:C:50:ARG:HE	2.31	0.44
2:E:111:ILE:HD11	2:E:167:ILE:HG13	2.00	0.44
2:H:161:LYS:O	2:H:164:VAL:HG22	2.16	0.44
1:I:92:GLU:C	1:I:94:GLU:H	2.21	0.44
1:J:43:LEU:CD2	1:J:50:ARG:HE	2.31	0.44
2:K:114:GLU:OE1	2:K:119:VAL:HG21	2.17	0.44
1:I:134:SER:HA	2:K:98:ARG:NE	2.33	0.44
1:A:49:MET:HA	1:A:62:MET:HE1	1.99	0.44
1:A:56:LEU:HD22	1:A:56:LEU:HA	1.86	0.44
1:C:86:TRP:CD1	1:C:95:ILE:HG23	2.52	0.44
1:D:46:LEU:H	1:D:46:LEU:HD22	1.83	0.44

Continued on next page...

PRELIMINARY VALIDATION REPORT

Continued from previous page...

Atom-1	Atom-2	Interatomic distance (Å)	Clash overlap (Å)
1:G:43:LEU:CD2	1:G:50:ARG:HE	2.31	0.44
1:I:119:VAL:HG22	1:I:144:ARG:HD3	2.00	0.44
1:I:43:LEU:CD2	1:I:50:ARG:HE	2.31	0.44
1:I:86:TRP:CD1	1:I:95:ILE:HG23	2.52	0.44
1:I:95:ILE:HD11	1:I:169:ILE:HD11	2.00	0.44
2:B:106:LYS:HD2	1:C:184:GLN:CA	2.46	0.44
1:G:46:LEU:H	1:G:46:LEU:HD22	1.83	0.44
2:H:93:HIS:HA	2:H:96:LYS:HB2	2.00	0.44
2:K:111:ILE:HD11	2:K:167:ILE:HG13	2.00	0.44
1:A:82:ILE:HG13	1:A:141:TYR:CZ	2.52	0.43
1:A:133:TRP:HB3	1:A:135:GLY:H	1.83	0.43
1:D:92:GLU:C	1:D:94:GLU:H	2.21	0.43
2:E:95:ILE:HD13	2:E:169:ILE:HG13	2.00	0.43
2:F:93:HIS:HA	2:F:96:LYS:HB2	2.00	0.43
1:J:82:ILE:HG13	1:J:141:TYR:CZ	2.52	0.43
1:J:92:GLU:C	1:J:94:GLU:H	2.21	0.43
2:K:95:ILE:HD13	2:K:169:ILE:HG13	2.00	0.43
2:B:161:LYS:O	2:B:164:VAL:HG22	2.18	0.43
2:B:111:ILE:HD11	2:B:167:ILE:HG13	2.00	0.43
1:D:43:LEU:CD2	1:D:50:ARG:HE	2.31	0.43
2:F:161:LYS:O	2:F:164:VAL:HG22	2.17	0.43
1:G:82:ILE:HG13	1:G:141:TYR:CZ	2.53	0.43
2:L:118:LEU:CD1	2:L:120:ILE:HG12	2.48	0.43
2:B:118:LEU:CD1	2:B:120:ILE:HG12	2.48	0.43
1:A:104:LEU:HD13	1:A:105:SER:O	2.19	0.43
1:A:119:VAL:HG22	1:A:144:ARG:HD3	2.00	0.43
1:A:158:ALA:HB2	1:A:167:ILE:HD13	2.00	0.43
1:A:46:LEU:H	1:A:46:LEU:HD22	1.83	0.43
1:A:82:ILE:HG21	1:A:99:PHE:HB2	2.00	0.43
2:B:97:MET:HB2	1:C:55:MET:HE1	2.00	0.43
1:C:85:PRO:HG2	2:E:132:SER:CB	2.47	0.43
1:D:104:LEU:HD13	1:D:105:SER:O	2.19	0.43
1:D:82:ILE:HG13	1:D:141:TYR:CZ	2.53	0.43
2:E:86:TRP:HA	1:D:55:MET:CE	2.49	0.43
2:E:97:MET:HB2	1:D:55:MET:HE1	2.00	0.43
1:G:92:GLU:C	1:G:94:GLU:H	2.21	0.43
2:H:97:MET:HE3	1:J:55:MET:CE	2.48	0.43
1:J:119:VAL:HG22	1:J:144:ARG:HD3	2.00	0.43
1:A:92:GLU:C	1:A:94:GLU:H	2.21	0.43
1:C:95:ILE:C	1:C:97:MET:H	2.21	0.43
1:D:110:LYS:HB2	1:D:121:LYS:HD3	2.01	0.43

Continued on next page...

PRELIMINARY VALIDATION REPORT

Continued from previous page...

Atom-1	Atom-2	Interatomic distance (Å)	Clash overlap (Å)
1:G:104:LEU:HD13	1:G:105:SER:O	2.19	0.43
1:I:111:ILE:HD11	1:I:165:LEU:HD21	2.01	0.43
1:I:98:ARG:HD2	1:I:164:VAL:HG11	2.00	0.43
1:J:158:ALA:HB2	1:J:167:ILE:HD13	2.00	0.43
1:J:46:LEU:HD22	1:J:46:LEU:H	1.83	0.43
1:C:111:ILE:HD11	1:C:165:LEU:HD21	2.01	0.43
2:B:86:TRP:HA	1:C:55:MET:CE	2.48	0.43
2:E:110:LYS:HZ1	1:D:179:ILE:CG1	2.31	0.43
1:D:85:PRO:HG3	2:F:134:SER:N	2.34	0.43
1:A:55:MET:CB	2:F:145:LEU:HD12	2.49	0.43
1:G:110:LYS:HB2	1:G:121:LYS:HD3	2.01	0.43
1:G:85:PRO:HG3	2:H:133:TRP:C	2.38	0.43
2:H:118:LEU:HD13	2:H:119:VAL:C	2.39	0.43
1:G:135:GLY:HA2	2:H:98:ARG:HD3	2.00	0.43
1:J:104:LEU:HD13	1:J:105:SER:O	2.19	0.43
1:J:82:ILE:HG21	1:J:99:PHE:HB2	2.00	0.43
1:I:55:MET:CE	2:L:86:TRP:HA	2.48	0.43
1:D:153:LYS:HE2	1:D:156:ILE:HG22	2.01	0.43
1:G:153:LYS:HE2	1:G:156:ILE:HG22	2.01	0.43
2:H:145:LEU:HD12	1:J:55:MET:CB	2.49	0.43
2:F:118:LEU:HD13	2:F:119:VAL:C	2.39	0.43
1:G:158:ALA:HB2	1:G:167:ILE:HD13	2.00	0.43
2:H:86:TRP:HA	1:J:55:MET:CE	2.49	0.43
1:J:95:ILE:C	1:J:97:MET:H	2.23	0.43
1:A:95:ILE:C	1:A:97:MET:H	2.23	0.43
1:C:111:ILE:HG21	1:C:167:ILE:HD13	2.01	0.43
1:D:158:ALA:HB2	1:D:167:ILE:HD13	2.00	0.43
1:D:82:ILE:HG21	1:D:99:PHE:HB2	2.00	0.43
1:C:134:SER:C	2:E:98:ARG:HD2	2.39	0.43
2:F:111:ILE:HD11	2:F:167:ILE:HG13	2.00	0.43
1:G:82:ILE:HG21	1:G:99:PHE:HB2	2.00	0.43
1:I:104:LEU:HD13	1:I:105:SER:O	2.19	0.43
1:J:85:PRO:HG3	2:L:134:SER:N	2.34	0.43
1:C:104:LEU:HD13	1:C:105:SER:O	2.19	0.42
1:D:132:SER:O	1:D:133:TRP:C	2.57	0.42
1:D:95:ILE:HD12	1:D:167:ILE:CG2	2.46	0.42
1:G:92:GLU:HG2	1:G:170:PRO:HA	2.01	0.42
1:G:95:ILE:HD12	1:G:167:ILE:CG2	2.46	0.42
2:H:95:ILE:HD13	2:H:169:ILE:HG13	2.01	0.42
1:G:95:ILE:HD11	1:G:169:ILE:HD11	2.00	0.42
2:H:111:ILE:HD11	2:H:167:ILE:HG13	2.00	0.42

Continued on next page...

PRELIMINARY VALIDATION REPORT

Continued from previous page...

Atom-1	Atom-2	Interatomic distance (Å)	Clash overlap (Å)
1:I:111:ILE:HG21	1:I:167:ILE:HD13	2.02	0.42
1:I:95:ILE:C	1:I:97:MET:H	2.22	0.42
2:L:111:ILE:HD11	2:L:167:ILE:HG13	2.00	0.42
1:A:92:GLU:HG2	1:A:170:PRO:HA	2.01	0.42
1:A:55:MET:CE	2:F:86:TRP:HA	2.49	0.42
1:A:85:PRO:HG3	2:B:134:SER:N	2.34	0.42
1:C:156:ILE:HD11	1:C:167:ILE:CG1	2.49	0.42
1:D:119:VAL:HG22	1:D:144:ARG:HD3	2.00	0.42
1:D:95:ILE:C	1:D:97:MET:H	2.22	0.42
1:G:156:ILE:HA	1:G:168:THR:O	2.20	0.42
1:J:156:ILE:HD11	1:J:167:ILE:CG1	2.49	0.42
1:I:96:LYS:NZ	2:K:132:SER:HA	2.33	0.42
1:A:156:ILE:HD11	1:A:167:ILE:CG1	2.49	0.42
1:C:82:ILE:HG21	1:C:99:PHE:HB2	2.00	0.42
1:D:156:ILE:HA	1:D:168:THR:O	2.20	0.42
2:E:141:TYR:HB2	1:D:53:ARG:NH2	2.34	0.42
2:E:93:HIS:HA	2:E:96:LYS:HB2	2.00	0.42
2:F:95:ILE:HD13	2:F:169:ILE:HG13	2.01	0.42
1:G:132:SER:O	1:G:133:TRP:C	2.58	0.42
1:G:119:VAL:HG22	1:G:144:ARG:HD3	2.00	0.42
1:G:95:ILE:C	1:G:97:MET:H	2.23	0.42
2:H:118:LEU:CD1	2:H:120:ILE:HG12	2.48	0.42
1:I:156:ILE:HD11	1:I:167:ILE:CG1	2.49	0.42
2:K:93:HIS:HA	2:K:96:LYS:HB2	2.00	0.42
1:C:110:LYS:HB2	1:C:121:LYS:HD3	2.01	0.42
1:D:92:GLU:HG2	1:D:170:PRO:HA	2.02	0.42
1:I:110:LYS:HB2	1:I:121:LYS:HD3	2.01	0.42
2:L:118:LEU:HD13	2:L:119:VAL:C	2.39	0.42
2:B:118:LEU:HD13	2:B:119:VAL:C	2.39	0.42
1:C:98:ARG:HD2	1:C:164:VAL:HG11	2.01	0.42
1:D:156:ILE:HD11	1:D:167:ILE:CG1	2.49	0.42
2:E:145:LEU:HD12	1:D:55:MET:CB	2.50	0.42
1:G:55:MET:HE3	2:K:86:TRP:CA	2.50	0.42
1:G:85:PRO:HG3	2:H:134:SER:N	2.35	0.42
1:J:132:SER:O	1:J:133:TRP:C	2.57	0.42
1:J:134:SER:C	2:L:98:ARG:HD2	2.39	0.42
1:J:92:GLU:HG2	1:J:170:PRO:HA	2.02	0.42
1:G:53:ARG:NH2	2:K:141:TYR:HB2	2.34	0.42
1:C:132:SER:O	1:C:133:TRP:C	2.57	0.42
2:F:118:LEU:CD1	2:F:120:ILE:HG12	2.48	0.42
1:A:55:MET:CE	2:F:97:MET:HE3	2.49	0.42

Continued on next page...

PRELIMINARY VALIDATION REPORT

Continued from previous page...

Atom-1	Atom-2	Interatomic distance (Å)	Clash overlap (Å)
1:I:92:GLU:HG2	1:I:170:PRO:HA	2.02	0.42
1:I:82:ILE:HG21	1:I:99:PHE:HB2	2.00	0.42
1:A:156:ILE:HA	1:A:168:THR:O	2.20	0.42
1:C:92:GLU:HG2	1:C:170:PRO:HA	2.02	0.42
2:E:118:LEU:HD13	2:E:119:VAL:C	2.39	0.42
1:I:158:ALA:HB2	1:I:167:ILE:HD13	2.00	0.42
1:G:55:MET:CB	2:K:145:LEU:HD12	2.50	0.42
1:A:48:PRO:HA	1:A:54:GLN:NE2	2.35	0.42
1:C:98:ARG:HA	1:C:165:LEU:O	2.20	0.42
1:D:95:ILE:HD11	1:D:169:ILE:HD11	2.00	0.42
2:E:92:GLU:HG3	2:E:168:THR:HG22	2.00	0.42
1:G:156:ILE:HD11	1:G:167:ILE:CG1	2.50	0.42
1:J:156:ILE:HA	1:J:168:THR:O	2.20	0.42
1:I:55:MET:HE3	2:L:86:TRP:CA	2.50	0.42
1:A:110:LYS:HB2	1:A:121:LYS:HD3	2.01	0.42
1:D:48:PRO:HA	1:D:54:GLN:NE2	2.35	0.42
1:D:95:ILE:C	1:D:97:MET:N	2.73	0.42
1:A:53:ARG:NH2	2:F:141:TYR:HB2	2.34	0.42
2:H:141:TYR:HB2	1:J:53:ARG:NH2	2.34	0.42
1:J:110:LYS:HB2	1:J:121:LYS:HD3	2.01	0.42
1:J:48:PRO:HA	1:J:54:GLN:NE2	2.35	0.42
2:K:118:LEU:HD13	2:K:119:VAL:C	2.39	0.42
1:A:111:ILE:HG21	1:A:167:ILE:HD13	2.02	0.41
2:B:95:ILE:HD13	2:B:169:ILE:HG12	2.02	0.41
1:A:135:GLY:HA2	2:B:98:ARG:HD3	2.02	0.41
1:C:48:PRO:HA	1:C:54:GLN:NE2	2.35	0.41
1:C:95:ILE:HD12	1:C:167:ILE:CG2	2.46	0.41
2:E:117:VAL:HG21	1:D:46:LEU:HD12	2.02	0.41
1:G:48:PRO:HA	1:G:54:GLN:NE2	2.35	0.41
1:I:48:PRO:HA	1:I:54:GLN:NE2	2.35	0.41
1:J:111:ILE:HG21	1:J:167:ILE:HD13	2.02	0.41
1:J:95:ILE:C	1:J:97:MET:N	2.74	0.41
1:G:46:LEU:HD12	2:K:117:VAL:HG21	2.02	0.41
1:G:55:MET:CG	2:K:95:ILE:HG22	2.50	0.41
1:I:55:MET:CG	2:L:95:ILE:HG22	2.50	0.41
1:A:95:ILE:C	1:A:97:MET:N	2.74	0.41
2:B:146:GLN:HE21	1:C:46:LEU:HB3	1.85	0.41
2:B:95:ILE:HD13	2:B:169:ILE:HG13	2.00	0.41
1:C:158:ALA:HB2	1:C:167:ILE:HD13	2.00	0.41
2:E:95:ILE:HG22	1:D:55:MET:CG	2.50	0.41
2:F:92:GLU:HG3	2:F:168:THR:HG22	2.00	0.41

Continued on next page...

PRELIMINARY VALIDATION REPORT

Continued from previous page...

Atom-1	Atom-2	Interatomic distance (Å)	Clash overlap (Å)
1:G:95:ILE:C	1:G:97:MET:N	2.73	0.41
2:K:84:ALA:C	2:K:97:MET:HE1	2.41	0.41
2:B:86:TRP:CB	1:C:55:MET:HE3	2.50	0.41
2:B:95:ILE:HG22	1:C:55:MET:CG	2.50	0.41
2:E:86:TRP:CA	1:D:55:MET:HE3	2.51	0.41
2:E:95:ILE:HD13	2:E:169:ILE:HG12	2.02	0.41
1:D:98:ARG:NH2	2:F:131:ASP:HB3	2.35	0.41
2:H:143:THR:HA	1:J:53:ARG:O	2.21	0.41
1:I:46:LEU:HB3	2:L:146:GLN:HE21	1.85	0.41
2:L:95:ILE:HD13	2:L:169:ILE:HG12	2.02	0.41
2:L:95:ILE:HD13	2:L:169:ILE:HG13	2.00	0.41
1:A:180:ASP:O	2:F:110:LYS:HE3	2.21	0.41
1:A:53:ARG:O	2:F:143:THR:HA	2.21	0.41
2:B:141:TYR:HB2	1:C:53:ARG:NH2	2.35	0.41
2:B:90:GLU:HB3	2:B:176:ARG:HB2	2.02	0.41
1:G:111:ILE:HG21	1:G:167:ILE:HD13	2.02	0.41
1:G:98:ARG:NH2	2:H:131:ASP:HB3	2.35	0.41
1:I:153:LYS:HE2	1:I:156:ILE:HG22	2.01	0.41
2:H:110:LYS:HE3	1:J:180:ASP:O	2.21	0.41
2:K:95:ILE:HD13	2:K:169:ILE:HG12	2.02	0.41
1:J:98:ARG:NH2	2:L:131:ASP:HB3	2.35	0.41
2:B:145:LEU:HD12	1:C:55:MET:CB	2.50	0.41
1:C:95:ILE:C	1:C:97:MET:N	2.73	0.41
1:D:111:ILE:HG21	1:D:167:ILE:HD13	2.02	0.41
1:D:49:MET:HA	1:D:62:MET:HE1	2.03	0.41
1:A:46:LEU:HB3	2:F:146:GLN:HE21	1.85	0.41
1:G:98:ARG:HB2	2:H:132:SER:CB	2.51	0.41
1:G:184:GLN:CA	2:K:106:LYS:HD2	2.45	0.41
2:K:92:GLU:HG3	2:K:168:THR:HG22	2.00	0.41
1:I:53:ARG:NH2	2:L:141:TYR:HB2	2.34	0.41
2:L:90:GLU:HB3	2:L:176:ARG:HB2	2.02	0.41
1:A:153:LYS:HE2	1:A:156:ILE:HG22	2.01	0.41
2:B:117:VAL:HG21	1:C:46:LEU:HD12	2.02	0.41
1:A:98:ARG:NH2	2:B:131:ASP:HB3	2.35	0.41
1:C:153:LYS:HE2	1:C:156:ILE:HG22	2.01	0.41
2:F:97:MET:SD	2:F:99:PHE:CZ	3.13	0.41
1:G:180:ASP:O	2:K:110:LYS:HE3	2.21	0.41
2:H:106:LYS:HD2	1:J:184:GLN:CA	2.46	0.41
1:I:95:ILE:C	1:I:97:MET:N	2.73	0.41
1:J:153:LYS:HE2	1:J:156:ILE:HG22	2.01	0.41
2:K:118:LEU:CD1	2:K:120:ILE:HG12	2.48	0.41

Continued on next page...

PRELIMINARY VALIDATION REPORT

Continued from previous page...

Atom-1	Atom-2	Interatomic distance (Å)	Clash overlap (Å)
1:G:53:ARG:O	2:K:143:THR:HA	2.21	0.41
1:I:46:LEU:HD12	2:L:117:VAL:HG21	2.02	0.41
1:J:96:LYS:HE2	2:L:132:SER:CB	2.51	0.41
1:C:156:ILE:HA	1:C:168:THR:O	2.20	0.41
1:I:156:ILE:HA	1:I:168:THR:O	2.19	0.41
1:I:98:ARG:HA	1:I:165:LEU:O	2.21	0.41
1:A:132:SER:O	1:A:133:TRP:C	2.59	0.41
2:E:106:LYS:HD2	1:D:184:GLN:CA	2.46	0.41
2:E:143:THR:HA	1:D:53:ARG:O	2.21	0.41
1:A:184:GLN:CA	2:F:106:LYS:HD2	2.46	0.41
1:G:134:SER:HA	2:H:98:ARG:HD2	2.02	0.41
2:H:146:GLN:HE21	1:J:46:LEU:HB3	1.85	0.41
2:H:92:GLU:HG3	2:H:168:THR:HG22	2.00	0.41
1:I:56:LEU:HD22	1:I:56:LEU:HA	1.87	0.41
1:I:55:MET:CB	2:L:145:LEU:HD12	2.50	0.41
2:B:86:TRP:CA	1:C:55:MET:HE3	2.50	0.41
1:C:56:LEU:HD22	1:C:56:LEU:HA	1.87	0.41
2:E:110:LYS:HE3	1:D:180:ASP:O	2.21	0.41
2:E:146:GLN:HE21	1:D:46:LEU:HB3	1.85	0.41
1:G:46:LEU:HB3	2:K:146:GLN:HE21	1.85	0.41
2:K:98:ARG:CZ	2:K:164:VAL:CG1	2.96	0.41
2:L:155:LYS:HB3	2:L:170:PRO:HG2	2.03	0.41
2:B:155:LYS:HB3	2:B:170:PRO:HG2	2.03	0.41
2:E:104:LEU:HD21	2:E:124:GLN:CG	2.51	0.41
2:H:95:ILE:HG22	1:J:55:MET:CG	2.51	0.41
2:H:97:MET:SD	2:H:99:PHE:CZ	3.14	0.41
1:J:56:LEU:HD13	1:J:56:LEU:C	2.41	0.41
1:I:85:PRO:HG3	2:K:133:TRP:C	2.42	0.41
2:L:104:LEU:CD2	2:L:124:GLN:HA	2.51	0.41
1:A:55:MET:CG	2:F:95:ILE:HG22	2.51	0.41
1:A:56:LEU:C	1:A:56:LEU:HD13	2.42	0.41
2:B:95:ILE:HG21	2:B:145:LEU:CB	2.51	0.41
1:D:111:ILE:HD11	1:D:165:LEU:HD21	2.01	0.41
1:C:98:ARG:NH2	2:E:131:ASP:HB3	2.35	0.41
1:G:98:ARG:HA	1:G:165:LEU:O	2.21	0.41
1:G:55:MET:HE1	2:K:97:MET:HB2	2.02	0.41
2:L:95:ILE:HG21	2:L:145:LEU:CB	2.51	0.41
2:E:86:TRP:CB	1:D:55:MET:HE3	2.50	0.40
2:F:90:GLU:HB3	2:F:176:ARG:HB2	2.02	0.40
2:H:90:GLU:HB3	2:H:176:ARG:HB2	2.02	0.40
2:K:104:LEU:HD21	2:K:124:GLN:CG	2.51	0.40

Continued on next page...

PRELIMINARY VALIDATION REPORT

Continued from previous page...

Atom-1	Atom-2	Interatomic distance (Å)	Clash overlap (Å)
1:I:98:ARG:NH2	2:K:131:ASP:HB3	2.35	0.40
1:I:85:PRO:HG3	2:K:134:SER:N	2.36	0.40
2:F:104:LEU:CD2	2:F:124:GLN:HA	2.51	0.40
2:H:104:LEU:CD2	2:H:124:GLN:HA	2.51	0.40
1:A:95:ILE:HD11	1:A:169:ILE:HD11	2.00	0.40
2:B:104:LEU:CD2	2:B:124:GLN:HA	2.52	0.40
2:B:143:THR:HA	1:C:53:ARG:O	2.21	0.40
1:C:99:PHE:CE2	1:C:109:VAL:HG21	2.56	0.40
1:C:82:ILE:HG21	1:C:99:PHE:CG	2.56	0.40
2:E:118:LEU:CD1	2:E:120:ILE:HG12	2.48	0.40
2:F:104:LEU:HD21	2:F:124:GLN:CG	2.51	0.40
1:G:49:MET:HA	1:G:62:MET:HE1	2.04	0.40
1:I:180:ASP:O	2:L:110:LYS:HE3	2.21	0.40
1:I:55:MET:N	2:L:145:LEU:HD12	2.37	0.40
1:I:82:ILE:HG21	1:I:99:PHE:CG	2.57	0.40
1:A:82:ILE:HG21	1:A:99:PHE:CG	2.56	0.40
2:B:117:VAL:HG13	2:B:145:LEU:O	2.22	0.40
2:E:106:LYS:HD3	1:D:184:GLN:HA	2.04	0.40
2:E:95:ILE:HG21	2:E:145:LEU:CB	2.51	0.40
2:F:160:LEU:HD23	2:F:160:LEU:C	2.42	0.40
2:F:155:LYS:HB3	2:F:170:PRO:HG2	2.03	0.40
1:G:111:ILE:HD11	1:G:165:LEU:HD21	2.01	0.40
2:H:104:LEU:HD21	2:H:124:GLN:CG	2.51	0.40
1:I:99:PHE:CE2	1:I:109:VAL:HG21	2.56	0.40
1:J:101:MET:SD	1:J:104:LEU:HG	2.62	0.40
1:J:82:ILE:HG21	1:J:99:PHE:CG	2.56	0.40
2:L:117:VAL:HG13	2:L:145:LEU:O	2.22	0.40
1:A:101:MET:SD	1:A:104:LEU:HG	2.62	0.40
1:C:98:ARG:CZ	1:C:164:VAL:HG11	2.51	0.40
2:B:145:LEU:HD12	1:C:55:MET:N	2.37	0.40
1:D:160:LEU:HD12	1:D:164:VAL:C	2.42	0.40
1:D:56:LEU:HD13	1:D:56:LEU:C	2.42	0.40
1:D:98:ARG:HA	1:D:165:LEU:O	2.22	0.40
1:G:135:GLY:HA2	2:H:98:ARG:NH1	2.35	0.40
1:G:133:TRP:HB3	1:G:135:GLY:H	1.86	0.40
1:G:160:LEU:HD12	1:G:164:VAL:C	2.42	0.40
1:J:96:LYS:HE2	2:L:132:SER:HB2	2.02	0.40
2:K:95:ILE:HG21	2:K:145:LEU:CB	2.51	0.40
1:I:53:ARG:O	2:L:143:THR:HA	2.21	0.40

There are no symmetry-related clashes.

PRELIMINARY VALIDATION REPORT

5.3 Torsion angles (i)

5.3.1 Protein backbone (i)

In the following table, the Percentiles column shows the percent Ramachandran outliers of the chain as a percentile score with respect to all PDB entries followed by that with respect to all EM entries.

The Analysed column shows the number of residues for which the backbone conformation was analysed, and the total number of residues.

Mol	Chain	Analysed	Favoured	Allowed	Outliers	Percentiles	
1	A	141/143 (99%)	131 (93%)	5 (4%)	5 (4%)	4	4
1	C	141/143 (99%)	131 (93%)	5 (4%)	5 (4%)	4	4
1	D	141/143 (99%)	131 (93%)	5 (4%)	5 (4%)	4	4
1	G	141/143 (99%)	131 (93%)	5 (4%)	5 (4%)	4	4
1	I	141/143 (99%)	130 (92%)	6 (4%)	5 (4%)	4	4
1	J	141/143 (99%)	131 (93%)	5 (4%)	5 (4%)	4	4
2	B	100/102 (98%)	92 (92%)	6 (6%)	2 (2%)	9	9
2	E	100/102 (98%)	92 (92%)	6 (6%)	2 (2%)	9	9
2	F	100/102 (98%)	92 (92%)	6 (6%)	2 (2%)	9	9
2	H	100/102 (98%)	92 (92%)	6 (6%)	2 (2%)	9	9
2	K	100/102 (98%)	92 (92%)	6 (6%)	2 (2%)	9	9
2	L	100/102 (98%)	92 (92%)	6 (6%)	2 (2%)	9	9
All	All	1446/1470 (98%)	1337 (92%)	67 (5%)	42 (3%)	9	5

All (42) Ramachandran outliers are listed below:

Mol	Chain	Res	Type
1	A	94	GLU
1	A	140	SER
2	B	94	GLU
1	C	94	GLU
1	C	140	SER
2	E	94	GLU
1	D	94	GLU
1	D	140	SER
2	F	94	GLU
1	G	94	GLU
1	G	140	SER
2	H	94	GLU

Continued on next page...

PRELIMINARY VALIDATION REPORT

Continued from previous page...

Mol	Chain	Res	Type
1	I	94	GLU
1	I	140	SER
2	K	94	GLU
1	J	94	GLU
1	J	140	SER
2	L	94	GLU
1	A	57	ASP
1	A	60	ASP
1	C	57	ASP
1	C	60	ASP
1	D	57	ASP
1	D	60	ASP
1	G	57	ASP
1	G	60	ASP
1	I	57	ASP
1	I	60	ASP
1	J	57	ASP
1	J	60	ASP
1	A	176	ARG
1	C	176	ARG
1	D	176	ARG
1	G	176	ARG
1	I	176	ARG
1	J	176	ARG
2	B	101	MET
2	E	101	MET
2	F	101	MET
2	H	101	MET
2	K	101	MET
2	L	101	MET

5.3.2 Protein sidechains (i)

In the following table, the Percentiles column shows the percent sidechain outliers of the chain as a percentile score with respect to all PDB entries followed by that with respect to all EM entries.

The Analysed column shows the number of residues for which the sidechain conformation was analysed, and the total number of residues.

Mol	Chain	Analysed	Rotameric	Outliers	Percentiles
1	A	132/132 (100%)	119 (90%)	13 (10%)	9 9

Continued on next page...

PRELIMINARY VALIDATION REPORT

Continued from previous page...

Mol	Chain	Analysed	Rotameric	Outliers	Percentiles	
1	C	132/132 (100%)	119 (90%)	13 (10%)	9	9
1	D	132/132 (100%)	119 (90%)	13 (10%)	9	9
1	G	132/132 (100%)	119 (90%)	13 (10%)	9	9
1	I	132/132 (100%)	119 (90%)	13 (10%)	9	9
1	J	132/132 (100%)	119 (90%)	13 (10%)	9	9
2	B	95/95 (100%)	81 (85%)	14 (15%)	3	3
2	E	95/95 (100%)	81 (85%)	14 (15%)	3	3
2	F	95/95 (100%)	81 (85%)	14 (15%)	3	3
2	H	95/95 (100%)	81 (85%)	14 (15%)	3	3
2	K	95/95 (100%)	82 (86%)	13 (14%)	4	4
2	L	95/95 (100%)	81 (85%)	14 (15%)	3	3
All	All	1362/1362 (100%)	1201 (88%)	161 (12%)	10	6

All (161) residues with a non-rotameric sidechain are listed below:

Mol	Chain	Res	Type
1	A	46	LEU
1	A	53	ARG
1	A	81	GLU
1	A	87	ASP
1	A	96	LYS
1	A	99	PHE
1	A	106	LYS
1	A	121	LYS
1	A	126	LYS
1	A	144	ARG
1	A	153	LYS
1	A	171	LYS
1	A	177	LYS
2	B	92	GLU
2	B	93	HIS
2	B	94	GLU
2	B	95	ILE
2	B	106	LYS
2	B	116	ASN
2	B	132	SER
2	B	134	SER
2	B	141	TYR

Continued on next page...

PRELIMINARY VALIDATION REPORT

Continued from previous page...

Mol	Chain	Res	Type
2	B	143	THR
2	B	145	LEU
2	B	155	LYS
2	B	167	ILE
2	B	176	ARG
1	C	46	LEU
1	C	53	ARG
1	C	81	GLU
1	C	87	ASP
1	C	96	LYS
1	C	99	PHE
1	C	106	LYS
1	C	121	LYS
1	C	126	LYS
1	C	144	ARG
1	C	153	LYS
1	C	171	LYS
1	C	177	LYS
2	E	92	GLU
2	E	93	HIS
2	E	94	GLU
2	E	95	ILE
2	E	106	LYS
2	E	116	ASN
2	E	132	SER
2	E	134	SER
2	E	141	TYR
2	E	143	THR
2	E	145	LEU
2	E	155	LYS
2	E	167	ILE
2	E	176	ARG
1	D	46	LEU
1	D	53	ARG
1	D	81	GLU
1	D	87	ASP
1	D	96	LYS
1	D	99	PHE
1	D	106	LYS
1	D	121	LYS
1	D	126	LYS
1	D	144	ARG

Continued on next page...

PRELIMINARY VALIDATION REPORT

Continued from previous page...

Mol	Chain	Res	Type
1	D	153	LYS
1	D	171	LYS
1	D	177	LYS
2	F	92	GLU
2	F	93	HIS
2	F	94	GLU
2	F	95	ILE
2	F	106	LYS
2	F	116	ASN
2	F	132	SER
2	F	134	SER
2	F	141	TYR
2	F	143	THR
2	F	145	LEU
2	F	155	LYS
2	F	167	ILE
2	F	176	ARG
1	G	46	LEU
1	G	53	ARG
1	G	81	GLU
1	G	87	ASP
1	G	96	LYS
1	G	99	PHE
1	G	106	LYS
1	G	121	LYS
1	G	126	LYS
1	G	144	ARG
1	G	153	LYS
1	G	171	LYS
1	G	177	LYS
2	H	92	GLU
2	H	93	HIS
2	H	94	GLU
2	H	95	ILE
2	H	106	LYS
2	H	116	ASN
2	H	132	SER
2	H	134	SER
2	H	141	TYR
2	H	143	THR
2	H	145	LEU
2	H	155	LYS

Continued on next page...

PRELIMINARY VALIDATION REPORT

Continued from previous page...

Mol	Chain	Res	Type
2	H	167	ILE
2	H	176	ARG
1	I	46	LEU
1	I	53	ARG
1	I	81	GLU
1	I	87	ASP
1	I	96	LYS
1	I	99	PHE
1	I	106	LYS
1	I	121	LYS
1	I	126	LYS
1	I	144	ARG
1	I	153	LYS
1	I	171	LYS
1	I	177	LYS
2	K	92	GLU
2	K	93	HIS
2	K	94	GLU
2	K	95	ILE
2	K	106	LYS
2	K	116	ASN
2	K	134	SER
2	K	141	TYR
2	K	143	THR
2	K	145	LEU
2	K	155	LYS
2	K	167	ILE
2	K	176	ARG
1	J	46	LEU
1	J	53	ARG
1	J	81	GLU
1	J	87	ASP
1	J	96	LYS
1	J	99	PHE
1	J	106	LYS
1	J	121	LYS
1	J	126	LYS
1	J	144	ARG
1	J	153	LYS
1	J	171	LYS
1	J	177	LYS
2	L	92	GLU

Continued on next page...

PRELIMINARY VALIDATION REPORT

Continued from previous page...

Mol	Chain	Res	Type
2	L	93	HIS
2	L	94	GLU
2	L	95	ILE
2	L	106	LYS
2	L	116	ASN
2	L	132	SER
2	L	134	SER
2	L	141	TYR
2	L	143	THR
2	L	145	LEU
2	L	155	LYS
2	L	167	ILE
2	L	176	ARG

Some sidechains can be flipped to improve hydrogen bonding and reduce clashes. All (12) such sidechains are listed below:

Mol	Chain	Res	Type
1	A	54	GLN
2	B	146	GLN
1	C	54	GLN
2	E	146	GLN
1	D	54	GLN
2	F	146	GLN
1	G	54	GLN
2	H	146	GLN
1	I	54	GLN
2	K	146	GLN
1	J	54	GLN
2	L	146	GLN

5.3.3 RNA (i)

There are no RNA molecules in this entry.

5.4 Non-standard residues in protein, DNA, RNA chains (i)

There are no non-standard protein/DNA/RNA residues in this entry.

PRELIMINARY VALIDATION REPORT

5.5 Carbohydrates (i)

There are no carbohydrates in this entry.

5.6 Ligand geometry (i)

There are no ligands in this entry.

5.7 Other polymers (i)

There are no such residues in this entry.

5.8 Polymer linkage issues (i)

There are no chain breaks in this entry.

PRELIMINARY VALIDATION REPORT
