# Supplementary material for: Structural model of dodecameric heat-shock protein Hsp21: Flexible N-terminal arms interact with client proteins while C-terminal tails maintain the dodecamer and chaperone activity
Source: J Biol Chem. 2017 Mar 21;292(19):8103–21. doi: 10.1074/jbc.M116.766816 (PMC5427286; doi:10.1074/jbc.M116.766816)

Structural model of dodecameric heat-shock protein Hsp21: Flexible N-terminal arms interact with client proteins while C-terminal tails maintain the dodecamer and chaperone activity

*Gudrun Rutsdottir[1], *Johan Härmark[2], Yoran Weide[1], Hans Hebert[2], Morten Ib Rasmussen[3], Sven Wernersson[4], Michal Respondek[4], Mikael Akke[4], Peter Højrup[3], Philip J. B. Koeck[2], Christopher A. G. Söderberg[5] and Cecilia Emanuelsson[1]
[1]Department of Biochemistry and Structural Biology, Lund University, Sweden, [2]School of Technology and Health, KTH/Royal Institute of Technology and Department of Biosciences and Nutrition, Karolinska Institutet, Stockholm, Sweden, [3]Department of Biochemistry and Molecular Biology, University of Southern Denmark, Odense, Denmark, [4]Department of Biophysical Chemistry, Lund University, Sweden, [5]MAX IV Laboratory, Lund University, Sweden. *equally shared first authors

Supplemental information 5 MS and MSMS spectra for crosslinked Hsp21 peptides

This file contains mass spectra (MSMS, or MSMS and ^{14}N-^{15}N MS) in support for the identified crosslinked peptides in Table 1.

File name: Supplemental information 5_MS and MSMS-spectra.pdf

MS and MSMS-spectra for crosslinked peptides in Table 1
List of content:

Mass spectrometric data (MSMS, or MSMS and ^{14}N-^{15}N MS) for crosslinked peptides follow the order in Table 1 accordingly:

Lys A	Lys B	Peptide A	Peptide B	[b] Detected in subunit or dimer band or IS	[c]Detected as hybrid crosslink in IS	**SI**
1	18	MQDQR	ENSIDVVQQGQQKGNQGSSVEK	IS	Y	**S-4, S-5**
1	27	MQDQR	GNQGSSVEKRPQQR	IG-M, IS	Y	**S-6 – S-8**
1	106	MQDQR	MRFDMPGLSKEDVK	IS	N	**S-9, S-10**
1	110	MQDQR	EDVKISVEDNVLVIK	IG-D	n.d	**S-11**
1	125	MQDQR	GEQKKEDSDDSWSGR	IG-M, IG-D, IS	Y	**S-12 – S-16**
1	126	MQDQR	KEDSDDSWSGR	IG-M, IS	Y	**S-17 – S-21**
1	157	MQDQR	IKAELK	IG-M, IS	Y	**S-22, S-23**
1	161	MQDQR	AELKNGVLFITIPK	IG-M, IG-D, IS	Y	**S-24 – S-27**
1	173	MQDQR	TKVER	IG-M, IS	Y	**S-28 – S-30**
27	121	GNQGSSVEKRPQQR	ISVEDNVLVIKGEQK	IS	Y*	**S-31, S-32**
89	125	APWDIKEEEHEIK	GEQKKEDSDDSWSGR	IG-M	n.d	**S-33**
89	126	APWDIKEEEHEIKMR	KEDSDDSWSGR	IG-M, IS	Y*	**S-34 – S-37**
89	173	APWDIKEEEHEIK	TKVER	IS	N	**S-38, S-39**
96	126	APWDIKEEEHEIKMR	KEDSDDSWSGR	IG-M	n.d	**S-40**
106	161	FDMPGLSKEDVK	AELKNGVLFITIPK	IG-M, IS	Y	**S-41 – S-43**
121	126	ISVEDNVLVIKGEQK	KEDSDDSWSGR	IG-M	N	**S-44 – S-46**
121	173	ISVEDNVLVIKGEQK	TKVER	IS	Y*	**S-47, S-48**
125	161	GEQKKEDSDDSWSGR	AELKNGVLFITIPK	IG-D	n.d	**S-49**
126	157	KEDSDDSWSGR	IKAELKNGVLFITIPK	IG-M	n.d	**S-50**
126	161	KEDSDDSWSGR	AELKNGVLFITIPK	IG-D, IS	Y*	**S-51 – S-55**
153	157	LQLPDNCEKDK	IKAELK	IG-M	n.d	**S-56**
157	173	IKAELK	TKVER	IG-M, IG-D	n.d	**S-58**

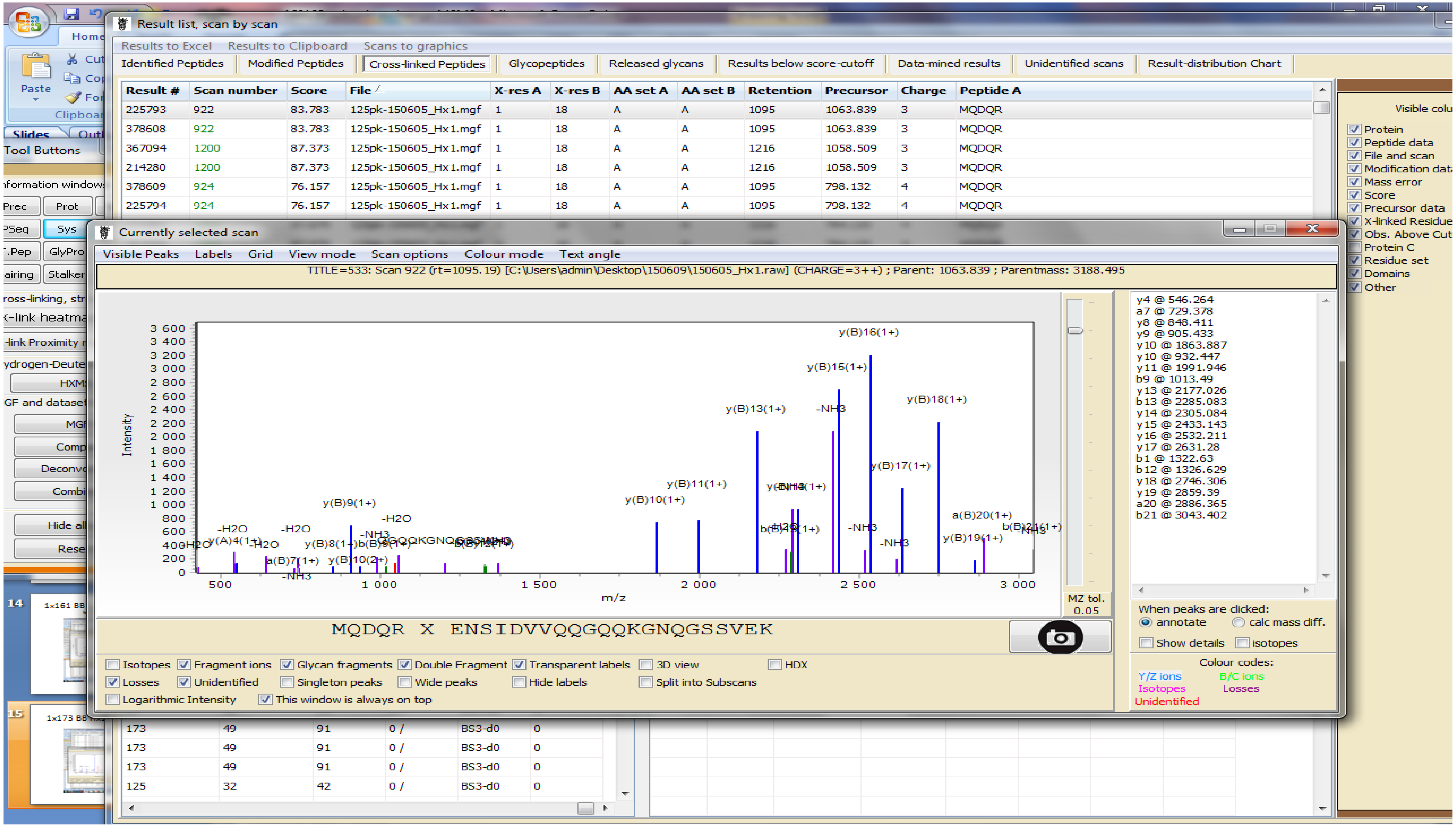

MS- and MSMS-spectra supporting crosslink data. An example of output from analysis of MSMS-data with the software MassAI is shown above, and the following pages show MSMS-spectra for each of the crosslinks listed in Table 1and additional MS MS-spectra for mixed iosotope labelling crosslinks needing further prooves for being intra- or inter-subunit crosslinks. The MS-spectra were exported from Mascot distiller and the MSMS-spectra were exported as emf-files from the MassAI software with all annotations of ions retained and presented with transparent labels for convenient overview. Fragment-rich spectra require zooming in for full legibility. Color coding in MSMS spectra: y-ions (blue), b-ions (green), peptide A/B ions (purple), unexplained (red).

MSMS
MQDQRxENSIDVVQQGQQKGNQGSSVEK 1x18 ^{14}Nx^{14}N
File: 150609_Hx1

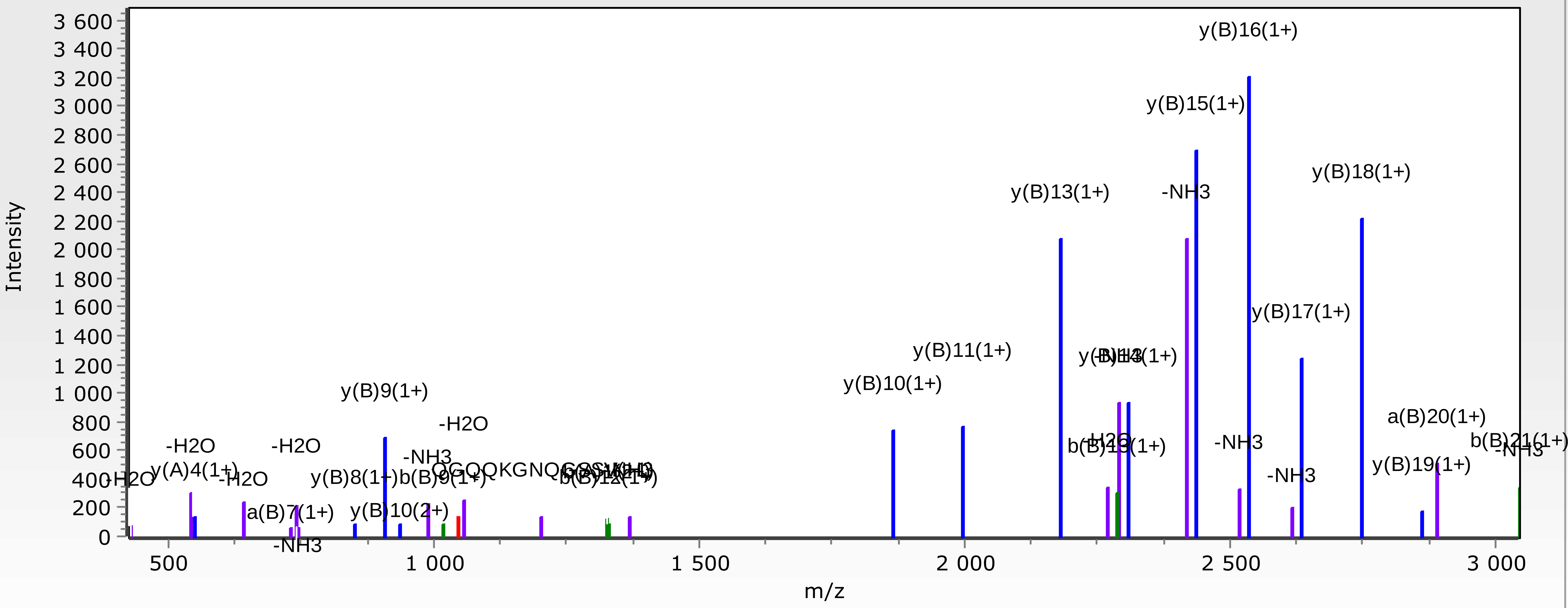

MSMS
MQDQRxENSIDVVQQGQQKGNQGSSVEK 1x18 ^{15}Nx^{14}N
File: 150609_Hx1

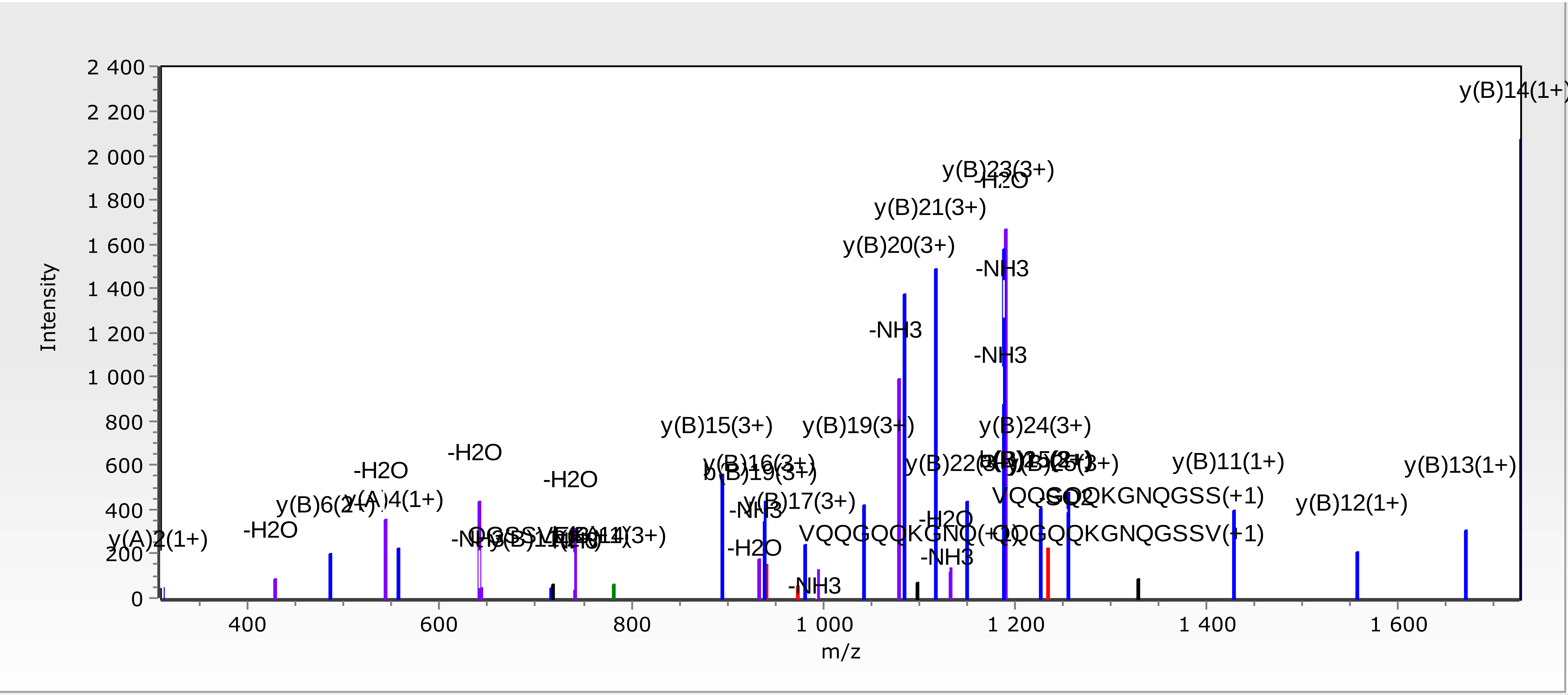

MSMS

MQDQRxGNQGSSVEKRPQQR 1x27 $^{14}N\text{x}^{14}N$

File: 150609_Hx1

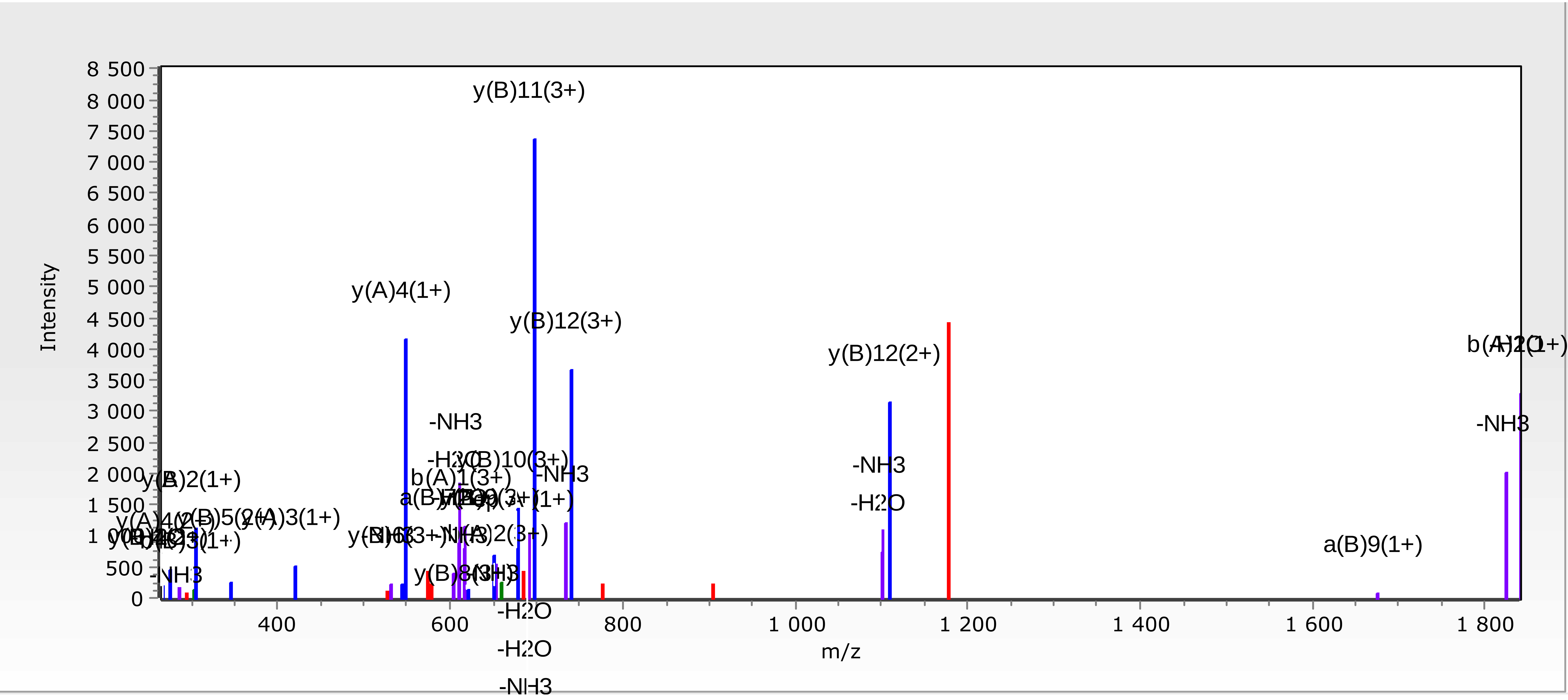

MS

MQDQRxGNQGSSVEKRPQQR 1x27

File: 150609_Hx1

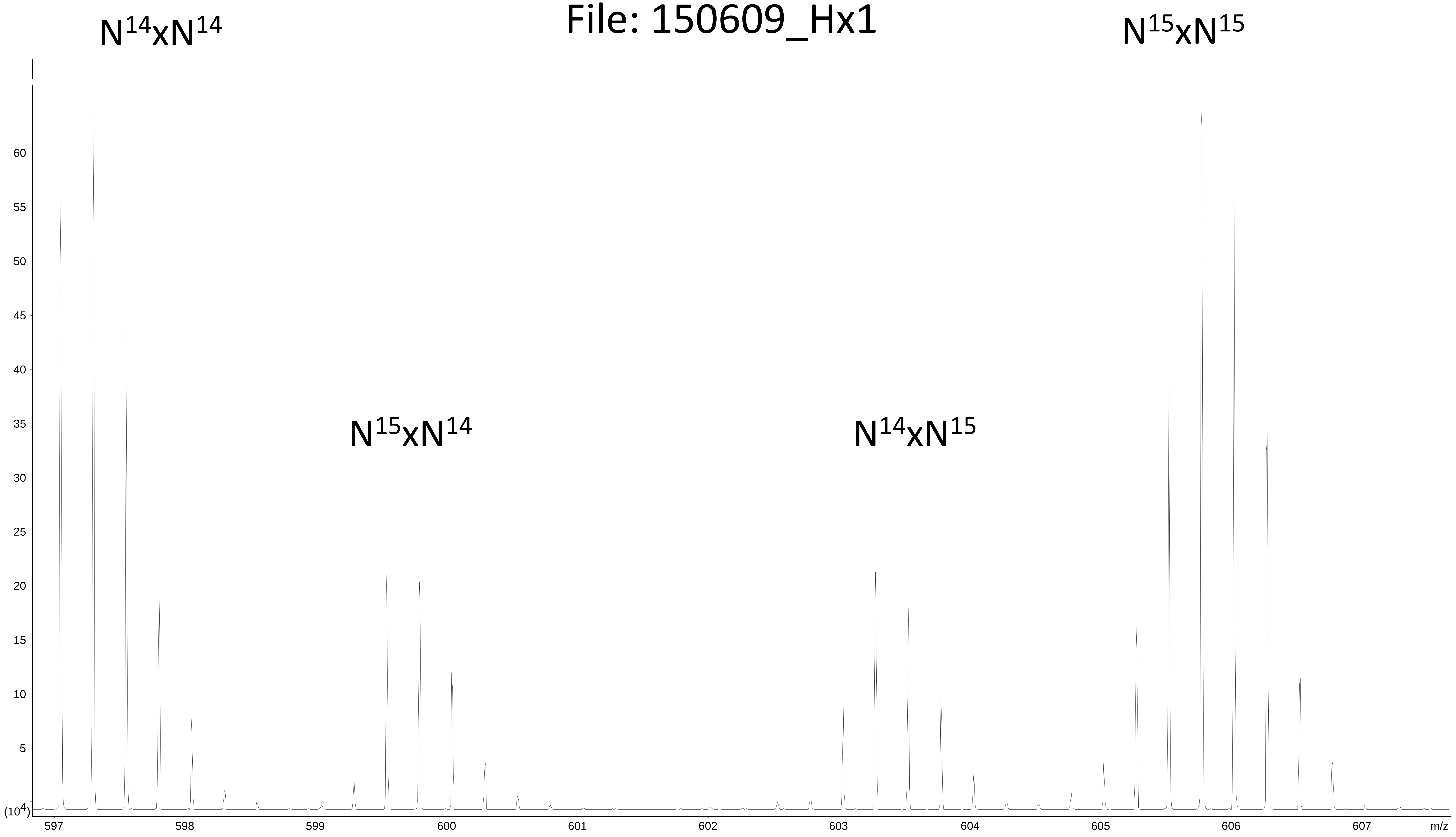

MS-spectrum showing hybrid-crosslinks **=> inter-subunit crosslink**
(MS-data shown since this crosslink only MSMS-fragmented as $N^{14}xN^{14}$)

MSMS
MQDQRxGNQGSSVEKRPQQR 1x27
File: 150121_Hsp21 monomeric band

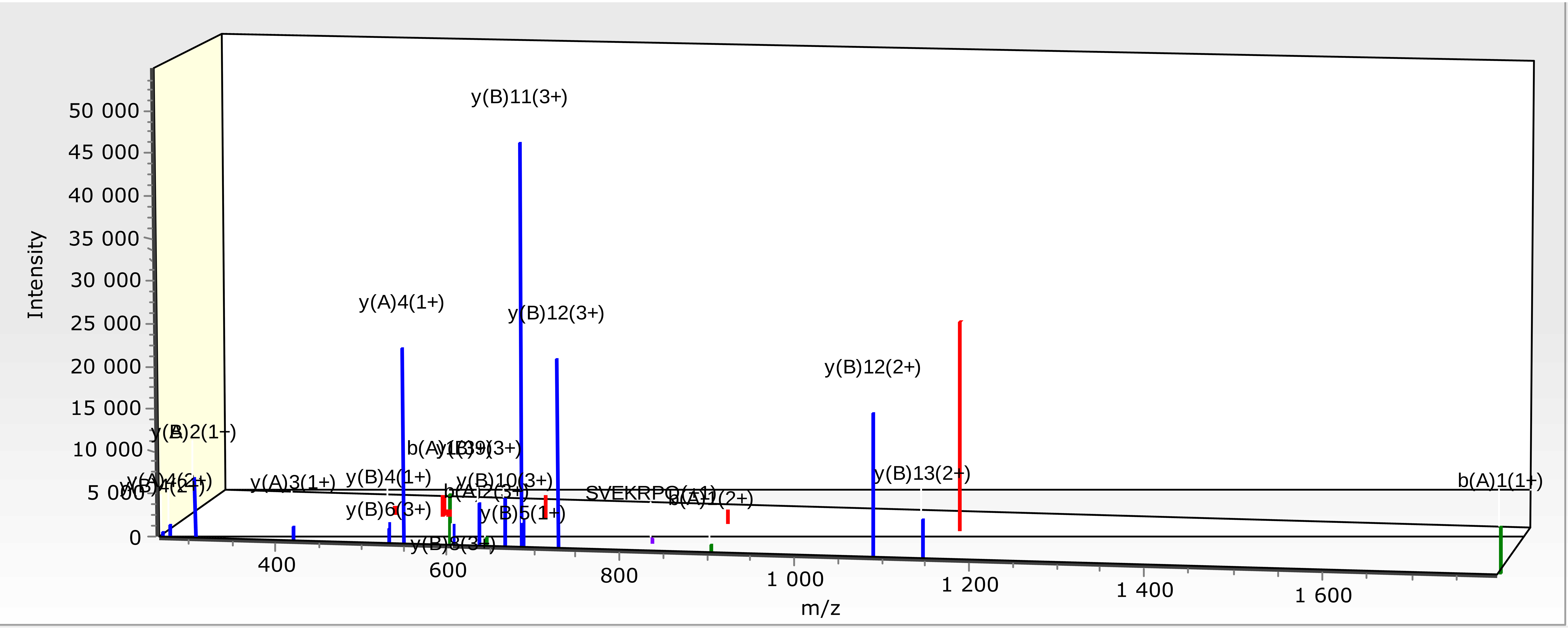

MSMS
MQDQRxMRFDMPGLSKEDVK 1x106 ^{14}Nx^{14}N
File: 150609_Hx1

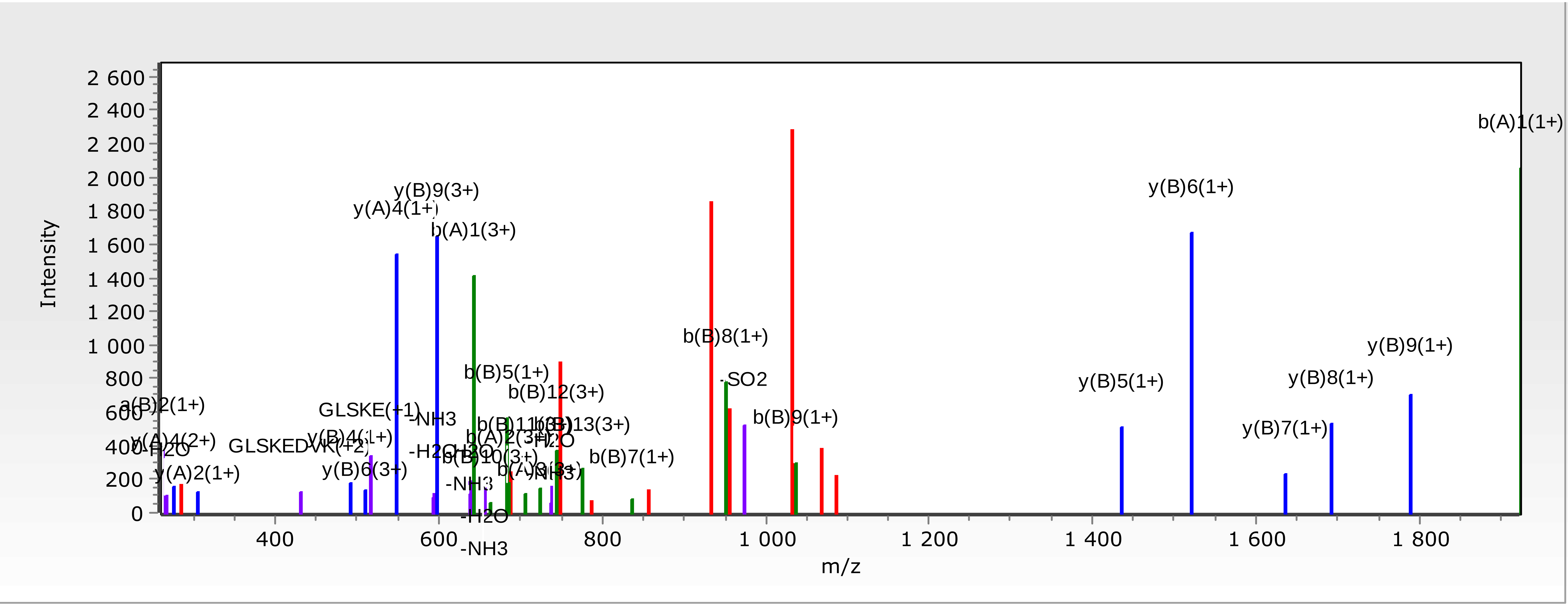

MS
MQDQRxMRFDMPGLSKEDVK 1x106
File: 150609_Hx1 rt: 1521

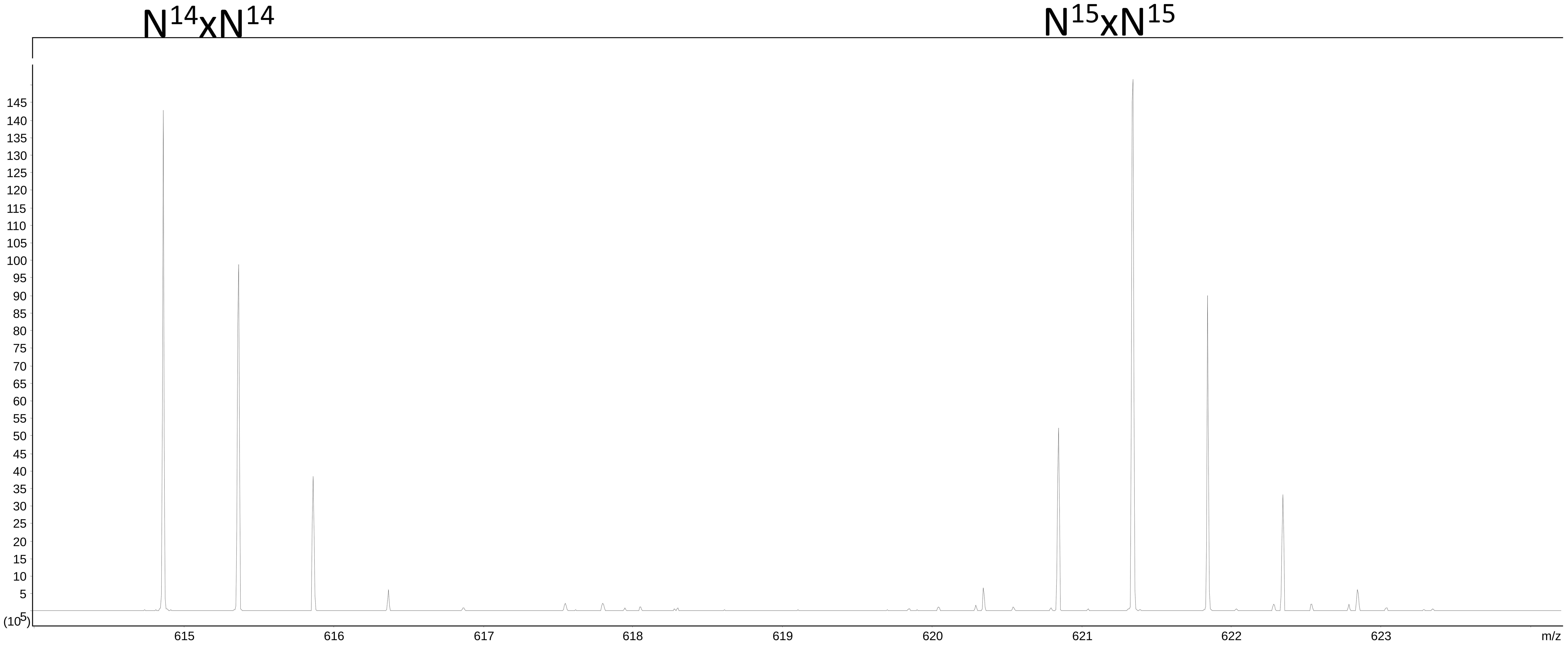

MS spectrum showing no hybrid crosslinks **=> intra-subunit crosslink**

MSMS
MQDQRxEDVKISVEDNVLVIK 1x110
File: 160307_Hsp21 dimeric band

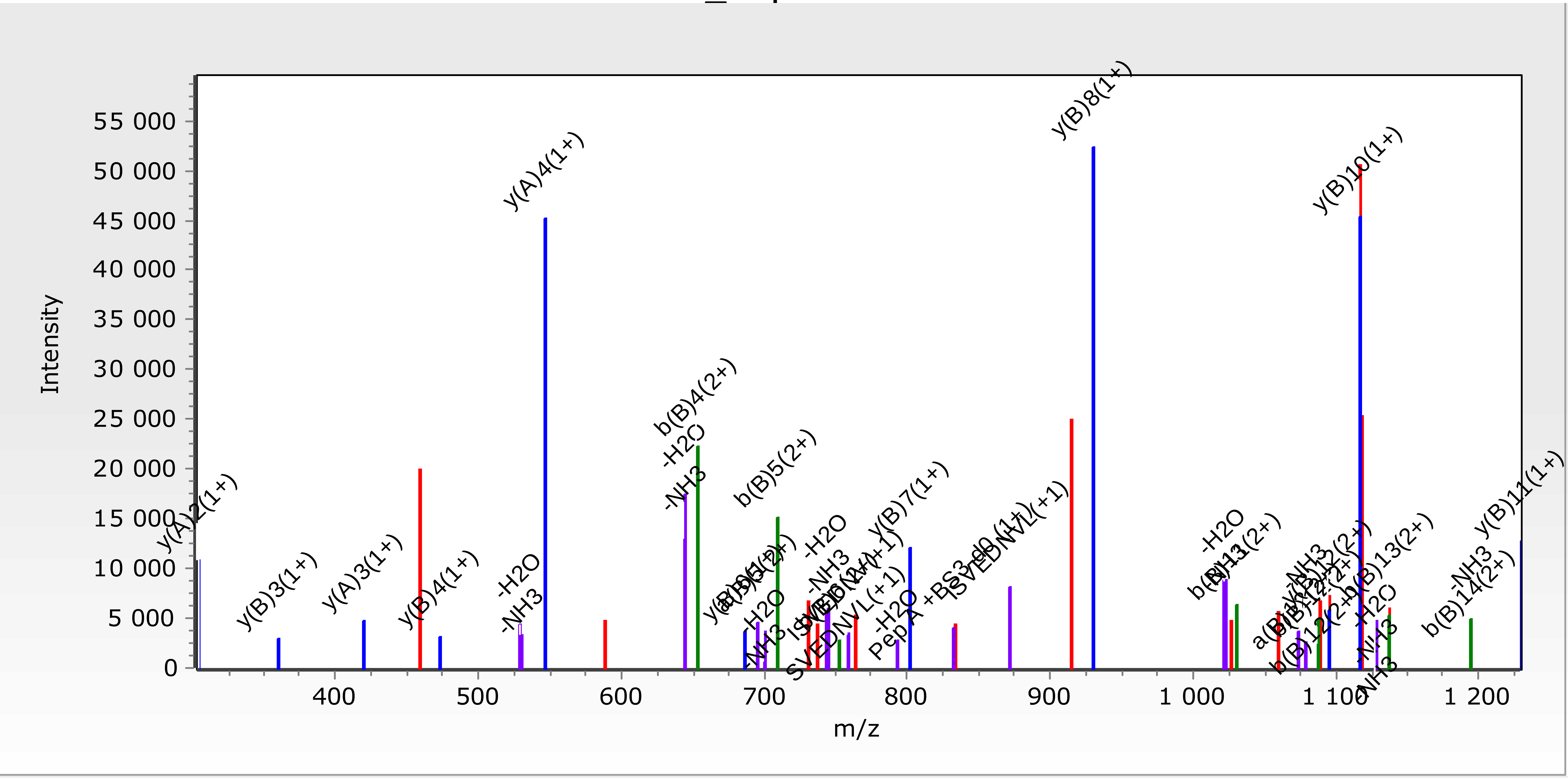

MSMS
MQDQRxGEQKKEDSDDSWSGR 1x125
Files: 150521_Hsp21 monomeric band

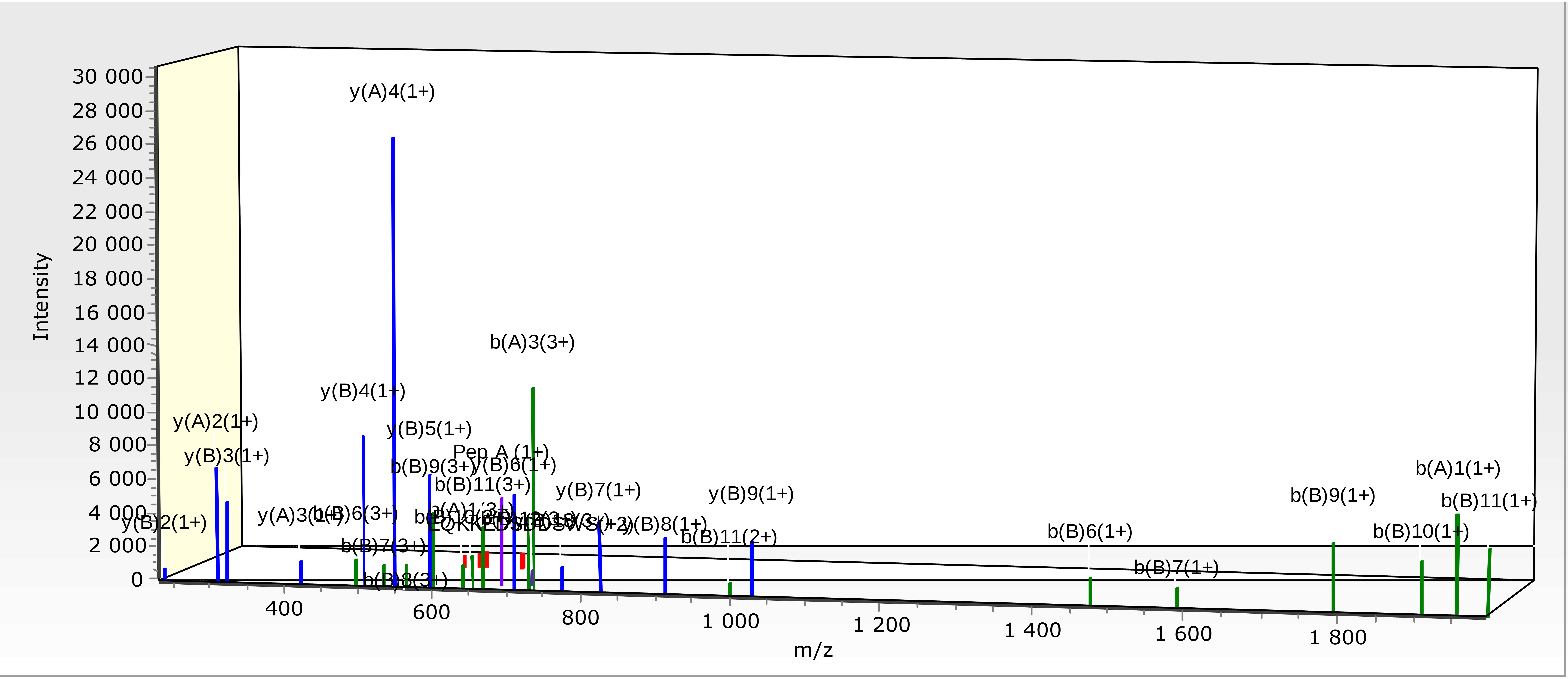

MSMS
MQDQRxGEQKKEDSDDSWSGR 1x125
File: 160307_Hsp21 dimeric band

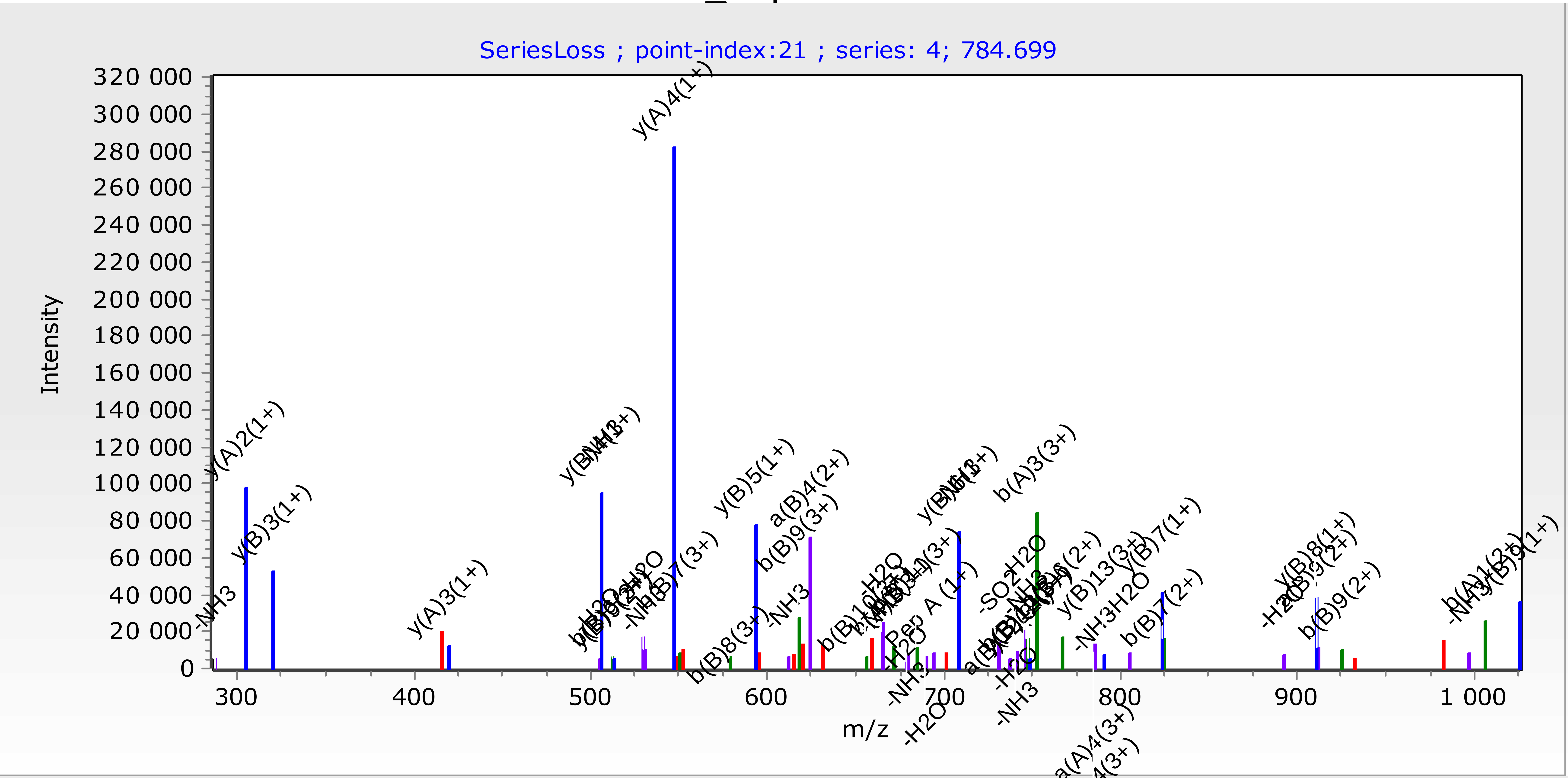

MSMS

MQDQRxGEQKKEDSDDSWSGR 1x125 ^{14}Nx^{14}N

File: 150609_Hx1

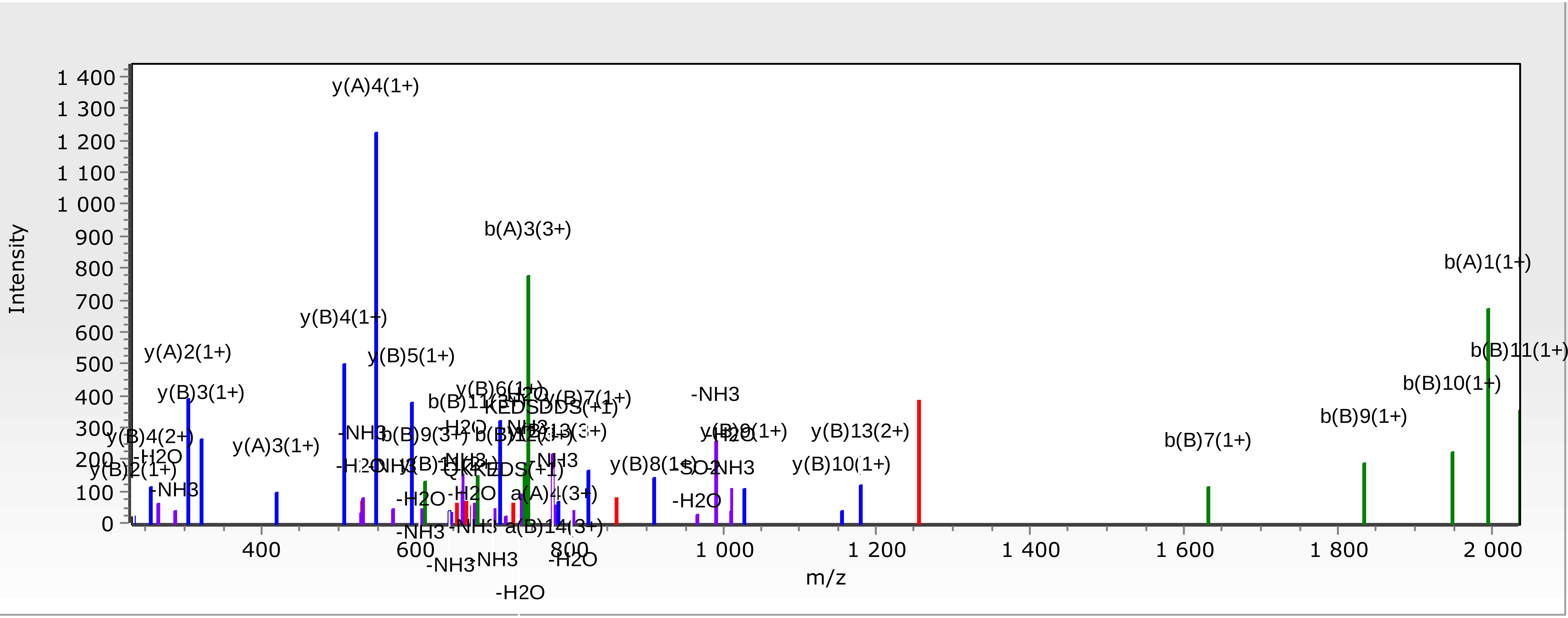

MSMS
MQDQRxGEQKKEDSDDSWSGR 1x125 ^{15}Nx^{15}N
File: 150609_Hx1

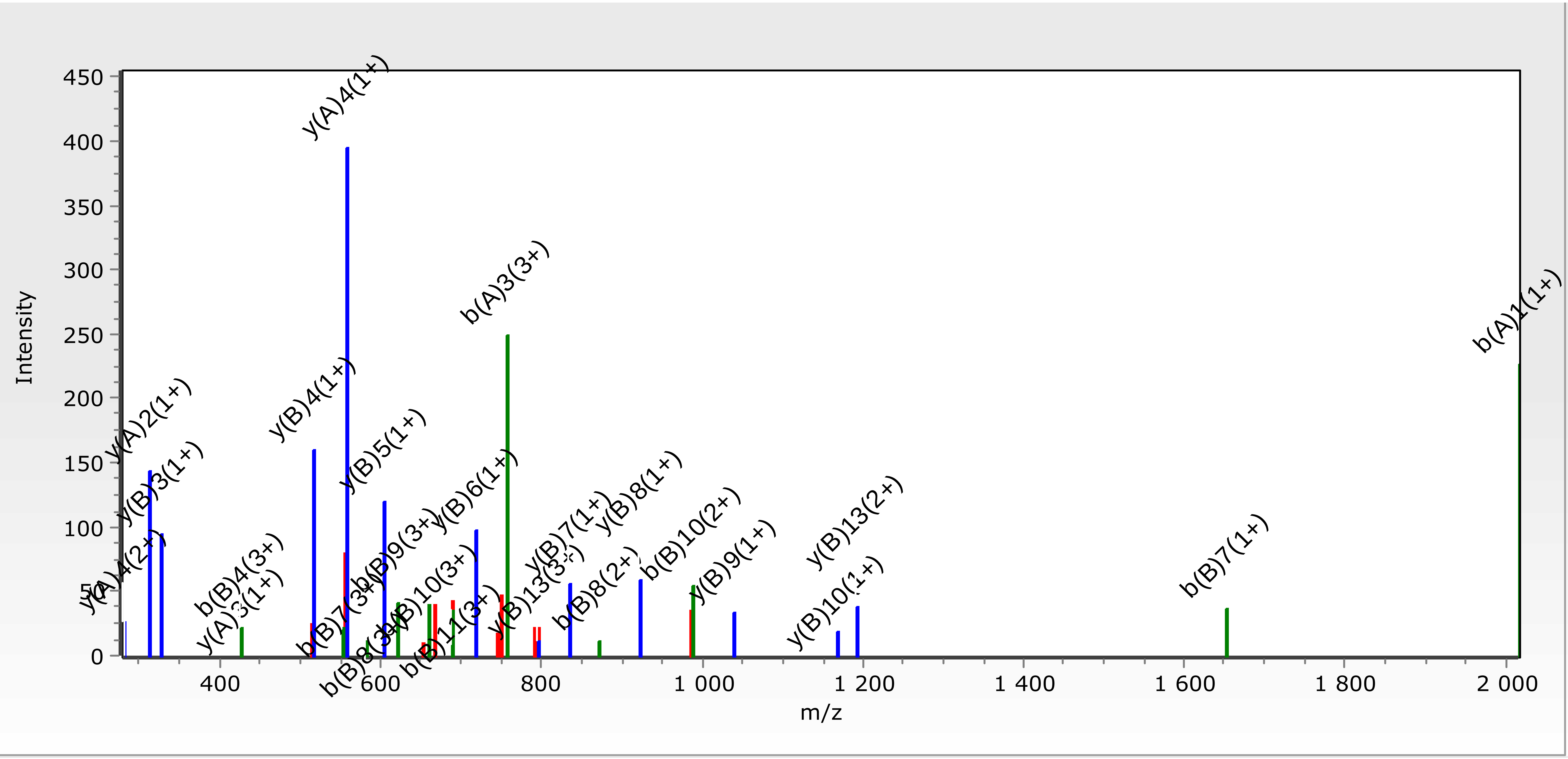

MSMS
MQDQRxGEQKKEDSDDSWSGR 1x125 ^{15}Nx^{14}N
File: 150609_Hx1

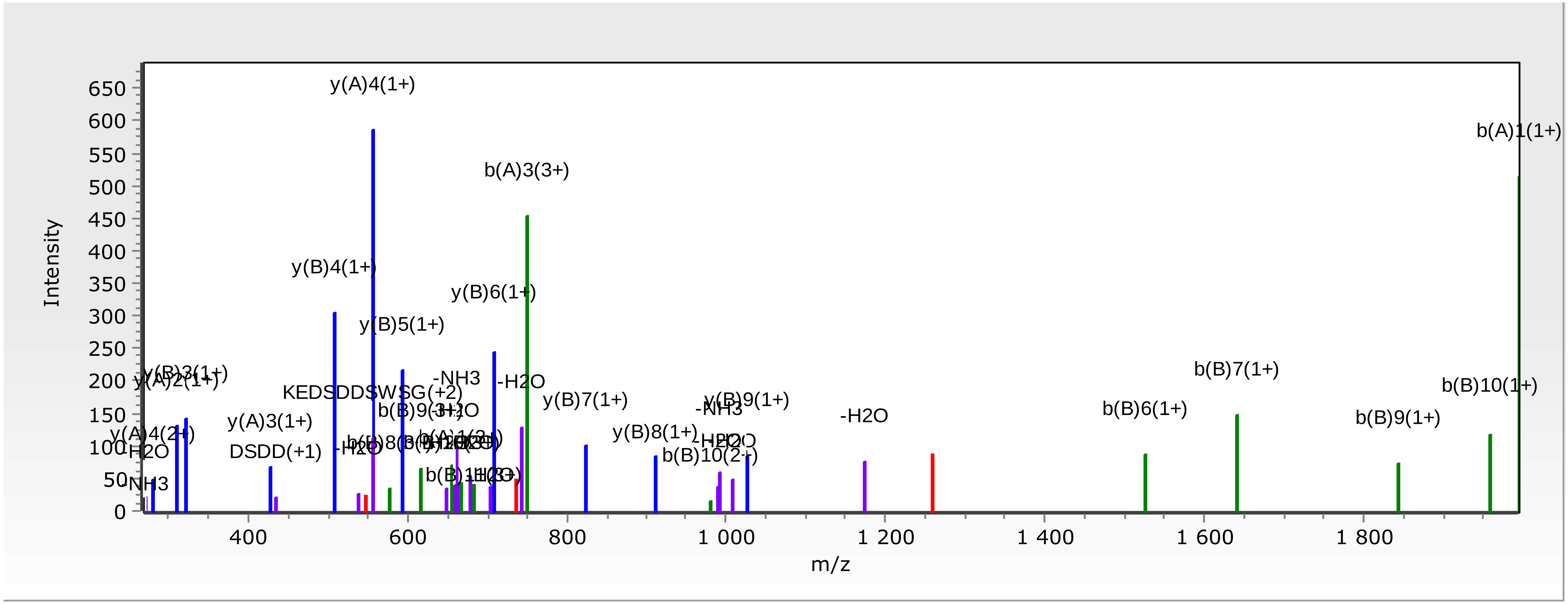

MSMS
MQDQRxKEDSDDSWSGR 1x126
File: 150525_Hsp21 monomeric band

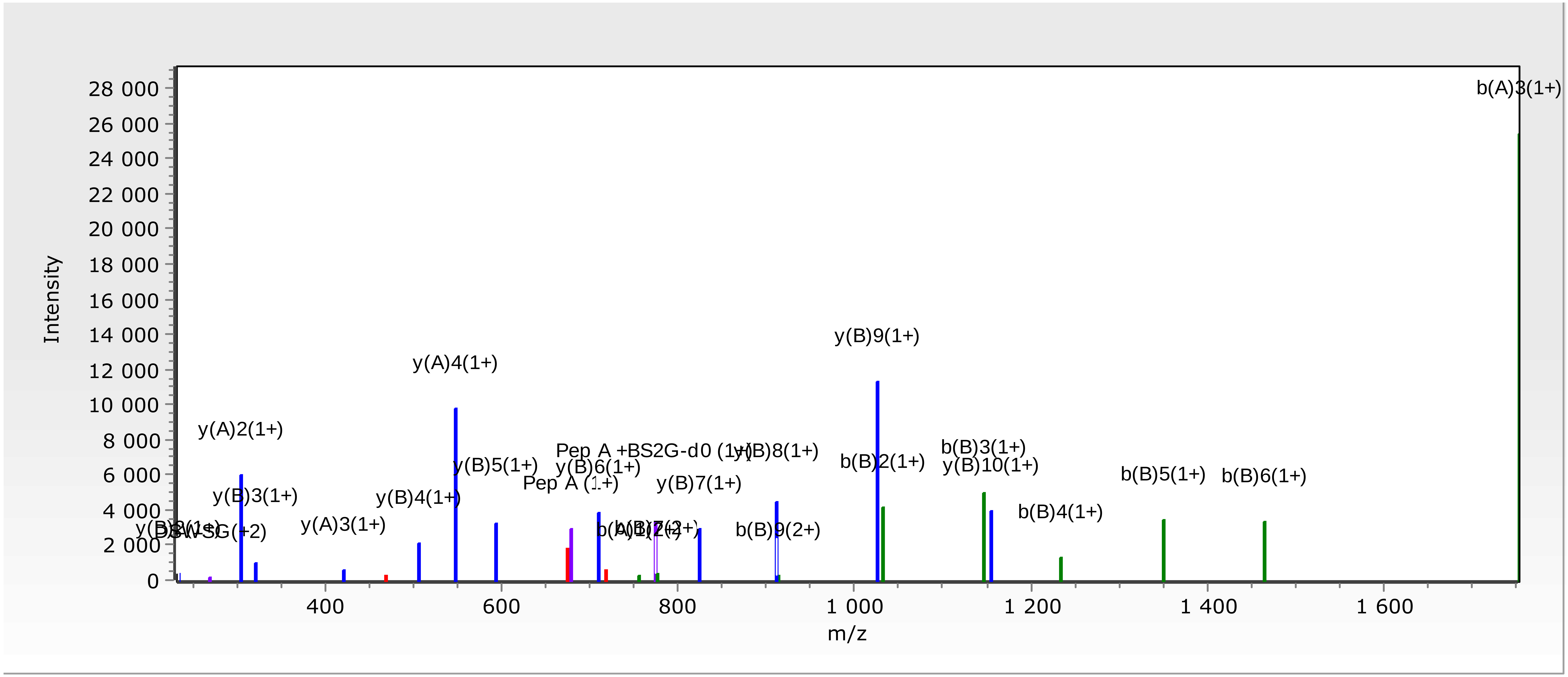

MSMS
MQDQRxKEDSDDSWSGR 1x126 ^{15}Nx^{15}N
File: 150609_Hx1

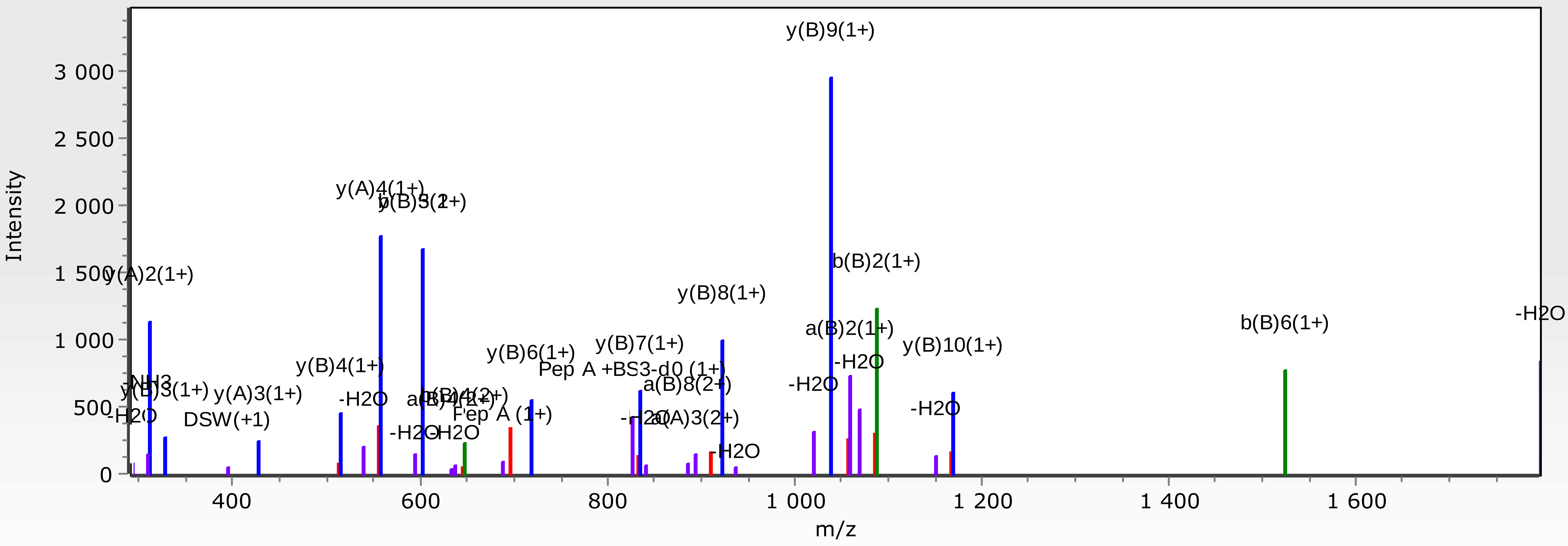

MSMS

MQDQRxKEDSDDSWSGR 1x126 ^{14}Nx^{15}N

File: 150609_Hx1

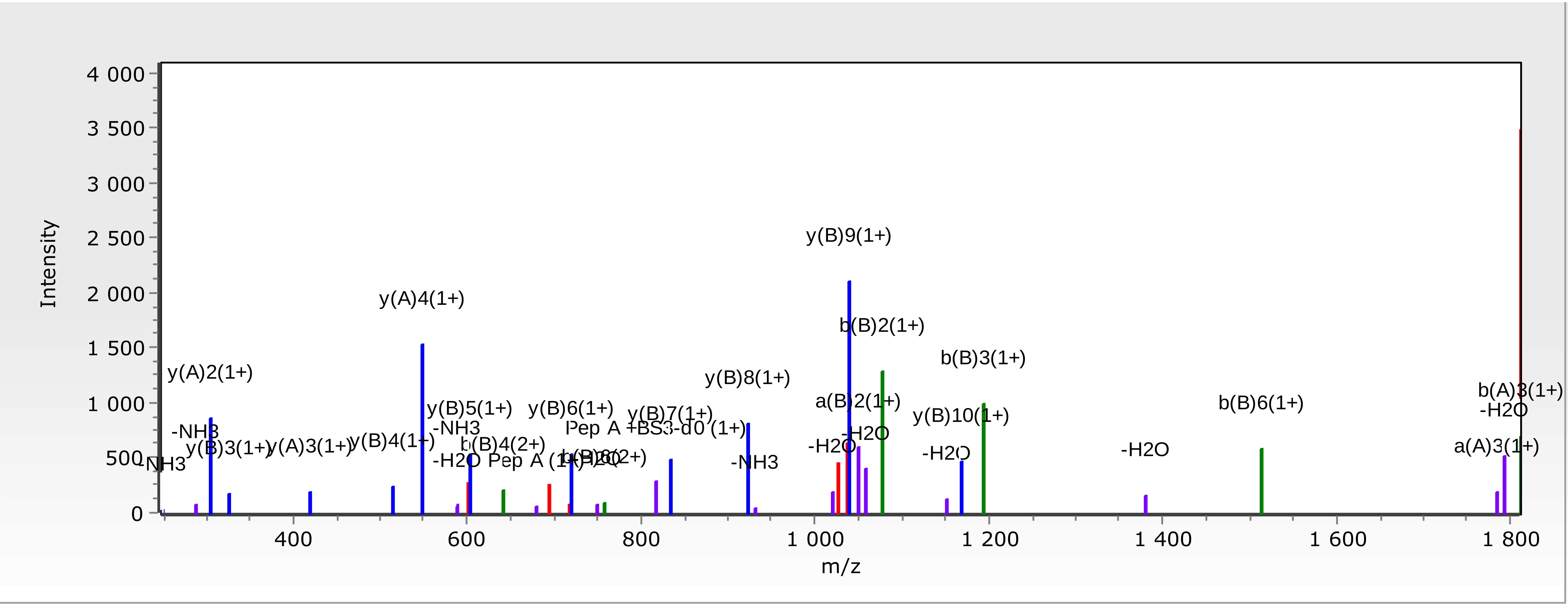

MSMS
MQDQRxKEDSDDSWSGR 1x126 ^{15}Nx^{14}N
File: 150609_Hx1

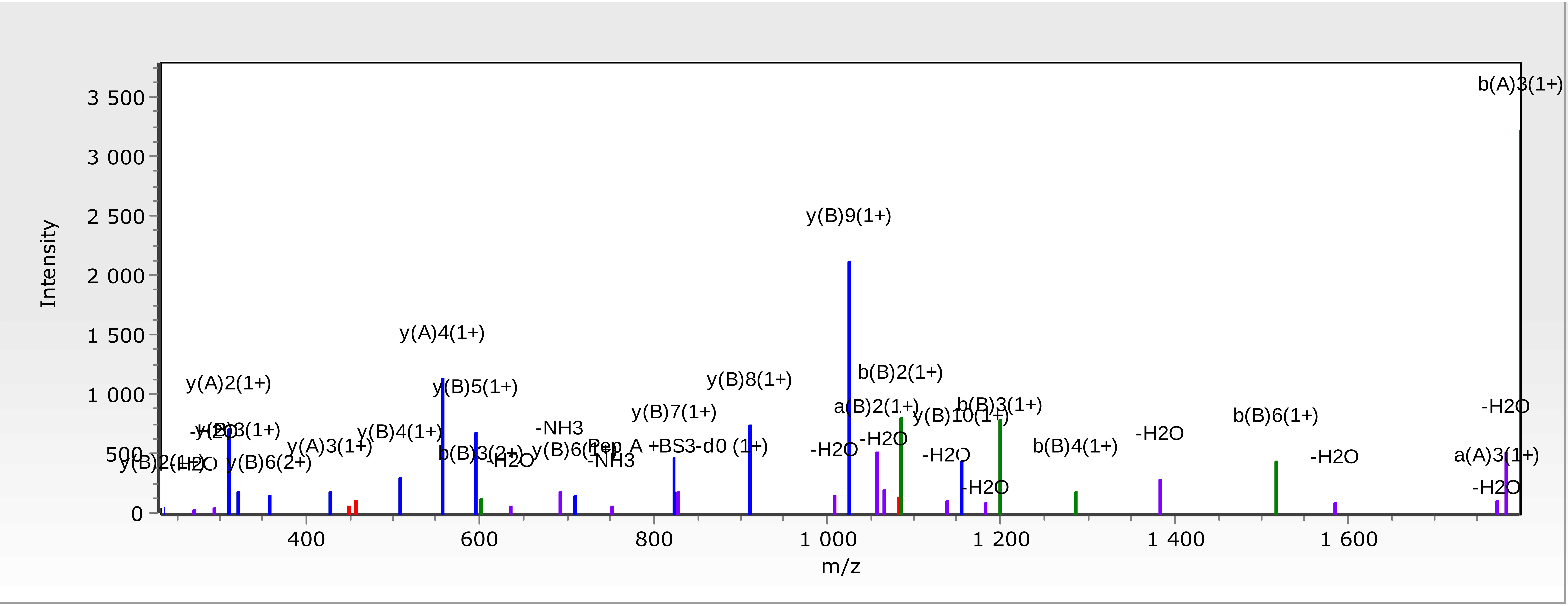

MSMS

MQDQRxKEDSDDSWSGR 1x126 ^{14}Nx^{14}N

File: 150609_Hx1

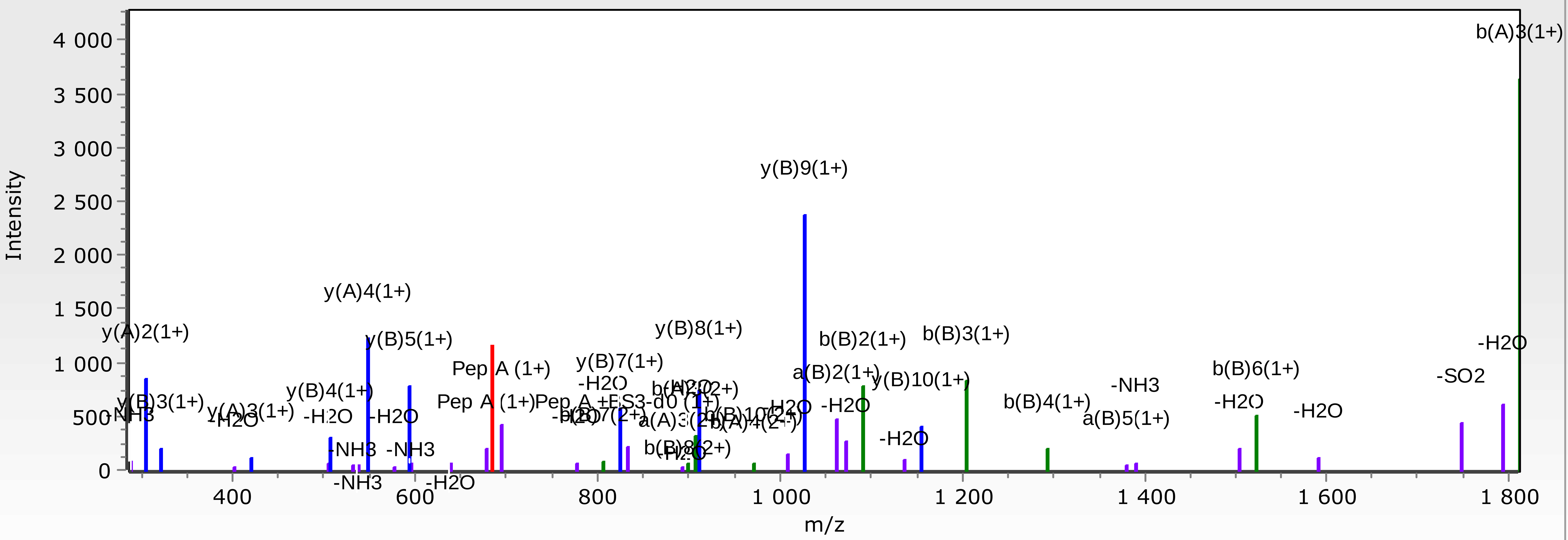

MSMS

MQDQRxIKAELK 1x157 $^{14}Nx^{14}N$

File: 150609_Hx1

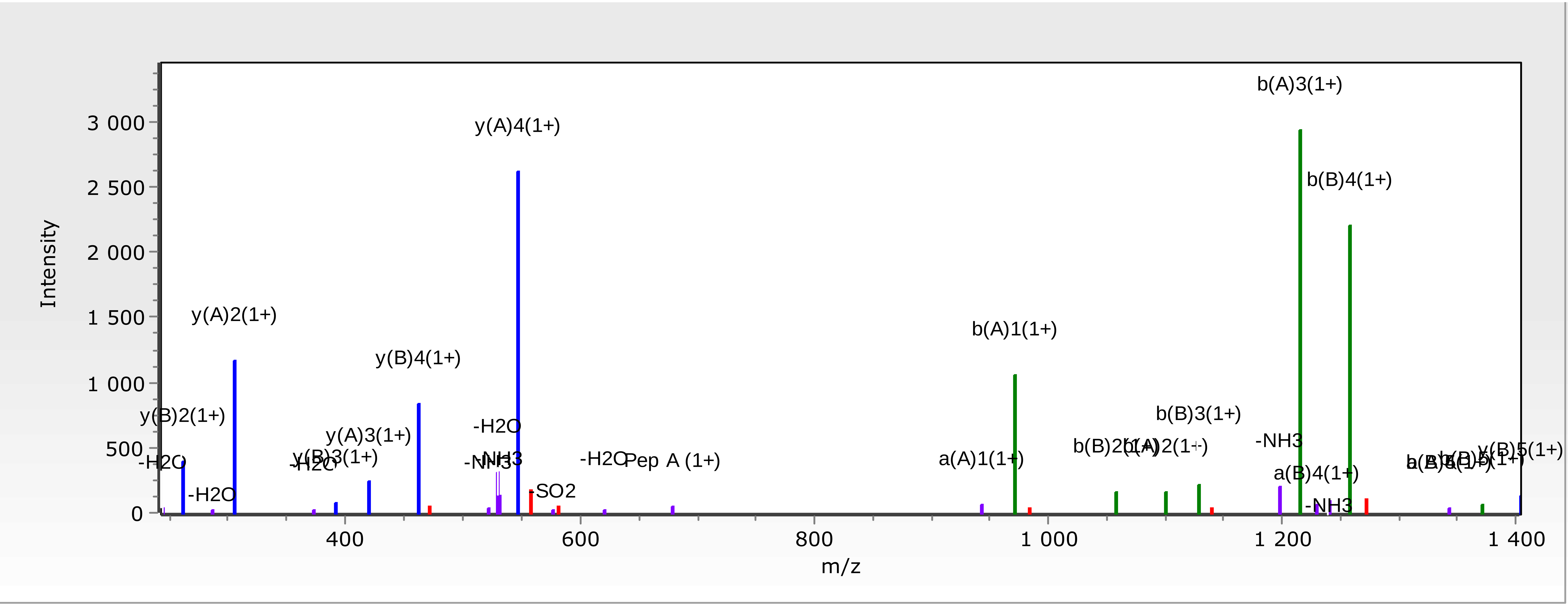

MSMS
MQDQRxIKAELK 1x157
File: 150521 Hsp21 monomeric band

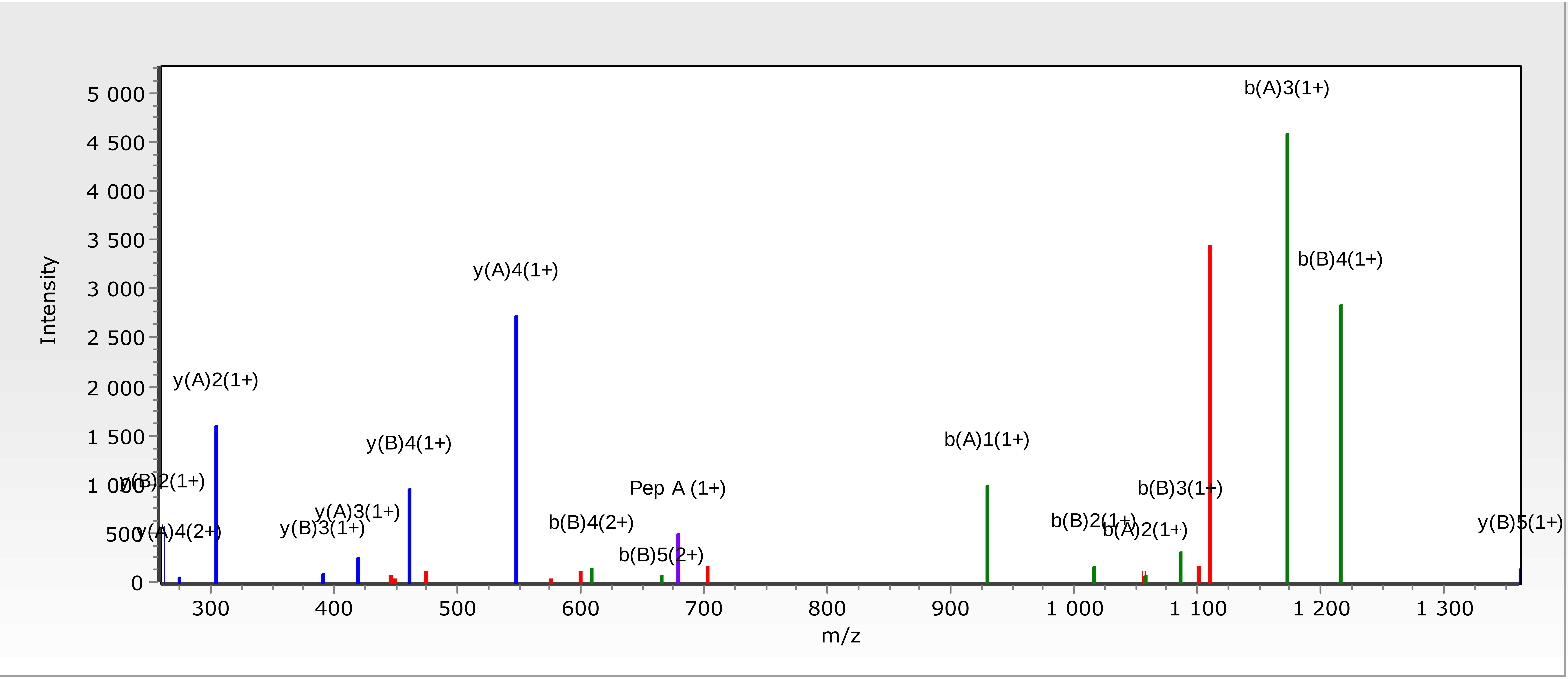

MSMS
MQDQRxAELKNGVLFITIPK 1x161
File: 150521_Hsp21 monomeric band

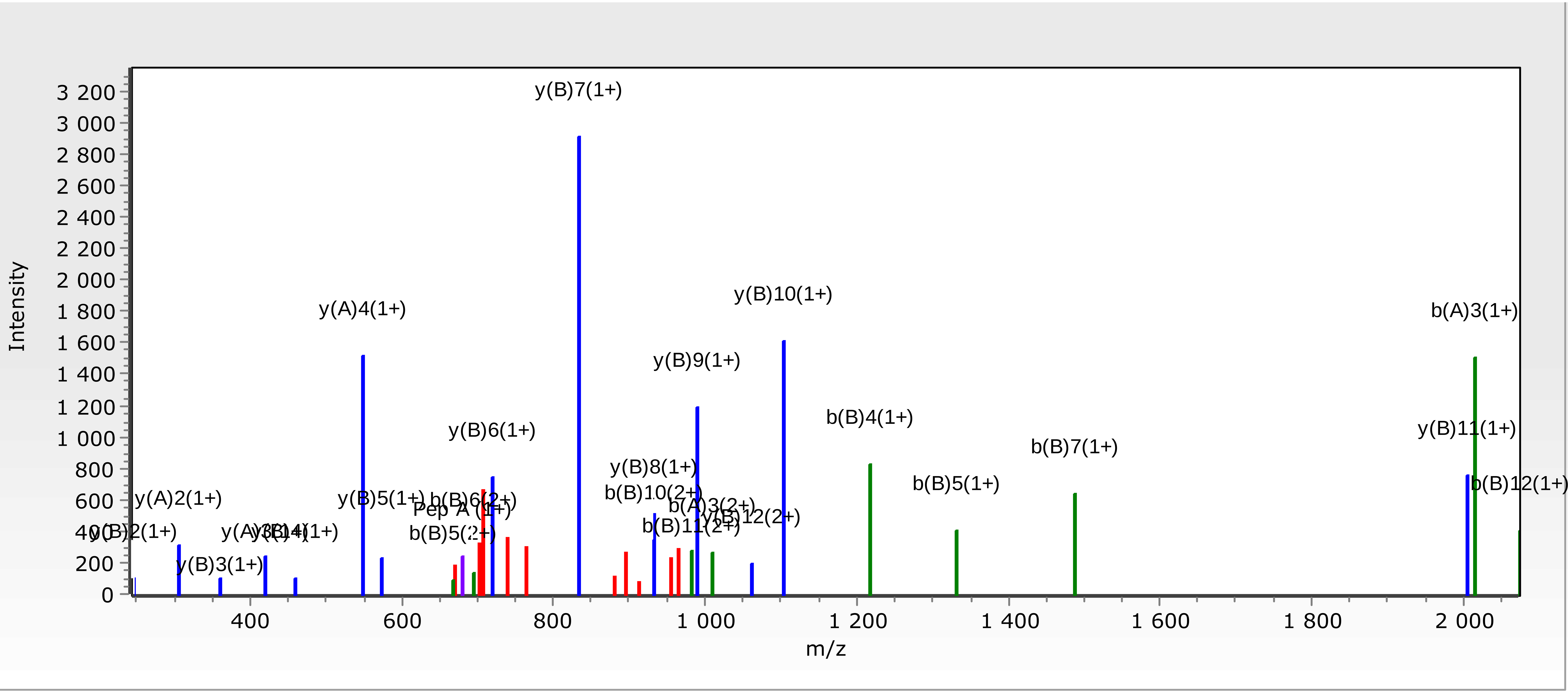

MSMS

MQDQRxAELKNGVLFITIPK 1x161

File: 160307_Hsp21 dimeric band

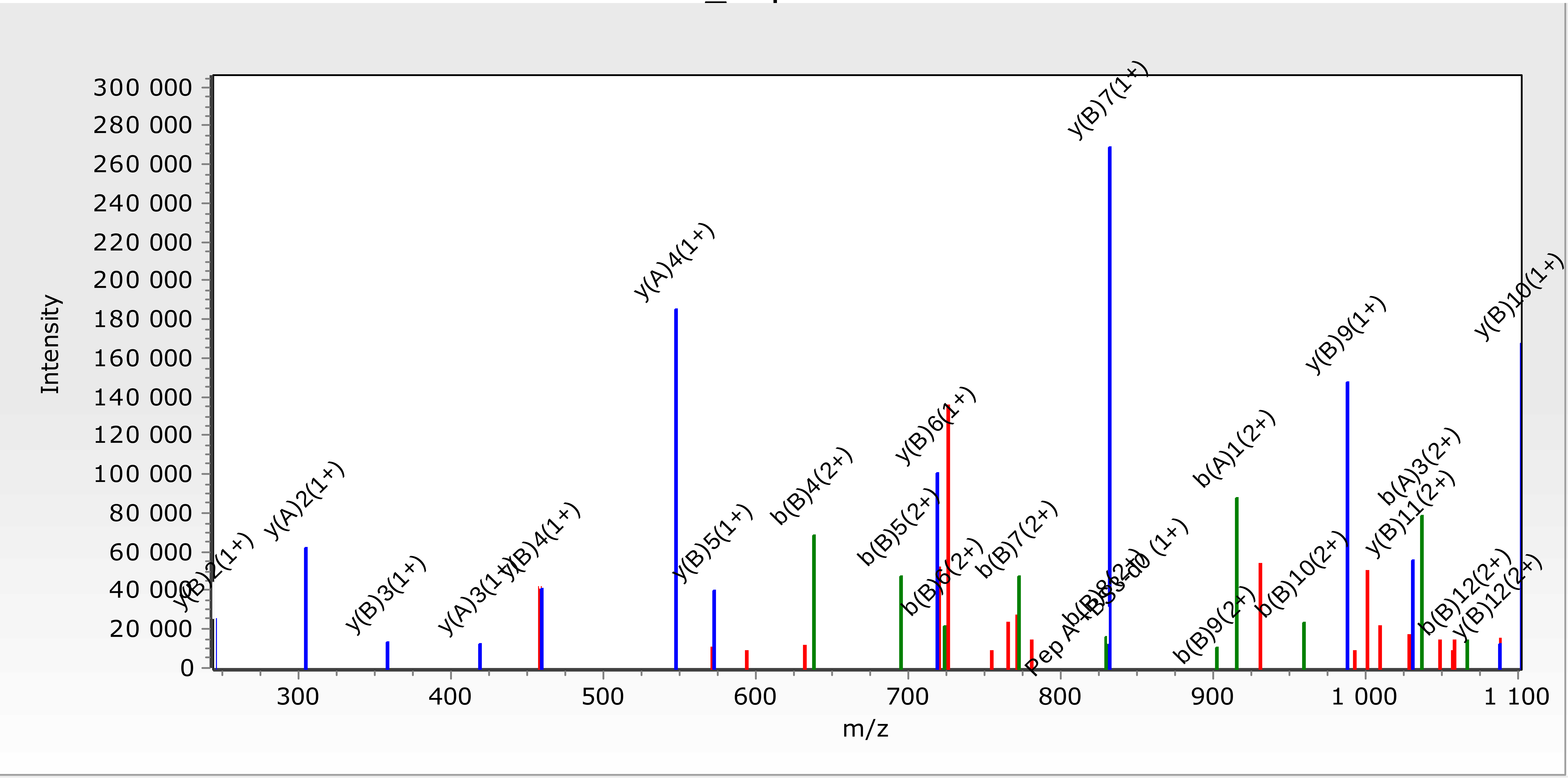

MSMS
MQDQRxAELKNGVLFITIPK 1x161 ^{14}Nx^{14}N
File: 150609_Hx1

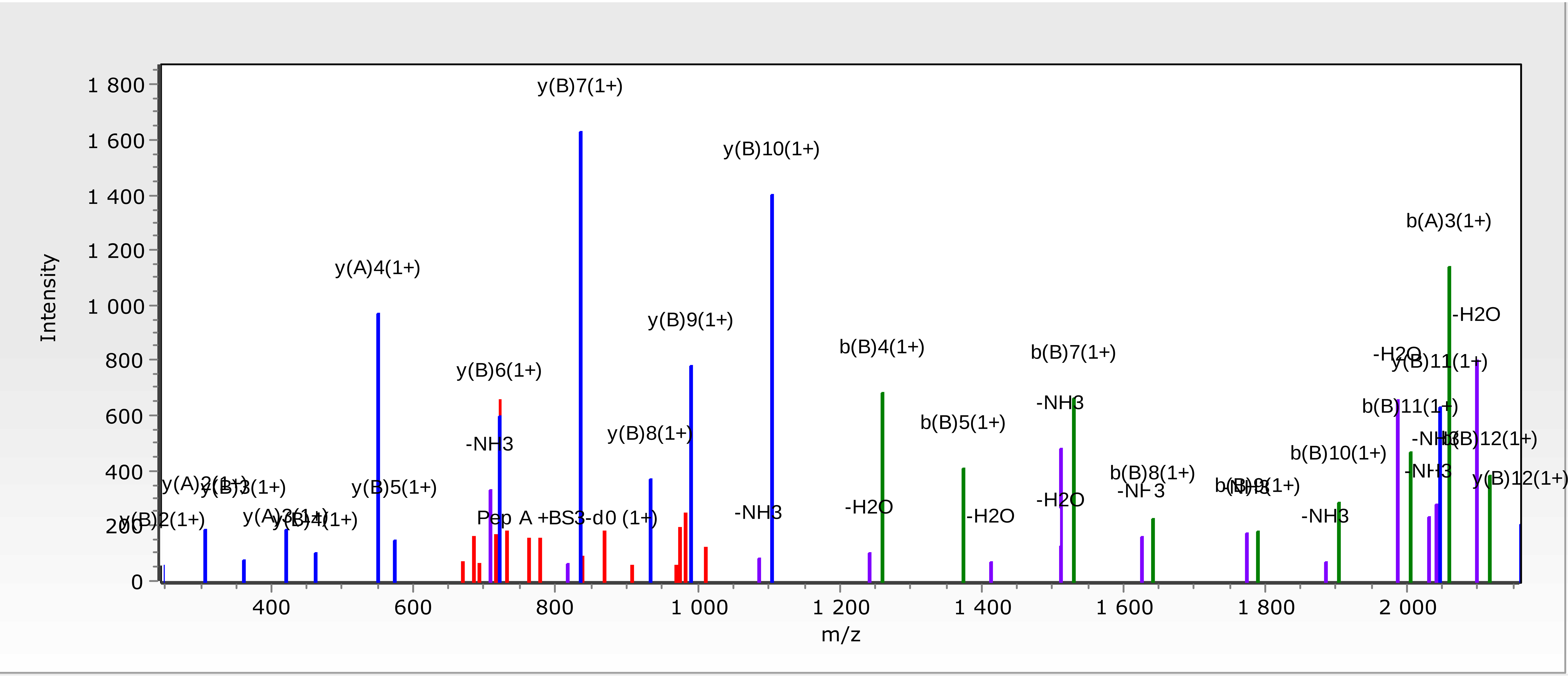

MS
MQDQRxAELKNGVLFITIPK 1x161
File: 150609_Hx1 rt: 1773

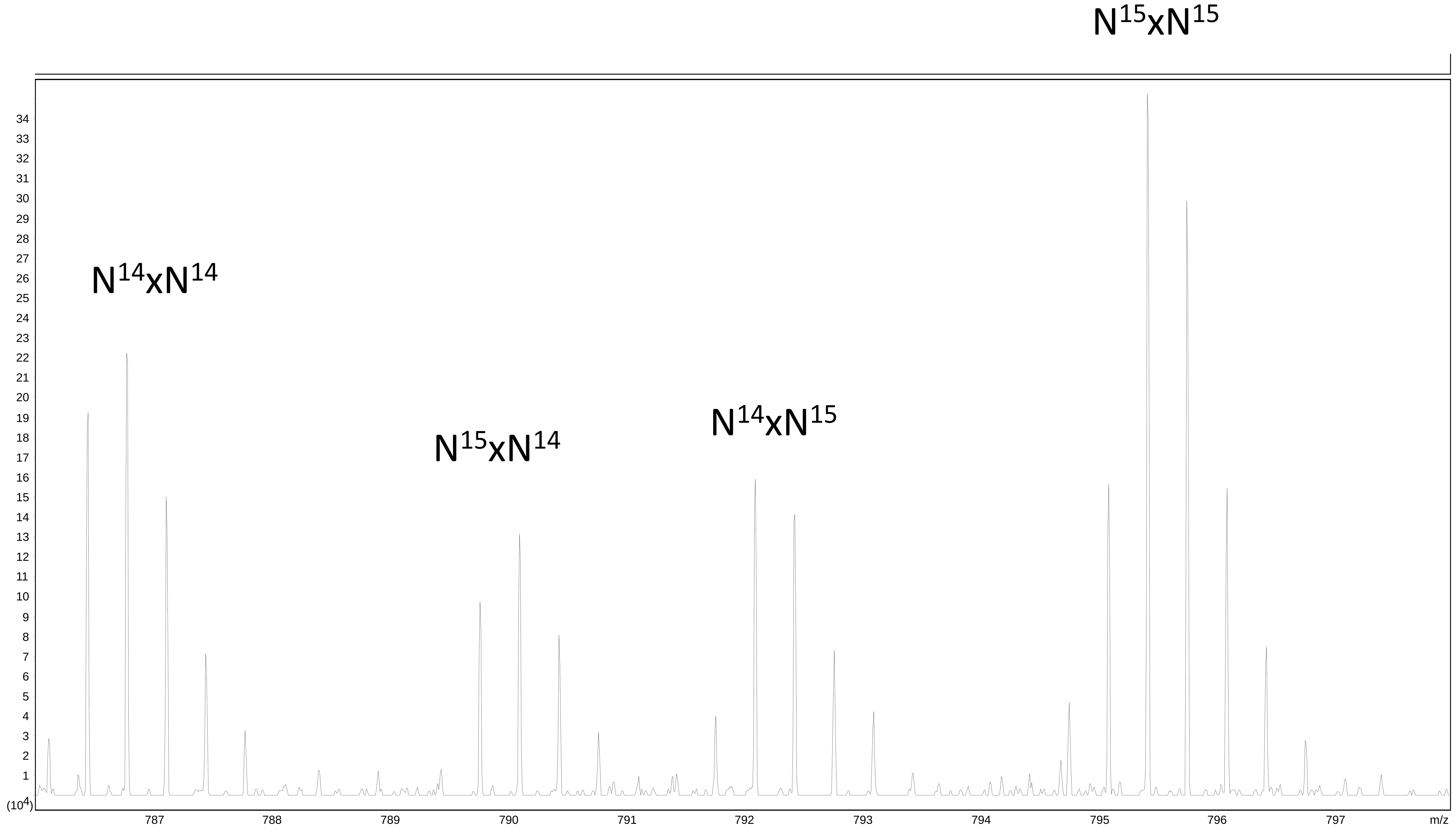

MSMS
MQDQRxTKVER 1x173 150521
File: 150521_Hsp21 monomeric band

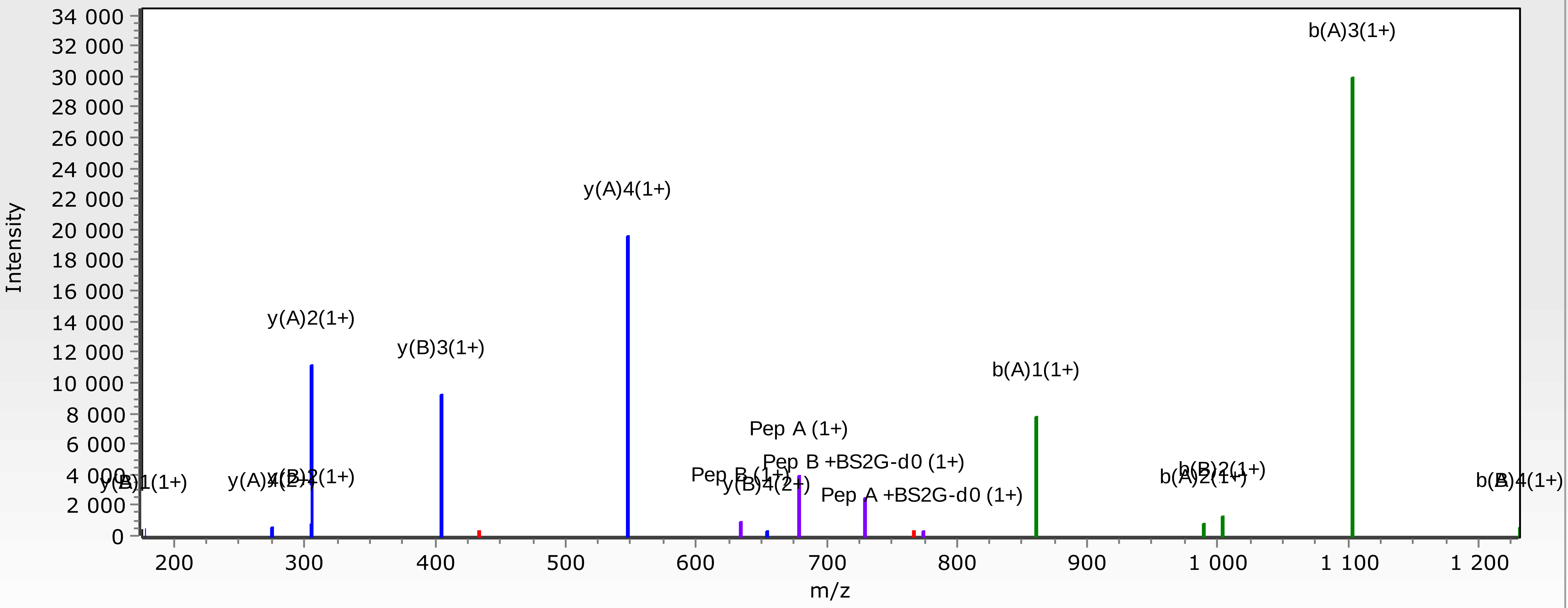

MSMS

MQDQRxTKVER 1x173 ^{14}Nx^{14}N

File: 150609_Hx1

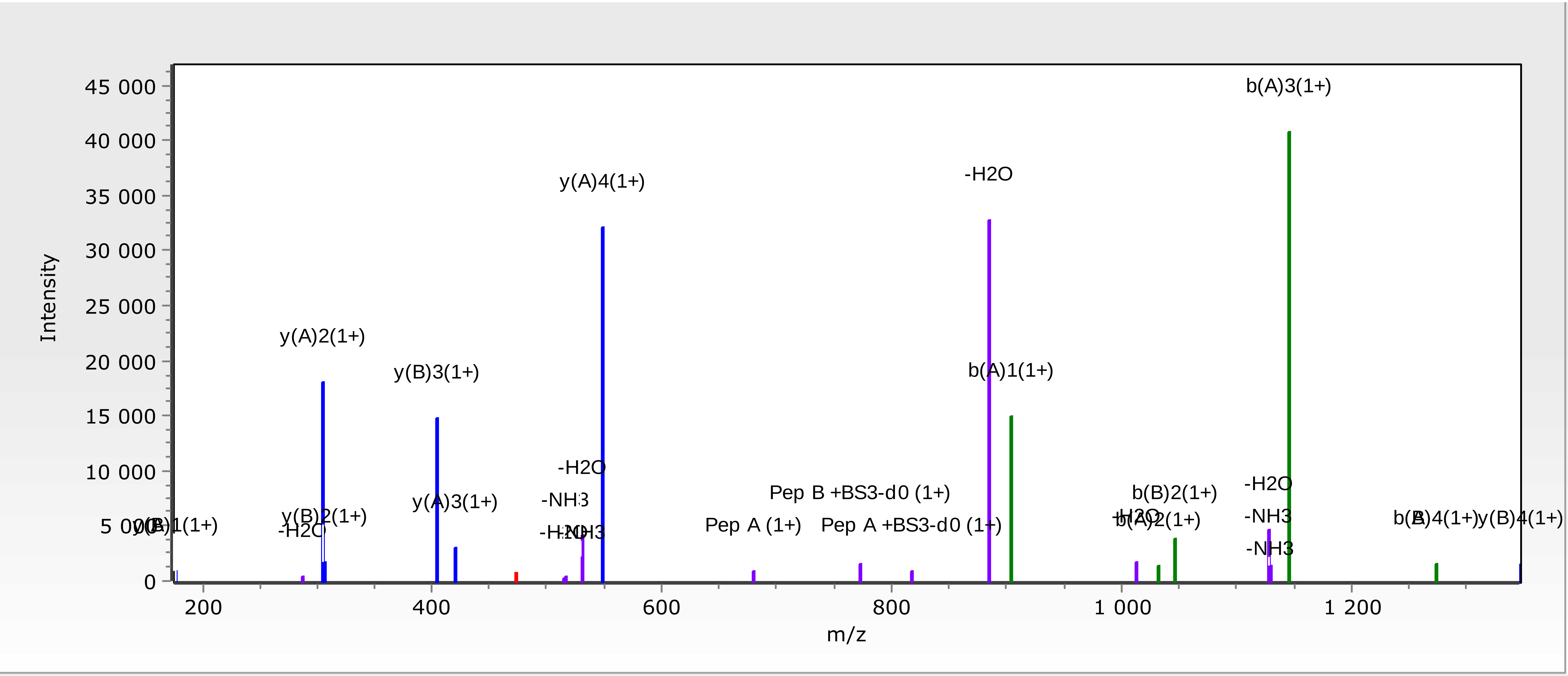

MS
MQDQRxTKVER 1x173
File: 150609_Hx1 rt: 837

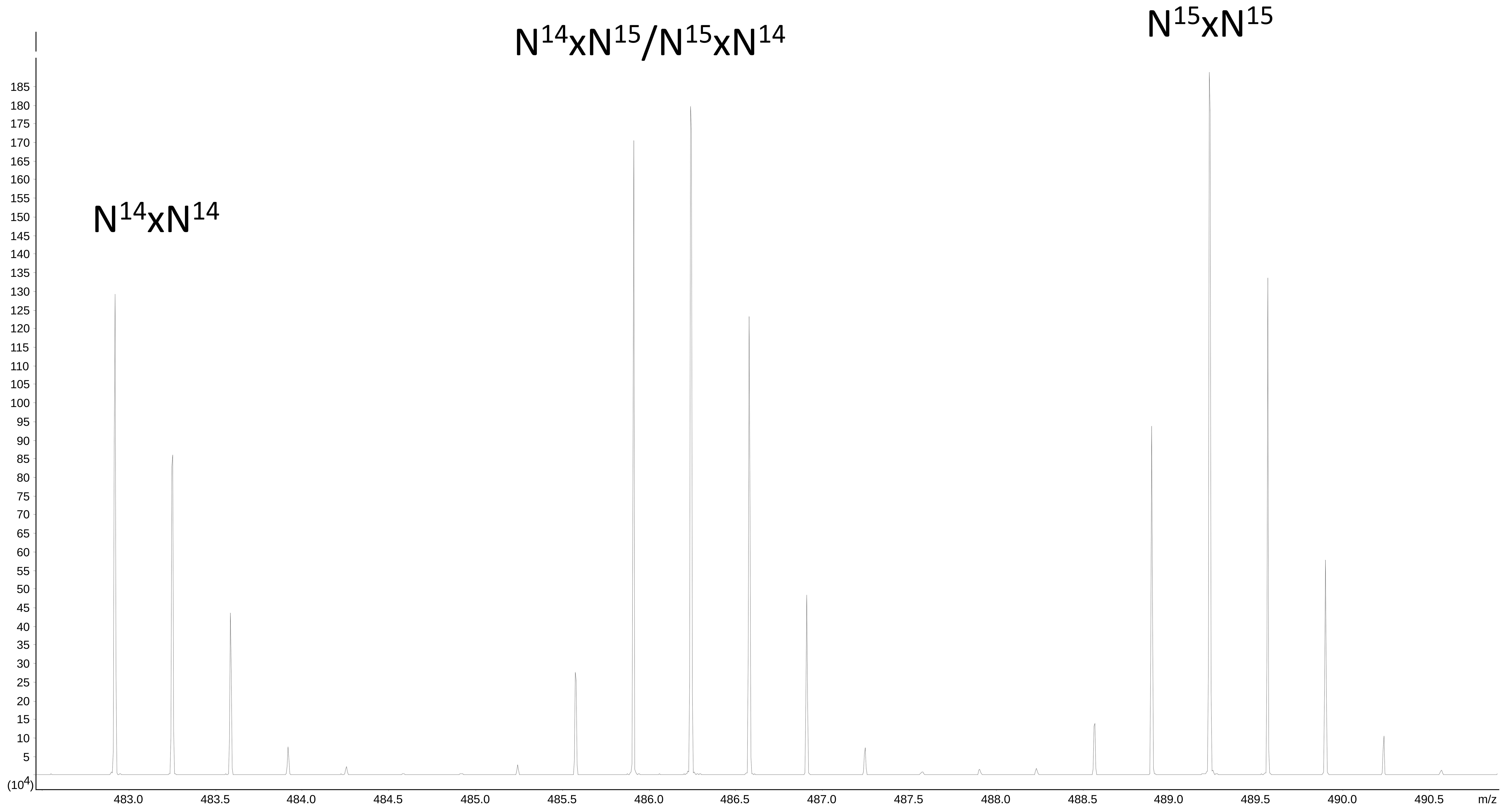

MSMS
ISVEDNVLVIKGEQKxGNQGSSVEKRPQQR 121x27 $N^{14}xN^{14}$
File: 150609_Hx5

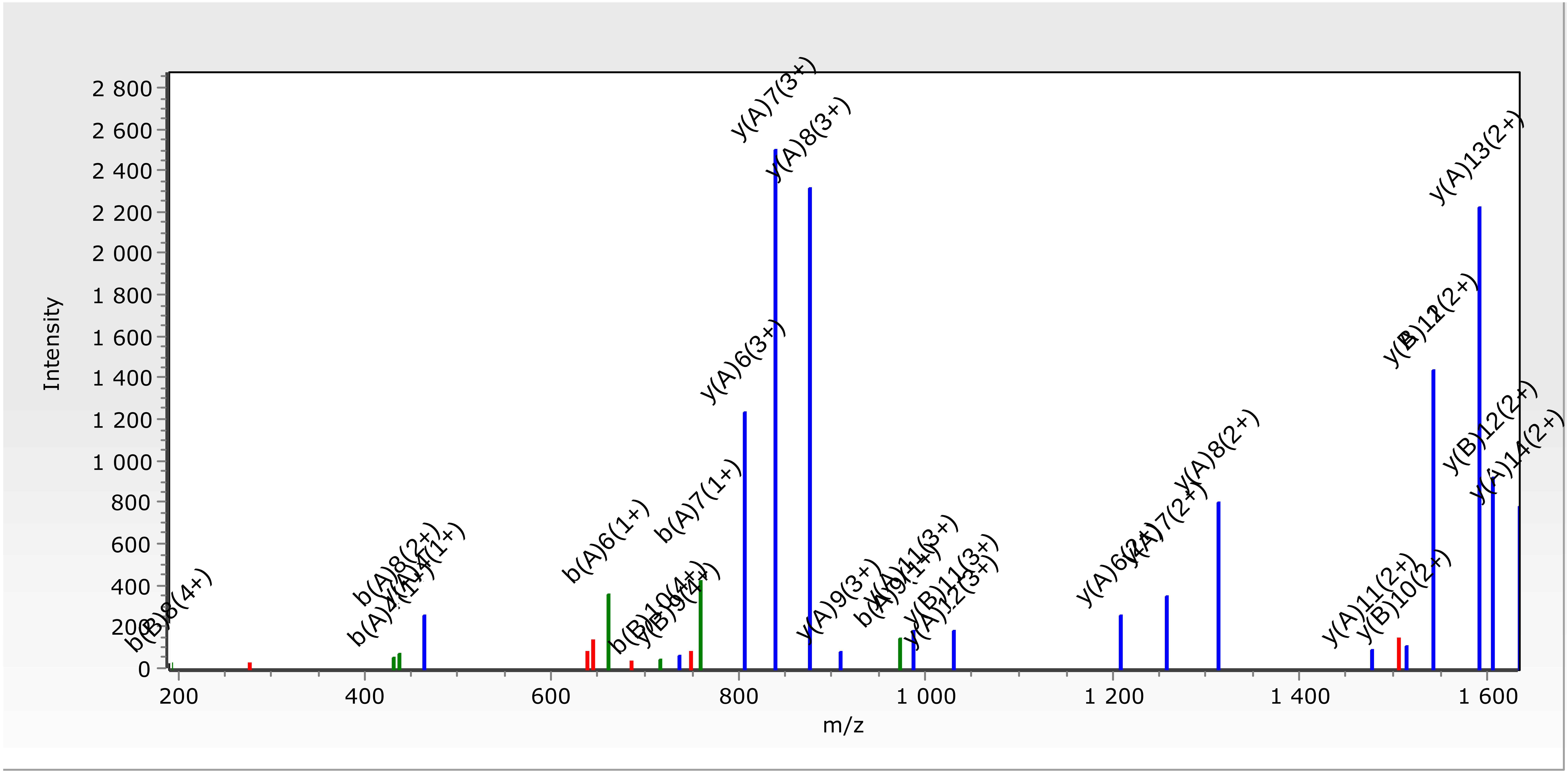

MS

ISVEDNVLVIKGEQKxGNQGSSVEKRPQQR 121x27

File: 150609_Hx5 rt: 1291

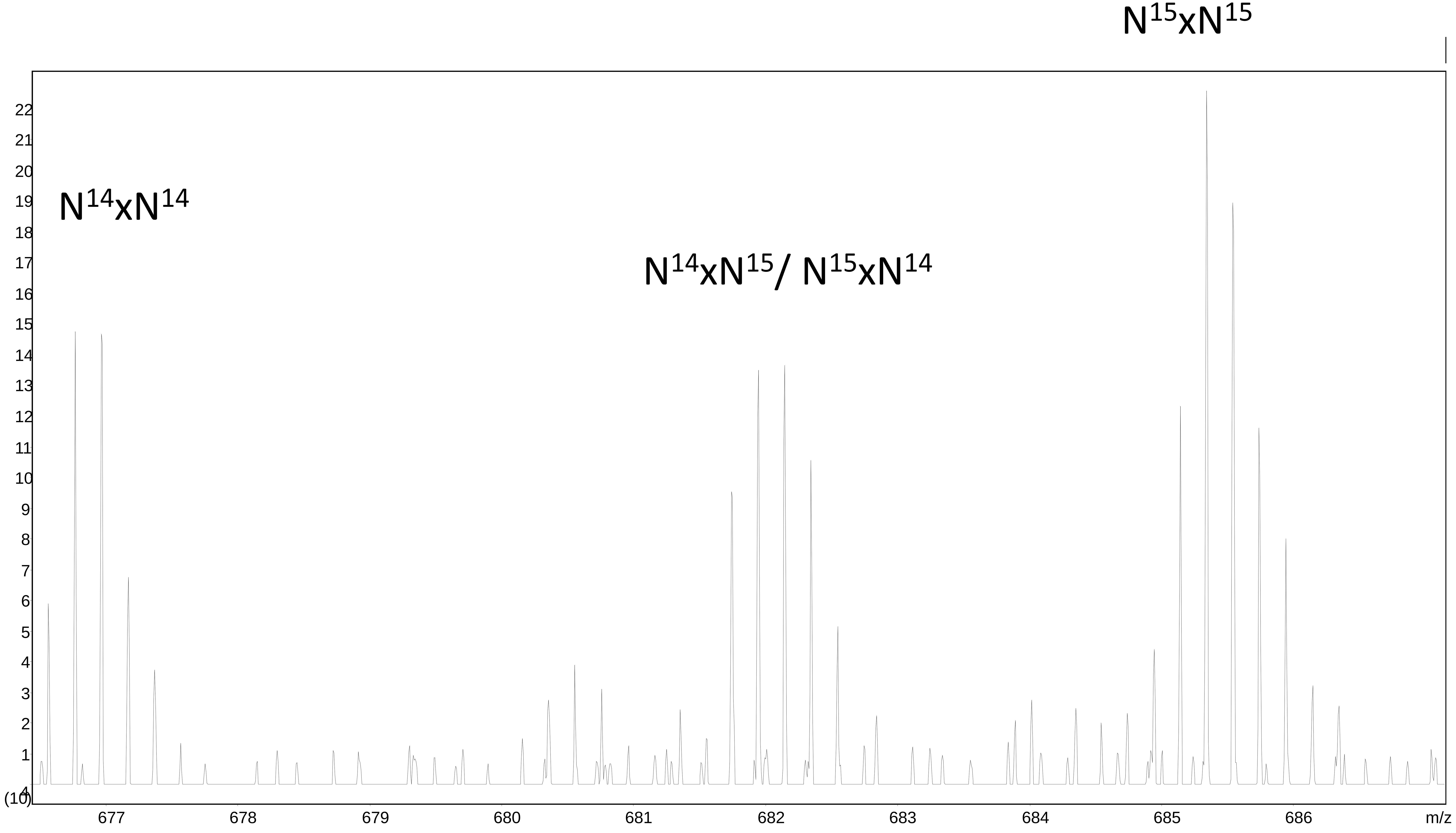

MSMS
APWDIKEEEHEIKxGEQKKEDSDDSWSGR 89x125
File: 150521_Hsp21 monomeric band

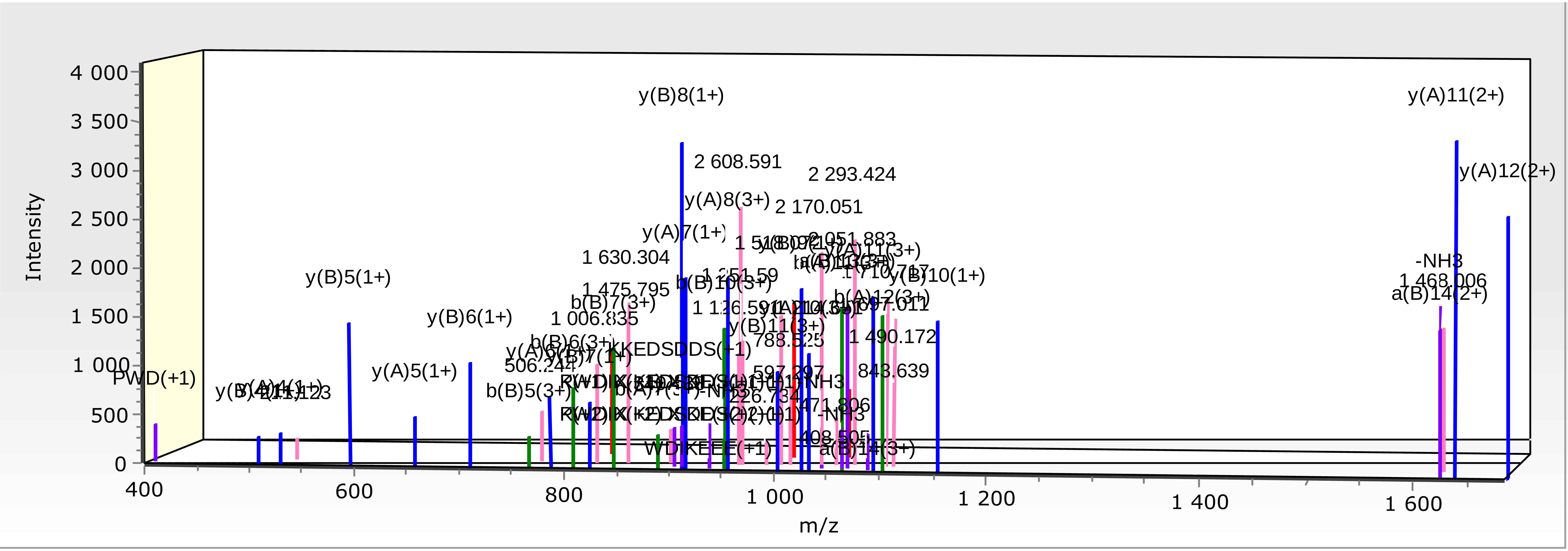

MSMS
KEDSDDSWSGRxAPWDIKEEEHEIKMR 126x89
File: 150521_Hsp21 monomeric band

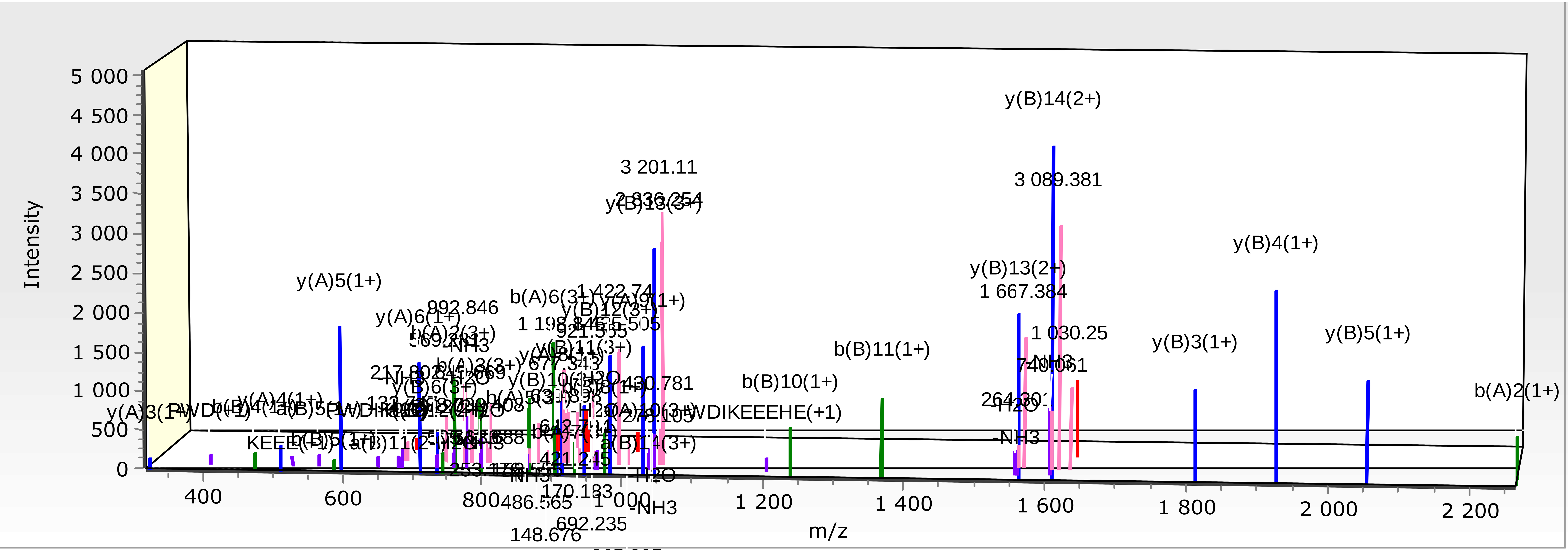

MSMS
APWDIKEEEHEIKMRxKEDSDDSWSGR 126x89 $N^{14}xN^{14}$
File: Hx5

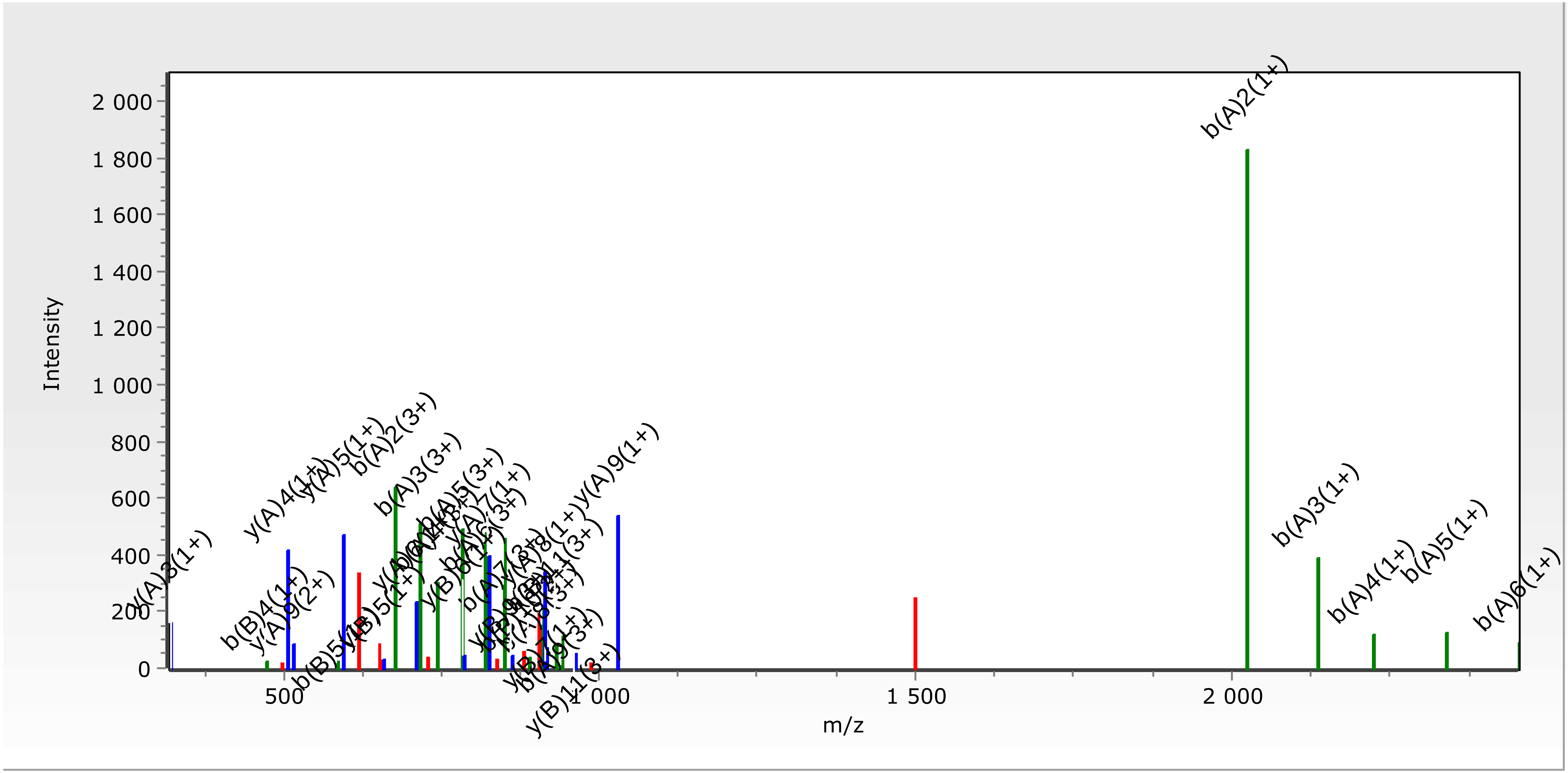

MSMS

APWDIKEEEHEIKMRxKEDSDDSWSGR 126x89 $N^{15}xN^{14}$

File: 150609_Hx5

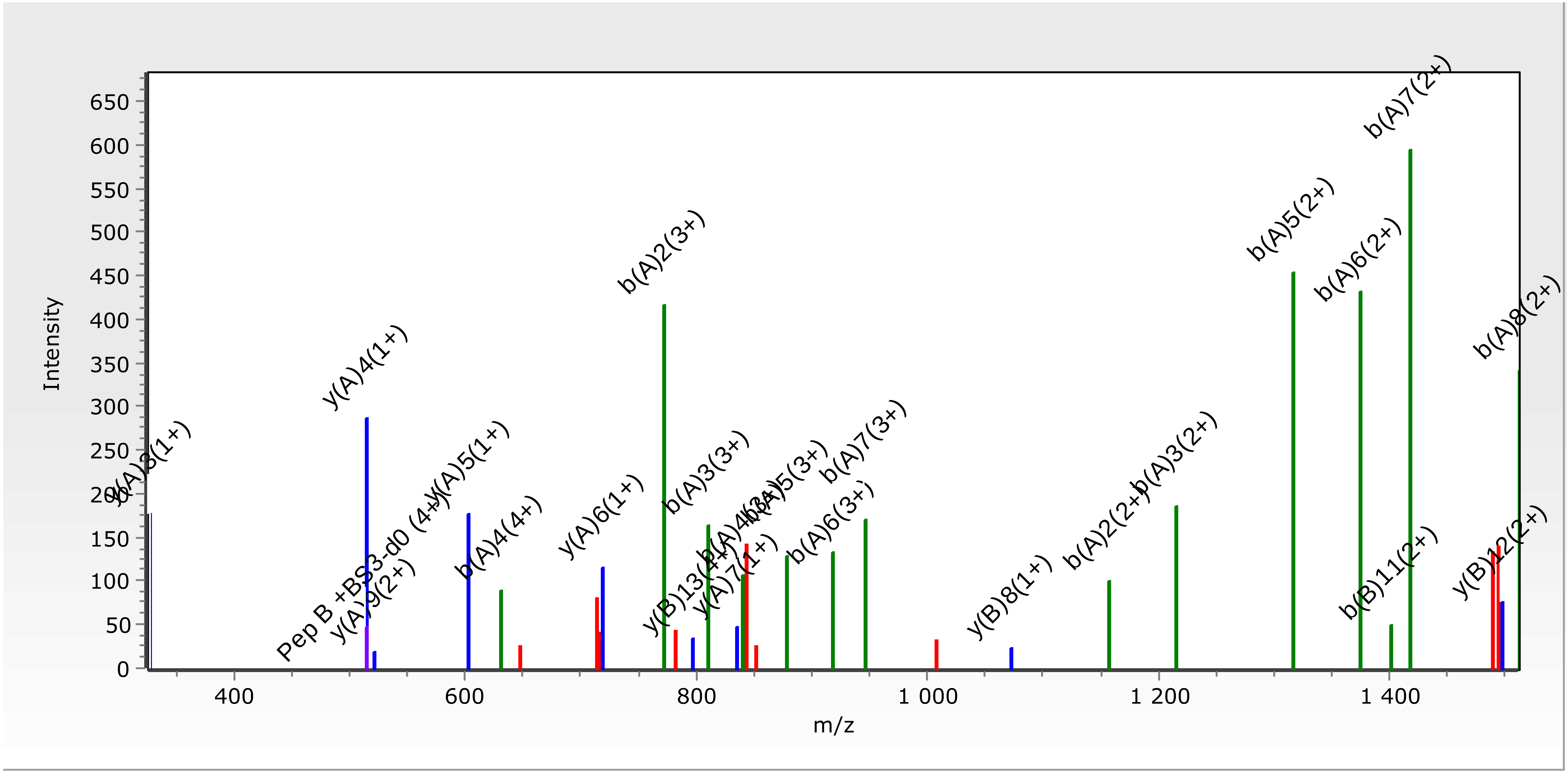

MS
APWDIKEEEHEIKMRxKEDSDDSWSGR 126x89
File: 150609_Hx5 rt: 1382

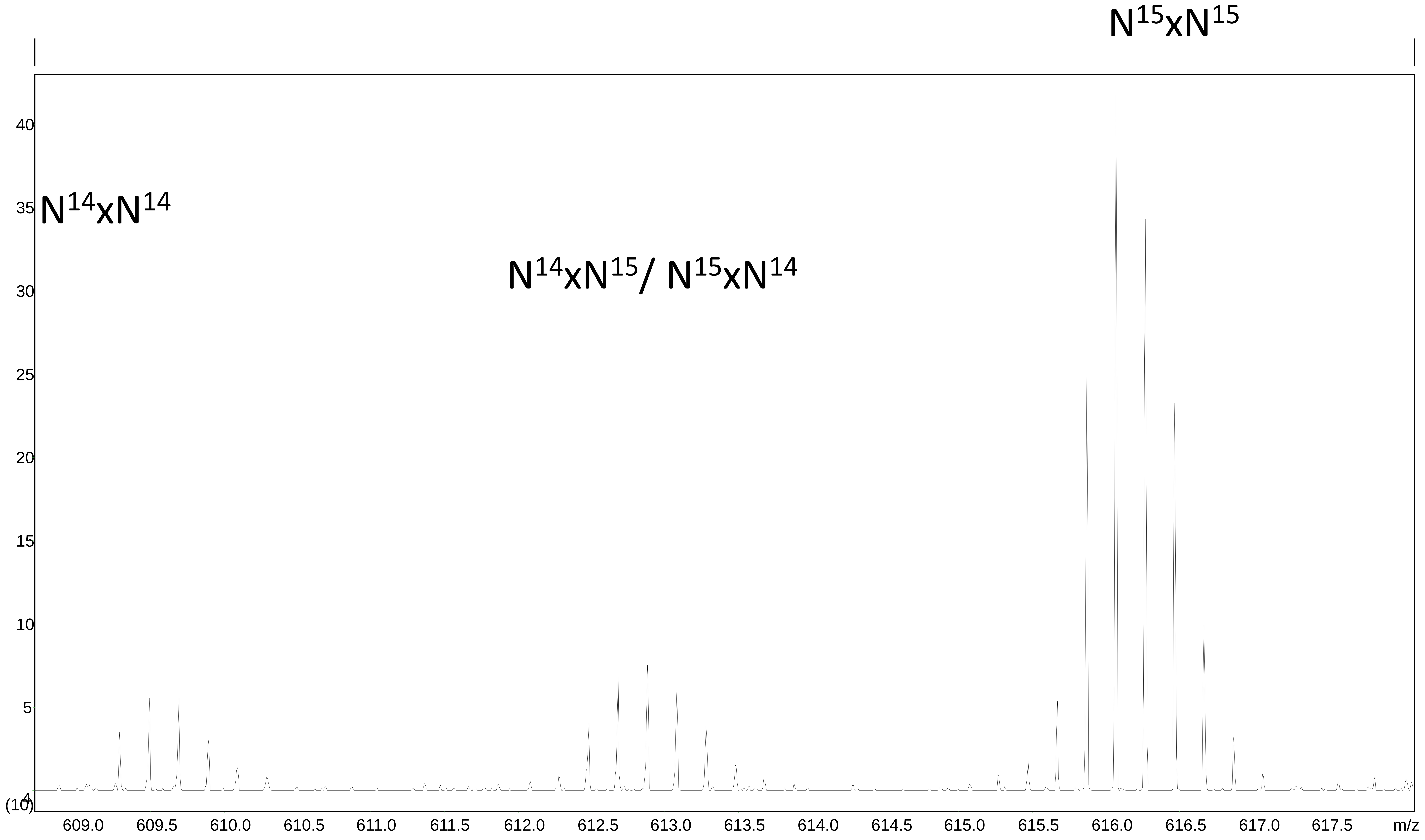

MSMS

APWDIKEEEHEIKxTKVER 89x173 ^{15}Nx^{15}N

File: 150609_Hx1

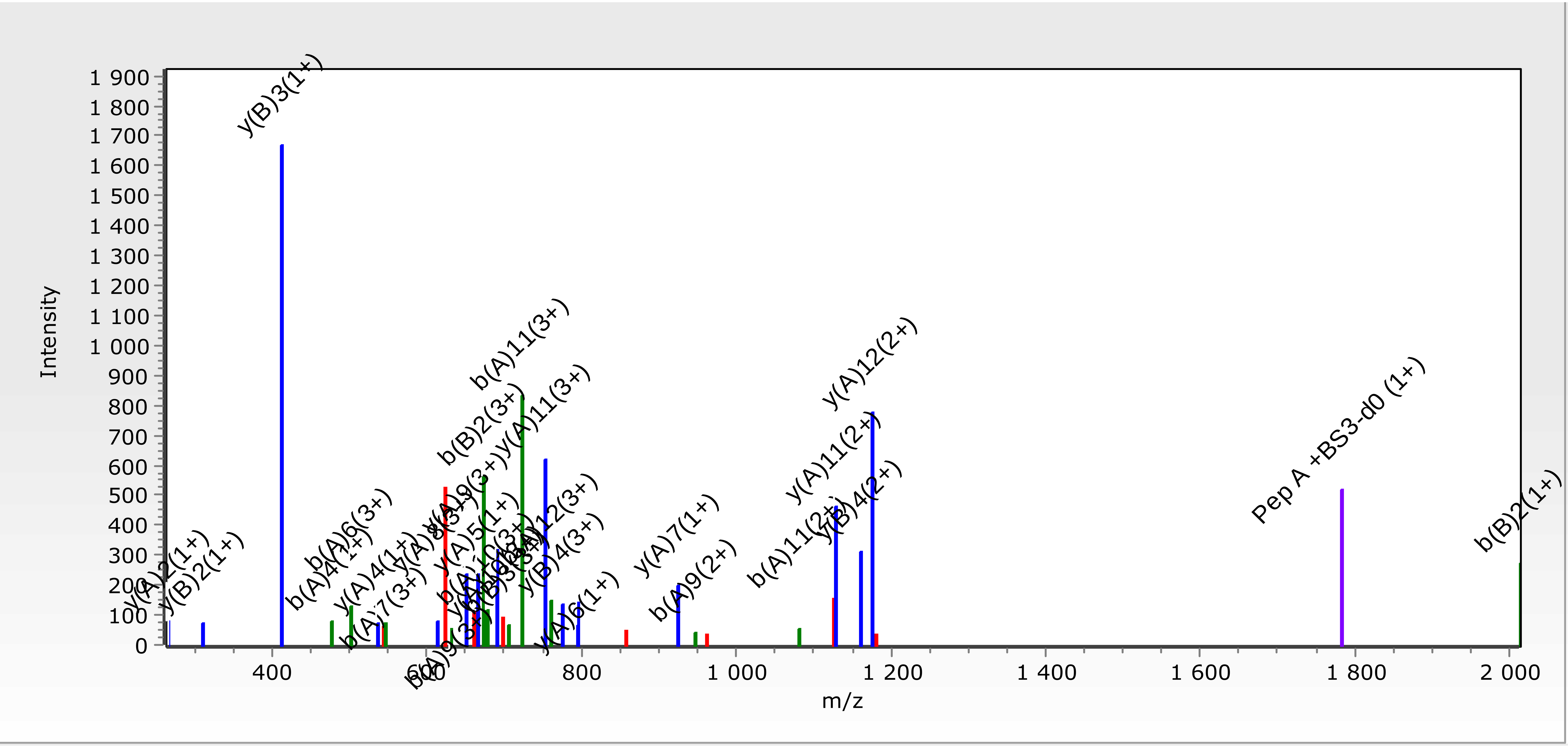

MS
APWDIKEEEHEIKxTKVER 89x173
File: 150609_Hx1 rt: 1242

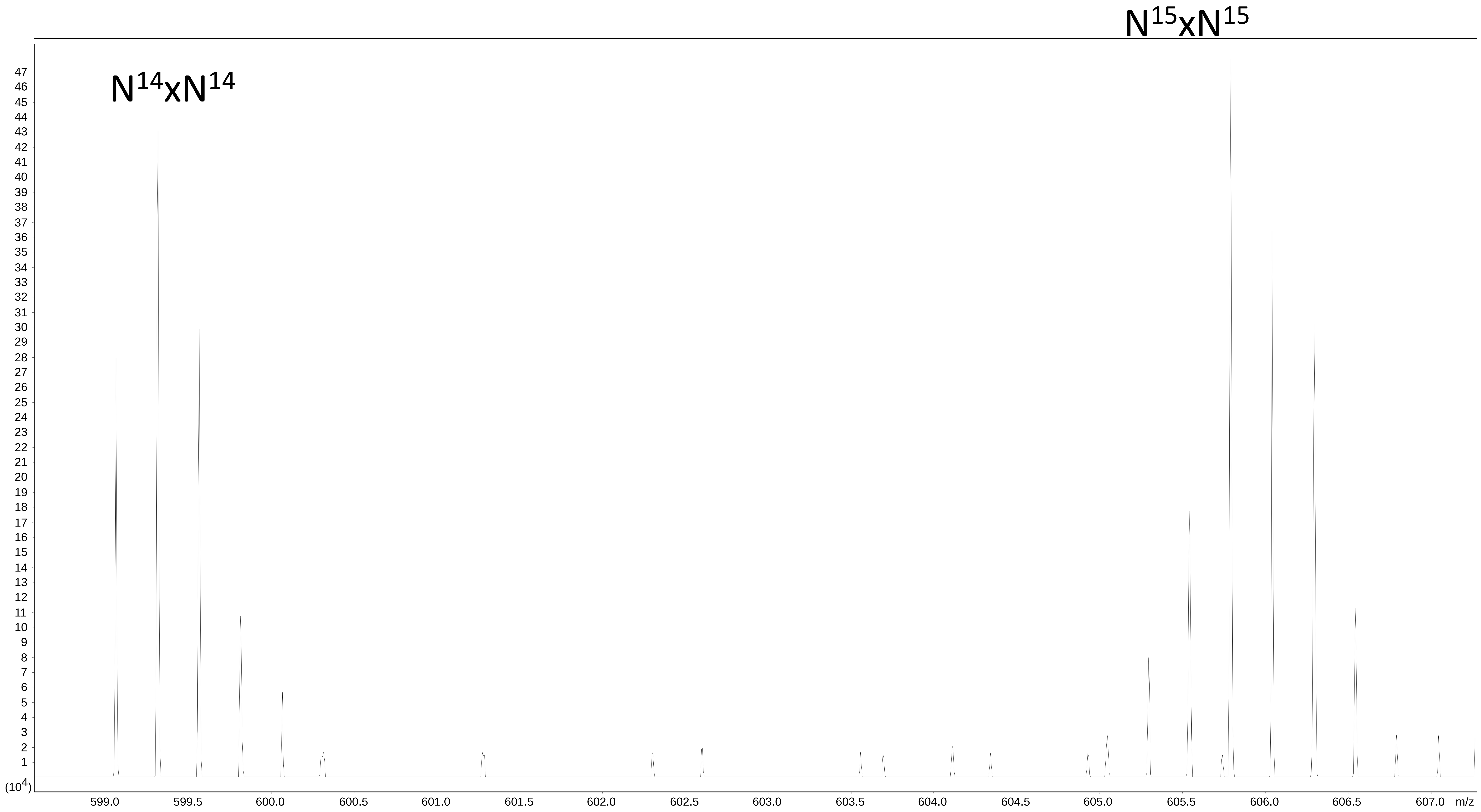

MSMS
KEDSDDSWSGRxAPWDIKEEEHEIKMR 126x96
File: 150521_Hsp21 monomeric band

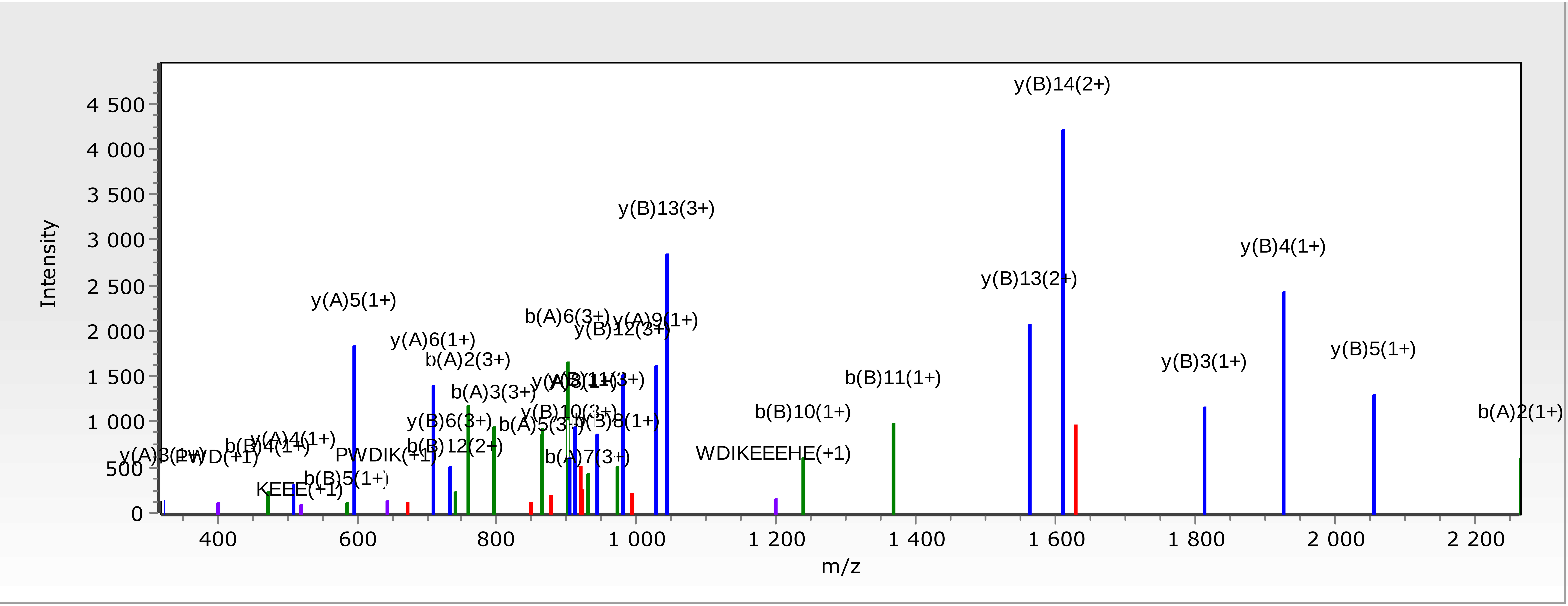

MSMS
AELKNGVLFITIPKxFDMPGLSKEDVK 161x106
File: 150521_Hsp21 monomeric band

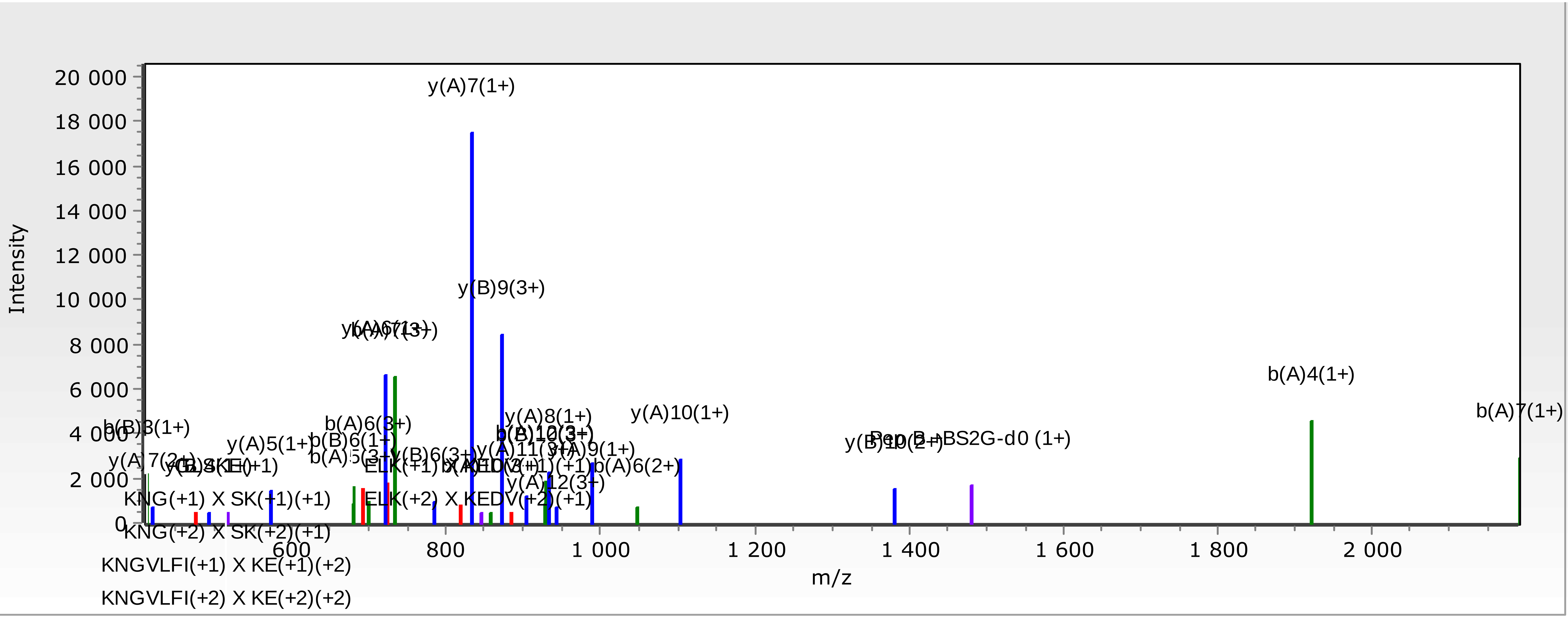

MSMS

FDMPGLSKEDVKxAELKNGVLFITIPK 106x161 ^{14}Nx^{14}N

File: 150609_Hx1

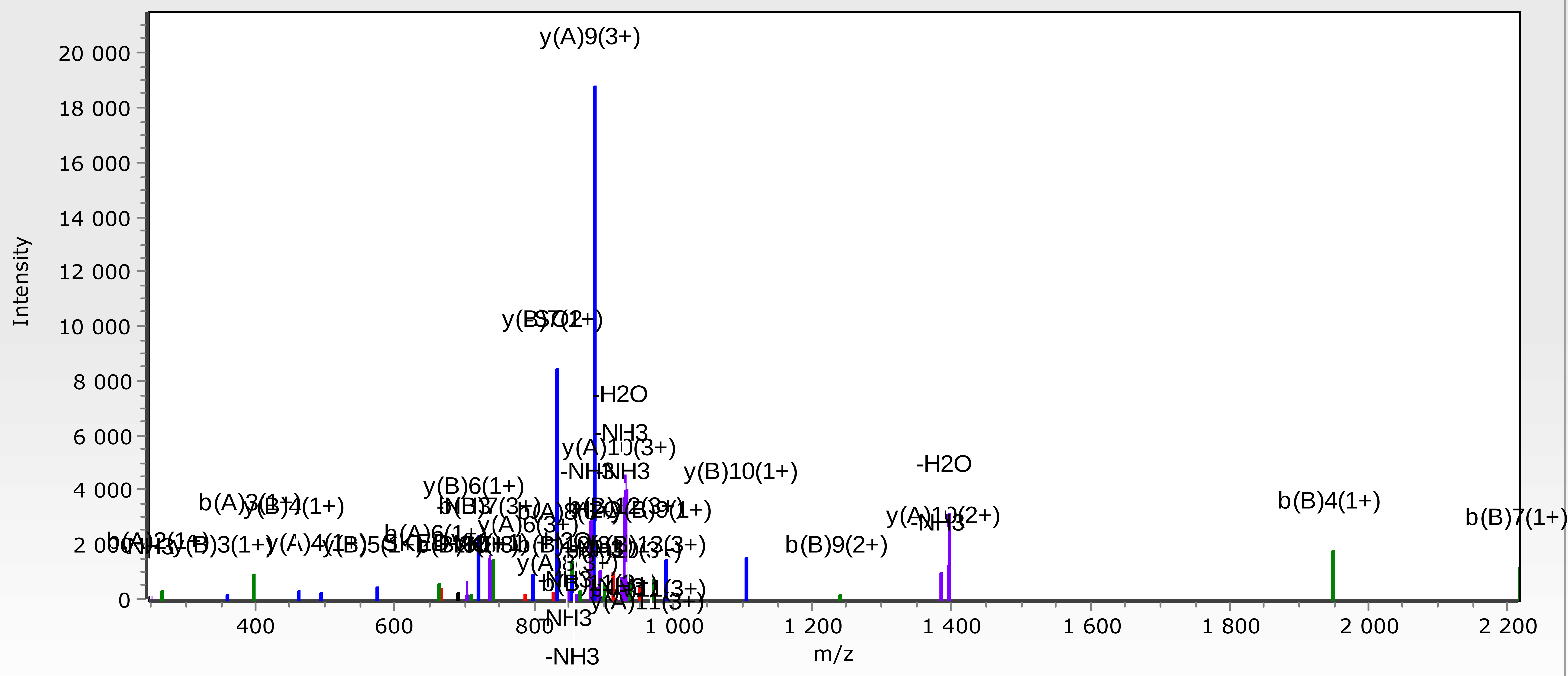

MS

FDMPGLSKEDVKxAELKNGVLFITIPK 106x161

File: 150609_Hx1 rt: 1874

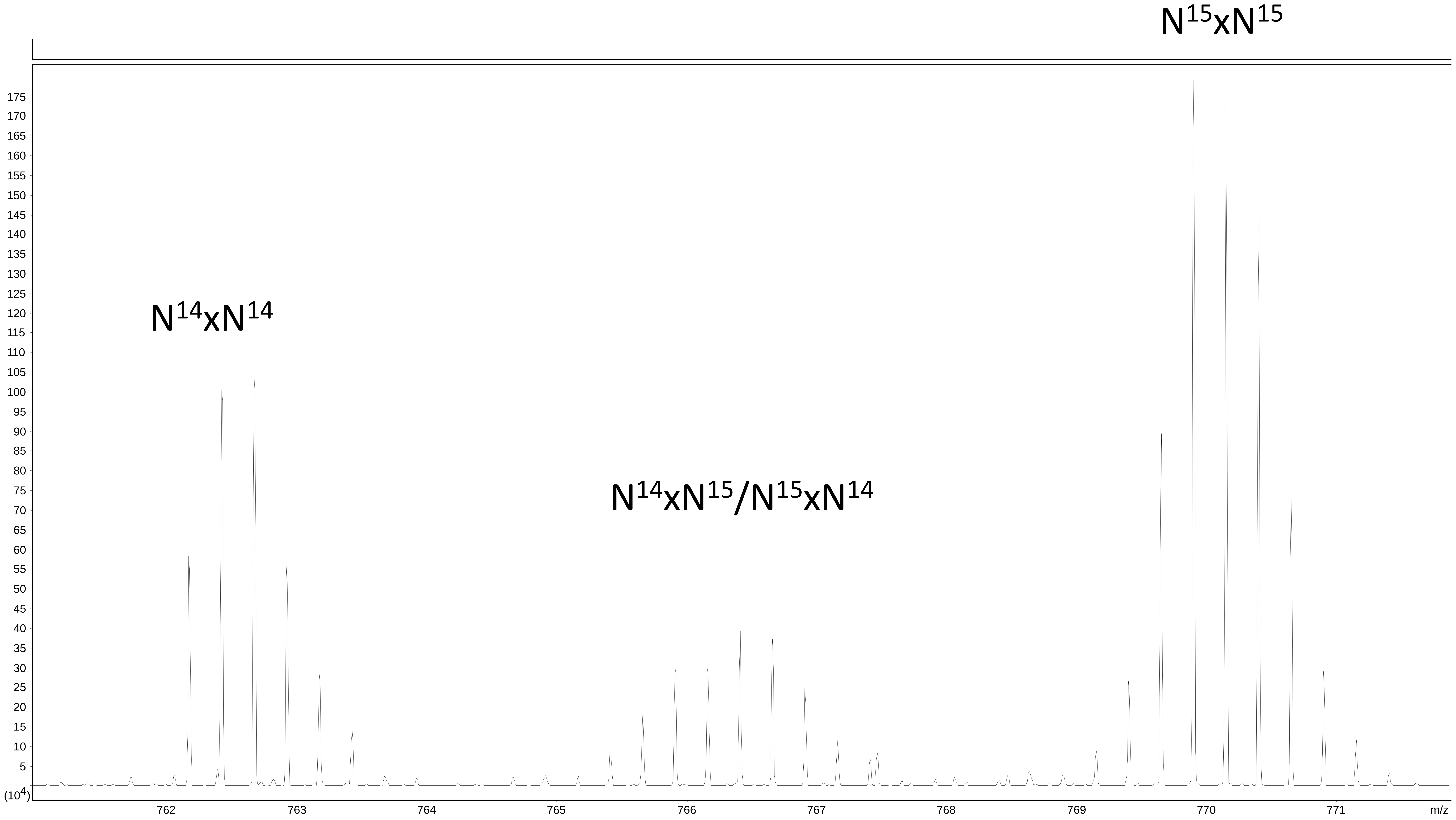

MSMS
ISVEDNVLVIKGEQKxKEDSDDSWSGR 121x126
File: 150521_Hsp21 monomeric band

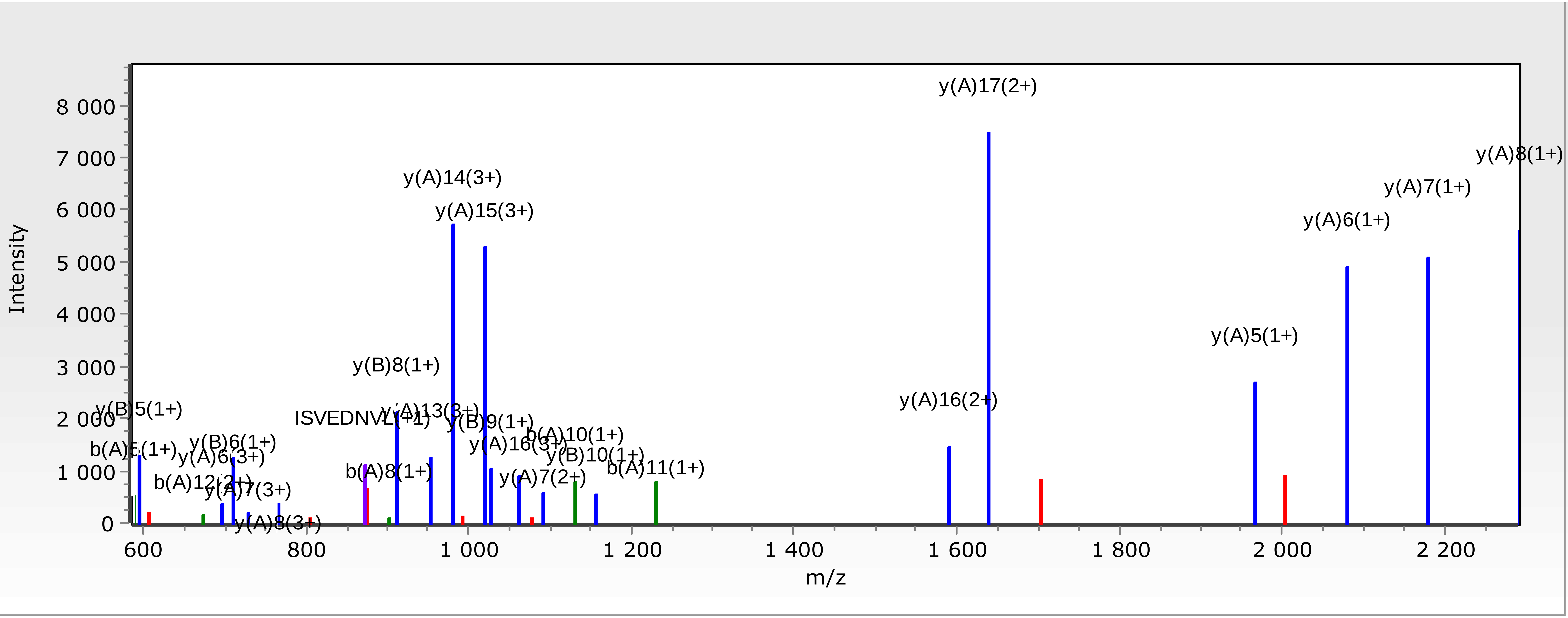

MSMS
ISVEDNVLVIKGEQKxKEDSDDSWSGR 121x126 ^{14}Nx^{14}N
File: 150609_Hx1

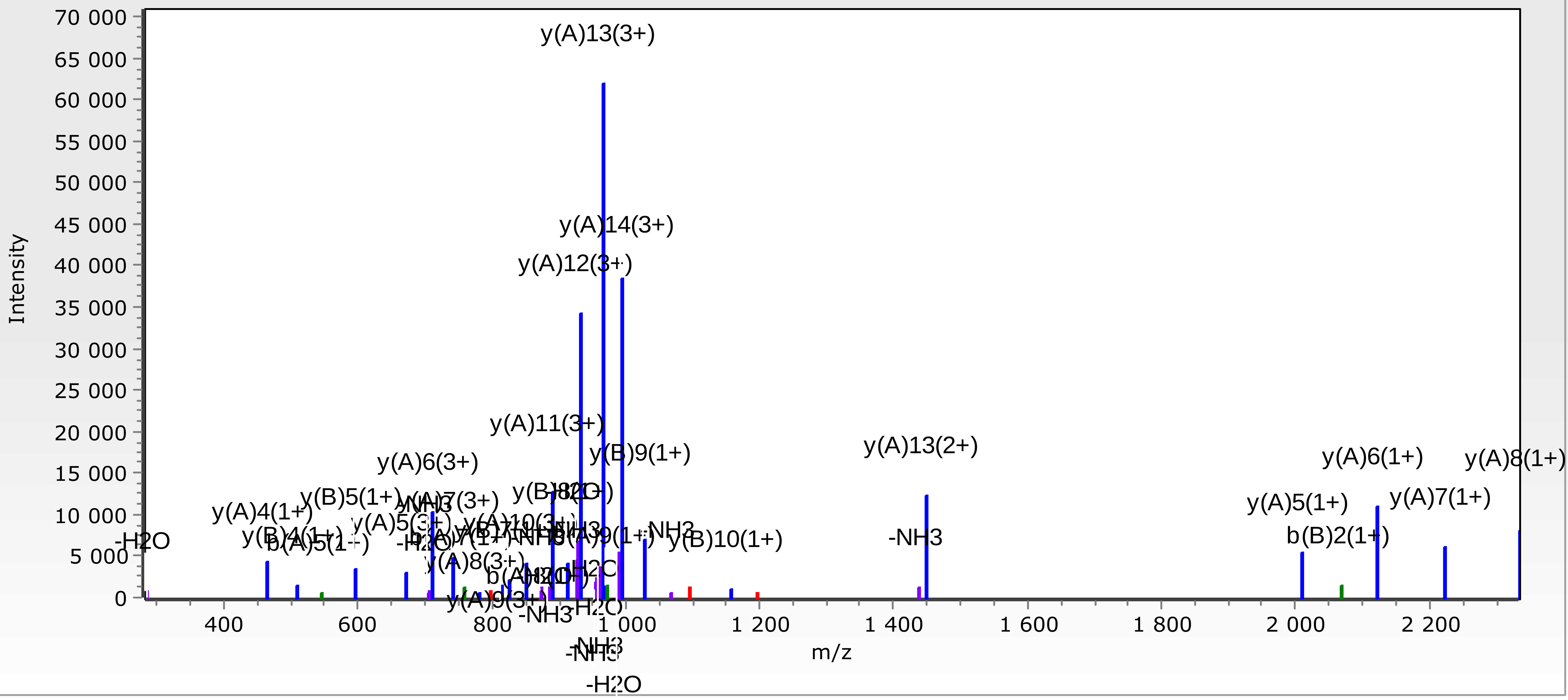

MS
ISVEDNVLVIKGEQKxKEDSDDSWSGR 121x126
File: 150609_Hx1 rt: 1535

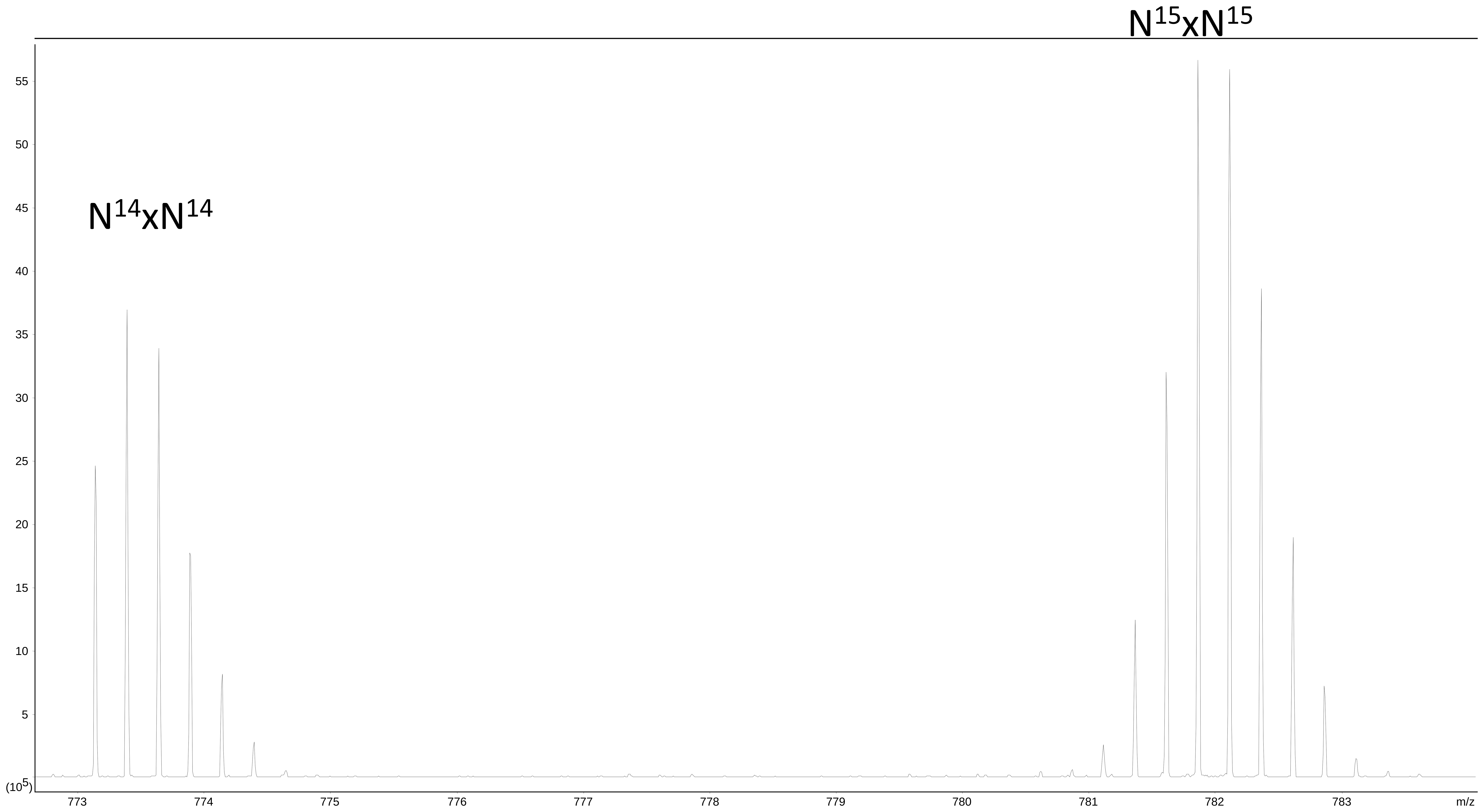

MSMS

ISVEDNVLVIKGEQKxTKVER 121x173 $N^{14}xN^{15}$

File: 150609_Hx4

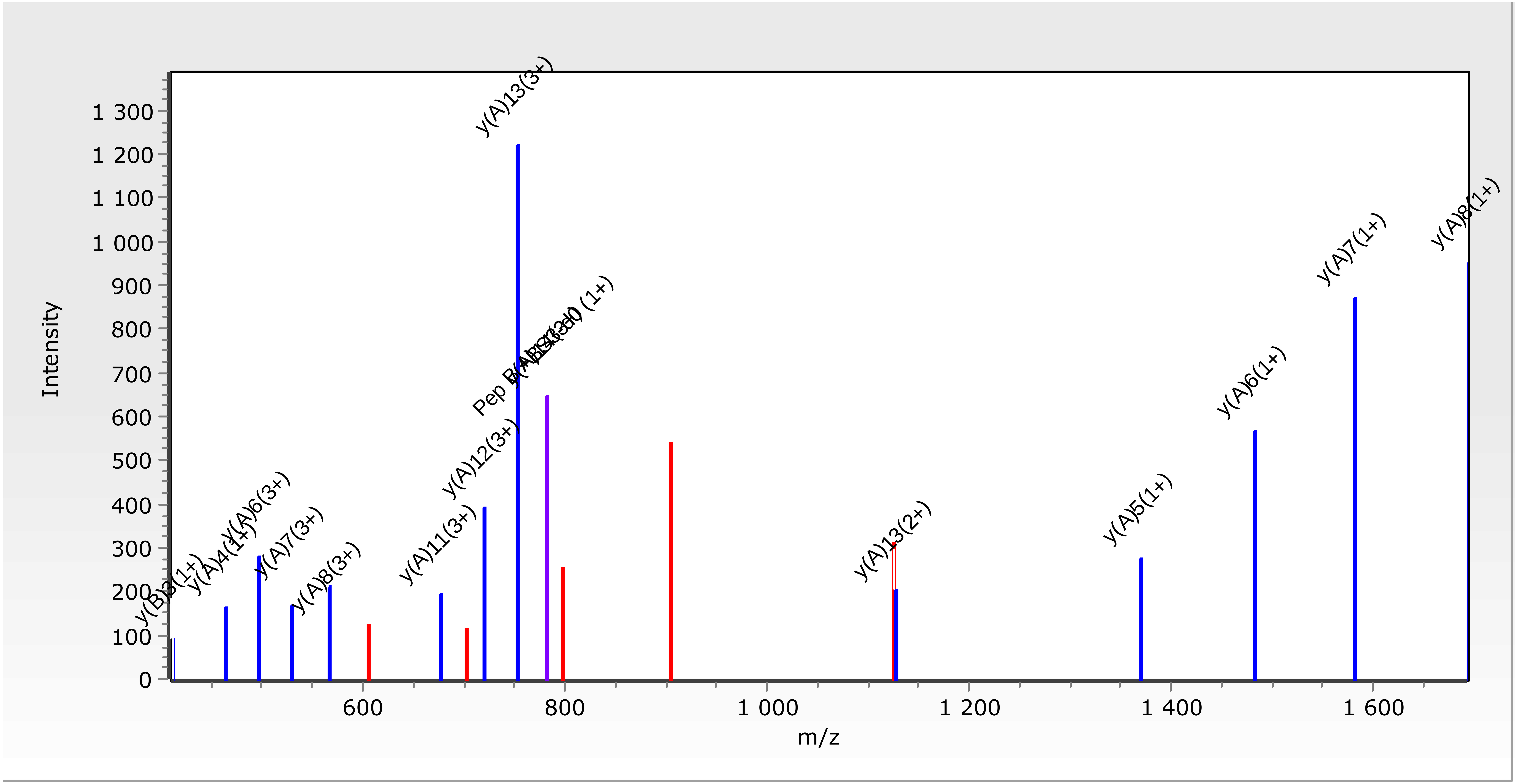

MS

ISVEDNVLVIKGEQKxTKVER 121x173

File: 150609_Hx4 rt: 1359

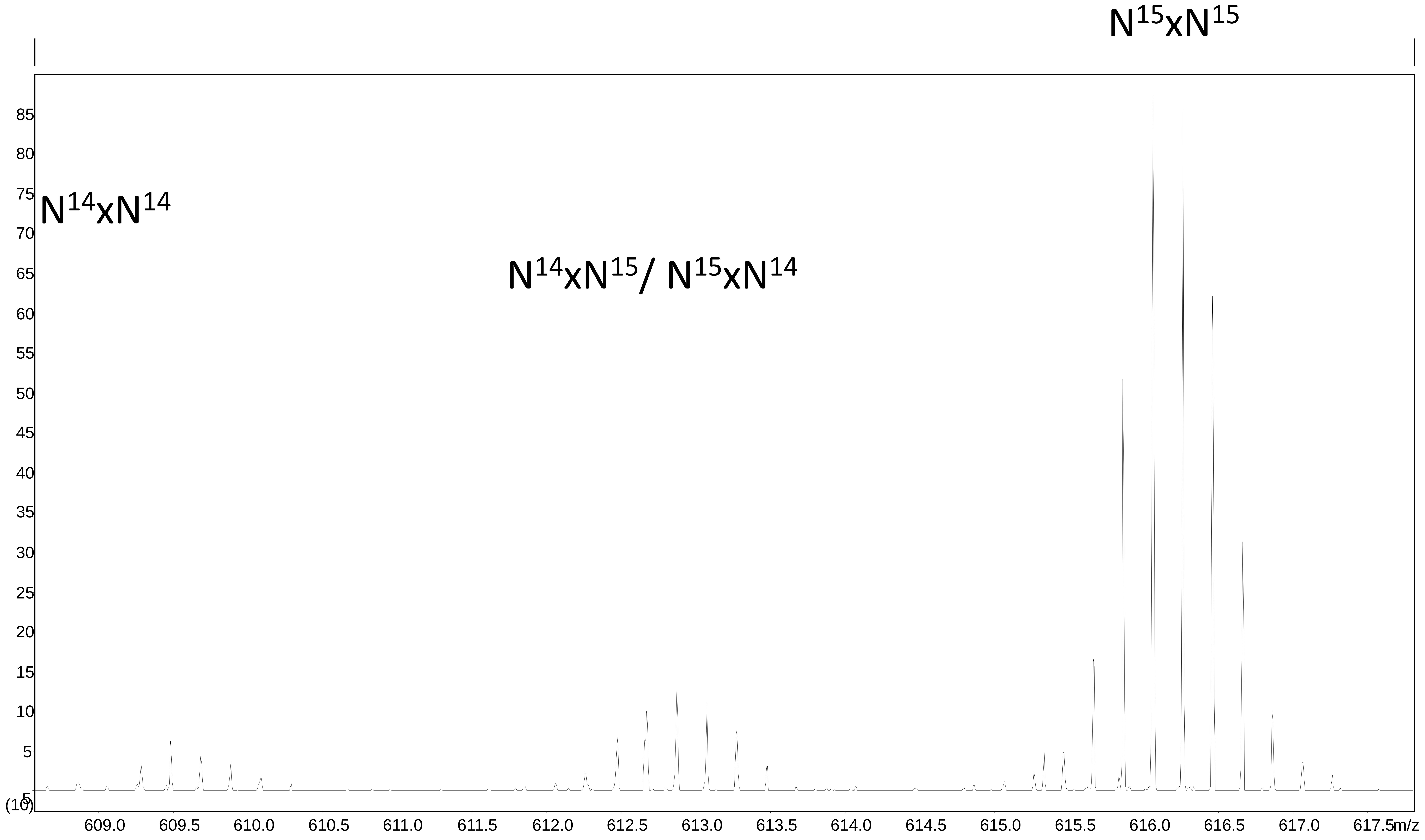

MSMS

AELKNGVLFITIPKxGEQKKEDSDDSWSGR 161x125

File: 160307_Hsp21 dimeric band

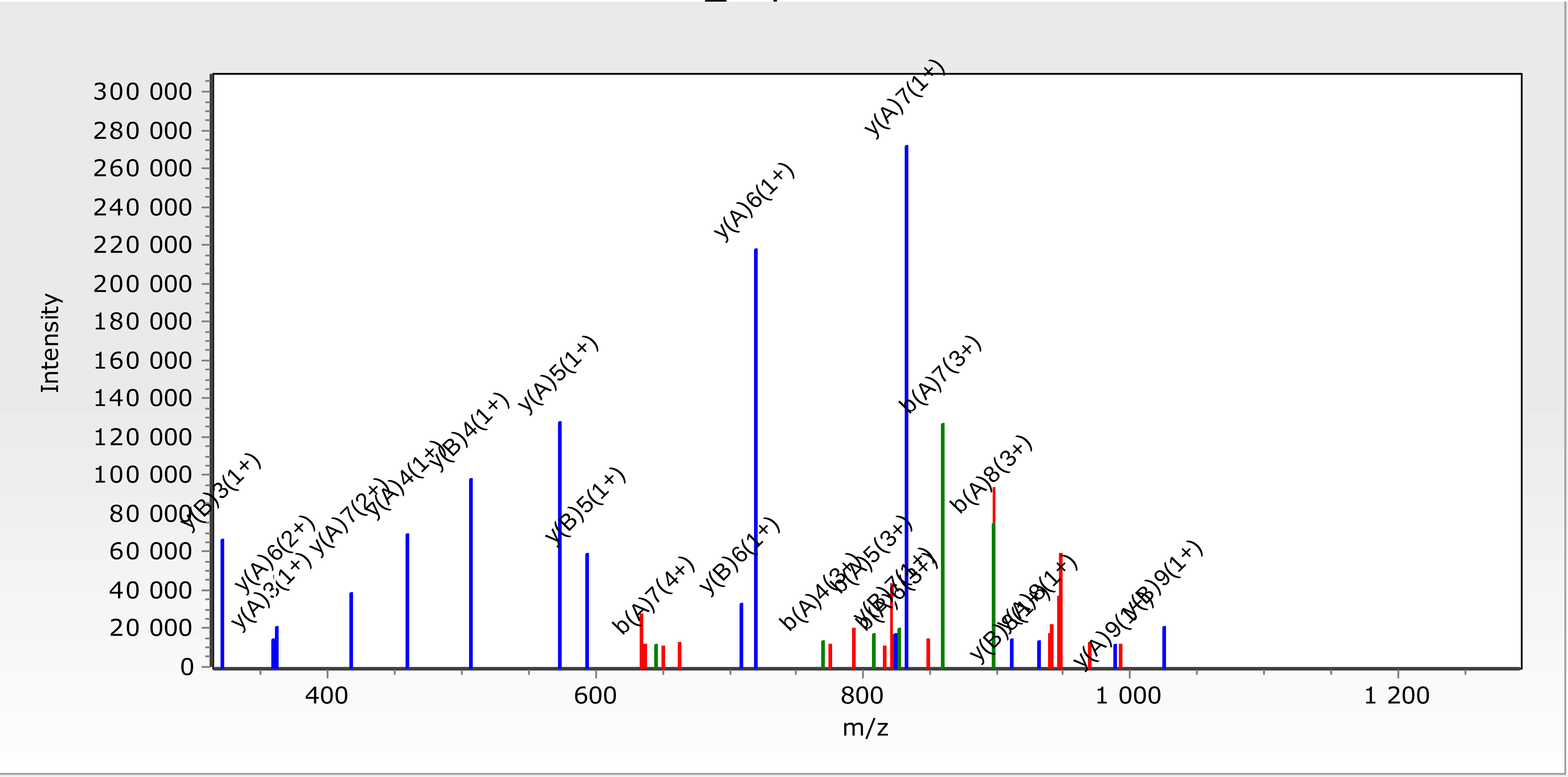

MSMS
KEDSDDSWSGRxIKAELKNGVLFITIPK 126x157
File: 150521_Hsp21 monomeric band

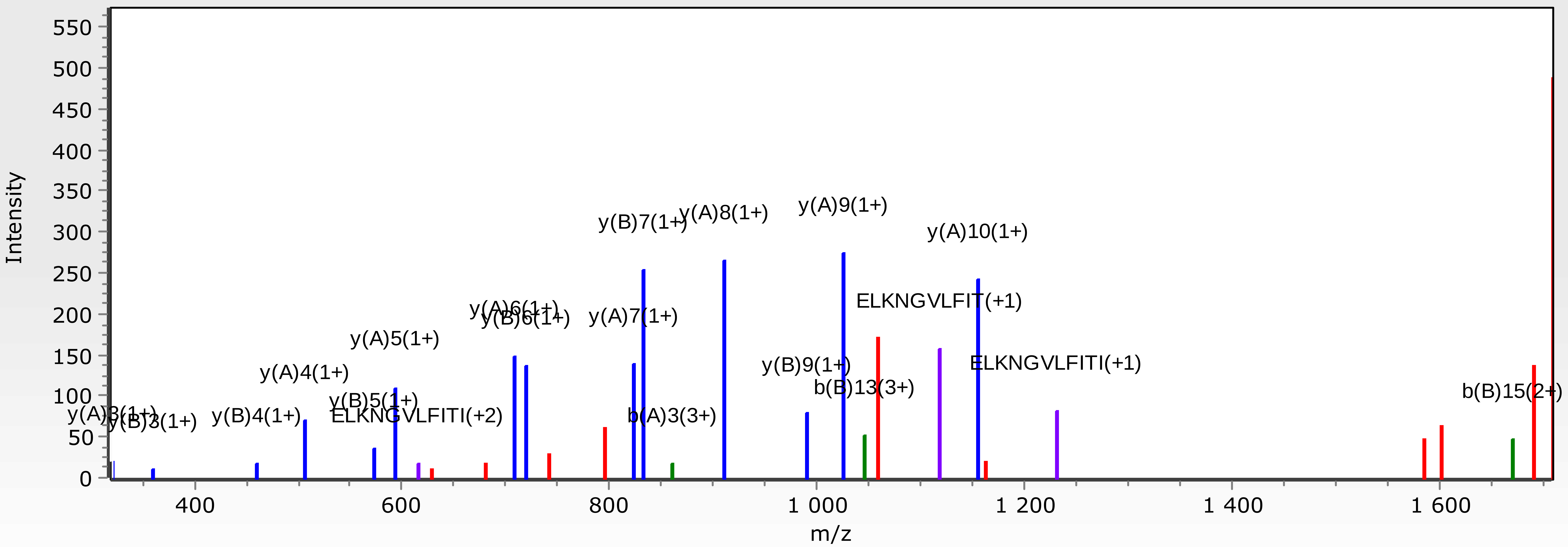

MSMS
KEDSDDSWSGRxAELKNGVLFITIPK 126x161
File: 160307_Hsp21 dimeric band

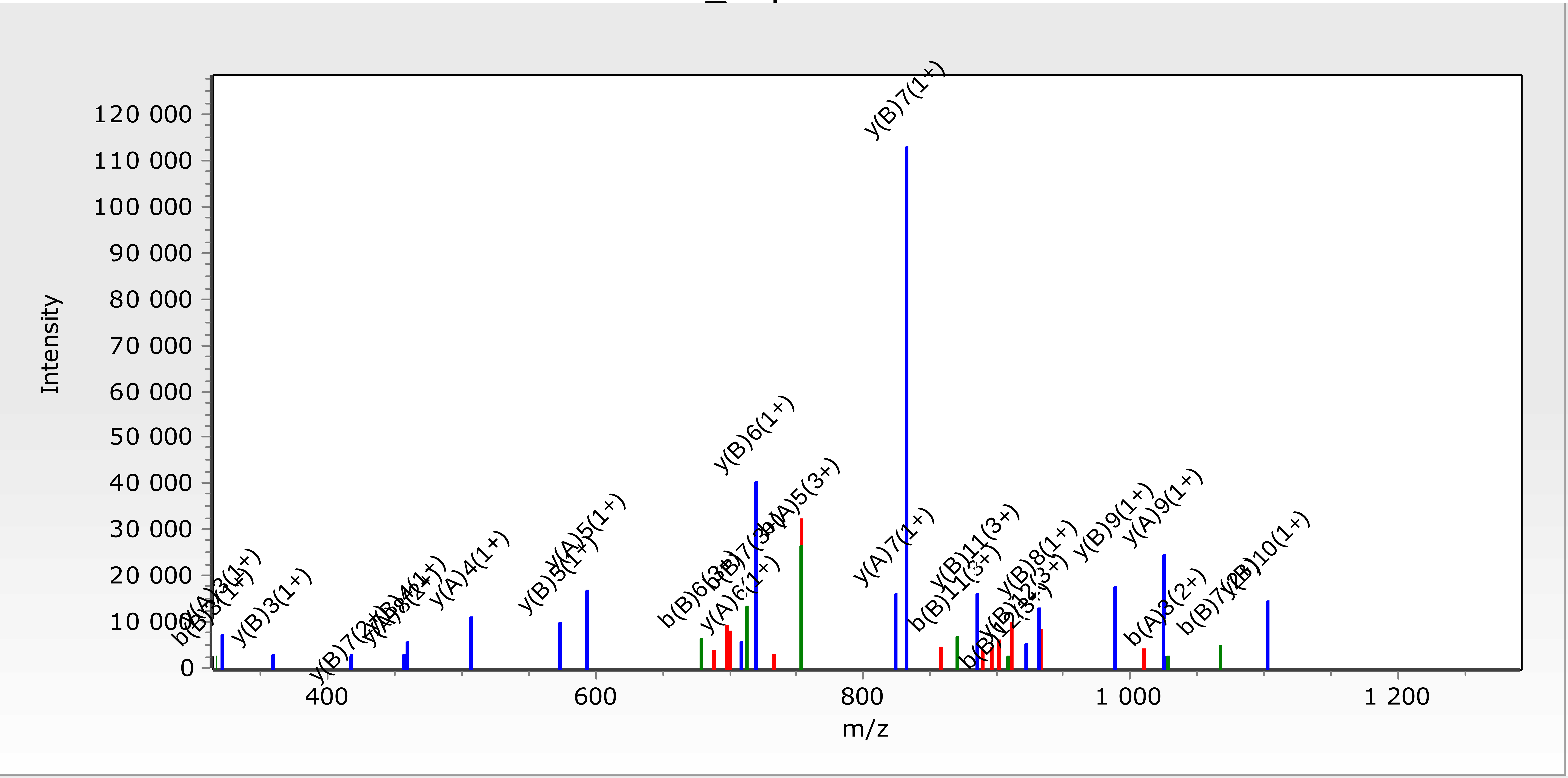

MSMS
KEDSDDSWSGRxAELKNGVLFITIPK 126x161 ^{14}Nx^{14}N
File: 150609_Hx1

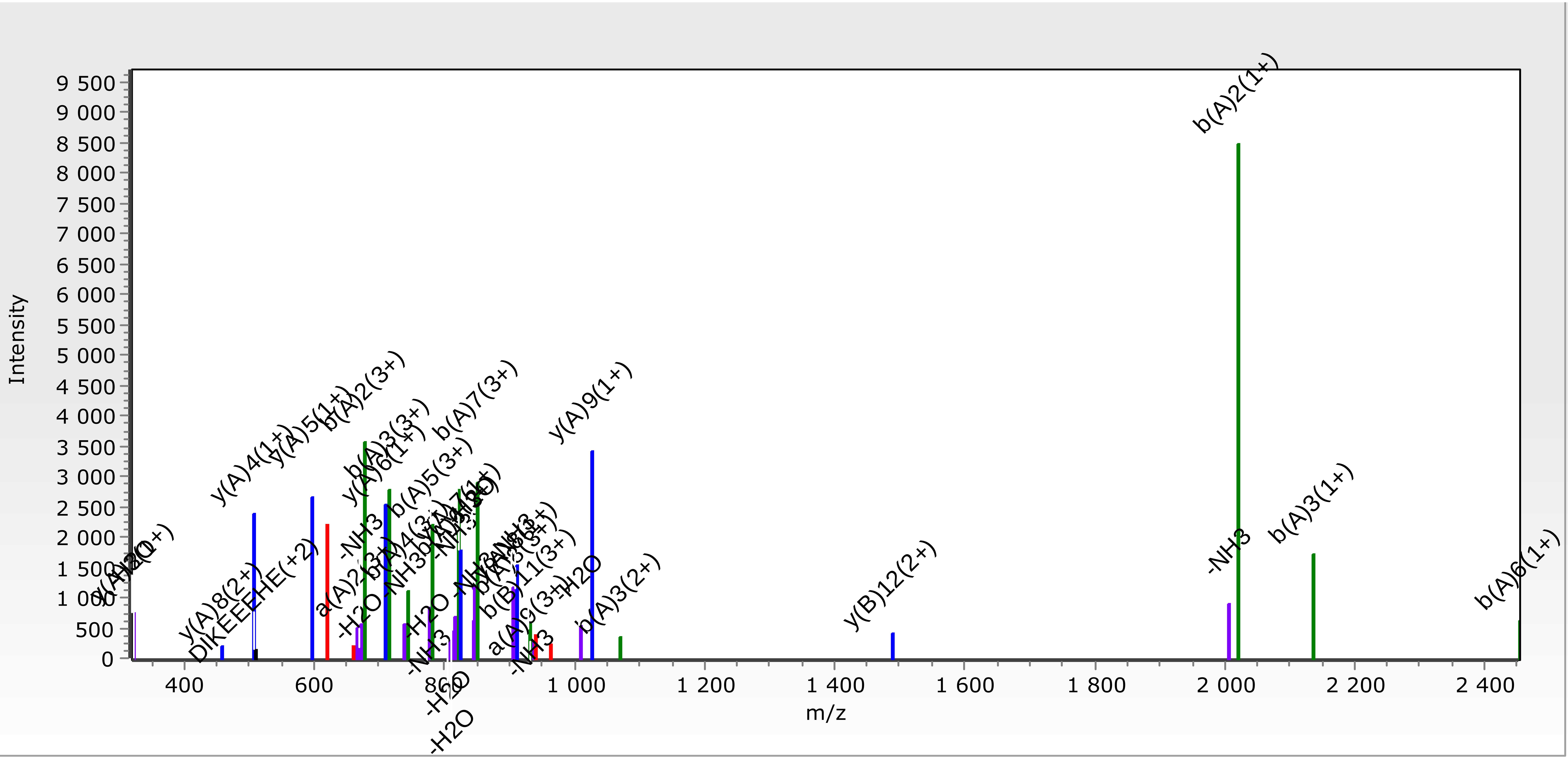

MS
KEDSDDSWSGRxAELKNGVLFITIPK 126x161
File: 150609_Hx1 rt: 1688

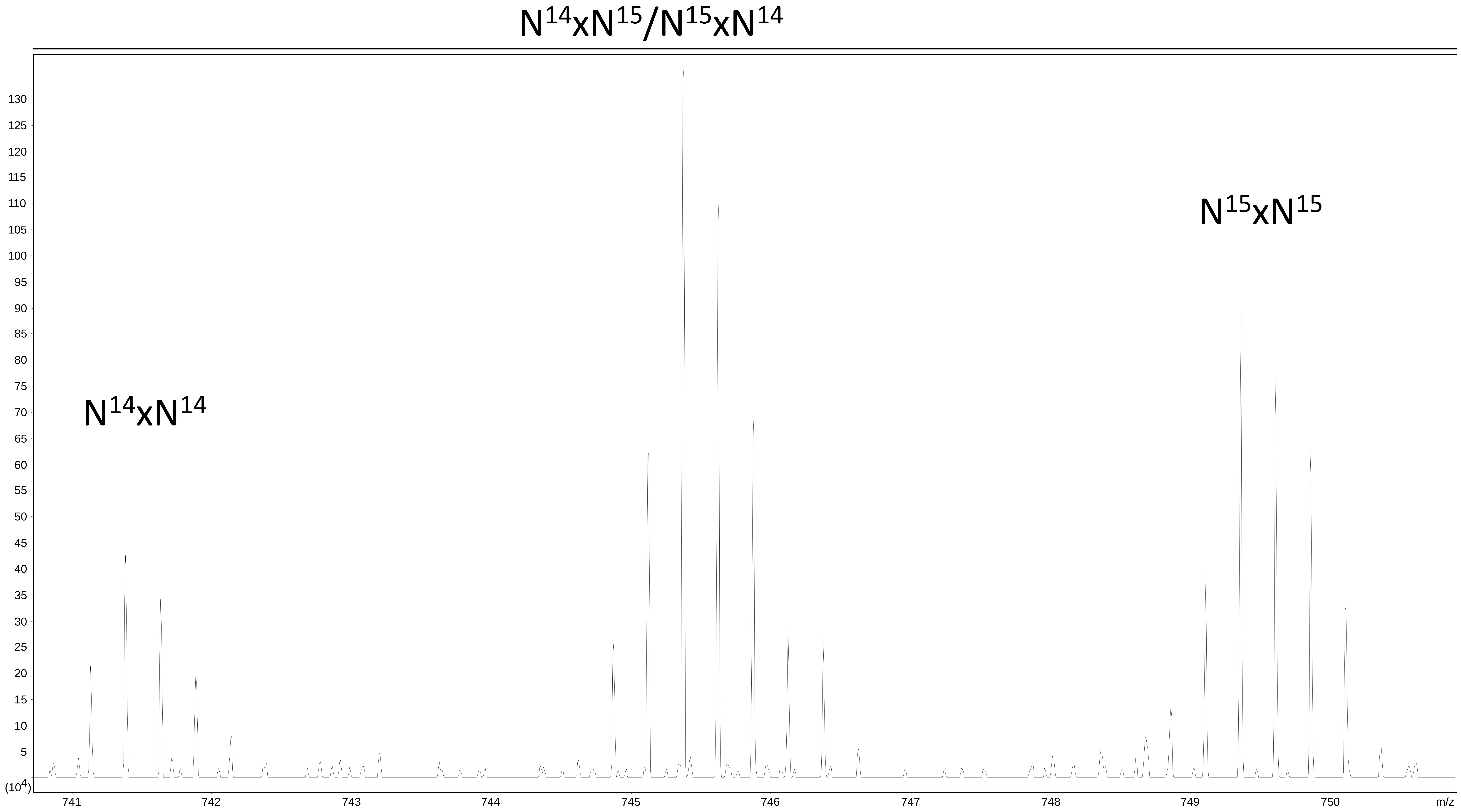

MSMS

KEDSDDSWSGRxAELKNGVLFITIPK 126x161 $N^{14}xN^{15}$

File: 150609_Hx5

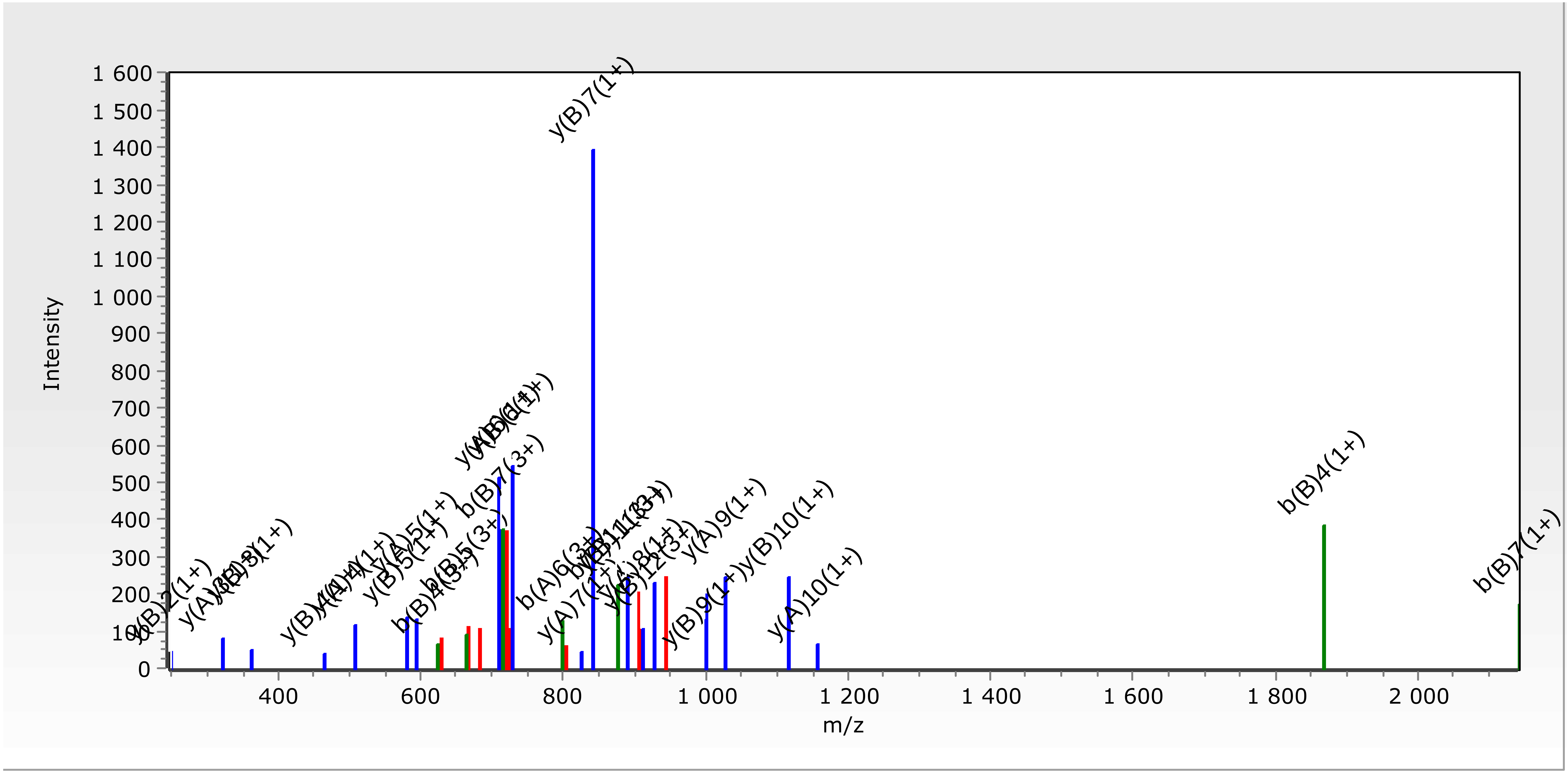

MS
KEDSDDSWSGRxAELKNGVLFITIPK 126x161
File: 150609_Hx5 rt: 1686

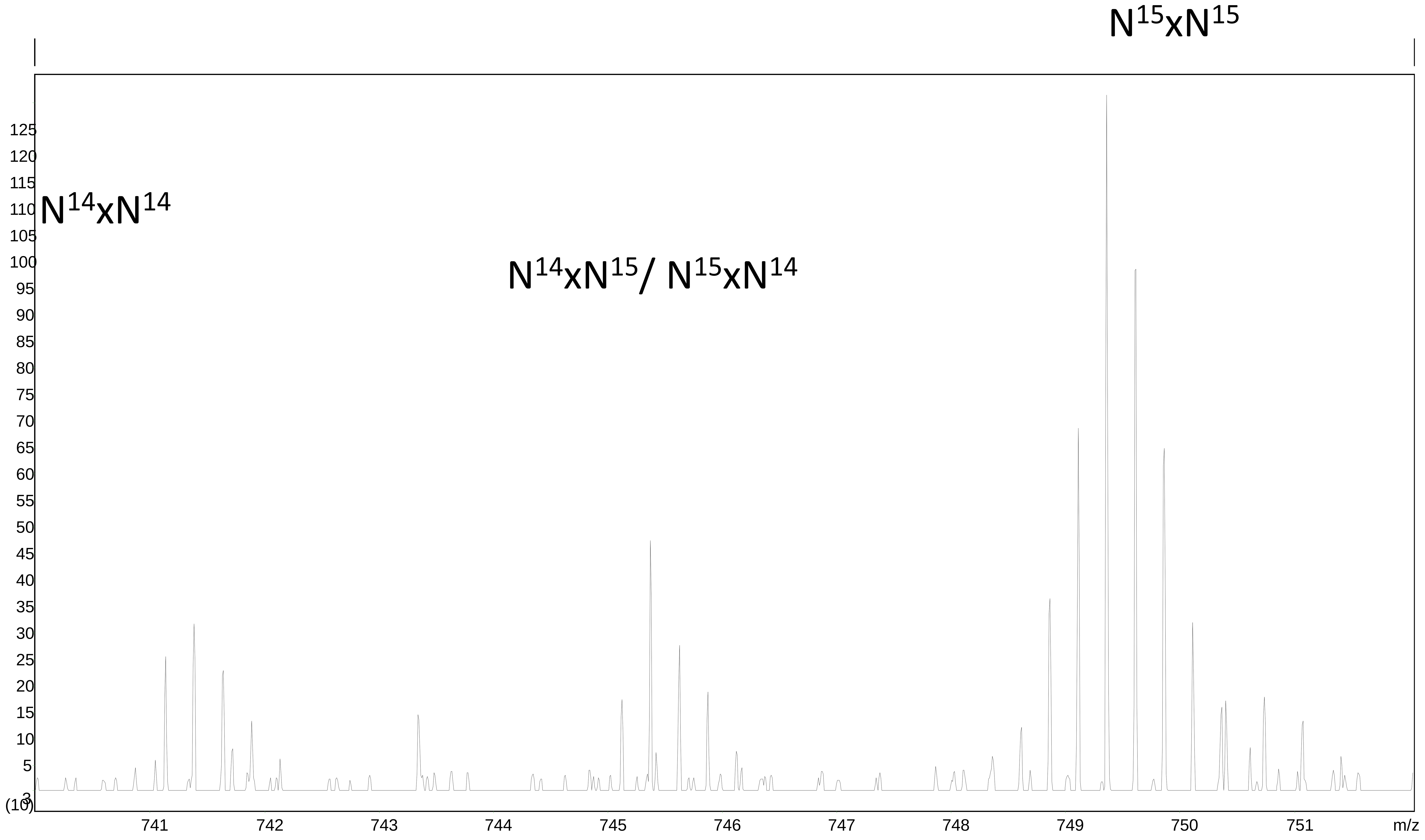

MSMS
LQLPDNCEKDKxIKAELK 153x157
File: 150521_Hsp21 monomeric band

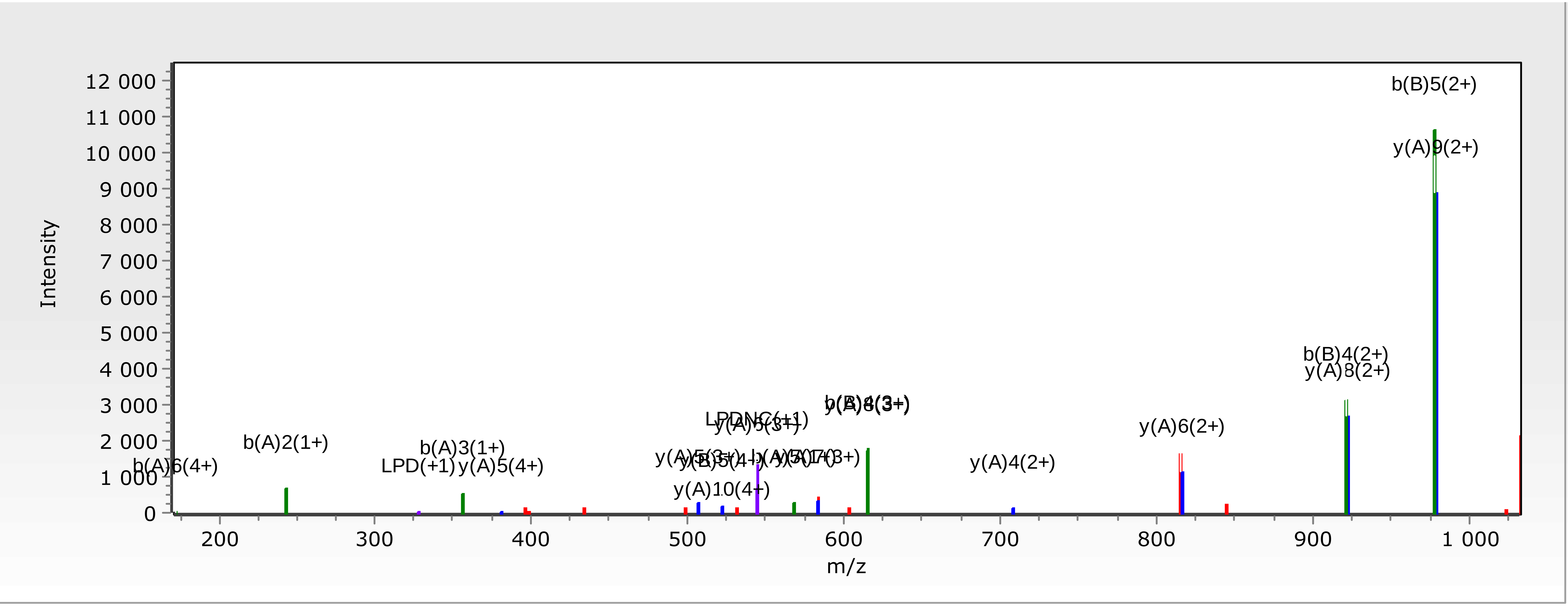

MSMS
IKAELKxTKVER 157x173
File: 160307_Hsp21 monomeric band

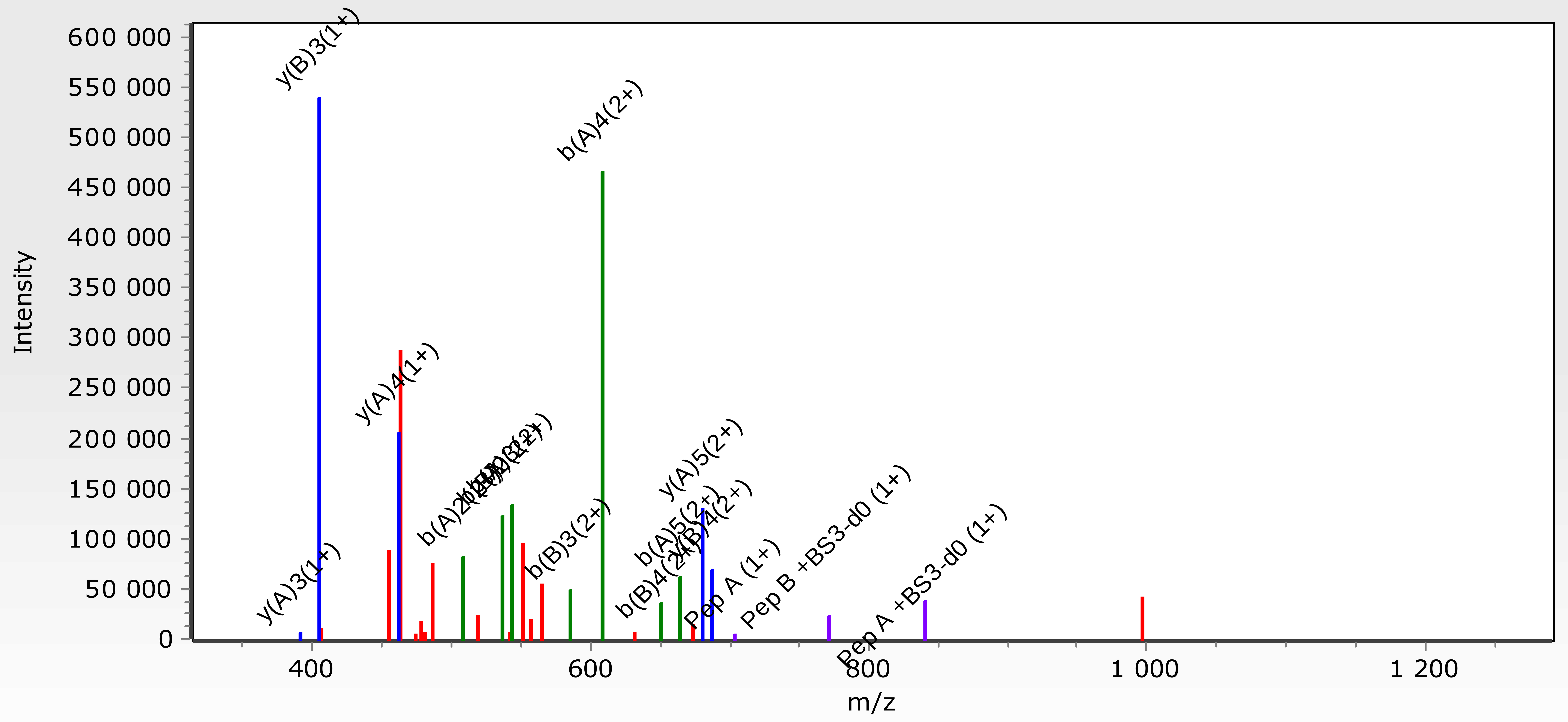

MSMS
IKAELKxTKVER 157x173
File: 160307 Hsp21 dimeric band

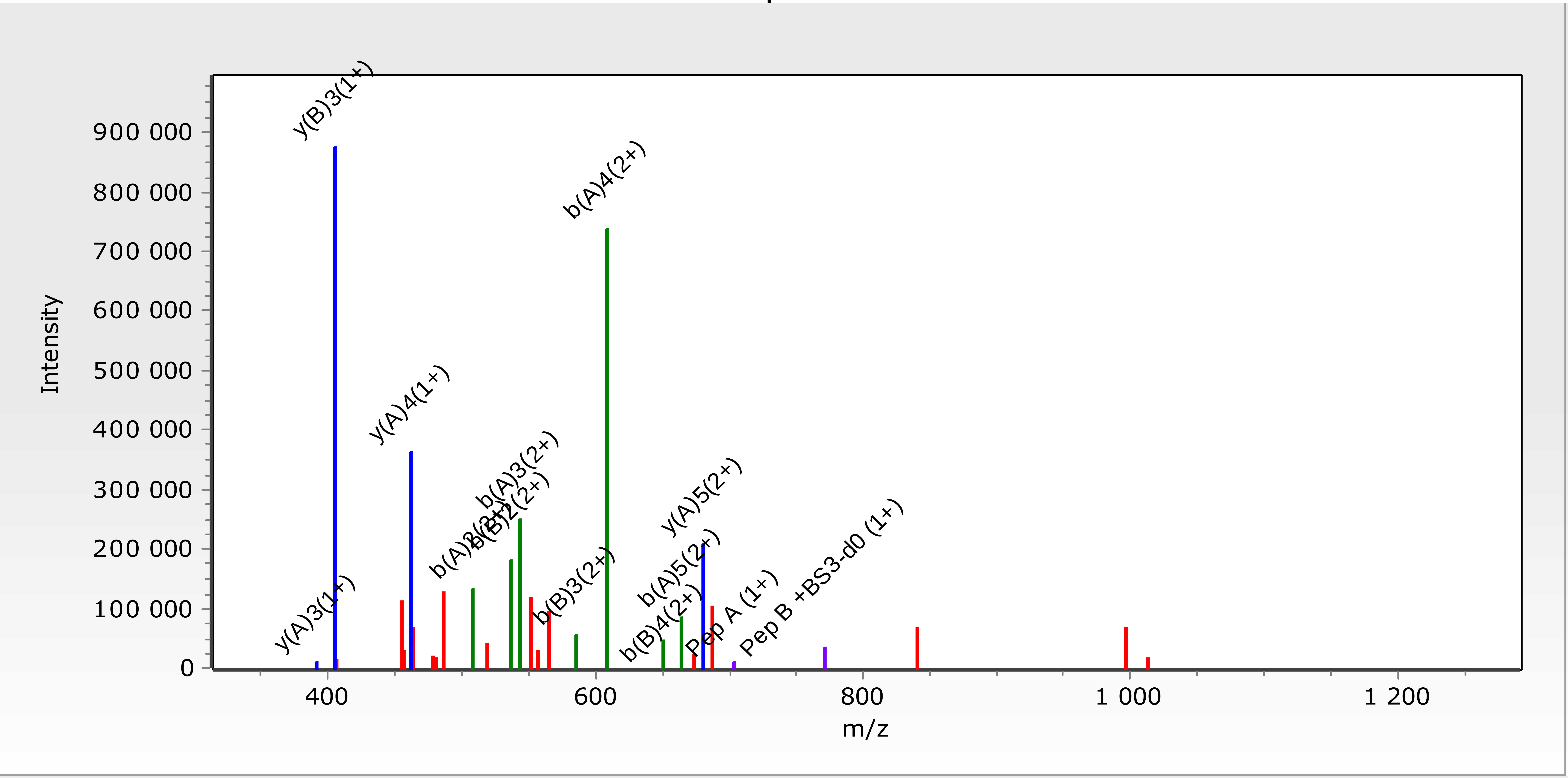

Supplement: Supplemental Data [file supp_M116.766816_Supplemental_information_5_MS_and_MSMS-spectra.zip › Supplemental information 5_MS and MSMS-spectra.pdf]
